# Coumarin C−H Functionalization by Mn(I) Carbonyls: Mechanistic Insight by Ultra‐Fast IR Spectroscopic Analysis

**DOI:** 10.1002/chem.202203038

**Published:** 2023-03-23

**Authors:** Thomas J. Burden, Kathryn P. R. Fernandez, Mary Kagoro, Jonathan B. Eastwood, Theo F. N. Tanner, Adrian C. Whitwood, Ian P. Clark, Michael Towrie, Jean‐Philippe Krieger, Jason M. Lynam, Ian J. S. Fairlamb

**Affiliations:** ^1^ Department of Chemistry University of York Heslington York YO10 5DD UK; ^2^ Central Laser Facility Research Complex at Harwell STFC Rutherford Appleton Laboratory Harwell Campus Didcot Oxfordshire OX11 0QX UK; ^3^ Syngenta Crop Protection AG Breitenloh 5 4333 Münchwilen Switzerland

**Keywords:** coumarin, cyclomanganation, infrared spectroscopy, manganese, organomanganese

## Abstract

Mn(I) C−H functionalization of coumarins provides a versatile and practical method for the rapid assembly of fused polycyclic pyridinium‐containing coumarins in a regioselective manner. The synthetic strategy enables application of bench‐stable organomanganese reagents in both *photochemical‐* and *thermal*‐promoted reactions. The cyclomanganated intermediates, and global reaction system, provide an ideal testing ground for structural characterization of the active Mn(I) carbonyl‐containing species, including transient species observable by ultra‐fast time‐resolved spectroscopic methods. The thermodynamic reductive elimination product, solely encountered from reaction between alkynes and air‐stable organometallic cyclomanganated coumarins, has enabled characterization of a critical seven‐membered Mn(I) intermediate, detected by time‐resolved infrared spectroscopy, enabling the elucidation of the temporal profile of key steps in the reductive elimination pathway. Quantitative data are provided. Manganated polycyclic products are readily decomplexed by AgBF_4_, opening‐up an efficient route to the formation of π‐extended hybrid coumarin‐pyridinium compounds.

## Graphical abstract

**Towards π‐extended hybrid coumarin‐pyridinium compounds**: We have developed a remarkable methodology for the synthesis of hybrid coumarin‐pyridinium compounds using Mn(I) carbonyl chemistry. The system was found suitable for forensic examination of the key steps underpinning CO loss, alkyne coordination and insertion, and subsequent reductive elimination, which were quantifiable by time‐resolved spectroscopic methods.

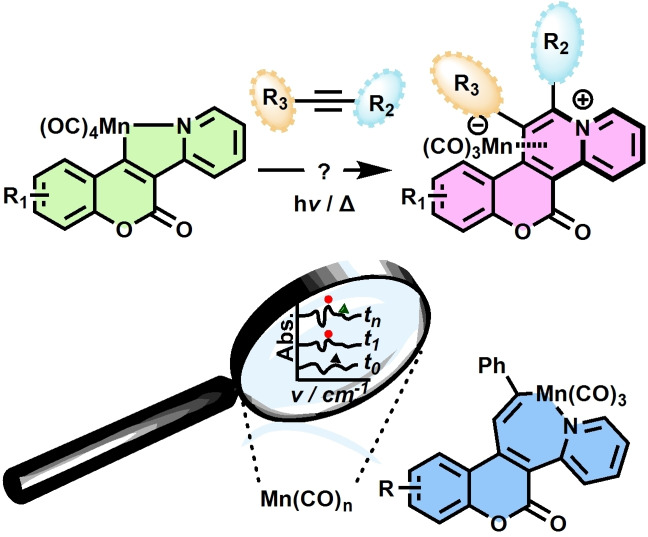

## Introduction

C−H activation and functionalization of organic molecules can provide a convenient and efficient method for the direct reaction of ubiquitous C−H sites. Transition metals have been widely applied in the C−H bond functionalization of a myriad of *O*, *S* and *N*‐containing heterocyclic compounds.^[1]^ Less focus has been placed on the biologically and medicinally‐relevant coumarin ring systems, and what has been achieved has been dominated by precious metals, for example Pd,[^2^, ^3^] Ru,^[4]^ Rh^[5]^ and Ir.^[6]^ The deployment of earth abundant metals for the functionalization of coumarins would represent an important step forward particularly from a from sustainability, environmental and metal‐security perspectives,^[7]^ with Satoh's recent work with precious and costly Rh highlighting the potential for diverse organic transformations.^[5]^ Manganese, a highly abundant transition metal, has emerged as a distinctive cost‐effective source for directed^[8]^ and non‐directed^[9]^ C−H bond functionalization reactions, exhibiting complementary and oftentimes differential reactivity to metals such as Pd.^[10]^ There can be no stronger argument for the employment of an earth abundant metal for a synthetic transformation if it is able to dictate the reaction outcome. The Mn(I)‐promoted C−H bond functionalization reactions of challenging heterocyclic substrates occur catalytically and stoichiometrically, as demonstrated by Ackermann,^[11]^ Wang,^[12]^ and Fairlamb and Lynam.^[13]^ The Mn‐catalyzed C−H bond functionalization reactions operate primarily via redox‐neutral processes. Alternative pathways were exemplified in the reaction of a 2‐pyrone derivative with [MnBr(CO)_5_] to give either alkenylated 2‐pyrone product **3 a’** or pyridinium adduct **3 a**, depending on the reaction conditions employed.^[15]^ This process revealed important mechanistic details that was directly translatable to archetypical 2‐aryl‐pyridine C−H bond functionalization processes. We anticipated that the reductive coupling pathway could be harnessed into a general synthetic route to access highly functionalized π‐conjugated chromeno‐pyridines/pyridinium salts, such as **5 a’** from coumarins such as **4‐(*7*‐NEt_2_)**. Related products exhibit an eclectic array of biological and medicinal properties,^[14]^ indeed coumarin‐pyridinium hybrid salts are active cholinesterase inhibitors.^[15]^

Crucially, we recognized that the coumarin ring system could confer stabilization of the transient intermediates formed en route to such products, enabling mechanistic details to be probed such as alkyne coordination, insertion and reductive elimination.

## Results and Discussion

**Figure 1:**
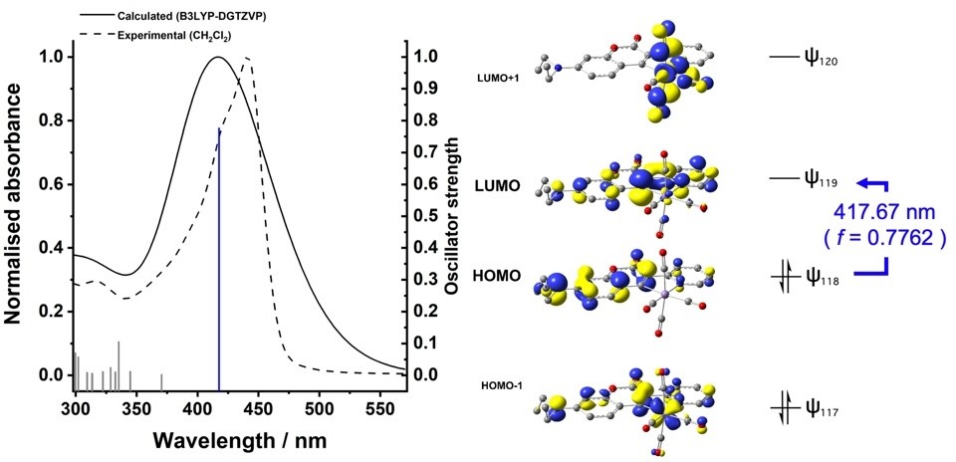
TD‐DFT calculations for complex **6‐(*7*‐NEt_2_)** highlighting the major transition (HOMO^ψ118^‐LUMO^ψ119^). Level of theory used ‐ B3LYP/DGTZVP/CPCM (CH_2_Cl_2_), 50 states (with empirical dispersion correction applied, GD3).

Ultra‐fast time‐resolved spectroscopy is a powerful tool to study these processes experimentally,[^13a^, ^13c^, ^13d^, ^13e^, ^13f^] and CO‐photodissociation provides a useful pathway to activate the complexes to study their interactions with the solvent and substrates of the reaction. The central hypothesis that these manganese complexes may undergo light‐induced CO dissociation was probed by time‐dependent density functional theory (TD‐DFT) calculations for novel cyclomanganated complex **6‐(*7*‐NEt_2_)** (which could be formed preparatively from **4‐(*7*‐NEt_2_)** by reaction with [BnMn(CO)_5_], in a quantitative manner). A minimized structure for **6‐(*7*‐NEt_2_)** was computationally identified using DFT methods through optimization in Gaussian16 using the B3LYP global hybrid functional and flexible DGTZVP basis set, along with Grimme's dispersion‐correction for improved long‐range dispersion energies (GD3), and a conductor‐like polarizable continuum model (CPCM) CH_2_Cl_2_ solvent model. These calculations are in keeping with those which we have conducted on closely related cyclomanganated compounds.^[16]^ For the subsequent TD‐DFT calculations, we assessed several functionals (B3LYP, cam‐B3LYP, BP86 and PBE0) employing the DGTZVP basis set, which allowed us to compare the experimental UV‐vis spectrum for **6‐(*7*‐NEt_2_)** in CH_2_Cl_2_ at 298 K with the computed UV‐vis spectral data. Thus, the data set best showing a match with the experimental data was found by B3LYP/DGTZVP. We calculated 50 low‐lying valence excited states. The TD‐DFT predicted and experimental UV‐vis spectra for **6‐(*7*‐NEt_2_)** are shown in Figure 1 (left‐hand side). The latter shows a strong UV‐vis absorption band *λ*_max_=444 nm, with a slight shoulder at ca. 420 nm. A broader, but slightly higher energy calculated dominant band was determined by TD‐DFT, the first lowest energy excited state being the HOMO_118_‐LUMO_119_ interaction with large oscillator strength (*f*=0.7762) and of singlet character. The HOMO is primarily centered on the coumarin framework, connecting the electron‐rich atom sites within the diethylamino‐substituted coumarin system. The LUMO is found across the ligand and manganese(I) center. An axial CO ligand is weakened in the LUMO suggesting that structure **6‐(*7*‐NEt_2_)** could be excited by light to enable photodissociation in the presence of a suitable and reactive alkynyl substrate.

**Scheme 1:**
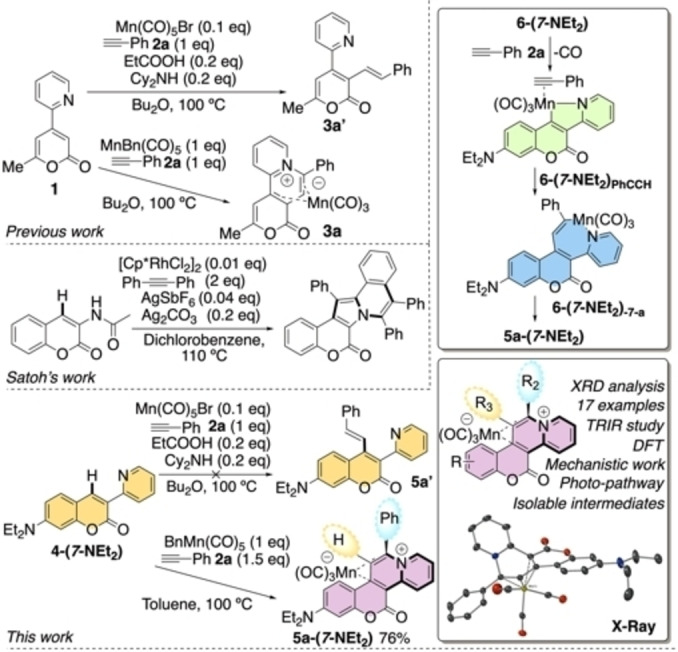
Manganese(I)‐promoted C−H activation and functionalization, The X‐ray diffraction (XRD) structure of **5 a‐(*7*‐NEt_2_)** is given (center right, ellipsoids set at 50 % probability; H‐atoms omitted). Inset: proposed species forming on route to **5 a‐(*7*‐NEt_2_)**.

In order to investigate the ability of Mn‐carbonyl complexes to functionalize the desired substrates, reaction of coumarin **4‐(*7*‐NEt_2_)** with phenylacetylene **2 a** in the presence of 10 mol % [MnBr(CO)_5_] (Scheme 1) was primarily found to not lead to the formation of alkenylated coumarins **5 a’**. Analysis of the resulting reaction mixture indicated that **5 a‐(*7*‐NEt_2_)** as the only product, which is structurally related to **3 a** (only trace amounts formed). The synthesis of **5 a‐(*7*‐NEt_2_)** was optimized so that it could also be prepared in 76 % yield from a reaction between [MnBn(CO)_5_] in toluene at 95 °C giving cyclometalated **6‐(*7*‐NEt_2_)** in quantitative yield (Scheme 1).

**Scheme 2:**
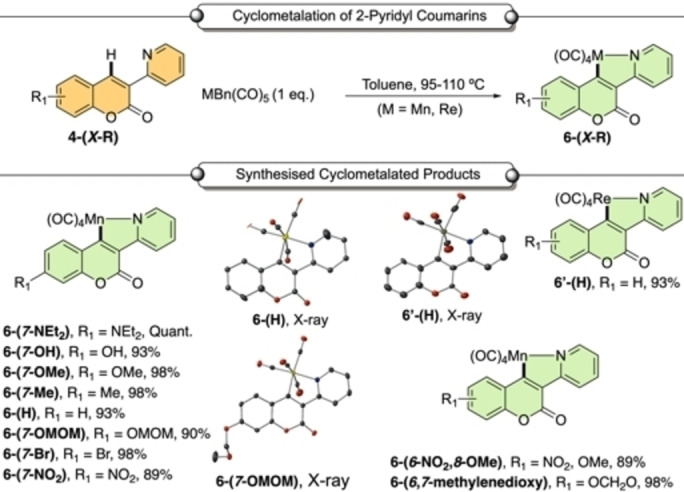
Cyclomanganation of functionalized coumarins. Reaction conditions: coumarin **4‐(*X*‐R)** (1.0 equiv.), [MBn(CO)_5_] (1.0 equiv.) toluene, (6 mL), N_2_, 95–110 °C, 2.5 h. Yields of isolated products. MOMO=methoxymethyl. Selected X‐ray diffraction structures are shown.

It was anticipated that this reaction proceeded through an initial cyclomanganation reaction between **4‐(*7*‐NEt_2_)** and [MnBn(CO)_5_] to give **6‐(*7*‐NEt_2_)**, followed by subsequent reaction with alkyne phenylacetylene **2 a** to give **5 a‐(*7*‐NEt_2_)**. A stoichiometric reaction between **4‐(*7*‐NEt_2_)** and [MnBn(CO)_5_] gave manganacycle **6‐(*7*‐NEt_2_)** in quantitative yield (Scheme 2). We determined that a series of coumarin derivatives **4‐(*X*‐R)** could be employed in this reaction giving the corresponding manganacycles **6‐(*X*‐R)** in high yields (Scheme 2).

**Scheme 3:**
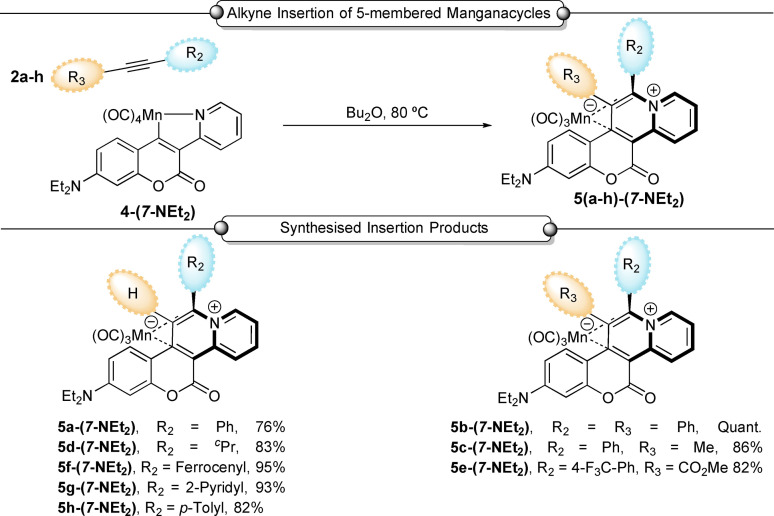
Alkyne reactivity towards manganacycles. Reaction conditions: alkyne **2 a**–**h** (1.2 equiv.), **6‐(*7*‐NEt_2_)** (1.0 equiv.), Bu_2_O, (6 mL), N_2_, 80 °C, 18 h. Isolated yields.

Consistent with the proposed mechanistic picture, reaction of **6‐(*7*‐NEt_2_)** with phenylacetylene **2 a**, afforded **5 a‐(*7*‐NEt_2_)**. As shown in Scheme 3, this method could be applied to a series of terminal and internal alkynes (**2 a**‐**h**) to afford complexes **5(a–h)‐(*7*‐NEt_2_)**. The products were obtained in a highly selective manner, with the regiochemical outcome, with respect to the unsymmetrical alkyne, being corroborated by single crystal X‐ray diffraction (XRD). The formation of ferrocene‐ and pyridine‐containing products (**5 f‐(*7*‐NEt_2_)** and **5 g‐(*7*‐NEt_2_)**, respectively) confirms the high tolerance of this protocol to potentially sensitive redox active and metal‐coordinating moieties, respectively.

Attempts to extend this reaction to other cyclomanganated coumarin derivatives revealed the importance of the diethylamino substituent in **6‐(*7*‐NEt_2_)** in aiding the formation of **5 a‐(*7*‐NEt_2_)** under our standard reaction conditions. For example, when alterative substituents were introduced into the coumarin framework (Table 1 entries 2–6) the product yield was reduced. This potential issue was circumvented by addition of 1 equivalent of TMNO (trimethylamine *N*‐oxide) to the reaction. The intervention by TMNO induces CO‐loss from the tetracarbonyl manganese(I) compound enabling *η* ^2^‐alkyne coordination,^[17]^ which is a finding that might be more broadly useful in Mn(I) C−H functionalization chemistry. Subsequent migratory insertion of the alkyne into the Mn−C bond, and final reductive elimination, affords novel complexes **5 a‐(*X*‐R)** (Table 1).

**Table 1:** Effect on yields due to coumarin functional group and in the presence of TMNO additive.

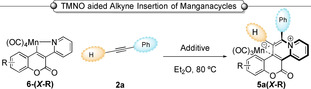				
Entry	Compound	TMNO [equiv.]	Product	Yield [%]
1	**6‐(*7*‐NEt_2_)**	0	**5 a‐(*7*‐NEt_2_)**	76
2	**6‐(*7*‐OH)**	0	**5 a‐(*7*‐OH)**	4
3	**6‐(*7*‐OMe)**	0	**5 a‐(*7*‐OMe)**	45
4	**6‐(H)**	0	**5 a‐(H)**	16
5	**6‐(*7*‐Me)**	1.0	**5 a‐(*7*‐Me)**	26
6	**6‐(*7*‐OMOM)**	0	**5 a‐(*7*‐OMOM)**	16
7	**6‐(*7*‐OH)**	1.0	**5 a‐(*7*‐OH)**	94
8	**6‐(*7*‐OMe)**	1.0	**5 a‐(*7*‐OMe)**	90
9	**6‐(H)**	1.0	**5 a‐(H)**	88
10	**6‐(*7*‐Br)**	1.0	**5 a‐(*7*‐Br)**	36
11	**6‐(*7*‐NO_2_)**	1.0	**5 a‐(*7*‐NO_2_)**	63
12	**6‐(*6***, ***7*** **‐methylenedioxy)**	1.0	**5 a‐(*6***, ***7*** **‐methylenedioxy)**	91
13	**6‐(*6*‐NO_2_**, ***8*** **‐OMe)**	1.0	**5 a‐(*6*‐NO_2_**, ***8*** **‐OMe)**	71

As dissociation of CO from Mn(I) complexes such as **6 a‐(*X*‐R)** can be promoted photochemically,^[13]^ it was proposed that **5 a‐(*7*‐NEt_2_)** could form from **6‐(*7*‐NEt_2_)** and **2 a** on exposure to light. This suggestion was supported by our TD‐DFT calculations. A reaction between **6‐(*7*‐NEt_2_)** and **2 a** was performed under irradiation (focussed LED, 355 nm) at room temperature in diethyl ether. This synthetic protocol afforded **5 a‐(*7*‐NEt_2_)** in 66 % yield, after 35 min of irradiation (see the Supporting Information for the reaction time course).

Analysis of the solid‐state structures of complexes **5 a‐(*X*‐R)**, determined by single crystal X‐ray diffraction, demonstrated that the manganese was *η* ^4^‐coordinated to the newly‐formed fused ring system. This resulted in a deviation in anticipated sp^2^ planarity of the fused 6‐membered ring derived from the alkyne and 2‐pyridyl fragments. This effect also underpins the observed upfield shifts in the ^13^C{^1^H} NMR spectrum of **5 a‐(*7*‐NEt_2_)**, with the four dienyl carbon environments appearing at *δ* 108.4, 102.8, 90.2 and 72.8 ppm.

**Figure 2:**
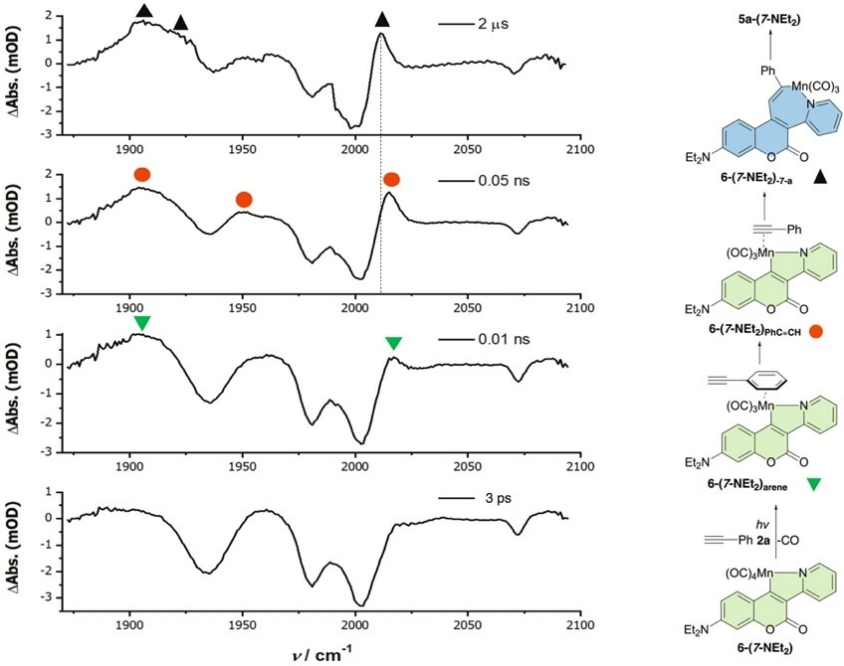
TR^M^PS‐IR data for the reaction between **6‐(*7*‐NEt_2_)** and **2 a** in neat alkyne **2 a**.

Further experiments were performed to gain mechanistic insight into the steps which underpin the formation of **5 a‐(*7*‐NEt_2_)**. Given that synthetic work had indicated that thermal (including TMNO‐induced CO‐loss) or photochemical loss (focused LED, 355 nm) of CO from **6‐(*7*‐NEt_2_)** was a key step in this reaction, the interaction between **6‐(*7*‐NEt_2_)** and **2 a** was studied employing Time‐Resolved Multiple‐Probe Spectroscopy (TR^M^PS) with IR detection using the state‐of‐the‐art LIFEtime facility at the Central Laser Facility (UK).^[18]^ These experiments were conducted through irradiation of the reaction mixture with ultra‐fast laser pulses (*λ*=355 nm) to induce CO‐loss. The nature and behavior of the resulting photoproducts were then studied by observing differences in the infrared spectrum between 1850–2100 cm^−1^, over pump‐probe delays between 1 ps and 1 ms.^[10]^ The resulting data are presented in Figure 2 as difference spectra, with negative peaks representing **6‐(*7*‐NEt_2_)** being consumed (bleached peaks) upon photolysis, and the positive peaks belonging to the new photoproducts.

Photoirradiation of **6‐(*7*‐NEt_2_)** and phenylacetylene **2 a** in MeCN solution resulted in the detection of one photoproduct **6‐(*7*‐NEt_2_)_MeCN_**, from which no subsequent species were formed. Repeating the reaction with **6‐(*7*‐NEt_2_)** in neat **2 a** showed that positive peaks appeared after 10 ps, which on the basis of our previous work,[^13a^, ^13f^] were assigned to arene‐coordinated **6‐(*7*‐NEt_2_)_arene_** complex. Over the course of 50 ps the bands for **6‐(*7*‐NEt_2_)_arene_** decreased in intensity, being replaced by those for the π‐bound *η* ^2^‐alkyne complex **6‐(*7*‐NEt_2_)_PhC≡CH_**. The observed rate constant for this process was determined (*k*_obs_=(5.83±0.44) ×10^10^ s^−1^). Remarkably, it was then possible to observe the subsequent C−C bond formation step corresponding to the migratory insertion of the alkyne into the Mn−C bond. This resulted in the formation of **6‐(*7*‐NEt_2_)_‐7‐a_** in ca. 2 μs, which is evidenced by the red shift in the frequencies of the manganese(I) carbonyl stretches associated with loss of the π‐accepting ligand to the 7‐membered manganacycle.^[10]^ The observed rate constant for the insertion process was determined (*k*_obs_=(5.62±1.91) ×10^5^ s^−1^). These data demonstrated that the reaction of alkyne and coumarin ligands is fast (<10 μs), however, no evidence for any additional photoproducts was obtained for the remainder of the experiment (to a timescale of 1 ms), indicating that the reductive elimination reaction to form **5 a‐(*7*‐NEt_2_)** is significantly slower.

**Scheme 4:**
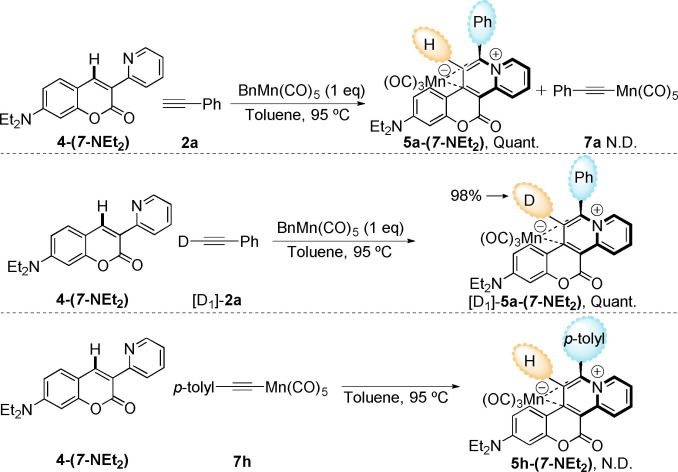
Study of potential reaction intermediates and deuterium incorporation.

**Scheme 5:**
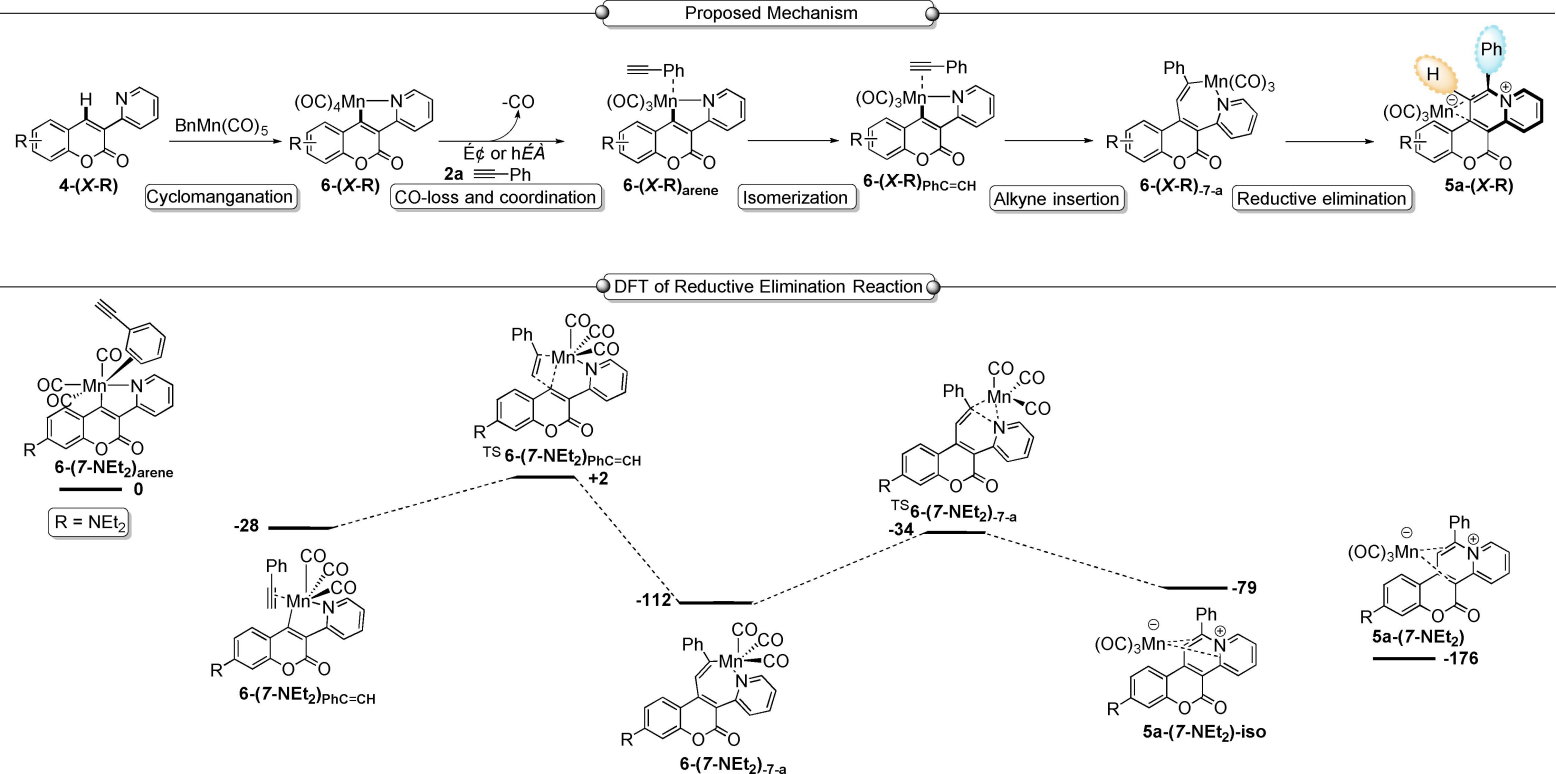
Upper: Mechanistic hypothesis informed by experimental work. Lower: DFT calculations showing the feasibility of reductive elimination from **6‐(*7*‐NEt_2_)** to **5 a‐(*7*‐NEt_2_)**, starting from intermediates **6‐(*7*‐NEt_2_)_arene_**. DFT calculations were performed at the pbe0/def2‐TZVPP//bp86/SV(P)level of theory, with a COSMO implicit solvent model (toluene) and Grimme's 3^rd^ empirical dispersion correction. The energies displayed are the Gibbs energies at 298.15 K in kJ mol^−1^.

Further experiments were conducted to shed light on the reaction mechanism. A one‐pot reaction with coumarin **4‐(*7*‐NEt_2_)** in the presence of both an equivalent amount of [BnMn(CO)_5_] and **2 a**, delivered **5 a‐(*7*‐NEt_2_)** in quantitative yield (Scheme 4). This demonstrated that formation of **6‐(*7*‐NEt_2_)** was not competitive compared to the *η* ^1^‐alkynyl species [Mn(CO)_5_(C≡CPh)] **7 a**, which was not detected under the reaction conditions. An analogous reaction using deuterated phenylacetylene [D_1_]‐**2 a** delivered [D_1_]‐**5 a‐(*7*‐NEt_2_)** in quantitative yield (Scheme 5). The observation that [D_1_]‐**5 a‐(*7*‐NEt_2_)** featured 98 % preservation of deuteration at the carbon atom derived from the terminal alkyne indicates that no [Mn(CO)_5_(C≡CPh)] was formed from **2 a** and [BnMn(CO)_5_] under these reaction conditions. Initial C−H bond activation is therefore proposed to occur at the electrophilic C−H site within the coumarin ring. Consistent with this observation is the reaction of [Mn(CO)_5_(C≡CC_6_H_5_‐4‐Me) with **4‐(*7*‐NEt_2_)** which did not afford any **5 h‐(*7*‐NEt_2_)**.^[8c]^ **5 h‐(*7*‐NEt_2_)** was prepared in 82 % yield using the route in Scheme 4 to prove its viability. These data demonstrate that *η* ^1^‐alkynyl species, including **7 a** and **7 h**, are not active species in this reaction. This observation stands in contrast to the coupling of **2 a** with 2‐phenylpyridine.^[13b]^

**Figure 3:**
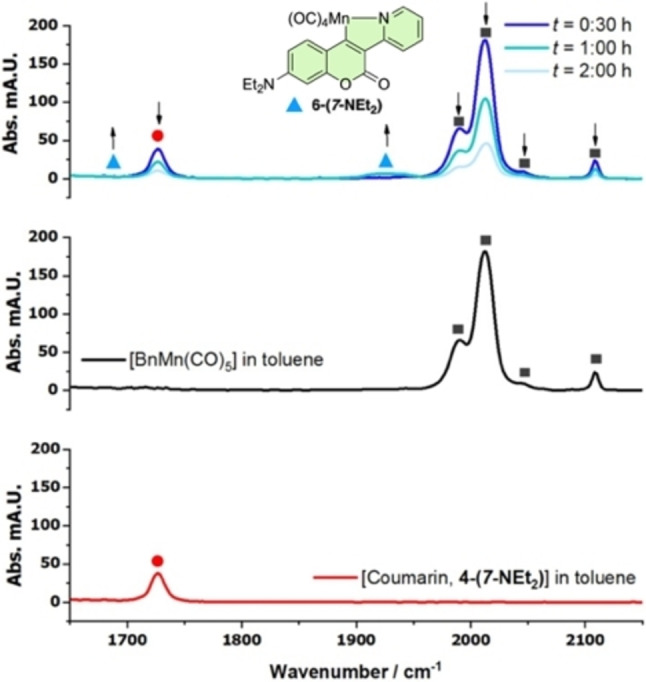
The in situ IR spectroscopic (ReactIR, Si‐Probe) data for the reaction between coumarin **4‐(7‐NEt_2_)**, [BnMn(CO)_5_] and phenylacetylene **2 a** in toluene, 60 °C to 2 h (middle and bottom: IR reference spectra; top: reaction monitoring).

A one‐pot experiment confirmed consumption of the coumarin **4‐(*7*‐NEt_2_)** in a reaction with [BnMn(CO)_5_] to give **6‐(*7*‐NEt_2_)**, as monitored by in situ IR using a Mettler‐Toledo ReactIR instrument, fitted with a Si probe. The spectral changes could be easily monitored on the second timescale, which is a clean process (Figure 3).

A proposed outline mechanism is shown in Scheme 5. Both thermal and photochemically induced reactions begin with loss of a CO ligand from **6‐(*7*‐NEt_2_)** resulting in the generation of a vacant coordination site that is subsequently coordinated by alkyne **2 a** to give **6‐(*7*‐NEt_2_)_PhC≡CH_**.^[10a]^ The alkyne then inserts into the Mn−C bond delivering the 7‐membered manganacycle **6‐(*7*‐NEt_2_)_‐7‐a_**. These steps were observed directly in the TR^M^PS experiments. The reaction is then completed by reductive elimination via ring closure giving **5 a‐(*7*‐NEt_2_)**.

To support the mechanistic hypothesis, the steps leading up to the formation of **5 a‐(*7*‐NEt_2_)** were studied by computation using DFT calculations (Scheme 5, see Supporting Information for details of the methodology used). The spectroscopically observed arene‐coordinated complex **6‐(*7*‐NEt_2_)_arene_**, formed through loss of a CO from **6‐(*7*‐NEt_2_)** and coordination of **2 a**, was taken as the reference state for the calculations. Insertion of **2 a** into the Mn−C(coumarin) bond proceeds via a low energy transition state (^**TS**^ **6‐(*7*‐NEt_2_)_PhC≡CH_**) to give **6‐(*7*‐NEt_2_)_‐7‐a_**, a process corresponding to that observed by TR^M^PS see above. Complex **5 a‐(*7*‐NEt_2_)** is then subsequently proceeding via transition state (^**TS**^ **6‐(*7*‐NEt_2_)_‐7‐a_**). A dynamic reaction coordinate (DRC) analysis revealed that (^**TS**^ **6‐(*7*‐NEt_2_)_‐7‐a_**) connects **6‐(*7*‐NEt_2_)_‐7‐a_** with **6‐(*7*‐NEt_2_)‐iso**; the latter is a coordination isomer of **6‐(*7*‐NEt_2_)**. It is presumed that **6‐(*7*‐NEt_2_)‐iso** undergoes a low energy π‐slip to give the final isolatable product **6‐(*7*‐NEt_2_)**. The DFT calculations also predict that the energetic span for the formation of **6‐(*7*‐NEt_2_)‐iso** from **6‐(*7*‐NEt_2_)_‐7‐a_** is much greater (78 kJ mol^−1^) than for the alkyne insertion process through (^**TS**^ **6‐(*7*‐NEt_2_)_PhC≡CH_**) (30 kJ mol^−1^), consistent with the former step being too slow to be observed by TR^M^PS (e. g., millisecond range).

With both the synthetic and mechanistic chemistry underpinning the formation of **6‐(*7*‐NEt_2_)** fully established, attempts were then made to remove the fused cationic ring system from the formally anionic tricarbonyl manganese.

**Scheme 6:**
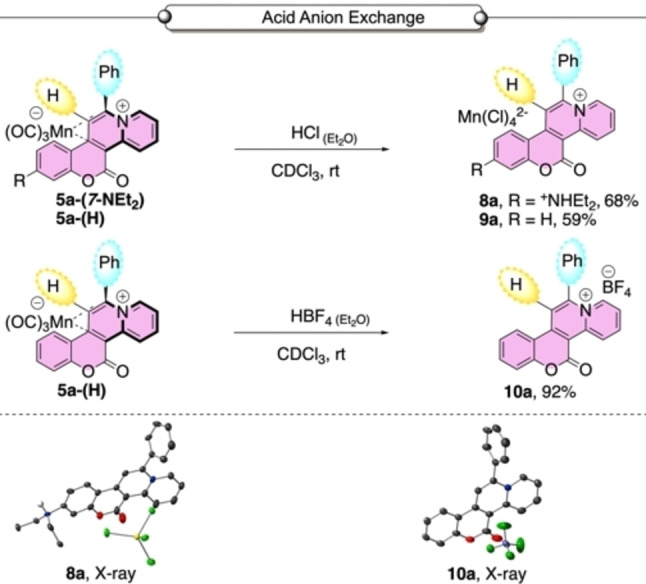
Decoordination of **5 a‐(*7*‐NEt_2_)** and **5 a‐(H)** under acidic conditions. The X‐ray structure of **8 a** and **10 a** are given (ellipsoids set at 50 % probability; H‐atoms omitted, except H‐atom on ammonium nitrogen in **8 a**).

Our first synthetic attempt involved treatment of **6‐(*7*‐NEt_2_)** with HCl in diethyl ether, which delivered **8 a** as the tetrachloromanganate(II) salt, where the diethylamino group had been protonated (Scheme 6). Single crystal XRD analysis confirmed the structural connectivity of **8 a**.

Treatment of **5 a‐(H)** under analogous conditions afforded the tetrachloromanganate(II) salt **9 a**. An alternative reaction of **5 a‐(H)** with HBF_4_ in diethyl ether afforded the tetrafluoroborate salt **10 a**, which was confirmed by single crystal XRD analysis (Scheme 6). A simplified procedure enabled direct formation of compounds **10 a‐13 a** on reaction of **5 a‐(*X*‐R)** with AgBF_4_.^[19]^ This delivered the corresponding BF_4_ salts of these cycloadducts (Table 2).

**Table 2:** Yields of BF_4_ salts from reaction with AgBF_4_.

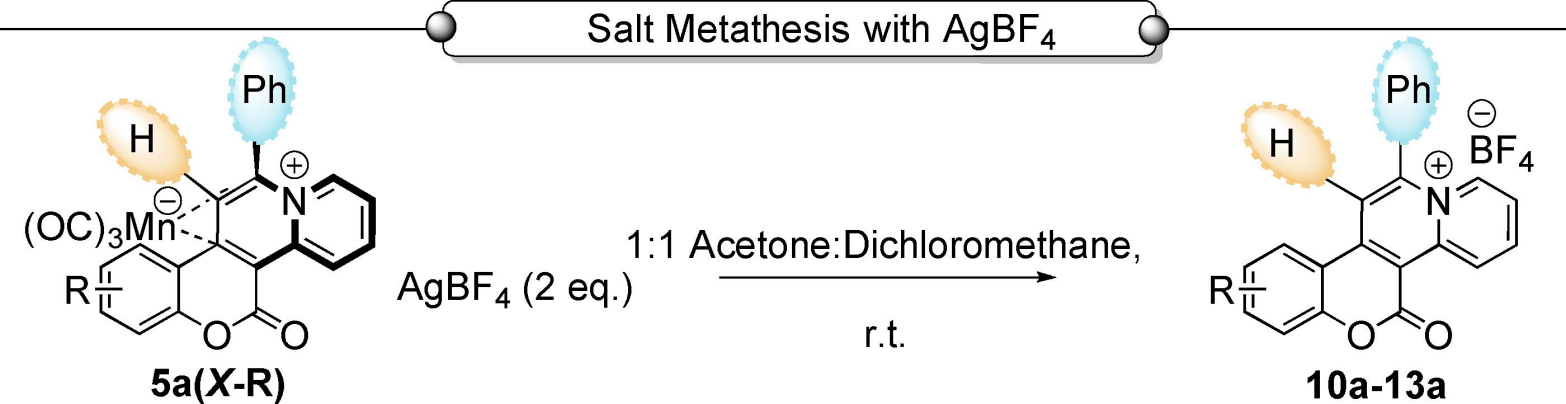				
Entry	Compound	AgBF_4_ [equiv.]	Product	Yield [%]
1	**5 a‐(H)**	2	**10 a**	83
2	**5 a‐(*7*‐NEt_2_)**	2	**11 a**	99
3	**5 a‐(*6*‐NO_2_**, ***8*** **‐OMe)**	2	**12 a**	98
4	**5 a‐(*7*‐Me)**	2	**13 a**	70

A literature survey revealed that there are scant reports on cyclorhenated structural variants, both in terms of the C−H bond activation step and an assessment of the feasibility of subsequent migratory insertion/reductive elimination steps. A reaction of benzyl rhenium(I) pentacarbonyl with **4‐(H)** afforded cyclorhenated complex **6’‐(H)**, using a higher reaction temperature of 110 °C (Scheme 2).^[20]^ No product was detected from the reaction of **6’‐(H)** with phenylacetylene **2 a**, under a variety of conditions (thermal‐TMNO aided and photochemical). The result is in keeping with the reduced propensity for rhenium to act as an effective catalyst, highlighting the uniqueness of manganese in facilitating these transformations.[^21^, ^22^]

## Conclusion

A useful synthetic methodology for accessing hybrid coumarin‐pyridinium compounds has been devised using Mn(I) carbonyl chemistry. It was possible to selectively remove the functionalized coumarin from the coordination sphere of the manganese(I) center, providing facile and efficient access to structurally diverse and potentially high‐value π‐conjugated hybrid coumarin‐pyridinium compounds in high yields. The coumarin scaffold was found suitable for forensic examination of the key steps underpinning CO loss, alkyne coordination and insertion, and subsequent reductive elimination at a manganese(I) carbonyl moiety. All steps were quantifiable by time‐resolved IR spectroscopic analysis. Furthermore, we have detected and characterized a coumarin‐based 7‐membered manganacycle which acts as an integral intermediate towards the reductive elimination products characterized in this work **5 a‐(*X*‐R)** and **5(a–h)‐(*7*‐NEt_2_)**. Our results, taken together, show the potential for developing new synthetic methodologies in concert with physical organometallic chemistry‐based measurements. Arguably, our approach provides a useful platform for directly comparing experimental results with theoretical data.

## Experimental Section

General experimental details and instrumentation used are included in the Supporting Information document. Included below are generalized experimental procedures for the cyclometallation of the coumarin ligands **6‐(*X*‐R)**.

Cyclometalation of coumarin ligands **6‐(*X*‐R)**: To a flame‐dried Schlenk tube under N_2_, equipped with a magnetic stirrer bar, was added coumarin **4‐(*X*‐R)** (1.0 equiv.) and benzyl manganese pentacarbonyl (1.0 equiv.), followed by dry toluene (50 mL mmol^−1^). The solution was heated to 95 °C with stirring, which was left to continue stirring for a further 2.5 h. Upon completion, the reaction was cooled to room temperature and the mixture concentrated *in vacuo* to yield the cyclometalated coumarins.

Thermally‐induced alkyne insertion and reductive elimination **5‐(*X*‐R)**: To a flame‐dried Schlenk tube under N_2_, equipped with a magnetic stirrer bar, was added cyclometalated coumarin **6‐(*X*‐R)** (1.0 equiv.) in dry Bu_2_O or Et_2_O (60 mL mmol^−1^). To the solution, alkyne **a**‐**h** (1.5 equiv.) and (trimethylamine *N*‐oxide (1.0 equiv.) if required) were added. The solution was heated to 80 °C, with the aid of a water‐filled cold‐finger and solution left stirring for a further 18 h. Upon cooling to room temperature, the reaction mixture was diluted with dichloromethane (15 mL) and the solution concentrated *in vacuo* (by rotavaporator) to give crude product. Crude product was dissolved in minimal dichloromethane and precipitated out with excess hexane (ca. 10‐fold. excess to dichloromethane). The precipitate was filtered and dried *in vacuo* (on a standard Schlenk line) to yield the reductive elimination products.

Deposition Number(s) https://www.ccdc.cam.ac.uk/services/structures?id=doi:10.1002/chem.202203038 2204313 (for 5a‐(7‐NEt_2_)), 2204314 (for 5b‐(7‐NEt_2_)), 2204315 (for 8a), 2204316 (for 5c‐(7‐NEt_2_)), 2204317 (for 5d‐(7‐NEt_2_)), 2204324 (for 5 h‐(7‐NEt_2_)), 2204319 (for 6(H)), 2204318 (for 5a‐(H)), 2204320 (for 6‐(7‐OMOM)), 2204321 (for 5a‐(7‐Me)), 2204322 (for 5a‐(7‐NO_2_)), 2204323 (for 10a), 2204325 (for benzyl rhenium(I) pentacarbonyl), 2204326 (for 6’(H)) contain(s) the supplementary crystallographic data for this paper. These data are provided free of charge by the joint Cambridge Crystallographic Data Centre and Fachinformationszentrum Karlsruhe http://www.ccdc.cam.ac.uk/structures Access Structures service.

## Conflict of interest

The authors declare no conflict of interest.

1

## Supporting information

As a service to our authors and readers, this journal provides supporting information supplied by the authors. Such materials are peer reviewed and may be re‐organized for online delivery, but are not copy‐edited or typeset. Technical support issues arising from supporting information (other than missing files) should be addressed to the authors.

Supporting Information

## Data Availability

The data that support the findings of this study are available in the supplementary material of this article.
