# Supplementary material for: Coumarin C−H Functionalization by Mn(I) Carbonyls: Mechanistic Insight by Ultra‐Fast IR Spectroscopic Analysis
Source: Chemistry. 2023 Mar 23;29(25):e202203038. doi: 10.1002/chem.202203038 (PMC10947090; doi:10.1002/chem.202203038)
Supplement: Supplementary file 1 — Supporting Information [file CHEM-29-0-s001.pdf]

Chemistry–A European Journal

Supporting Information

Coumarin C–H Functionalization by Mn(I) Carbonyls: Mechanistic Insight by Ultra-Fast IR Spectroscopic Analysis

Thomas J. Burden, Kathryn P. R. Fernandez, Mary Kagoro, Jonathan B. Eastwood, Theo F. N. Tanner, Adrian C. Whitwood, Ian P. Clark, Michael Towrie, Jean-Philippe Krieger, Jason M. Lynam,* and Ian J. S. Fairlamb*

Supporting Information

Contents

1.0 Experimental Details	S3
1.1 General Information	S3
1.2 Experimental Procedures and Characterization Data	S5
2.0 Infrared Spectroscopic Measurements	S36
2.1 Time-Resolved Infrared Spectroscopy (TRIR) Experiments	S36
2.2 ReactIR (*in situ* IR) Experiments on the Second Timescale	S40
2.3 Light-Induced Reaction Time Course	S42
3.0 Computational Calculations Using Density Functional Theory (DFT) Methods	S43
3.1 DFT xyz Coordinates and Collated Energies	S44
3.2 Time-Dependent Density Functional Theory (TD-DFT) Calculations	S64
4.0 Crystallographic Data	S66
5.0 Representative ^{1}H and ^{13}C NMR Spectra	S94
6.0 References	S145

Important Safety Note

General safety precautions when working with diethyl ether at 80 ºC were performed, no incidents occurred handling the solvent at this temperature, but readers should be aware of potential danger and exercise caution.

1 Experimental Details

1.1 General Information

Commercially sourced solvents and reagents were purchased from Acros Organics, Alfa Aesar, Fisher Scientific, Fluorochem, Sigma-Aldrich or VWR and used as received unless otherwise noted. Petrol refers to the fraction of petroleum ether boiling in the range of 40–60 °C. Room temperature (RT) refers to reactions where no thermostatic control was applied and was recorded as 16–23 °C.

Thin layer chromatography (TLC) analysis was performed using Merck 5554 aluminum backed silica plates. Spots were visualized by the quenching of ultraviolet light (λ_{max} = 254 nm) or through staining with $KMnO_4$. Retention factors (R_f) are quoted to two decimal places and reported along with the solvent system used in parentheses. All flash column chromatography was performed using either Merck 60 or Fluorochem 60 Å silica gel (particle size 40–63 µm) and the solvent system used is reported in parentheses.

Melting points were recorded using a Stuart digital SMP3 machine using a temperature ramp of 5 °C min^{-1} and are quoted to the nearest whole number. Where applicable, decomposition (dec.) is noted.

All NMR spectra were recorded on either Jeol ECS400, Jeol ECX400, Bruker AVIIIHD 500, or Bruker AVIIIHD 600 (typically at 298 K). Chemical shifts are reported in parts per million (ppm) of tetramethyl silane. Coupling constants (*J*) are reported in Hz and quoted to ±0.5 Hz. Multiplicities are described as singlet (s), doublet (d), triplet (t), quartet (q), quintet (quin), sextet, (sext), heptet (hept), multiplet (m), apparent (app) and broad (br). Spectra were processed using MestReNova version 12. NMR spectra are representative of the compounds prepared.

Proton (^{1}H) spectra were typically recorded at 600, 500, and 400 MHz. Chemical shifts are internally referenced to residual non-deuterated solvent (CD_2Cl_2 $\delta H = 5.32$ ppm), ($CDCl_3$ $\delta H = 7.26$ ppm), (CD_3CN $\delta H = 1.94$ ppm), and (DMSO-D_6 $\delta H = 2.50$ ppm), given to two decimal places.

Carbon-13 (^{13}C) spectra were recorded at 125 and 101 MHz. Chemical shifts are internally referenced to residual solvent (CD_2Cl_2 $\delta C = 58.0$ ppm), ($CDCl_3$ $\delta C = 77.0$ ppm), (CD_3CN $\delta C = 118.3$ ppm), and (DMSO-D_6 $\delta C = 39.5$ ppm) and given to one decimal place.

Fluorine-19 (^{19}F) spectra were recorded at 376 MHz and obtained with ^{1}H decoupling. Chemical shifts are externally referenced to $CFCl_3$ and given to one decimal place.

Electrospray ionisation (ESI) mass spectrometry was performed using a Bruker Daltronics micrOTOF spectrometer. Electron impact (EI) mass spectrometry was performed using a Waters GCT Premier mass spectrometer. Mass to charge ratios (m/z) are reported in Daltons with percentage abundance in parentheses along with the corresponding fragment ion, where known. Where complex isotope patterns were observed, the most abundant ion is reported. High resolution mass spectra (HRMS) are reported with less than 5 ppm error.

Infrared spectra were recorded on a Perkin Elmer UATR Two FT-IR spectrometer. Absorption maxima (ν_{max}) are reported in wavenumbers (cm^{-1}) to the nearest whole number.

Time-resolved infra-red spectra were recorded on the LIFEtime instrument in the ULTRA facility at the STFC's Rutherford Appleton Laboratories.[1] The pump source was the output of a Yb:KGW amplifier providing 15W, 260 fs pulses at 1030 nm with a 100 kHz repetition rate (Pharos) that is divided down to 1 kHz. This was used to drive a BBO-based 515 nm pumped optical parametric amplifier (OPA) to deliver pulses at 355 nm. The pump beam was collimated, travelled over a computer programmable 0 - 16 ns optical delay (1200 mm long, double pass), and focused onto the sample. The pump energy at the sample was attenuated down to 500 nJ and focused down to a 120 μm diameter spot. The probe source was the output of a Yb:KGW amplifier providing 6 W, 180 fs pulses at 1030 nm with a 100 kHz repetition rate (Pharos). This was split 50/50 to pump two 3 W BBO/KTA based OPAs. The two Pharos sources (pump and probe) share a common 80 MHz oscillator to allow for pump-probe delay steps of 12.5 ns. The probe beam was split to provide probe and reference pulses. To go beyond pump-probe delays of 12.5 ns, subsequent seed pulses were selected from the 80 MHz oscillator. Data were collected using pump-probe delays

ranging from 1 ps to 988.8 μs. The probe beams were collimated, synchronised by a fixed optical delay, and focused by a single gold parabolic mirror onto the sample. The three beams were overlapped on the sample with the aid of a 50 μm pinhole in the sample plain. The probe beams were measured by two separate spectrometers and 128-element mercury cadmium telluride (MCT) detectors. To cover the full spectroscopic window required, data from different detector positions were combined to generate the required spectra. Data were initially visualized in the ULTRA View version 2 software,[2] where baseline correction was undertaken. The resulting spectra were then exported as comma-separated variable files into Origin2019.[3] The spectra were calibrated against samples of polystyrene (200 μm thick), [Mn(ppy)$(CO)_4$] in heptane and 1,4-dioxane. Kinetic analysis was performed by fitting to the expgro, expdec, or expgrodec functions in Origin2019. All quoted errors are 95% confidence limits.

ReactIR infrared spectra were recorded on a ReactIR silicon-tipped ATR-IR probe. Reactions were typically performed in dry toluene (6 mL) in a 100 mL two necked round-bottomed flask equipped with a stir bar. A background spectrum was collected of the reaction solvent toluene once at 60 ºC to be used as a reference. The sample measurements were thereafter started, coumarin **4-(7-NEt$_2$)** (0.136 mmol, 0.040 g, 1.0 eq.), was added. After the corresponding IR peaks had stabilized, phenylacetylene **2a**, (0.204 mmol, 22 μL, 1.5 eq.) was added, followed by benzyl manganese pentacarbonyl (0.136 mmol, 0.038 g, 1.0 eq.). IR spectra were recorded every 0.5 min. The data were exported to Origin2019 to be plotted as a function of time.

1.2 Experimental Procedures and Characterisation

Benzyl manganese pentacarbonyl and *p*-tolylacetylide pentacarbonyl Mn(I) **7h** were prepared as reported by our group.[4]

7-(Diethylamino)-3-(pyridin-2-yl)-*2H*-chromen-2-one 4-(*7*-NEt$_2$)

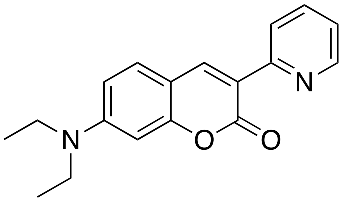

To a round bottomed flask, equipped with a magnetic stirrer bar, was added 4-(*N,N*-diethylamino)salicylaldehyde (10.0 mmol, 1.93 g, 1.0 eq.) and pyridine-2-acetonitrile (10.0 mmol, 1.18 g, 1.0 eq.) in absolute ethanol (20 mL). The solution was cooled to 0 °C and then piperidine (0.6 mL) was added dropwise with stirring, resulting solution was left to continue stirring for a further 12 h at 0 ºC. Upon completion, to the reaction was added HCl (3.5%, 50 mL) with refluxing for 8 h to hydrolyse iminocoumarin. The solution was then neutralised with aqueous ammonia until the pH was 7. The solvent was then removed *in vacuo*, and the dried product was extracted with dichloromethane (2 x 50 mL). Organic phase was washed with water (2 x 50 mL), the combined organic extracts were dried ($MgSO_4$), filtered and concentrated *in vacuo*. The crude product was suspended in minimal dichloromethane (4 mL) and purified by automated silica gel column chromatography (hexane:EtOAc, 8:1, v/v) to afford the product **4-(*7*-NEt$_2$)** as an orange solid (1.33 g, 45%). MP 113.0-114.2 ºC. ^{1}H NMR (400 MHz, Chloroform-*d*) δ 8.71 (s, 1H), 8.64 (ddd, *J* = 4.8, 1.9, 0.9 Hz, 1H), 8.44 (d, *J* = 8.1 Hz, 1H), 7.84 – 7.69 (m, 1H), 7.43 (d, *J* = 8.1 Hz, 1H), 7.22 (ddd, *J* = 7.5, 4.8, 1.1 Hz, 1H), 6.62 (dd, *J* = 8.9, 2.5 Hz, 1H), 6.53 (d, *J* = 2.5 Hz, 1H), 3.44 (q, *J* = 7.1 Hz, 4H), 1.23 (t, *J* = 7.1 Hz, 6H). ^{13}C NMR (101 MHz, chloroform-*d*) δ 161.6, 156.9, 152.7, 151.3, 149.1, 143.1, 136.6, 130.2, 123.4, 122.2, 117.7, 109.3, 109.2, 96.8, 45.0, 12.6. IR (solid-state ATR, cm^{-1}) 2980, 2929, 1713, 1596, 1514, 1471, 1412, 1347, 1272. MS; HRMS (ESI+) m/z: [MH]+ calcd for $C_{18}H_{19}N_2O_2$ 295.1441; Found 295.1441.

(7-(Diethylamino)-3-(pyridin-2-yl)coumarin)-tetracarbonyl manganese 6-(*7*-NEt$_2$)

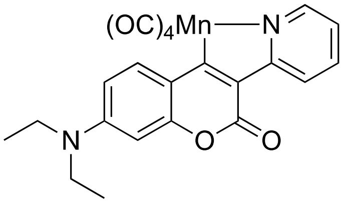

To a flame-dried Schlenk tube under N_2, equipped with a magnetic stirrer bar, was added 7-(diethylamino)-3-(pyridin-2-yl)coumarin (0.68 mmol, 0.200 g, 1.0 eq.) and benzylmanganese pentacarbonyl (0.68 mmol, 0.194 g, 1.0 eq.), followed by dry toluene (10.8 mL). The solution was heated to 95 °C with stirring, which was left to continue stirring for a further 2.5 h. Upon completion, the reaction was cooled to room temperature and the mixture concentrated *in vacuo*. The crude product was dissolved in dichloromethane

(10 mL) and solution was filtered through a plug of Celite™. The filtrate was concentrated *in vacuo* to afford the product **6-(7-NEt$_2$)** as a yellow solid. (0.310 g, 99%). MP 90-91 °C. ^{1}H NMR (400 MHz, Methylene Chloride-d_2) δ 8.93 (d, *J* = 8.4 Hz, 1H), 8.76 (d, *J* = 5.7 Hz, 1H), 8.08 (d, *J* = 9.1 Hz, 1H), 7.81 (ddd, *J* = 8.6, 7.3, 1.6 Hz, 1H), 7.09 (ddd, *J* = 7.3, 5.7, 1.5 Hz, 1H), 6.73 (dd, *J* = 9.2, 2.7 Hz, 1H), 6.49 (d, *J* = 2.7 Hz, 1H), 3.48 (q, *J* = 7.1 Hz, 4H), 1.24 (t, *J* = 7.1 Hz, 6H). ^{13}C NMR (101 MHz, Chloroform-*d*) δ 219.4, 218.5, 214.6, 211.7, 166.0, 155.5, 152.9, 151.4, 138.1, 135.0, 125.8, 124.1, 121.2, 120.5, 109.2, 96.6, 45.0, 12.8. IR (solid-state ATR, cm^{-1}) 2971, 2078, 1968, 1921, 1680, 1607, 1591, 1454, 1403, 1350, 1277, 1250, 1130, 1075, 1016, 950, 826, 789, 748, 630, 552, 445. Solution phase IR (in MeCN cm^{-1}) 2082, 2002, 1982, 1934, 1682, 1132. MS; HRMS (ESI+) m/z: [MH]+ calcd for $C_{22}H_{18}MnN_2O_6$ 461.0543; Found 461.0540.

8-(Diethylamino)-5-oxo-12-phenyl-5*H*-chromeno[3,4-*a*]quinolizin-13-ium manganese tricarbonyl 5a-(7-NEt$_2$)

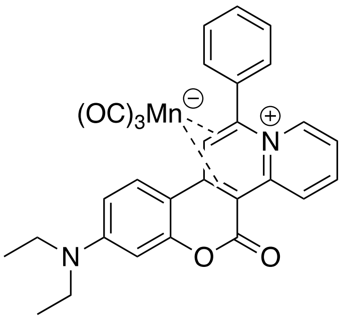

To a flame-dried Schlenk tube under N_2, equipped with a magnetic stirrer bar, was added (7-(diethylamino)-3-(pyridin-2-yl)coumarin)-tetracarbonyl manganese (0.119 mmol, 0.055 g, 1.0 eq.) in dry ^{n}Bu$_2$O (7 mL). To the solution, phenylacetylene (0.143 mmol, 0.016 mL, 1.2 eq.) and the solution was heated to 80 ºC, it was then left to continue stirring for a further 18 h. Upon cooling to room temperature, the reaction mixture was diluted with EtOAc (15 mL) and the solution concentrated *in vacuo*. The precipitate was then collected by filtration. The product was not further purified, giving **5a-(7-NEt$_2$)** as a bronze powder (0.048 g, 71%). MP decomp. 287 °C. ^{1}H NMR (600 MHz, Methylene Chloride-d_2) δ 7.95 (d, *J* = 8.7 Hz, 1H), 7.82 (ddd, *J* = 8.3, 1.5, 0.7 Hz, 1H), 7.71 – 7.66 (m, 1H), 7.59 (dt, *J* = 8.1, 1.5 Hz, 1H), 7.55 – 7.47 (m, 3H), 7.44 – 7.34 (m, 2H), 6.76 (ddd, *J* = 7.4, 6.4, 1.5 Hz, 1H), 6.59 (dd, *J* = 8.8, 2.6 Hz, 1H), 6.51 (s, 1H), 6.45 (d, *J* = 2.6 Hz, 1H), 3.41 (dq, *J* = 7.5, 4.4 Hz, 4H), 1.20 (t, *J* = 7.1 Hz, 6H). ^{13}C NMR (151 MHz, Methylene Chloride-d_2) δ 231.9, 223.3, 218.6, 165.7, 153.6, 150.3, 139.1, 137.7, 137.4, 134.2, 130.3, 130.0, 129.1, 128.7, 124.2, 122.6, 120.6, 108.4, 108.3, 102.8, 99.1, 90.2, 72.9, 45.1, 12.9. IR (solid-state ATR, cm^{-1}) 3083, 2971, 2930, 1980, 1909, 1879, 1709, 1607, 1503, 1353, 1192, 799. MS; HRMS (ESI+) m/z: [MH]+ calcd for $C_{29}H_{24}MnN_2O_5$ 535.1060; Found 535.1068.

8-(Diethylamino)-5-oxo-12-phenyl-5*H*-chromeno[3,4-*a*]quinolizin-13-ium manganese tricarbonyl [D$_1$]-5a-(*7*-NEt$_2$)

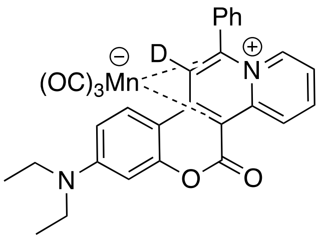

[D$_1$]**-5a-(*7*-NEt$_2$)** was synthesised on an identical scale to **5a-(*7*-NEt$_2$)**. [D$_1$]**-5a-(*7*-NEt$_2$)** was not further purified, appeared as a bronze powder (0.063 g, 100%). ^{1}H NMR (600 MHz, Methylene Chloride-*d*$_2$) δ 7.95 (d, *J* = 8.7 Hz, 1H), 7.82 (ddd, *J* = 8.3, 1.5, 0.7 Hz, 1H), 7.71 – 7.66 (m, 1H), 7.59 (dt, *J* = 8.2, 1.5 Hz, 1H), 7.55 – 7.47 (m, 3H), 7.44 – 7.34 (m, 2H), 6.76 (ddd, *J* = 7.4, 6.1, 1.5 Hz, 1H), 6.59 (dd, *J* = 8.8, 2.6 Hz, 1H), 6.51 (s, 0.02H), 6.45 (d, *J* = 2.6 Hz, 1H), 3.41 (dq, *J* = 7.1, 4.4 Hz, 4H), 1.20 (t, *J* = 7.1 Hz, 6H). HRMS (ESI+) m/z: [MH]+ calcd for $C_{29}H_{23}DMnN_2O_5$ 536.1123; Found 536.1122.

8-(Diethylamino)-5-oxo-11,12-diphenyl-5*H*-chromeno[3,4-*a*]quinolizin-13-ium manganese tricarbonyl 5b-(*7*-NEt$_2$)

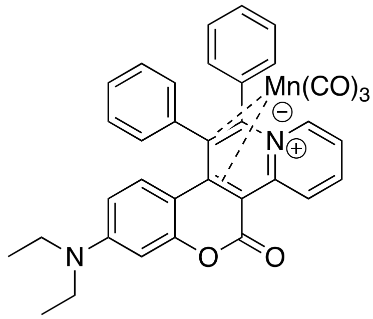

To a flame-dried Schlenk tube under N$_2$, equipped with a magnetic stirrer bar, was added (7-(diethylamino)-3-(pyridin-2-yl)coumarin)-tetracarbonyl manganese (0.109 mmol, 0.05 g, 1.0 eq.) in dry ^{n}Bu$_2$O (7 mL). To the solution, diphenylacetylene (0.123 mmol, 0.022 mL, 1.1 eq.) solution was heated to 80 °C left to continue stirring for a further 18 h. Upon completion, the reaction mixture was diluted with dichloromethane (15 mL) and the solution concentrated *in vacuo*. The crude material was then suspended in minimal volume of dichloromethane and hexane was added to precipitate product. Desired product was collected by gravity filtration, solid was dried *in vacuo*. Product **5b-(*7*-NEt$_2$)** was a red powder (0.040 g, 60%). MP decomp. 246 °C. ^{1}H NMR (600 MHz, Methylene Chloride-*d*$_2$) δ 7.96 (dq, *J* = 7.7, 1.2 Hz, 1H), 7.88 (ddd, *J* = 8.3, 1.5, 0.7 Hz, 1H), 7.79 (dt, *J* = 7.9, 1.7 Hz, 1H), 7.63 – 7.56 (m, 2H), 7.55 – 7.50 (m, 1H), 7.45 – 7.37 (m, 4H), 7.21 (tt, *J* = 7.4, 1.2 Hz, 1H), 7.03 (td, *J* = 7.7, 1.5 Hz, 1H), 6.90 (dt, *J* = 8.1, 1.6 Hz, 1H), 6.81 (ddd, *J* = 7.3, 6.5, 1.4 Hz, 1H), 6.41 – 6.36 (m, 2H), 6.07 (dd, *J* = 9.2, 2.7 Hz, 1H), 3.30 (q, *J* = 7.1 Hz, 4H), 1.11 (t, *J* = 7.1 Hz, 6H). ^{13}C NMR (151 MHz, Methylene Chloride-*d*$_2$) δ 231.4, 223.8, 218.1,

166.0, 157.3, 154.0, 149.1, 138.5, 137.6, 137.5, 137.2, 136.3, 133.6, 133.1, 131.8, 130.24, 130.1, 128.8, 128.5, 128.1, 126.6, 123.2, 120.5, 108.7, 107.3, 101.5, 99.1, 97.6, 93.3, 44.7, 12.7. IR (solid-state ATR, cm^{-1}) 2972, 2929, 2079, 1975, 1929, 1896, 1702, 1683, 1592, 1457, 1406, 1352, 1159, 1131, 1073, 688, 652, 631, 552. MS; HRMS (ESI+) m/z: [MH]+ calcd for $C_{35}H_{27}MnN_2O_5$ 611.1373; Found 611.1370.

8-(Diethylamino)-11-methyl-5-oxo-12-phenyl-5*H*-chromeno[3,4-*a*]quinolizin-13-ium manganese tricarbonyl 5c-(*7*-NEt₂)

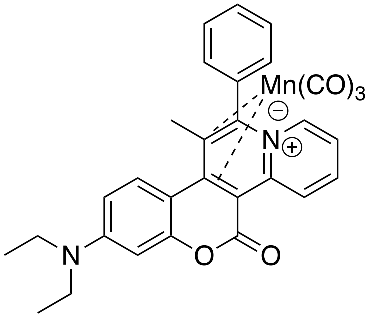

To a flame-dried Schlenk tube under N_2, equipped with a magnetic stirrer bar, was added (7-(diethylamino)-3-(pyridin-2-yl)coumarin)-tetracarbonyl manganese (0.04 mmol, 0.0185 g, 1.0 eq.) in dry Et_2O (6 mL). To the solution, 1-phenyl-1-propyne (0.04mmol, 0.005 mL, 1.0 eq.) solution was heated to 80 ºC left to continue stirring for a further 18 h. Upon cooling to room temperature, the reaction mixture was diluted with dichloromethane (5 mL) and the solution concentrated *in vacuo*. Product **5c-(*7*-NEt_2)** was not further purified, giving product as a red oil (0.019 g, 86%). ^{1}H NMR (400 MHz, Methylene Chloride-d_2) δ 8.01 (d, *J* = 8.9 Hz, 1H), 7.92 – 7.80 (m, 1H), 7.70 (ddd, *J* = 8.2, 1.5, 0.7 Hz, 1H), 7.64 – 7.58 (m, 1H), 7.58 – 7.53 (m, 2H), 7.50 – 7.46 (m, 1H), 7.30 – 7.26 (m, 1H), 7.19 (ddd, *J* = 6.5, 1.4, 0.7 Hz, 1H), 6.68 (ddd, *J* = 7.3, 6.5, 1.5 Hz, 1H), 6.59 (dd, *J* = 8.9, 2.7 Hz, 1H), 6.52 (d, *J* = 2.7 Hz, 1H), 3.42 (qd, *J* = 7.2, 4.7 Hz, 4H), 2.68 (s, 3H), 1.21 (t, *J* = 7.1 Hz, 6H). ^{13}C NMR (101 MHz, Methylene Chloride-d_2) δ 166.3, 154.9, 149.3, 138.6, 138.1, 134.3, 131.9, 131.3, 129.9, 129.0, 128.8, 127.1, 122.4, 120.1, 109.3, 107.4, 99.7, 97.7, 95.9, 91.0, 45.0, 23.5, 12.9. IR (solid-state ATR, cm^{-1}) 3056, 2972, 2929, 1983, 1883, 1706, 1617, 1496, 1356, 1252, 1101, 734, 669. MS; HRMS (ESI+) m/z: [M–H]+ calcd for $C_{30}H_{26}MnN_2O_5$ 549.1217; Found 549.1123.

12-Cyclopropyl-8-(diethylamino)-5-oxo-5*H*-chromeno[3,4-*a*]quinolizin-13-ium manganese tricarbonyl 5d-(*7*-NEt$_2$)

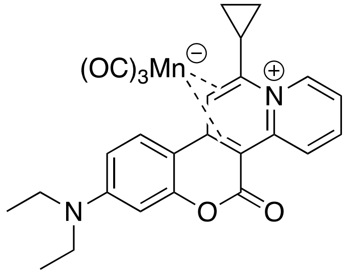

To a flame-dried Schlenk tube under N_2, equipped with a magnetic stirrer bar, was added (7-(diethylamino)-3-(pyridin-2-yl)coumarin)-tetracarbonyl manganese (0.069 mmol, 0.032 g, 1.0 eq.) in dry Et_2O (5 mL). To the solution, ethynylcyclopropane (0.069 mmol, 0.006 mL, 1.0 eq.) was added, solution was heated to 80 ºC left to continue stirring for a further 18 h. Upon cooling to room temperature, the reaction mixture was diluted with dichloromethane (5 mL) and the solution concentrated *in vacuo*. Product **5d-(*7*-NEt$_2$)** was not further purified, giving product as a red oil (0.029 g, 83%). ^{1}H NMR (500 MHz, Methylene Chloride-d_2) δ 8.60 (d, *J* = 6.4 Hz, 1H), 7.83 (d, *J* = 8.7 Hz, 1H), 7.73 (d, *J* = 8.3 Hz, 1H), 7.54 (app.t, *J* = 7.8 Hz, 1H), 6.98 (app.t, *J* = 6.9 Hz, 1H), 6.55 (dd, *J* = 8.7, 2.6 Hz, 1H), 6.41 (d, *J* = 2.5 Hz, 1H), 5.74 (s, 1H), 3.44 – 3.35 (m, 4H), 1.19 (t, *J* = 7.1 Hz, 6H), 1.09 – 0.99 (m, 2H), 0.91 – 0.84 (m, 2H), 0.59 – 0.52 (m, 1H). ^{13}C NMR (101 MHz, Methylene Chloride-d_2) δ 218.2, 212.5, 166.4, 155.5, 153.4, 151.8, 150.0, 138.5, 135.4, 126.2, 124.1, 121.6, 120.6, 109.6, 96.6, 96.6, 87.1, 71.1, 45.4, 15.6, 12.9, 10.5, 6.4. IR (solid-state ATR, cm^{-1}) 2974, 2931, 2079, 1972, 1926, 1662, 1592, 1456, 1405, 1352, 1252, 1131, 1017, 750, 631. MS; HRMS (ESI+) m/z: [M–Na]+ calcd for $C_{26}H_{23}MnN_2NaO_5$ 521.0880; Found 521.0897.

8-(Diethylamino)-11-(methoxycarbonyl)-5-oxo-12-(4-(trifluoromethyl)phenyl)-5*H*-chromeno[3,4-*a*]quinolizin-13-ium manganese tricarbonyl 5e-(*7*-NEt$_2$)

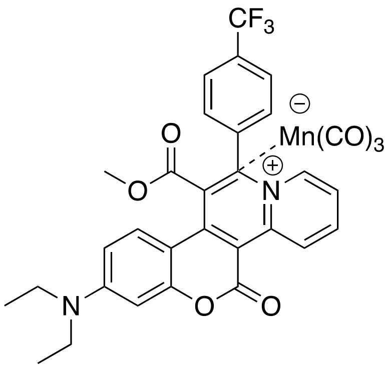

To a flame-dried Schlenk tube under N_2, equipped with a magnetic stirrer bar, was added (7-(diethylamino)-3-(pyridin-2-yl)coumarin)-tetracarbonyl manganese (0.13 mmol, 0.06 g, 1.0 eq.) in dry Et_2O (6 mL). To the solution, methyl 3-(4-(trifluoromethyl)phenyl)propiolate (0.13 mmol, 0.03 g, 1.0 eq.) was added,

resulting solution was heated to 80 ºC left to continue stirring for a further 18 h. Upon cooling to room temperature, the reaction mixture was diluted with dichloromethane (5 mL) and the solution concentrated *in vacuo*. Product **5e-(7-NEt$_2$)** was not further purified, giving product as a red oil (0.070 g, 82%). ^{1}H NMR (400 MHz, Methylene Chloride-d_2) δ 10.87 (d, *J* = 8.3 Hz, 1H), 10.70 (dd, *J* = 5.7, 1.5 Hz, 1H), 10.02 (d, *J* = 9.1 Hz, 1H), 9.75 (ddd, *J* = 8.5, 7.3, 1.6 Hz, 1H), 9.69 – 9.64 (m, 2H), 9.63 – 9.58 (m, 2H), 9.03 (ddd, *J* = 7.1, 5.7, 1.1 Hz, 1H), 8.67 (dd, *J* = 9.1, 2.6 Hz, 1H), 8.44 (d, *J* = 2.7 Hz, 1H), 5.77 (s, 3H), 5.42 (q, *J* = 7.2 Hz, 4H), 3.18 (t, *J* = 7.1 Hz, 6H). ^{19}F NMR (376 MHz, Methylene Chloride-d_2) δ -63.42.^{13}C NMR (75 MHz, Methylene Chloride-d_2) δ 171.2, 166.5, 154.4, 153.5, 153.4, 151.9, 138.6, 135.4, 133.8, 130.0, 126.2, 126.1, 124.2, 124.0, 122.5, 121.6, 120.7, 114.4, 109.6, 96.7, 84.4, 82.4, 53.5, 45.4, 13.0. IR (thin-film ATR, cm^{-1}) 2973, 2231, 2080, 1976, 1928, 1703, 1684, 1592, 1457, 1406, 1321, 1128, 1065, 735, 631. MS; HRMS (ESI+) m/z: [M–Na]+ calcd for $C_{32}H_{24}F_3MnN_2NaO_7$ 683.0808; Found 683.0833.

8-(Diethylamino)-5-oxo-12-(ferrocenyl-)-5*H*-chromeno[3,4-*a*]quinolizin-13-ium manganese tricarbonyl 5f-(7-NEt$_2$)

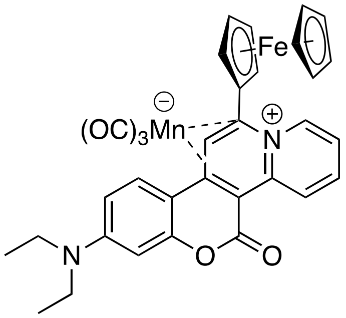

To a flame-dried Schlenk tube under N$_2$, equipped with a magnetic stirrer bar, was added (7-(diethylamino)-3-(pyridin-2-yl)coumarin)-tetracarbonyl manganese (0.06 mmol, 0.028 g, 1.0 eq.) in dry Et$_2$O (5 mL). To the solution, ethynylferrocene (0.06 mmol, 0.013 g, 1.0 eq.) solution was heated to 80 ºC left to continue stirring for a further 18 h. Upon cooling to room temperature, the reaction mixture was diluted with dichloromethane (10 mL) and the solution concentrated *in vacuo*. Product **5f-(7-NEt$_2$)** was not further purified, giving product as a red oil (0.037 g, 95%). ^{1}H NMR (500 MHz, Methylene Chloride-d_2) δ 8.00 (d, *J* = 8.7 Hz, 1H), 7.77 (dd, *J* = 8.3, 1.5 Hz, 1H), 7.52 (ddd, *J* = 8.6, 7.4, 1.4 Hz, 1H), 6.90 (t, *J* = 7.0 Hz, 1H), 6.62 (dd, *J* = 8.8, 2.6 Hz, 1H), 6.49 (s, 1H), 6.44 (d, *J* = 2.6 Hz, 1H), 4.47 – 4.41 (m, 2H), 4.37 – 4.34 (m, 1H), 4.31 (s, 5H), 4.09 – 4.04 (m, 1H), 3.42 (hept, *J* = 7.6 Hz, 4H), 1.21 (t, *J* = 7.1 Hz, 6H), 0.89 (s, 1H). ^{13}C NMR (126 MHz, Methylene Chloride-d_2) δ 218.2, 215.0, 212.5, 166.5, 155.6, 153.4, 151.9, 150.2, 138.6, 135.4, 126.2, 124.2, 121.6, 120.7, 109.6, 99.2, 96.7, 83.0, 73.9, 72.3, 70.6, 70.2, 69.4, 45.4, 12.9. IR (thin-film ATR, cm^{-1}) 3297, 3094, 2973, 2930, 2079, 1973, 1927, 1682, 1607, 1591, 1456, 1405, 1377, 1252, 1211, 1161, 1018, 791, 648, 631. MS; HRMS (ESI+) m/z: [M–H]+ calcd for $C_{33}H_{28}FeMnN_2O_5$ 643.0723; Found 643.0710.

8-(Diethylamino)-5-oxo-12-(2-pyridyl)-5*H*-chromeno[3,4-*a*]quinolizin-13-ium manganese tricarbonyl 5g-(*7*-NEt$_2$)

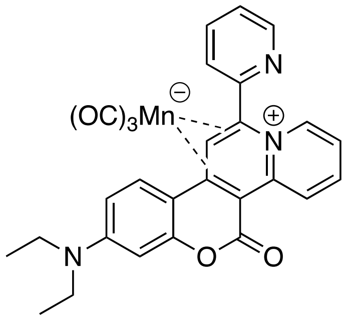

To a flame-dried Schlenk tube under N_2, equipped with a magnetic stirrer bar, was added (7-(diethylamino)-3-(pyridin-2-yl)coumarin)-tetracarbonyl manganese (0.109 mmol, 0.05 g, 1.0 eq.) in dry Et_2O (6 mL). To the solution, 2-ethynylpyridine (0.109 mmol, 0.011 mL, 1.0 eq.) was added to the solution and was heated to 80 ºC left to continue stirring for a further 18 h. Upon cooling to room temperature, the reaction mixture was diluted with dichloromethane (5 mL) and the solution concentrated *in vacuo*. Product **5b-(*7*-NEt$_2$)** was not further purified, giving product as a brown oil (0.064 g, 93%). ^{1}H NMR (400 MHz, Methylene Chloride-*d*$_2$) δ 8.90 (d, *J* = 8.4 Hz, 1H), 8.73 (d, *J* = 5.8 Hz, 1H), 8.66 – 8.36 (m, 2H), 8.05 (dd, *J* = 9.0, 1.7 Hz, 1H), 7.79 (t, *J* = 7.9 Hz, 1H), 7.69 – 7.57 (m, 1H), 7.54 – 7.43 (m, 1H), 7.30 – 7.19 (m, 1H), 7.12 – 7.02 (m, 1H), 6.70 (dd, *J* = 9.1, 2.2 Hz, 1H), 6.47 (s, 1H), 3.45 (qd, *J* = 7.1, 1.7 Hz, 4H), 1.22 (t, *J* = 7.2 Hz, 6H). ^{13}C NMR (101 MHz, Methylene-Chloride-*d*$_2$) δ 220.1, 218.2, 212.5, 166.4, 155.5, 153.4, 151.8, 150.6, 142.8, 138.5, 136.7, 135.4, 129.8, 128.1, 126.2, 124.1, 122.5, 121.60, 120.6, 112.4, 109.6, 96.6, 83.4, 77.1, 45.4, 12.9. IR (thin-film ATR, cm^{-1}) 3297, 3054, 2974, 2931, 2079, 1973, 1925, 1682, 1607, 1591, 1456, 1405, 1351, 1131, 750, 629, 444. MS; HRMS (ESI+) m/z: [M-H]+ calcd for $C_{28}H_{23}MnN_3O_5$ 536.1013; Found 536.1019.

8-(Diethylamino)-5-oxo-12-(*p*-tolyl)-5H-chromeno[3,4-a]quinolizin-13-ium manganese tricarbonyl 5h-(*7*-NEt$_2$)

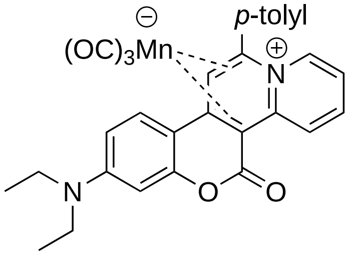

To a flame-dried Schlenk tube under N_2, equipped with a magnetic stirrer bar, was added (7-(diethylamino)-3-(pyridin-2-yl)coumarin)-tetracarbonyl manganese (0.109 mmol, 0.050 g, 1.0 eq.) in dry toluene (6 mL). To the solution, 4-ethynyltoluene (0.164 mmol, 0.019 g, 1.5 eq.) solution was heated to 80 ºC, left to

continue stirring for a further 18 h. Upon cooling to room temperature, the reaction mixture was diluted with EtOAc (15 mL) and the solution concentrated in vacuo. The crude material was dissolved in dichloromethane (5 mL) and desired product precipitated with hexane (50 mL). Product was dried *in vacuo* to give **5h-(*7*-NEt$_2$)** as a red powder (0.049 g, 82%). ^{1}H NMR (400 MHz, Methylene Chloride-d_2) δ 7.94 (d, J = 8.7 Hz, 1H), 7.80 (d, J = 8.2 Hz, 1H), 7.59 – 7.52 (m, 1H), 7.52 – 7.44 (m, 2H), 7.37 (d, J = 6.3 Hz, 1H), 7.32 (t, J = 7.1 Hz, 2H), 6.74 (t, J = 6.3 Hz, 1H), 6.58 (dd, J = 8.8, 2.5 Hz, 1H), 6.47 (s, 1H), 6.44 (d, J = 2.6 Hz, 1H), 3.47 – 3.35 (m, 3H), 2.41 (s, 3H), 1.20 (t, J = 7.1 Hz, 6H). 13C NMR (151 MHz, CD2Cl2) δ 231.9, 223.5, 219.6, 165.7, 156.9, 153.6, 150.3, 139.4, 139.0, 137.4, 134.6, 134.2, 131.2, 130.5, 128.9, 124.2, 122.5, 120.5, 108.3, 102.7, 99.2, 90.2, 73.2, 45.1, 21.5, 12.9. IR (solid-state ATR, cm^{-1}) 2972, 1980, 1882, 1708, 1616, 1563, 1542, 1496, 1465, 1262, 1196, 1120, 1026, 957, 798, 666. MS; HRMS (ESI+) m/z: [MH]+ calcd for $C_{30}H_{26}MnN_2O_5$ 549.1217; Found 549.1221.

7-Hydroxy-3-(pyridin-2-yl)-*2H*-chromen-2-one 4-(*7*-OH)

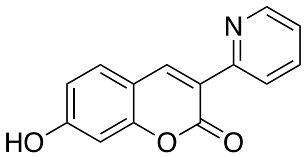

To a round bottomed flask, equipped with a magnetic stirrer bar, was added 2,4-dihydroxybenzaldehyde (5.07 mmol, 0.700 g, 1.0 eq.), pyridine-2-acetonitrile (5.07 mmol, 0.565 mL, 1.0 eq.) and piperidine (0.05 mmol, 0.05 mL, 0.01 eq.) in ethanol (6 mL). The solution was heated to reflux for 1 h, solution was left to stir at room temperature for a further 18 h. To the solution, 5% sulfuric acid (7 mL) was added and resulting solution stirred at reflux for 6 h. The solution was then neutralised with aqueous ammonium hydroxide until pH = 7. The Precipitate was then filtered by vacuum filtration and solid was washed with cold water (25 mL) solid was allowed to dry for 2 h. Solid was then dissolved in minimal hot methanol, solution then cooled over 12 h and crystals of **4-(*7*-OH)** collected then dried *in vacuo*. (0.753 g, 62%). MP 254–255 °C. ^{1}H NMR (400 MHz, DMSO-d_6) δ 10.81 (s, 1H), 8.82 (s, 1H), 8.69 – 8.65 (m, 1H), 8.25 (dd, J = 8.1, 1.1 Hz, 1H), 7.88 (td, J = 7.8, 1.9 Hz, 1H), 7.77 (d, J = 8.5 Hz, 1H), 7.38 (ddd, J = 7.5, 4.8, 1.1 Hz, 1H), 6.84 (dd, J = 8.5, 2.3 Hz, 1H), 6.78 (d, J = 2.2 Hz, 1H). ^{13}C NMR (101 MHz, DMSO-d_6) δ 162.2, 159.9, 155.6, 151.5, 149.3, 143.0, 136.7, 131.0, 123.1, 123.0, 119.9, 113.8, 111.8, 101.7. IR (solid-state ATR, cm^{-1}) 3272, 3045, 1702, 1680, 1615, 1577, 1432 ,1300, 1214, 1162, 795. MS; HRMS (ESI+) m/z: [M-H]+ calcd for $C_{14}H_{10}NO_3$ 240.0655; Found 240.0657.

7-(Hydroxy)-3-(pyridin-2-yl)-*2H*-chromen-2-one-tetracarbonyl manganese 6-(*7*-OH)

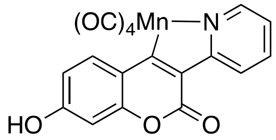

To a flame-dried Schlenk tube under N_2, equipped with a magnetic stirrer bar, was added 7-(hydroxy)-3-(pyridin-2-yl)coumarin (0.22 mmol, 0.053 g, 1.0 eq.) and benzylmanganese pentacarbonyl (0.22 mmol, 0.063 g, 1.0 eq.), followed by dry toluene (6 mL). The solution was heated to 95 °C with stirring, which was left to continue stirring for a further 2.5 h. Upon completion, the reaction was cooled to room temperature and the mixture concentrated *in vacuo.* The product **6-(*7*-OH)** was afforded as the product as a light brown solid. (0.083 g, 93%). ^{1}H NMR (400 MHz, Acetonitrile-d_3) δ 8.92 (d, J = 8.4 Hz, 1H), 8.87 (d, J = 5.6 Hz, 1H), 8.18 (d, J = 8.8 Hz, 1H), 7.95 (t, J = 7.8 Hz, 1H), 7.26 (t, J = 6.6 Hz, 1H), 6.92 (d, J = 8.8 Hz, 1H), 6.77 (s, 1H). No ^{13}C NMR data could be obtained of **6-(*7*-OH)** in chloroform, methylene chloride, methanol, acetonitrile, acetone or DMSO. IR (solid-state ATR, cm^{-1}) 2920, 2162, 2085, 1985, 1917,1721, 1701, 1160, 1606, 1437, 1238, 1125, 863, 793, 741. MS; HRMS (ESI+) m/z: [M-Na]+ calcd for $C_{18}H_8MnNNaO_7$ 427.9573; Found 427.9571.

7-(Hydroxy)-5-oxo-12-phenyl-5*H*-chromeno[3,4-*a*]quinolizin-13-ium manganese tricarbonyl 5a-(*7*-OH)

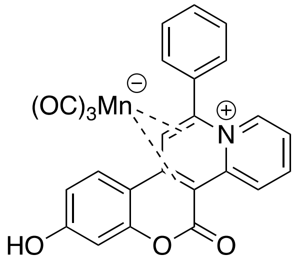

To a flame-dried Schlenk tube under N_2, equipped with a magnetic stirrer bar, was added (7-(hydroxy)-3-(pyridin-2-yl)coumarin)-tetracarbonyl manganese (0.116 mmol, 0.049 g, 1.0 eq.) in dry Et_2O (6 mL). To the solution, phenylacetylene (0.174 mmol, 0.019 mL, 1.5 eq.) and trimethylamine *N*-oxide (0.116 mmol, 0.087 g, 1.0 eq.) were added solution was heated to 80 ºC, with the aid of a water-filled cold-finger and solution left stirring for a further 18 h. Upon cooling to room temperature, the reaction mixture was diluted with dichloromethane (15 mL) and the solution concentrated *in vacuo* to give product **5a-(*7*-OH)** as a light brown powder (0.044 g, 94%). ^{1}H NMR (400 MHz, Acetonitrile-d_3) δ 7.99 (d, J = 8.5 Hz, 1H), 7.63 (d, J = 8.3 Hz, 1H), 7.57 (d, J = 7.9 Hz, 1H), 7.52 (d, J = 9.8 Hz, 1H), 7.43 – 7.33 (m, 4H), 7.31 – 7.20 (m, 2H), 6.78 (t, J = 7.0 Hz, 1H), 6.66 – 6.61 (m, 1H), 6.50 (s, 1H), 3.30 (s, 1H). ^{13}C NMR (126 MHz, Methylene Chloride-d_2) δ 231.7, 223.0, 218.4, 165.4, 156.7, 153.0, 139.4, 137.6, 137.6, 137.5, 134.1, 130.4, 130.0,

129.2, 128.7, 124.3, 122.7, 120.9, 113.5, 104.7, 101.6, 90.4, 73.9. IR (solid-state ATR, cm^{-1}) 3212, 1989, 1899, 1876, 1712, 1592, 1489, 1426, 1198, 1130, 1011, 814, 618. MS; HRMS (ESI–) m/z: [M]– calcd for $C_{25}H_{14}MnNO_6$ 478.0129; Found 478.0128.

7-Methoxy-3-(pyridin-2-yl)-*2H*-chromen-2-one 4-(*7*-OMe)

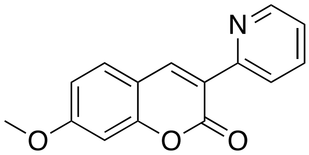

To a round bottomed flask, equipped with a magnetic stirrer bar, was added 2-hydroxy-4-methoxybenzaldehyde (5.0 mmol, 0.760 g, 1.0 eq.), pyridine-2-acetonitrile (5.0 mmol, 0.557 mL, 1.0 eq.) and piperidine (0.05 mmol, 0.05 mL, 0.01 eq.) in ethanol (5 mL). The solution was heated to reflux for 2.5 h. To the solution, 3% hydrochloric acid (9 mL) was added and resulting solution stirred at reflux for 6 h. The solution was then neutralised with aqueous ammonium hydroxide until pH = 7. The precipitate was then filtered by vacuum filtration and solid was washed with cold water (25 mL) product **4-(*7*-OMe)** was allowed to dry for 2 h. (1.18 g, 93%). MP 152–154 °C. ^{1}H NMR (400 MHz, Chloroform-*d*) δ 8.79 (s, 1H), 8.68 (ddd, *J* = 4.8, 1.9, 0.9 Hz, 1H), 8.43 (d, *J* = 8.1 Hz, 1H), 7.81 (app.t, *J* = 7.7 Hz, 1H), 7.57 (d, *J* = 8.6 Hz, 1H), 7.31 (dd, *J* = 7.7, 4.8 Hz, 1H), 6.90 (dd, *J* = 8.5, 2.4 Hz, 1H), 6.87 (d, *J* = 2.6 Hz, 1H), 3.91 (s, 3H). ^{13}C NMR (101 MHz, Chloroform-*d*) δ 163.4, 160.8, 156.0, 151.8, 149.4, 142.7, 136.7, 130.0, 123.8, 123.1, 121.9, 113.4, 113.2, 100.3, 55.9. IR (solid-state ATR, cm^{-1}) 3029, 1729, 1609, 1584, 1556, 1506, 1439, 1199, 993, 888, 794, 723, 622, 481. MS; HRMS (ESI+) m/z: [M-H]+ calcd for $C_{15}H_{12}NO_3$ 254.0812; Found 254.0812.

7-(Methoxy)-3-(pyridin-2-yl)-*2H*-chromen-2-one-tetracarbonyl manganese 6-(*7*-OMe)

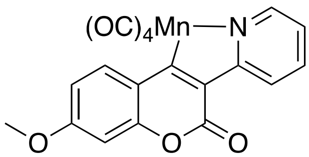

To a flame-dried Schlenk tube under N_2, equipped with a magnetic stirrer bar, was added 7-(methoxy)-3-(pyridin-2-yl)coumarin (0.22 mmol, 0.056 g, 1.0 eq.) and benzylmanganese pentacarbonyl (0.22 mmol, 0.063 g, 1.0 eq.), followed by dry toluene (6 mL). The solution was heated to 95 °C with stirring, which was left to continue stirring for a further 2.5 h. Upon completion, the reaction was cooled to room temperature and the mixture concentrated *in vacuo.* The product **6-(*7*-OMe)** was afforded as the product as a light brown solid. (0.090 g, 98%). ^{1}H NMR (400 MHz, Methylene Chloride-d_2) δ 9.00 (d, *J* = 8.0 Hz, 1H), 8.82 (d, *J* =

4.9 Hz, 1H), 8.22 (d, *J* = 8.9 Hz, 1H), 7.88 (ddd, *J* = 8.8, 7.5, 1.6 Hz, 1H), 7.19 (ddd, *J* = 7.3, 5.7, 1.5 Hz, 1H), 6.97 (dd, *J* = 8.9, 2.6 Hz, 1H), 6.86 (d, *J* = 2.6 Hz, 1H), 3.93 (s, 3H). ^{13}C NMR (151 MHz, Methylene Chloride-d_2) δ 219.4, 214.9, 212.0, 165.8, 163.9, 154.7, 153.6, 152.6, 138.9, 135.2, 129.7, 129.5, 124.9, 124.5, 122.7, 112.9, 100.5, 56.5. IR (solid-state ATR, cm^{-1}) 3062, 2081, 1993, 1973, 1941, 1921, 1691, 1594, 1436, 1360, 1140, 795, 628. MS; HRMS (ESI+) m/z: [M-H]+ calcd for $C_{19}H_{11}MnNO_7$ 419.9910; Found 419.9916.

7-(Methoxy)-5-oxo-12-phenyl-5*H*-chromeno[3,4-*a*]quinolizin-13-ium manganese tricarbonyl 5a-(7-OMe)

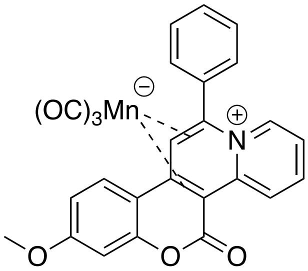

To a flame-dried Schlenk tube under N_2, equipped with a magnetic stirrer bar, was added (7-(methoxy)-3-(pyridin-2-yl)coumarin)-tetracarbonyl manganese (0.116 mmol, 0.049 g, 1.0 eq.) in dry Et_2O (6 mL). To the solution, phenylacetylene (0.174 mmol, 0.019 mL, 1.5 eq.) and trimethylamine *N*-oxide (0.116 mmol, 0.087 g, 1.0 eq.) were added solution was heated to 80 ºC, with the aid of a water-filled cold-finger and solution left stirring for a further 18 h. Upon cooling to room temperature, the reaction mixture was diluted with dichloromethane (15 mL) and the solution concentrated *in vacuo* to give product **5a-(7-OMe)** as a light brown powder (0.032 g, 56%). ^{1}H NMR (400 MHz, Methylene Chloride-d_2) δ 8.04 (d, *J* = 8.6 Hz, 1H), 7.81 (d, *J* = 8.2 Hz, 1H), 7.66 (d, *J* = 7.7 Hz, 1H), 7.56 (t, *J* = 7.8 Hz, 2H), 7.52 – 7.46 (m, 2H), 7.39 (d, *J* = 6.8 Hz, 2H), 6.83 (dd, *J* = 8.6, 2.6 Hz, 1H), 6.77 (t, *J* = 6.9 Hz, 1H), 6.73 (d, *J* = 2.5 Hz, 1H), 6.52 (s, 1H), 3.83 (s, 3H). 13C NMR (126 MHz, Methylene Chloride-d_2) δ 231.5, 222.8, 218.4, 165.2, 162.0, 156.6, 153.0, 139.5, 137.6, 134.1, 130.4, 130.0, 129.3, 128.7, 124.1, 122.7, 121.0, 114.5, 111.6, 102.5, 101.0, 90.4, 74.1, 56.3. IR (solid-state ATR, cm^{-1}) 2966, 2838, 1988, 1905, 1881, 1698, 1611, 1584, 1439, 1305, 1087, 1001, 911, 820, 798, 773, 736. MS; HRMS (ESI+) m/z: [M-H]+ calcd for $C_{19}H_{11}MnNO_6$ 403.9961; Found 403.9966.

3-(Pyridin-2-yl)-*2H*-chromen-2-one. 4-(H)

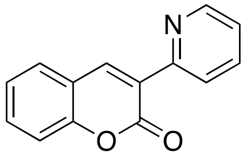

To a round bottomed flask, equipped with a magnetic stirrer bar, was added salicylaldehyde (8.47 mmol, 1.03 g, 1.0 eq.), pyridine-2-acetonitrile (8.47 mmol, 1.00 g, 1.0 eq.) and sodium hydroxide (2.10 mmol, 0.084 g, 0.25 eq.) in water (42 mL). The solution was heated to 90 °C for 3 h, solution was left to cool to room temperature. Precipitate was then filtered by gravity filtration and solid was allowed to dry for 2 h. Solid was then dissolved in minimal hot ethanol, solution then cooled over 12 h and crystals of **4-(H)** collected then dried *in vacuo*. (0.560 g, 30%). MP 141-142 °C. ^{1}H NMR (400 MHz, Chloroform-*d*) δ 8.77 (s, 1H), 8.69 (ddd, J = 4.8, 1.9, 0.9 Hz, 1H), 8.42 (dt, J = 8.1, 1.0 Hz, 1H), 7.80 (ddd, J = 8.1, 7.5, 1.9 Hz, 1H), 7.65 (dd, J = 7.7, 1.6 Hz, 1H), 7.57 (ddd, J = 8.4, 7.3, 1.6 Hz, 1H), 7.41 – 7.36 (m, 1H), 7.35 – 7.28 (m, 2H). ^{13}C NMR (101 MHz, Chloroform-*d*) δ 160.5, 154.0, 151.4, 149.5, 142.6, 136.8, 132.3, 129.0, 125.5, 124.8, 124.2, 123.6, 119.6, 116.5. IR (solid-state ATR, cm^{-1}) 3052, 3001, 1719, 1660, 1605, 1576, 1356, 1108, 960, 797, 757, 530. MS; HRMS (ESI+) m/z: [M-H]+ calcd for $C_{14}H_{10}NO_2$ 224.0706; Found 224.0707.

3-(Pyridin-2-yl)coumarin)-tetracarbonyl manganese 6-(H)

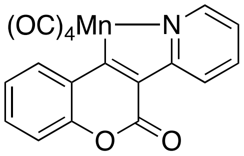

To a flame-dried Schlenk tube under N_2, equipped with a magnetic stirrer bar, was added 3-(pyridin-2-yl)coumarin (0.224 mmol, 0.050 g, 1.0 eq.) and benzylmanganese pentacarbonyl (0.224 mmol, 0.064 g, 1.0 eq.), followed by dry toluene (3.3 mL). The solution was heated to 95 °C with stirring, which was left to continue stirring for a further 2.5 h. Upon completion, the reaction was cooled to room temperature and the mixture concentrated *in vacuo.* The crude product was dissolved in dichloromethane (10 mL) and solution was filtered through a plug of Celite^{TM}. The filtrate was concentrated *in vacuo* to afford the product **6-(H)** as a cream solid. (0.080 g, 93%). MP decomp. 197 °C. ^{1}H NMR (400 MHz, Methylene Chloride-*d*$_2$) δ 9.07 (d, J = 8.3 Hz, 1H), 8.88 – 8.84 (m, 1H), 8.33 (dd, J = 8.0, 1.5 Hz, 1H), 7.92 (ddd, J = 8.7, 7.5, 1.7 Hz, 1H), 7.60 (ddd, J = 8.5, 7.2, 1.5 Hz, 1H), 7.44 – 7.33 (m, 2H), 7.24 (ddd, J = 7.3, 5.6, 1.5 Hz, 1H). ^{13}C NMR (101 MHz, Methylene-Chloride-*d*$_2$) δ 165.4, 154.4, 153.8, 139.1, 136.4, 133.9, 132.8, 132.8, 130.1, 125.4, 124.8, 123.3, 117.1, 116.3. IR (solid-state ATR, cm^{-1}) 2075, 1971, 1928, 1683, 1594, 1574, 1472, 1448,

1341, 1265, 1168, 1104, 985, 748, 635, 512, 448. MS; HRMS (ESI+) m/z: [M-H]+ calcd for $C_{18}H_9MnNO_6$ 389.9809; Found 389.9805.

12-Phenyl-5*H*-chromeno[3,4-*a*]quinolizin-13-ium manganese tricarbonyl 5a-(H)

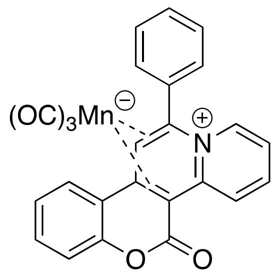

To a flame-dried Schlenk tube under N_2, equipped with a magnetic stirrer bar, was added (3-(pyridin-2-yl)coumarin)-tetracarbonyl manganese (0.263 mmol, 0.102 g, 1.0 eq.) in dry Et_2O (9 mL). To the solution, phenylacetylene (0.394 mmol, 0.043 mL, 1.5 eq.) and trimethylamine *N*-oxide (0.263 mmol, 0.020 g, 1.0 eq.) were added solution was heated to 80 ºC, with the aid of a water-filled cold-finger and solution left stirring for a further 20 h. Upon cooling to room temperature, the reaction mixture was diluted with dichloromethane (15 mL) and the solution concentrated *in vacuo* to give product as a red brown solid. Product was purified by dissolving crude in dichloromethane (5 mL) then precipitating out the product with hexane (15 mL). Product **5a-(H)** then isolated through filtration (0.107 g, 88%). ^{1}H NMR (600 MHz, Methylene Chloride-d_2) δ 8.18 (dd, *J* = 7.7, 1.6 Hz, 1H), 7.86 (dd, *J* = 8.4, 0.8 Hz, 1H), 7.72 – 7.70 (m, 1H), 7.61 (dt, *J* = 8.4, 1.7 Hz, 1H), 7.58 (ddd, *J* = 8.5, 7.4, 1.4 Hz, 1H), 7.53 (tdd, *J* = 6.7, 5.7, 1.1 Hz, 2H), 7.45 – 7.40 (m, 3H), 7.30 (td, *J* = 7.5, 1.2 Hz, 1H), 7.21 (dd, *J* = 8.2, 1.2 Hz, 1H), 6.81 (ddd, *J* = 7.7, 6.5, 1.5 Hz, 1H), 6.63 (s, 1H). 13C NMR (126 MHz, Methylene Chloride-d_2) δ 231.4, 222.4, 218.3, 165.1, 156.6, 151.7, 139.7, 137.7, 137.2, 134.1, 130.6, 130.5, 130.1, 129.4, 128.8, 124.8, 123.2, 122.8, 121.1, 117.6, 100.1, 90.6, 75.0. IR (solid-state ATR, cm^{-1}) 2927, 1983, 1883, 1710, 1607, 1587, 1485, 1319, 1229, 1158, 1114, 860, 761, 644. MS; HRMS (ESI+) m/z: [M–Na]+ calcd for $C_{25}H_{14}MnNNaO_5$ 486.0145; Found 486.0146.

7-Methyl-3-(pyridin-2-yl)-*2H*-chromen-2-one 4-(*7*-Me)

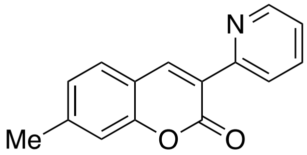

To a round bottomed flask, equipped with a magnetic stirrer bar, was added 2-hydroxy-4-methylbenzaldehyde (3.67 mmol, 0.500 g, 1.0 eq.), pyridine-2-acetonitrile (3.67 mmol, 0.434 mL, 1.0 eq.) and piperidine (0.05 mmol, 0.05 mL, 0.01 eq.) in ethanol (5 mL). The solution was heated to reflux for 2.5 h. To the solution, 3% hydrochloric acid (8 mL) was added and resulting solution stirred at reflux for 6 h. The solution was then neutralised with aqueous ammonium hydroxide until pH = 7. The Precipitate was then filtered by vacuum filtration and solid was washed with cold water (25 mL) product **4-(*7*-Me)** was allowed to dry for 2 h. (0.590 g, 68%). MP 146–147 °C. ^{1}H NMR (400 MHz, Chloroform-*d*) δ 8.75 (s, 1H), 8.68 (d, *J* = 5.4 Hz, 1H), 8.41 (d, *J* = 8.1 Hz, 1H), 7.79 (td, *J* = 7.8, 1.8 Hz, 1H), 7.53 (d, *J* = 7.9 Hz, 1H), 7.30 (ddd, *J* = 7.6, 4.8, 1.1 Hz, 1H), 7.19 (s, 1H), 7.16 – 7.10 (m, 1H), 2.48 (s, 3H). ^{13}C NMR (101 MHz, Chloroform-*d*) δ 160.6, 154.2, 151.5, 149.3, 143.8, 142.8, 136.93, 128.7, 126.0, 124.1, 124.0, 123.4, 117.2, 116.6, 22.1. IR (solid-state ATR, cm^{-1}) 3059, 1712, 1672, 1620, 1608, 1576, 1554, 1462, 1435, 1284, 1236, 1192, 1090, 945, 795, 742. MS; HRMS (ESI+) m/z: [M-H]+ calcd for $C_{15}H_{12}NO_2$ 238.0863; Found 238.0865.

7-(Methyl)-3-(pyridin-2-yl)-*2H*-chromen-2-one-tetracarbonyl manganese 6-(*7*-Me)

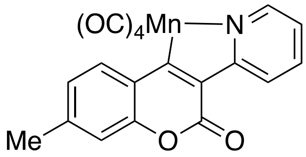

To a flame-dried Schlenk tube under N_2, equipped with a magnetic stirrer bar, was added 7-(methyl)-3-(pyridin-2-yl)coumarin (0.22 mmol, 0.052 g, 1.0 eq.) and benzylmanganese pentacarbonyl (0.22 mmol, 0.063 g, 1.0 eq.), followed by dry toluene (6 mL). The solution was heated to 95 °C with stirring, which was left to continue stirring for a further 2.5 h. Upon completion, the reaction was cooled to room temperature and the mixture concentrated *in vacuo*. The crude product was afforded as the product **6-(*7*-Me)** as a light brown solid. (0.087 g, 98 %) ^{1}H NMR (400 MHz, Methylene Chloride-*d*$_2$) δ 9.07 – 9.00 (m, 1H), 8.84 (s, 1H), 8.23 – 8.15 (m, 1H), 7.95 – 7.84 (m, 1H), 7.26 – 7.20 (m, 2H), 7.18 (s, 1H), 2.50 (s, 3H). ^{13}C NMR (151 MHz, Methylene Chloride-*d*$_2$) δ 219.5, 214.9, 211.9, 165.6, 154.7, 153.7, 150.8, 144.4, 139.0, 133.7, 131.8, 128.0, 126.1, 125.2, 123.0, 117.1, 21.8. IR (solid-state ATR, cm^{-1}) 3066, 2081, 1996, 1972, 1912,

1688, 1595, 1566, 1470, 1340, 1313, 1295, 1267, 1162, 1106, 795, 648. MS; HRMS (ESI+) m/z: [M-H]+ calcd for $C_{19}H_{11}MnNO_6$ 403.9961; Found 403.9966.

7-(Methyl)-5-oxo-12-phenyl-5*H*-chromeno[3,4-*a*]quinolizin-13-ium manganese tricarbonyl 5a-(*7*-Me)

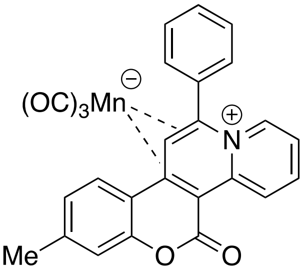

To a flame-dried Schlenk tube under N_2, equipped with a magnetic stirrer bar, was added (7-(methyl)-3-(pyridin-2-yl)coumarin)-tetracarbonyl manganese (0.076 mmol, 0.031 g, 1.0 eq.) in dry Et_2O (8 mL). To the solution, phenylacetylene (0.114 mmol, 0.013 mL, 1.5 eq.) and trimethylamine *N*-oxide (0.076 mmol, 0.006 g, 1.0 eq.) were added solution was heated to 80 ºC, with the aid of a water-filled cold-finger and solution left stirring for a further 20 h. Upon cooling to room temperature, the reaction mixture was diluted with dichloromethane (15 mL) and the solution concentrated *in vacuo* to give product as a red brown solid. Product was purified by dissolving crude in dichloromethane (5 mL) then precipitating out the product with hexane (15 mL). Product **5a-(*7*-Me)** then isolated through filtration (0.009 g, 26%). ^{1}H NMR (400 MHz, Methylene Chloride-d_2) δ 8.05 (d, *J* = 7.9 Hz, 1H), 7.84 (d, *J* = 8.3 Hz, 1H), 7.69 (d, *J* = 8.1 Hz, 1H), 7.62 – 7.56 (m, 2H), 7.55 – 7.51 (m, 2H), 7.42 (d, *J* = 6.2 Hz, 2H), 7.12 (d, *J* = 7.9 Hz, 1H), 7.03 (s, 1H), 6.88 – 6.70 (m, 1H), 6.59 (s, 1H), 2.43 (s, 3H). ^{13}C NMR (126 MHz, Methylene Chloride-d_2) δ 231.5, 222.7, 218.4, 165.2, 156.7, 151.7, 141.5, 139.6, 137.6, 137.3, 134.1, 130.4, 130.1, 129.3, 128.7, 125.7, 122.9, 122.8, 121.0, 117.9, 100.5, 90.5, 74.6, 21.8. IR (solid-state ATR, cm^{-1}) 2962, 1982, 1885, 1713, 1608, 1579, 1566, 1442, 1354, 1298, 1117, 1025, 990, 860, 817. MS; HRMS (ESI+) m/z: [M-Na]+ calcd for $C_{26}H_{16}MnNNaO_5$ 500.0301; Found 500.0306.

7-(Methoxymethoxy)-3-(pyridin-2-yl)-*2H*-chromen-2-one 4-(*7*-OMOM)

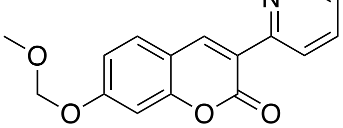

To a round bottomed flask, equipped with a magnetic stirrer bar, was added 7-hydroxy-3-(pyridin-2-yl)-*2H*-chromen-2-one (0.418 mmol, 0.100 g, 1.0 eq.), potassium carbonate (0.836 mmol, 0.116 g, 2.0 eq.) in

acetone (15 mL). To the solution chloromethyl methyl ether (0.440 mmol, 0.035 mL, 1.06 eq.), was added *via* syringe slowly. The solution was heated to reflux for 3 h, reaction was monitored by TLC upon completion solution was left to cool to room temperature. The solution was then filtered through a glass cinter by vacuum filtration to remove remaining potassium carbonate, and filtrate was concentrated *in vacuo*. The product **4-(*7*-OMOM)** was afforded an orange solid. (0.117 g, 98%). MP 106–108 °C. ^{1}H NMR (400 MHz, Chloroform-*d*) δ 8.76 (s, 1H), 8.67 (d, *J* = 4.8 Hz, 1H), 8.42 (d, *J* = 8.1 Hz, 1H), 7.79 (t, *J* = 7.8 Hz, 1H), 7.56 (d, *J* = 8.6 Hz, 1H), 7.32 – 7.27 (m, 1H), 7.07 (d, *J* = 2.4 Hz, 1H), 7.00 (dd, *J* = 8.6, 2.3 Hz, 1H), 5.26 (s, 2H), 3.51 (s, 3H). ^{13}C NMR (101 MHz, Chloroform-*d*) δ 160.9, 160.7, 155.6, 151.7, 149.4, 142.6, 136.8, 130.1, 123.9, 123.2, 122.5, 114.2, 114.1, 103.3, 94.6, 56.5. IR (solid-state ATR, cm^{-1}) 3060, 2921, 2853, 2832, 1722, 1705, 1606, 1580, 1436, 1362, 1255, 1237, 1156, 884, 633, 620. MS; HRMS (ESI+) m/z: [M-H]+ calcd for $C_{16}H_{14}NO_4$ 284.0917; Found 284.0921.

7-(Methoxymethoxy)-3-(pyridin-2-yl)-*2H*-chromen-2-one-tetracarbonyl manganese 6-(*7*-OMOM)

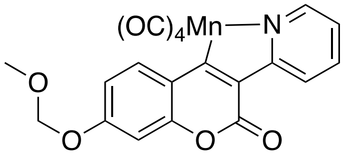

To a flame-dried Schlenk tube under N_2, equipped with a magnetic stirrer bar, was added 7-(methoxymethoxy)-3-(pyridin-2-yl)coumarin (0.22 mmol, 0.062 g, 1.0 eq.) and benzylmanganese pentacarbonyl (0.22 mmol, 0.063 g, 1.0 eq.), followed by dry toluene (6 mL). The solution was heated to 95 °C with stirring, which was left to continue stirring for a further 2.5 h. Upon completion, the reaction was cooled to room temperature and the mixture concentrated *in vacuo.* The crude product was dissolved in dichloromethane (15 mL) and filtered through a pad of celite®, filtrate was then concentrated *in vacuo* to afford the product **6-(*7*-OMOM)** as a yellow oil. (0.089 g, 90 %). ^{1}H NMR (600 MHz, Methylene Chloride-*d*$_2$) δ 9.02 (ddd, *J* = 8.4, 1.5, 0.8 Hz, 1H), 8.83 (ddd, *J* = 5.7, 1.6, 0.8 Hz, 1H), 8.24 (d, *J* = 8.9 Hz, 1H), 7.89 (ddd, *J* = 8.4, 7.4, 1.6 Hz, 1H), 7.20 (ddd, *J* = 7.3, 5.6, 1.5 Hz, 1H), 7.08 (dd, *J* = 8.9, 2.6 Hz, 1H), 7.01 (d, *J* = 2.5 Hz, 1H), 5.29 (s, 2H), 3.51 (s, 3H). ^{13}C NMR (151 MHz, Methylene Chloride-*d*$_2$) δ 219.3, 214.8, 211.9, 165.7, 161.3, 154.6, 153.7, 152.3, 139.0, 135.2, 130.2, 129.1, 126.4, 125.0, 122.8, 113.9, 103.3, 95.0, 56.8. IR (thin-film ATR, cm^{-1}) 2926, 2106, 2083, 1980, 1936, 1702, 1597, 1569, 1468, 1415, 1268, 1156, 849, 797. MS; HRMS (ESI+) m/z: [M-H]+ calcd for $C_{20}H_{13}MnNO_8$ 450.0016; Found 450.0018.

7-(Methoxymethoxy)-5-oxo-12-phenyl-5*H*-chromeno[3,4-*a*]quinolizin-13-ium manganese tricarbonyl 5a-(*7*-OMOM)

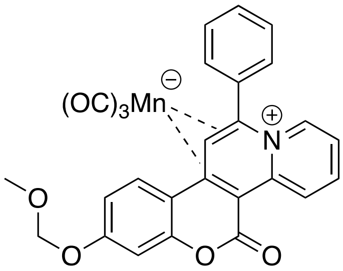

To a flame-dried Schlenk tube under N_2, equipped with a magnetic stirrer bar, was added (7-(methoxymethoxy)-3-(pyridin-2-yl)coumarin)-tetracarbonyl manganese (0.092 mmol, 0.041 g, 1.0 eq.) in dry Et_2O (8 mL). To the solution, phenylacetylene (0.132 mmol, 0.014 mL, 1.5 eq.) and trimethylamine *N*-oxide (0.092 mmol, 0.007 g, 1.0 eq.) were added solution was heated to 80 ºC, with the aid of a water-filled cold-finger and solution left stirring for a further 20 h. Upon cooling to room temperature, the reaction mixture was diluted with dichloromethane (15 mL) and the solution concentrated *in vacuo* to give product as a red brown solid. Product was purified by dissolving crude in dichloromethane (5 mL) then precipitating out the product with hexane (15 mL). Product **5a-(*7*-OMOM)** then isolated through filtration (0.046 g, 90%) ^{1}H NMR (400 MHz, Methylene Chloride-d_2) δ 8.07 (d, *J* = 8.6 Hz, 1H), 7.85 – 7.81 (m, 1H), 7.74 – 7.64 (m, 1H), 7.64 – 7.46 (m, 4H), 7.46 – 7.38 (m, 2H), 6.98 (dd, *J* = 8.5, 2.5 Hz, 1H), 6.89 (d, *J* = 2.4 Hz, 1H), 6.84 – 6.76 (m, 1H), 6.56 (s, 1H), 5.28 – 5.16 (m, 2H), 3.49 (s, 3H). ^{13}C NMR (126 MHz, Methylene Chloride-d_2) δ 231.4, 222.7, 218.3, 165.1, 159.5, 156.6, 152.8, 139.5, 137.6, 137.3, 134.1, 130.4, 130.1, 129.3, 128.7, 124.1, 122.7, 121.0, 115.8, 113.1, 105.2, 100.7, 95.2, 90.4, 74.3, 56.7, 55.7. IR (solid-state ATR, cm^{-1}) 2928, 1994, 1902, 1661, 1597, 1564, 1500, 1466, 1314, 1265, 1153, 1075, 1009, 839, 794. MS; HRMS (ESI+) m/z: [M-Na]+ calcd for $C_{27}H_{18}MnNNaO_7$ 546.0356; Found 546.0363.

7-Bromo-3-(pyridin-2-yl)-*2H*-chromen-2-one 4-(*7*-Br)

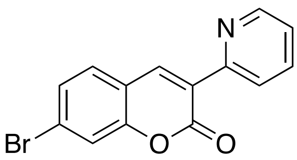

To a round bottomed flask, equipped with a magnetic stirrer bar, was added 4-bromosalicylaldehyde (2.00 mmol, 0.402 g, 1.0 eq.), pyridine-2-acetonitrile (2.00 mmol, 0.22 mL, 1.0 eq.) and piperidine (0.05 mmol, 0.05 mL, 0.01 eq.) in ethanol (5 mL). The solution was heated to reflux for 2.5 h. To the solution, 3% hydrochloric acid (8 mL) was added and resulting solution stirred at reflux for 6 h. The solution was then neutralised with aqueous ammonium hydroxide until pH = 7. The Precipitate was then filtered by vacuum

filtration and solid was washed with cold water (25 mL) solid **4-(*7*-Br)** was allowed to dry for 2 h. (0.484 g, 80%). MP 140–141 °C. ^{1}H NMR (400 MHz, Chloroform-*d*) δ 8.76 (s, 1H), 8.69 (dd, *J* = 4.9, 1.0 Hz, 1H), 8.42 (dd, *J* = 8.1, 1.2 Hz, 1H), 7.82 (tt, *J* = 7.8, 1.5 Hz, 1H), 7.57 (s, 1H), 7.54 – 7.48 (m, 1H), 7.45 (dt, *J* = 8.3, 1.5 Hz, 1H), 7.36 – 7.31 (m, 1H). ^{13}C NMR (101 MHz, Chloroform-*d*) δ 159.7, 154.1, 150.9, 149.4, 141.9, 137.1, 129.9, 128.3, 126.4, 125.4, 124.3, 123.9, 119.8, 118.5. IR (solid-state ATR, cm^{-1}) 3052, 1730, 1683, 1619, 1586, 1557, 1482, 1470, 1432, 1407, 1135, 1065, 966, 794. MS; HRMS (ESI+) m/z: [M(^{79}Br)-H]+ calcd for $C_{14}H_9BrNO_2$ 301.9811; Found 301.9810.

7-(Bromo)-3-(pyridin-2-yl)-*2H*-chromen-2-one-tetracarbonyl manganese 6-(*7*-Br)

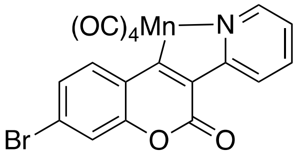

To a flame-dried Schlenk tube under N_2, equipped with a magnetic stirrer bar, was added 7-(bromo)-3-(pyridin-2-yl)coumarin (0.22 mmol, 0.067 g, 1.0 eq.) and benzylmanganese pentacarbonyl (0.22 mmol, 0.063 g, 1.0 eq.), followed by dry toluene (6 mL). The solution was heated to 95 °C with stirring, which was left to continue stirring for a further 2.5 h. Upon completion, the reaction was cooled to room temperature and the mixture concentrated *in vacuo.* The crude product was dissolved in dichloromethane (15 mL) and filtered through a pad of celite®, filtrate was then concentrated *in vacuo* to afford the product **6-(*7*-Br)** as a brown oil. (0.101 g, 98%). ^{1}H NMR (400 MHz, Methylene Chloride-*d*$_2$) δ 9.04 (d, *J* = 8.4 Hz, 1H), 8.85 (s, 1H), 8.18 (d, *J* = 8.3 Hz, 1H), 7.93 (t, *J* = 8.1 Hz, 1H), 7.56 (s, 1H), 7.53 (d, *J* = 9.8 Hz, 1H), 7.34 – 7.19 (m, 1H). ^{13}C NMR (126 MHz, Methylene Chloride-*d*$_2$) δ 219.3, 218.7, 214.6, 211.4, 165.2, 153.9, 153.7, 151.1, 139.2, 135.0, 132.9, 129.2, 129.1, 128.1, 126.5, 125.5, 123.5, 120.2. IR (thin-film ATR, cm^{-1}) 3066, 2083, 1999, 1972, 1940, 1920, 1720, 1703, 1683, 1565, 1470, 1434, 1333, 1291, 1105, 983, 810, 795, 669. MS; HRMS (ESI+) m/z: [M(^{81}Br)-H]+ calcd for $C_{18}H_8BrMnNO_6$ 467.8910; Found 467.8909.

7-(Bromo)-5-oxo-12-phenyl-5*H*-chromeno[3,4-*a*]quinolizin-13-ium manganese tricarbonyl 5a-(*7*-Br)

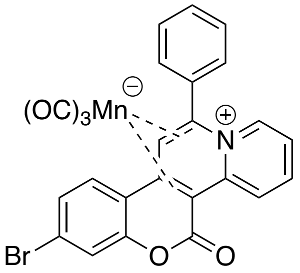

To a flame-dried Schlenk tube under N_2, equipped with a magnetic stirrer bar, was added (7-(bromo)-3-(pyridin-2-yl)coumarin)-tetracarbonyl manganese (0.0192 mmol, 0.009 g, 1.0 eq.) in dry Et_2O (8 mL). To the solution, phenylacetylene (0.029 mmol, 0.003 mL, 1.5 eq.) and trimethylamine *N*-oxide (0.0192 mmol, 0.001 g, 1.0 eq.) were added solution was heated to 80 ºC, with the aid of a water-filled cold-finger and solution left stirring for a further 20 h. Upon cooling to room temperature, the reaction mixture was diluted with dichloromethane (15 mL) and the solution concentrated *in vacuo* to give product as a red brown solid. Product was purified by dissolving crude in dichloromethane (5 mL) then precipitating out the product with hexane (15 mL). Product **5a-(*7*-Br)** then isolated through filtration (0.004 g, 36%). ^{1}H NMR (400 MHz, Methylene Chloride-d_2) δ 8.04 (d, J = 8.3 Hz, 1H), 7.84 (d, J = 8.4 Hz, 1H), 7.70 (d, J = 7.7 Hz, 1H), 7.62 – 7.56 (m, 1H), 7.57 – 7.51 (m, 2H), 7.51 – 7.47 (m, 1H), 7.47 – 7.40 (m, 2H), 7.38 (s, 1H), 7.34 (d, J = 6.9 Hz, 1H), 6.83 (t, J = 7.0 Hz, 1H), 6.59 (s, 1H). ^{13}C NMR (126 MHz, Methylene Chloride-d_2) δ 230.8, 221.8, 217.7, 164.5, 156.0, 151.1, 139.1, 137.1, 136.6, 133.5, 130.0, 129.9, 129.5, 128.8, 128.2, 124.3, 122.6, 122.3, 120.5, 117.0, 99.5, 90.1, 74.4. IR (solid-state ATR, cm^{-1}) 2961, 1987, 1904, 1884, 1714, 1602, 1582, 1565, 1481, 1415, 1313, 1269, 1155, 1070, 979, 850, 771, 676. MS; HRMS (ESI+) m/z: [M(^{81}Br)-Na]+ calcd for $C_{25}H_{13}BrMnNNaO_5$ 563.9250; Found 563.9257.

7-Nitro-3-(pyridin-2-yl)-*2H*-chromen-2-one 4-(*7*-NO_2)

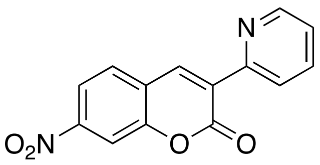

To a round bottomed flask, equipped with a magnetic stirrer bar, was added 4-nitrosalicylaldehyde (1.80 mmol, 0.300 g, 1.0 eq.), pyridine-2-acetonitrile (1.80 mmol, 0.20 mL, 1.0 eq.) and piperidine (0.05 mmol, 0.05 mL, 0.01 eq.) in ethanol (5 mL). The solution was heated to reflux for 2.5 h. To the solution, 3% hydrochloric acid (6 mL) was added and resulting solution stirred at reflux for 6 h. The solution was then neutralised with aqueous ammonium hydroxide until pH = 7. The Precipitate was then filtered by vacuum filtration and solid was washed with cold water (25 mL) solid **4-(*7*-NO_2)** was allowed to dry for 2 h. (0.352

g, 73%). MP 163–164 °C. ^{1}H NMR (400 MHz, Chloroform-*d*) δ 8.85 (s, 1H), 8.72 (ddd, *J* = 4.8, 1.9, 0.9 Hz, 1H), 8.45 (d, *J* = 8.1 Hz, 1H), 8.23 (d, *J* = 2.2 Hz, 1H), 8.18 (dd, *J* = 8.5, 2.2 Hz, 1H), 7.86 – 7.82 (m, 1H), 7.82 (d, *J* = 8.2, Hz, 1H), 7.38 (ddd, *J* = 7.6, 4.7, 1.1 Hz, 1H).^{13}C NMR (101 MHz, Chloroform-*d*) δ 159.0, 153.4, 150.1, 149.8, 149.3, 137.0, 129.7, 128.5, 124.6, 124.5, 124.4, 119.4, 112.2. IR (solid-state ATR, cm^{-1}) 3048, 1734, 1587, 1520, 1472, 1346, 1287, 1140, 1122, 795, 753. MS; HRMS (ESI+) m/z: [M-H]+ calcd for $C_{14}H_9N_2O_4$ 269.0557; Found 269.0552.

7-(Nitro)-3-(pyridin-2-yl)-*2H*-chromen-2-one-tetracarbonyl manganese 6-(*7*-NO$_2$)

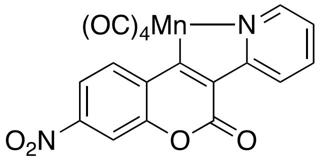

To a flame-dried Schlenk tube under N_2, equipped with a magnetic stirrer bar, was added 7-(nitro)-3-(pyridin-2-yl)coumarin (0.314 mmol, 0.085 g, 1.0 eq.) and benzylmanganese pentacarbonyl (0.314 mmol, 0.090 g, 1.0 eq.), followed by dry toluene (5 mL). The solution was heated to 95 °C with stirring, which was left to continue stirring for a further 2.5 h. Upon completion, the reaction was cooled to room temperature and the mixture concentrated *in vacuo.* The product **6-(*7*-NO$_2$)** was afforded as a deep brown solid. (0.124 g, 91 %) ^{1}H NMR (400 MHz, Methylene Chloride-*d*$_2$) δ 9.09 (d, *J* = 8.3 Hz, 1H), 8.90 (d, *J* = 5.8 Hz, 1H), 8.48 (dd, *J* = 9.4, 2.5 Hz, 1H), 8.27 – 8.14 (m, 2H), 7.97 (t, *J* = 7.8 Hz, 1H), 7.33 (dd, *J* = 7.7, 5.5 Hz, 1H). ^{13}C NMR (151 MHz, Methylene Chloride-*d*$_2$) δ 219.2, 216.9, 214.4, 211.0, 164.5, 154.1, 153.2, 150.6, 149.5, 139.5, 135.5, 134.7, 134.6, 129.1, 126.2, 124.3, 119.0, 112.7. IR (solid-state ATR, cm^{-1}) 3091, 3066, 2088, 2001, 1982, 1928, 1715, 1565, 1345, 1149, 990, 618. MS; HRMS (ESI+) m/z: [M-H]+ calcd for $C_{18}H_8MnN_2O_8$ 434.9656; Found 434.9647.

7-(Nitro)-5-oxo-12-phenyl-5*H*-chromeno[3,4-*a*]quinolizin-13-ium manganese tricarbonyl 5a-(*7*-NO$_2$)

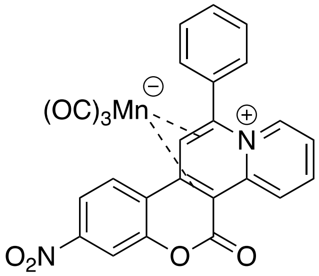

To a flame-dried Schlenk tube under N_2, equipped with a magnetic stirrer bar, was added (7-(nitro)-3-(pyridin-2-yl)coumarin)-tetracarbonyl manganese (0.104 mmol, 0.045 g, 1.0 eq.) in dry Et_2O (8 mL). To the solution, phenylacetylene (0.156 mmol, 0.017 mL, 1.5 eq.) and trimethylamine *N*-oxide (0.104 mmol,

0.008 g, 1.0 eq.) were added solution was heated to 80 ºC, with the aid of a water-filled cold-finger and solution left stirring for a further 20 h. Upon cooling to room temperature, the reaction mixture was diluted with dichloromethane (15 mL) and the solution concentrated *in vacuo* to give product as a red brown solid. Product was purified by dissolving crude in dichloromethane (5 mL) then precipitating out the product with hexane (15 mL). Product **5a-(*7*-NO$_2$)** then isolated through filtration (0.033 g, 63 %). ^{1}H NMR (600 MHz, Methylene Chloride-*d*$_2$) δ 8.29 (d, *J* = 8.5 Hz, 1H), 8.14 (dd, *J* = 8.6, 2.3 Hz, 1H), 8.03 (d, *J* = 2.2 Hz, 1H), 7.87 (d, *J* = 8.2 Hz, 1H), 7.71 (d, *J* = 8.1 Hz, 1H), 7.67 – 7.60 (m, 2H), 7.58 – 7.49 (m, 2H), 7.50 – 7.42 (m, 2H), 6.94 – 6.84 (m, 1H), 6.66 (s, 1H). ^{13}C NMR (126 MHz, Methylene Chloride-*d*$_2$) δ 164.0, 155.8, 151.5, 148.6, 140.3, 133.0, 136.5, 133.9, 130.6, 130.2, 129.7, 128.8, 123.7, 123.0, 121.7, 119.9, 113.1, 96.6, 91.3, 76.5. IR (solid-state ATR, cm^{-1}) 3105, 1989, 1890, 1714, 1571, 1517, 1339, 1231, 995, 834, 627. MS; HRMS (ESI+) m/z: [M-Na]+ calcd for $C_{25}H_{13}MnN_2NaO_7$ 530.995; Found 531.0005.

6,7-Methylenedioxy-(pyridin-2-yl)-*2H*-chromen-2-one 4-(*6,7*-methylenedioxy)

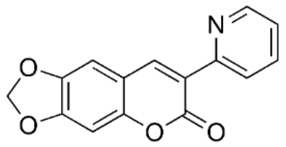

To a round bottomed flask, equipped with a magnetic stirrer bar, was added 4,5-methylenedioxy-2-hydroxybenzaldehyde (0.309 mmol, 0.051 g, 1.0 eq.), pyridine-2-acetonitrile (0.309 mmol, 0.034 mL, 1.0 eq.) and piperidine (4 drops) in ethanol (3 mL). The solution was heated to reflux for 4 h. To the solution, 3% hydrochloric acid (10 mL) was added and resulting solution stirred at reflux for overnight. The solution was then neutralised with aqueous ammonium hydroxide until pH = 7. The precipitate was then filtered by vacuum filtration and solid was washed with cold water (25 mL) product **4-(*6,7*-methylenedioxy)** was allowed to dry for 2 h. (0.050 g, 61%). ^{1}H NMR (400 MHz, Chloroform-*d*) δ 8.68 (s, 1H), 8.66 (dt, *J* = 4.8, 1.1 Hz, 1H), 8.41 (d, *J* = 8.1 Hz, 1H), 7.82 – 7.73 (m, 1H), 7.31 – 7.27 (m, 1H), 6.98 (s, 1H), 6.88 (s, 1H), 6.10 (s, 2H). ^{13}C NMR (101 MHz, Chloroform-*d*) δ 160.7, 152.0, 151.6, 151.5, 149.4, 145.2, 142.8, 136.8, 123.9, 123.2, 122.1, 113.6, 105.8, 102.5, 98.0. IR (solid-state ATR, cm^{-1}) 3102, 1700, 1631, 1568, 1307, 924, 752, 707, 680. MS; HRMS (ESI+) m/z: [M-H]+ calcd for $C_{15}H_{10}NO_4$ 268.0604; Found 268.0605.

6,7-Methylenedioxy-3-(pyridin-2-yl)-*2H*-chromen-2-one-tetracarbonyl manganese 6-(*6,7*-methylenedioxy)

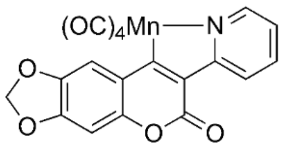

To a flame-dried Schlenk tube under N_2, equipped with a magnetic stirrer bar, was added 7-(pyridin-2-yl)-6*H*-[1,3]dioxolo[4,5-*g*]chromen-6-one (0.174 mmol, 0.046 g, 1.0 eq.) and benzylmanganese pentacarbonyl (0.174 mmol, 0.050 g, 1.0 eq.), followed by dry toluene (5 mL). The solution was heated to 95 ºC with stirring, which was left to continue stirring for a further 2.5 h. Upon completion, the reaction was cooled to room temperature and the mixture concentrated *in vacuo*. The product **6-(6,7-methylenedioxy)** was afforded as a dark brown solid. (0.074 g, 98%.) ^{1}H NMR (400 MHz, Methylene Chloride-d_2) δ 9.01 (d, *J* = 8.6 Hz, 1H), 8.82 (d, *J* = 5.0 Hz, 1H), 7.88 (td, *J* = 7.8, 1.6 Hz, 1H), 7.70 (s, 1H), 7.20 (ddd, *J* = 7.2, 5.6, 1.5 Hz, 1H), 6.86 (s, 1H), 6.12 (s, 2H). ^{13}C NMR (151 MHz, Methylene Chloride-d_2) δ 219.8, 218.5, 214.8, 211.9, 165.8, 153.6, 152.1, 147.9, 145.7, 139.0, 129.8, 129.1, 126.4, 125.0, 124.8, 122.8, 110.8, 103.2, 97.8. IR (solid-state ATR, cm^{-1}) 2915, 2107, 2079, 1997, 1976, 1905, 1702, 1599, 1502, 1466, 1402, 1389, 1271, 1246, 1182, 1139, 985, 823, 797. MS; HRMS (ESI+) m/z: [M-Na]+ calcd for $C_{19}H_8MnNNaO_8$ 455.9523; Found 455.9500.

6,7-Methylenedioxy-5-oxo-12-phenyl-5*H*-chromeno[3,4-*a*]quinolizin-13-ium manganese tricarbonyl 5a-(*6,7*-methylenedioxy)

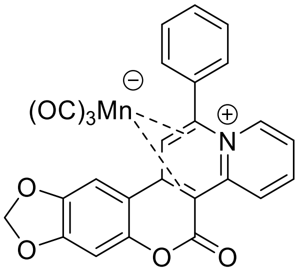

To a flame-dried Schlenk tube under N_2, equipped with a magnetic stirrer bar, was added (7,8-methylenedioxy)-3-(pyridin-2-yl)coumarin)-tetracarbonyl manganese (0.081 mmol, 0.035 g, 1.0 eq.) in dry $^{n}Bu_2O$ (4 mL). To the solution, phenylacetylene (0.122 mmol, 0.013 mL, 1.5 eq.) and TMNO (0.108 mmol, 0.008 g, 1.0 eq.) was added and resulting solution heated to 80 ºC, left to continue stirring for a further 18 h. Upon cooling to room temperature, the reaction mixture was diluted with dichloromethane (15 mL) and the solution concentrated *in vacuo*. The precipitate was then collected by filtration. Product was not further purified, giving product **5a-(*6,7*-methylenedioxy)** as a brown powder (0.038 g, 91%). ^{1}H NMR (400 MHz, Methylene Chloride-d_2) δ 7.84 (d, *J* = 8.3 Hz, 1H), 7.75 – 7.65 (m, 1H), 7.60 – 7.47 (m, 5H), 7.43 – 7.38 (m, 2H), 6.86 – 6.76 (m, 1H), 6.73 (s, 1H), 6.47 (s, 1H), 6.08 (d, *J* = 1.3 Hz, 1H), 6.05 (d, *J* = 1.3 Hz, 1H).

^{13}C NMR (126 MHz, Methylene Chloride-*d*$_2$) δ 231.4, 222.7, 218.4, 165.1, 156.5, 149.9, 147.2, 145.0, 139.5, 137.6, 137.3, 134.1, 130.4, 130.0, 129.3, 128.7, 122.7, 121.0, 114.6, 102.8, 102.0, 101.2, 99.4, 90.2. IR (solid-state ATR, cm^{-1}) 1986, 1886, 1708, 1484, 1465, 1441, 1342, 1252, 1114, 1033, 934, 770, 700. MS; HRMS (ESI+) m/z: [MH]+ calcd for $C_{26}H_{15}MnNO_7$ 508.0223; Found 508.0226.

7-(Methoxy)-9-(nitro)-(pyridin-2-yl)-*2H*-chromen-2-one 4-(*6*-NO$_2$,*8*-OMe)

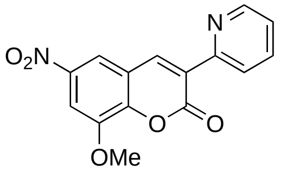

To a round bottomed flask, equipped with a magnetic stirrer bar, was added 3-methoxy-5-nitrosalicylaldehyde (0.76 mmol, 0.150 g, 1.0 eq.), pyridine-2-acetonitrile (0.76 mmol, 0.085 mL, 1.0 eq.) and piperidine (4 drops) in ethanol (5 mL). The solution was heated to reflux for 4 h. To the solution, 3% hydrochloric acid (10 mL) was added and resulting solution stirred at reflux for overnight. The solution was then neutralised with aqueous ammonium hydroxide until pH = 7. The precipitate was then filtered by vacuum filtration and solid was washed with cold water (25 mL) product **4-(*6*-NO$_2$,*8*-OMe)** was allowed to dry for 2 h. (0.180 g, 79%). MP 249–250 °C. ^{1}H NMR (400 MHz, Chloroform-*d*) δ 8.84 (s, 1H), 8.72 – 8.69 (m, 1H), 8.43 (*d*, J = 7.9 Hz, 1H), 8.19 (*d*, J = 2.5 Hz, 1H), 7.94 (*d*, J = 2.4 Hz, 1H), 7.82 (td, *J* = 7.8, 1.9 Hz, 1H), 7.38 – 7.32 (m, 1H), 4.10 (s, 3H). 13C NMR (101 MHz, Chloroform-*d*) δ 158.5, 150.2, 149.8, 147.7, 147.4, 144.3, 141.3, 137.0, 127.6, 124.3, 119.6, 116.0, 107.9, 57.0. IR (solid-state ATR, cm^{-1}) 3101, 3058, 1725, 1606, 1527, 1479, 1438, 1362, 1200, 1102, 993, 900, 741. MS; HRMS (ESI+) m/z: [M-H]+ calcd for $C_{15}H_{11}N_2O_5$ 299.0662; Found 299.0666.

7-(Methoxy)-9-(nitro)-3-(pyridin-2-yl)-*2H*-chromen-2-one-tetracarbonyl manganese 6-(*6*-NO$_2$,*8*-OMe)

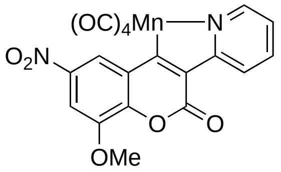

To a flame-dried Schlenk tube under N_2, equipped with a magnetic stirrer bar, was added 7-(methoxy)-9-(nitro)-3-(pyridin-2-yl)coumarin (0.218 mmol, 0.065 g, 1.0 eq.) and benzylmanganese pentacarbonyl (0.218 mmol, 0.062 g, 1.0 eq.), followed by dry toluene (6 mL). The solution was heated to 95 ºC with stirring, which was left to continue stirring for a further 2.5 h. Upon completion, the reaction was cooled to room temperature and the mixture concentrated *in vacuo*. The product **6-(*6*-NO$_2$,*8*-OMe)** was afforded as a light brown solid. (0.090 g, 89%.) ^{1}H NMR (400 MHz, Methylene Chloride-*d*$_2$) δ 9.06 (d, *J* = 8.3 Hz, 1H), 8.93 (d, *J* = 2.3 Hz, 1H), 8.90 (d, *J* = 5.8 Hz, 1H), 7.98 (d, *J* = 2.2 Hz, 1H), 7.95 (dd, *J* = 7.8, 2.1 Hz, 1H), 7.31 (ddd, *J* = 7.4, 5.6, 1.6 Hz, 1H), 4.11 (s, 3H). ^{13}C NMR (151 MHz, Methylene Chloride-*d*$_2$) δ 219.0, 214.6, 211.1, 164.7, 154.1, 152.6, 148.5, 145.1, 144.4, 139.5, 134.4, 129.8, 128.8, 125.9, 124.2, 121.2, 108.2, 57.7. IR (solid-state ATR, cm^{-1}) 2090, 2002, 1969, 1925, 1715, 1614, 1597, 1523, 1497, 1474, 1345, 1322, 1243, 1214, 1159, 1116, 956, 887, 761. MS; HRMS (ESI+) m/z: [M-Na]+ calcd for $C_{19}H_9MnN_2NaO_9$ 486.9581; Found 486.9582.

7-(Methoxy)-6-(nitro)-5-oxo-12-phenyl-5*H*-chromeno[3,4-*a*]quinolizin-13-ium manganese tricarbonyl 5a-(*6*-NO$_2$,*8*-OMe)

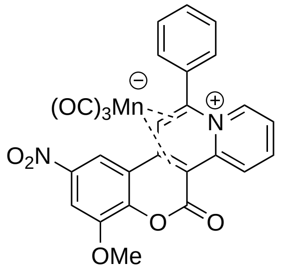

To a flame-dried Schlenk tube under N_2, equipped with a magnetic stirrer bar, was added (7-(methoxy)-6-(nitro)-3-(pyridin-2-yl)coumarin)-tetracarbonyl manganese (0.108 mmol, 0.050 g, 1.0 eq.) in dry ^{n}Bu$_2$O (5 mL). To the solution, phenylacetylene (0.162 mmol, 0.018 mL, 1.2 eq.) and TMNO (0.108 mmol, 0.008 g, 1.0 eq.) was added and resulting solution heated to 80 ºC, left to continue stirring for a further 18 h. Upon cooling to room temperature, the reaction mixture was diluted with dichloromethane (15 mL) and the solution concentrated *in vacuo*. The precipitate was then collected by filtration. Product was not further purified, giving product **5a-(*6*-NO$_2$,*8*-OMe)** as an orange powder (0.048 g, 71%). ^{1}H NMR (400 MHz, Methylene Chloride-*d*$_2$) δ 8.64 (t, *J* = 2.8 Hz, 1H), 7.83 – 7.78 (m, 2H), 7.68 (d, *J* = 7.7 Hz, 1H), 7.62 – 7.57 (m, 2H), 7.52 (t, *J* = 6.6 Hz, 2H), 7.46 – 7.38 (m, 2H), 6.84 (t, *J* = 6.9 Hz, 1H), 6.66 (d, *J* = 2.6 Hz,

1H), 4.02 (s, 3H). ^{13}C NMR (151 MHz, CD_2Cl_2) δ 230.6, 221.7, 218.1, 163.5, 155.7, 148.6, 145.4, 144.5, 140.2, 138.0, 136.6, 133.9, 130.6, 130.2, 129.7, 128.9, 123.6, 122.8, 121.6, 110.9, 107.5, 97.7, 91.0, 57.5. IR (solid-state ATR, cm^{-1}) 3100, 2930, 2862, 1992, 1896, 1728, 1610, 1588, 1569, 1363, 1197, 772. MS; HRMS (ESI+) m/z: [MH]+ calcd for $C_{26}H_{16}MnN_2O_8$ 539.0282; Found 539.0284.

8-(Diethylammonio)-5-oxo-12-phenyl-5*H*-chromeno[3,4-*a*]quinolizin-13-ium tetrachloromanganate 8a

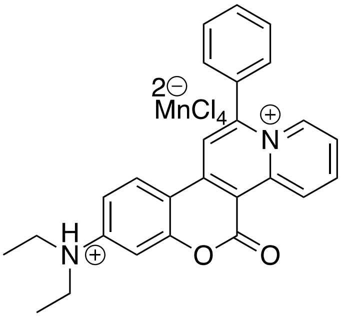

To a round bottom flask, equipped with a magnetic stirrer bar, was added 8-(diethylamino)-5-oxo-12-phenyl-5*H*-chromeno[3,4-*a*]quinolizin-13-ium tricarbonyl manganese 0.155 mmol, 0.083 g, 1.0 eq.) in chloroform (5 mL). To the solution, 4 M HCl in diethyl ether (3 mL) was added dropwise with stirring. Solution stirring continued until persistent precipitate forms and solution decolourises. Precipitate then collected by gravity filtration and solid then washed with chloroform (1 mL). Solid on filter paper then dried *in vacuo*. Product was not further purified, giving product **8a** as a bright red solid. (0.042 g, 68%). ^{1}H NMR (400 MHz, Deuterium Oxide) δ 9.81 (d, *J* = 9.0 Hz, 1H), 8.86 (d, *J* = 6.9 Hz, 1H), 8.35 – 8.27 (m, 1H), 8.17 (s, 1H), 7.84 (d, *J* = 9.4 Hz, 1H), 7.76 – 7.68 (m, 3H), 7.66 – 7.61 (m, 3H), 6.76 (d, *J* = 10.2 Hz, 1H), 6.56 (s, 1H), 3.44 (q, J = 7.2 Hz, 4H), 1.16 (t, J = 7.0 Hz, 6H). ^{13}C NMR (126 MHz, Deuterium Oxide) δ 159.2, 155.6, 153.6, 149.0, 144.2, 142.5, 140.6, 135.7, 131.9, 130.8, 130.0, 129.2, 126.4, 124.7, 122.1, 118.1, 111.3, 105.0, 102.5, 96.6, 45.1, 11.7. IR (solid-state ATR, cm^{-1}) 2973, 2931, 1717, 1597, 1495, 1436, 1352, 1268, 1077, 781. MS; HRMS (ESI+) m/z: [M]+ calcd for $C_{26}H_{23}N_2O_2$ 395.1754; Found 395.1757.

12-Phenyl-5*H*-chromeno[3,4-*a*]quinolizin-13-ium tetrachloromanganate 9a

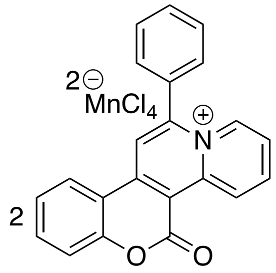

To a round bottom flask, equipped with a magnetic stirrer bar, was added 5-oxo-12- phenyl-5*H*-chromeno[3,4-*a*]quinolizin-13-ium tricarbonyl manganese 0.09 mmol, 0.082 g, 1.0 eq.) in chloroform (5 mL). To the solution, 4 M HCl in diethyl ether (3 mL) was added dropwise with stirring. Solution stirring continued until persistent precipitate forms and solution decolourises. Precipitate then collected by gravity filtration and solid then washed with chloroform (1 mL). Solid on filter paper then dried *in vacuo*. Product was not further purified, giving product **9a** as a bright orange solid. (0.028 g, 59%). ^{1}H NMR (400 MHz, Deuterium Oxide) δ 10.08 (d, *J* = 9.0 Hz, 1H), 9.04 (d, *J* = 7.0 Hz, 1H), 8.54 (s, 1H), 8.43 (t, *J* = 8.1 Hz, 1H), 8.20 (d, *J* = 8.0 Hz, 1H), 7.81 (t, *J* = 7.0 Hz, 1H), 7.70 (t, *J* = 7.8 Hz, 1H), 7.63 – 7.56 (m, 3H), 7.56 – 7.52 (m, 2H), 7.41 (d, *J* = 8.0 Hz, 2H). ^{13}C NMR (101 MHz, Methanol-d_4) δ 187.6, 157.0, 153.7, 150.5, 143.7, 140.7, 136.1, 135.6, 131.7, 131.4, 129.8, 129.4, 126.1, 125.9, 125.6, 124.0, 119.3, 117.2, 114.8, 112.7. IR (solid-state ATR, cm^{-1}) 1720, 1633, 1607, 1596, 1561, 1505, 1453, 1325, 1244, 1186, 1113, 1034, 995, 869, 800, 762. MS; HRMS (ESI+) m/z: [M]+ calcd for $C_{22}H_{14}NO_2$ 324.1019; Found 324.1013.

12-Phenyl-5*H*-chromeno[3,4-*a*]quinolizin-13-ium tetrafluoroborate 10a

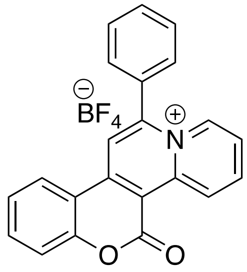

To a round bottom flask, equipped with a magnetic stirrer bar, was 5-oxo-12-phenyl-5H-chromeno[3,4-a]quinolizin-13-ium tricarbonyl manganese (0.022 mmol, 0.01 g, 1.0 eq.) in chloroform (5 mL). To the solution, tetrafluoroboric acid diethyl ether complex (2 mL) was added dropwise with stirring. Solution stirring continued until persistent precipitate forms and solution decolorises. Precipitate was gravity filtered and dried *in vacuo* to give colourless product **10a**. (0.008 g, 92%).^{1}H NMR (400 MHz, DMSO-d_6) δ 10.15 (d, *J* = 8.6 Hz, 1H), 9.11 (d, *J* = 7.0 Hz, 1H), 8.99 (s, 1H), 8.79 (d, *J* = 8.2 Hz, 1H), 8.71 (t, *J* = 8.2 Hz, 1H),

8.13 – 8.06 (m, 1H), 7.89 (t, *J* = 8.0 Hz, 1H), 7.83 – 7.72 (m, 5H), 7.66 (d, *J* = 8.3 Hz, 1H), 7.56 (t, *J* = 7.6 Hz, 1H). ^{19}F NMR (376 MHz, Methanol-d_4) δ -154.8. ^{13}C NMR (151 MHz, DMSO-d_6) δ 157.9, 153.9, 150.9, 144.1, 143.8, 142.2, 137.5, 136.6, 132.4, 132.3, 130.7, 130.6, 127.6, 126.5, 126.4, 125.4, 120.1, 118.1, 115.8, 113.3. IR (solid-state ATR, cm^{-1}) 1724, 1645, 1610, 1597, 1561, 1452, 1417, 1283, 1191, 1000, 880, 744, 699. MS; HRMS (ESI+) m/z: [M]+ calcd for $C_{22}H_{14}NO_2$ 324.1019; Found 324.1024.

Benzyl rhenium(I) pentacarbonyl

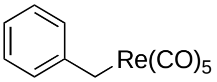

To a flame-dried Schlenk tube under N_2, 2 mL of mercury was added. To this, finely divided pieces of sodium (0.100 g, 4.35 mmol, 7.7 equiv.) were added to the mercury slowly. Once dissolved and cooled to r.t. dry THF (5 mL) was added. To the stirred solution, $Re_2(CO)_{10}$ (0.500 g, 0.766 mmol, 1.0 equiv.) was added under N_2 flow. The resulting solution was stirred over the sodium amalgam for 1.5 h. To a separate oven-dried flask under N_2 benzyl chloride was (0.18 mL, 1.53 mmol, 2.0 equiv.) added. The THF solution of $NaRe(CO)_5$ was then cannula transferred onto the benzyl chloride, taking care not to draw any amalgam. The resulting solution was then stirred for 2 h at r.t. then refluxed under N_2 for 15 mins. Once cooled to r.t. the crude material was adsorbed on to silica and purified *via* flash column chromatography eluting in 100% hexanes (R_f = 0.15). The desired product was then concentrated *in vacuo* to give product as a colourless crystalline solid, (0.468 g, 73%). ^{1}H NMR (400 MHz, Methylene Chloride-d_2) δ 7.15 (dd, *J* = 8.1, 7.2 Hz, 2H), 7.08 – 7.03 (m, 2H), 6.82 (tt, *J* = 7.2, 1.4 Hz, 1H), 2.43 (s, 2H). ^{13}C NMR (101 MHz, Methylene Chloride-d_2) δ 185.7, 181.8, 155.8, 128.7, 125.9, 122.7, -2.0. Solution Phase FTIR (hexanes) (ν / cm^{-1}): 2127, 1985, 1954, 1945, 1595, 1487, 1450, 1206, 755, 697. MS; HRMS (APCI+) m/z: [M-2CO]+ calcd for $C_{10}H_8O_3Re$ 363.0026; Found 363.0034.

3-(Pyridin-2-yl)coumarin)-tetracarbonyl rhenium 6'-(H)

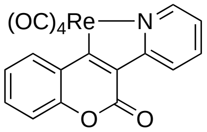

To a flame-dried Schlenk tube under N_2, equipped with a magnetic stirrer bar, was added 3-(pyridin-2-yl)coumarin (0.179 mmol, 0.040 g, 1.0 eq.) and benzyl rhenium pentacarbonyl (0.179 mmol, 0.075 g, 1.0 eq.), followed by dry toluene (5.0 mL). The solution was heated to 110 °C with stirring, which was left to continue stirring for a further 24 h. Upon completion, the reaction was cooled to room temperature, supernatant was removed *via* syringe and precipitate was washed with toluene (2 x 2 mL). Precipitate dried *in vacuo* to afford product **6'-(H)** as colourless solid. (0.0423 g, 45%) ^{1}H NMR (600 MHz, Methylene Chloride-d_2) δ 9.20 (dt, *J* = 8.5, 1.1 Hz, 1H), 8.99 – 8.94 (m, 1H), 8.25 (dd, *J* = 8.0, 1.6 Hz, 1H), 7.98 (ddd, *J* = 8.7, 7.4, 1.7 Hz, 1H), 7.58 (ddd, *J* = 8.5, 7.2, 1.6 Hz, 1H), 7.39 (ddd, *J* = 8.3, 7.2, 1.3 Hz, 1H), 7.33 (dd, *J* = 8.1, 1.3 Hz, 1H), 7.25 (ddd, *J* = 7.2, 5.6, 1.5 Hz, 1H). ^{13}C NMR (151 MHz, Methylene Chloride-d_2) δ 205.4, 193.0, 192.2, 187.1, 167.0, 156.7, 151.4, 139.6, 136.9, 134.1, 133.0, 130.5, 126.2, 124.8, 123.8, 116.8. IR (solid-state ATR, cm^{-1}) 2091, 1991, 1965, 1915, 1684, 1596, 1577, 1466, 1445, 1417, 1267, 1167, 1103, 988, 799. MS; HRMS (ESI+) m/z: [M-Na]+ calcd for $C_{18}H_9NNaO_6Re$ 543.9801; Found 543.9843.

8-(Diethylamino)-5-oxo-12-phenyl-5*H*-chromeno[3,4-*a*]quinolizine-13-ium tetrafluoroborate 10a

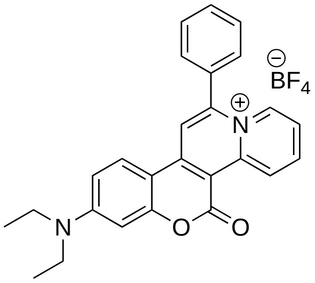

To a round bottom flask equipped with a magnetic stirbar, 8-(diethylamino)-5-oxo-12-phenyl-5*H*-chromeno[3,4-*a*]quinolizin-13-ium manganese tricarbonyl (0.037 mmol, 0.020 g, 1.0 eq.) was dissolved in dichloromethane (5 mL). Silver tetrafluoroborate (0.074 mmol, 0.014 g, 2.0 eq.) was separately dissolved in acetone (5 mL), the acetone solution was added to the dichloromethane solution. The solution was stirred for 15 mins. The reaction mixture was then filtered through a filter paper which was flushed with dichloromethane (5 mL). The filtrate was then concentrated *in vacuo* to give product **10a** as a red crystalline solid, (14.5 mg, 99%). ^{1}H NMR (400 MHz, Methylene Chloride-d_2) δ 10.15 (d, *J* = 9.2 Hz, 1H), 8.71 (d, *J* = 7.1 Hz, 1H), 8.37 – 8.24 (m, 1H), 8.08 (s, 1H), 7.88 (d, *J* = 9.6 Hz, 1H), 7.80 – 7.56 (m, 6H), 6.82 (d, *J* = 9.5 Hz, 1H), 6.61 (d, *J* = 2.3 Hz, 1H), 3.51 (d, *J* = 7.2 Hz, 4H), 1.26 (d, *J* = 7.4 Hz, 6H). ^{13}C NMR (101 MHz, Methylene Chloride-d_2) δ 158.3, 157.4, 154.8, 149.3, 145.7, 144.0, 140.8, 135.4, 132.7, 131.3, 130.9,

129.9, 127.5, 126.3, 122.7, 121.1, 119.6, 115.6, 111.8, 97.8, 46.0, 12.8. ^{11}B NMR (128 MHz, Methylene Chloride-d_2) δ -2.35 (s). ^{19}F NMR (376 MHz, Methylene Chloride-d_2) δ -153.14 (s). IR (solid-state ATR, cm^{-1}) 2982, 1721, 1632, 1594, 1532, 1496, 1469, 1448, 1428, 1352, 1233, 1197, 1152, 1064, 954, 777. MS; HRMS (ESI+) m/z: [M]+ calcd for $C_{26}H_{23}N_2O_2$ 395.1754; Found 395.1767.

7-(Methoxy)-9-(nitro)-5-oxo-12-phenyl-5*H*-chromeno[3,4-*a*]quinolizine-13-ium tetrafluoroborate 12a

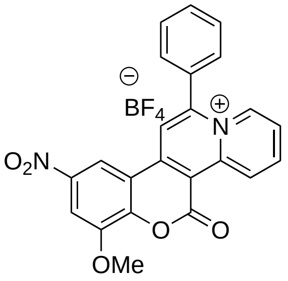

To a round bottom flask equipped with a magnetic stirbar, 7-(methoxy)-6-(nitro)-5-oxo-12-phenyl-5*H*-chromeno[3,4-*a*]quinolizin-13-ium manganese tricarbonyl (0.042 mmol, 0.023 g, 1.0 eq.) was dissolved in dichloromethane (5 mL). Silver tetrafluoroborate (0.084 mmol, 0.016 g, 2.0 eq.) was separately dissolved in acetone (5 mL), the acetone solution was added to the dichloromethane solution. The solution was stirred for 15 mins. The reaction mixture was then filtered through a filter paper which was flushed with dichloromethane (5 mL). The filtrate was then concentrated *in vacuo* to give product **12a** as a brown crystalline solid, (20.1 mg, 98%). ^{1}H NMR (400 MHz, Methylene Chloride-d_2) δ 10.42 (d, J = 9.1 Hz, 1H), 9.12 (d, J = 7.0 Hz, 1H), 8.70 (s, 1H), 8.67 – 8.54 (m, 1H), 8.46 (s, 1H), 8.14 (s, 1H), 8.10 – 7.98 (m, 1H), 7.89 – 7.66 (m, 5H), 4.15 (s, 3H). ^{13}C NMR (101 MHz, DMSO-d_6) δ 156.5, 151.0, 148.1, 147.2, 145.1, 143.1, 142.6, 142.2, 137.5, 132.1, 131.7, 130.5, 130.1, 126.1, 125.7, 120.2, 116.2, 114.1, 114.0, 111.3, 57.8. ^{11}B NMR (128 MHz, Methylene Chloride-d_2) δ -2.35. ^{19}F NMR (376 MHz, Methylene Chloride-d_2) δ -153.14. IR (solid-state ATR, cm^{-1}) 1742, 1635, 1623, 1537, 1517, 1439, 1345, 1006, 950, 878, 810, 800, 784, 739. MS; HRMS (ESI+) m/z: [M]+ calcd for $C_{23}H_{15}N_2O_5$ 399.0975; Found 399.0974.

8-(Methyl)-5-oxo-12-phenyl-5*H*-chromeno[3,4-*a*]quinolizine-13-ium tetrafluoroborate 13a

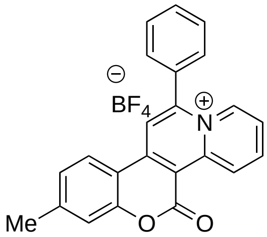

To a round bottom flask equipped with a magnetic stirbar, 8-(methyl)-5-oxo-12-phenyl-5*H*-chromeno[3,4-*a*]quinolizin-13-ium manganese tricarbonyl (0.063 mmol, 0.030 g, 1.0 eq.) was dissolved in dichloromethane (5 mL). Silver tetrafluoroborate (0.126 mmol, 0.025 g, 2.0 eq.) was separately dissolved in acetone (5 mL), the acetone solution was added to the dichloromethane solution. The solution was stirred for 15 mins. The reaction mixture was then filtered through a filter paper which was flushed with dichloromethane (5 mL). The filtrate was then concentrated *in vacuo* to give product **13a** as a brown crystalline solid, (20.9 mg, 70%). ^{1}H NMR (400 MHz, DMSO-d_6) δ 10.10 (d, J = 8.8 Hz, 1H), 9.05 (d, J = 6.8 Hz, 1H), 8.91 (s, 1H), 8.72 – 8.59 (m, 2H), 8.05 (t, J = 7.0 Hz, 1H), 7.79 – 7.69 (m, 5H), 7.47 (s, 1H), 7.38 (d, J = 8.2 Hz, 1H), 2.02 (s, 3H). ^{13}C NMR (101 MHz, DMSO-d_6) δ 156.8, 152.7, 149.6, 147.0, 142.9, 142.6, 140.9, 136.2, 132.4, 131.1, 129.5, 129.3, 126.4, 126.1, 124.9, 123.8, 118.7, 116.7, 112.0, 111.2, 42.8, 21.0. ^{11}B NMR (128 MHz, DMSO-d_6) δ -2.30. ^{19}F NMR (376 MHz, DMSO-d_6) δ -148.04. IR (solid-state ATR, cm^{-1}) 1718, 1643, 1613, 1590, 1556, 1457, 1421, 1286, 1251, 1159, 1137, 1027, 883, 865, 823, 797, 739. MS; HRMS (ESI+) m/z: [M]+ calcd for $C_{23}H_{16}NO_2$ 338.1176; Found 338.1181.

2.0 Infrared Spectroscopic Measurements

2.1 Time-Resolved Infrared Spectroscopy (TRIR) Experiments

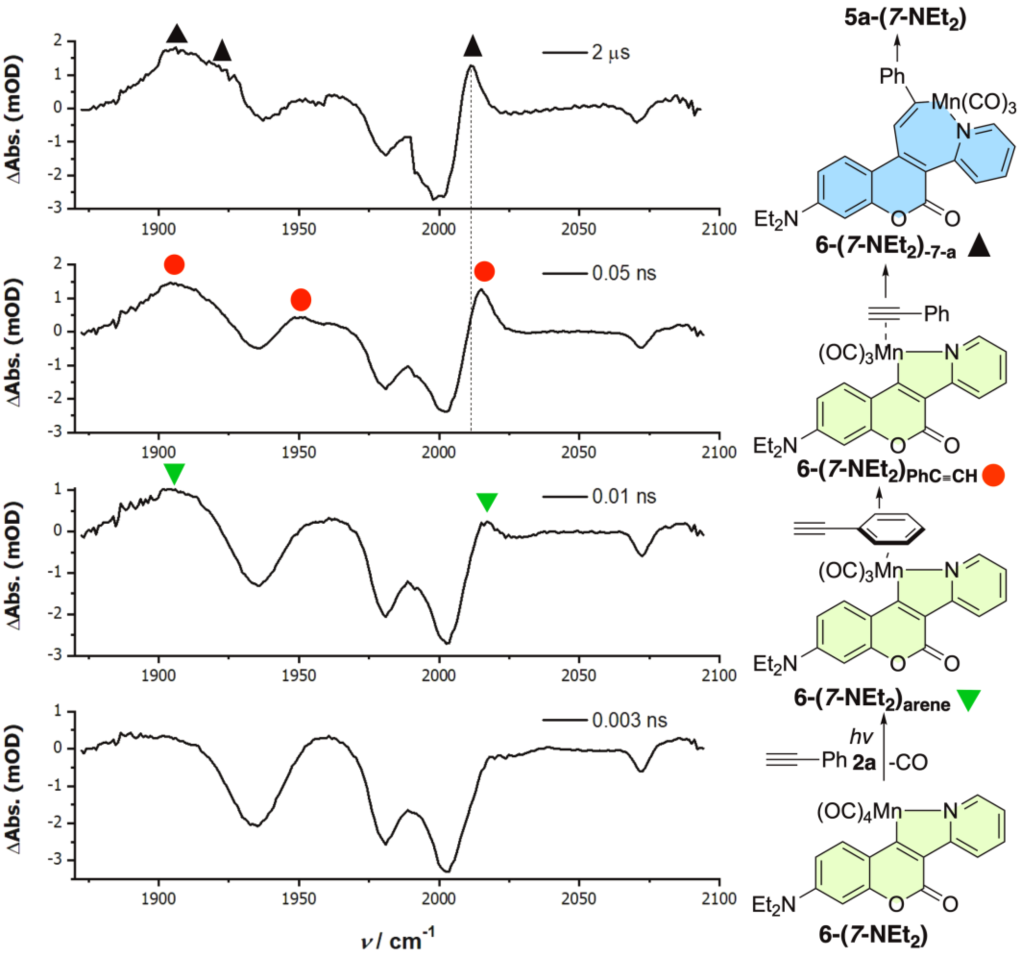

Figure S1. TRIR data for the photoactivation of **6-(7-NEt₂)** in phenylacetylene **2a** (1.52 mmol dm^{-3}). Bottom: metal carbonyl infrared spectrum at 3 ps. Third from top: metal carbonyl infrared spectrum at 10 ps showing intermediate **6-(7-NEt$_2$)$_{arene}$**. Second from the top metal carbonyl infrared spectrum at 50 ps showing intermediate **6-(7-NEt$_2$)$_{PhC=CH}$**. Top: metal carbonyl infrared spectrum at 2 µs showing intermediate **6-(7-NEt$_2$)$_{-7-a}$**. Reaction Scheme going upwards on right.

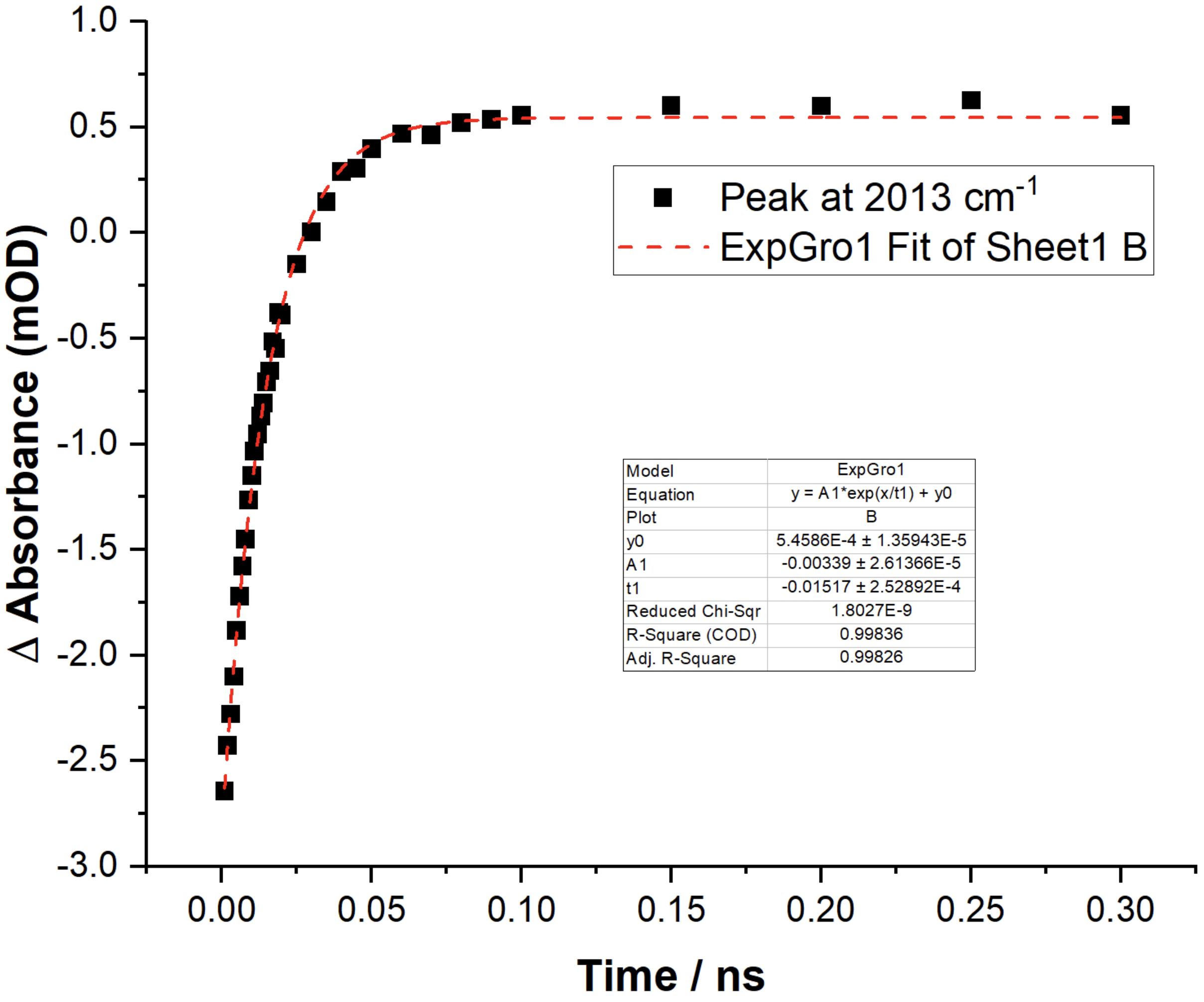

Figure S2. Kinetic plot for the reaction of **6-(*7*-NEt$_2$)** in MeCN (1.52 mmol dm^{-3}), with 20 µL phenylacetylene 2**a**. Black squares correspond to the growth of **6-(*7*-NEt$_2$)$_{MeCN}$** (band at 2013 cm^{-1}). The dashed line fits to an exponential growth function (**6-(7-NEt$_2$)$_{MeCN}$**, k_{growth} = (-6.59 ± 0.11) x 10^{10} s^{-1}). The kinetic analysis was performed using data from intensity of the peak at 2013 cm^{-1}.

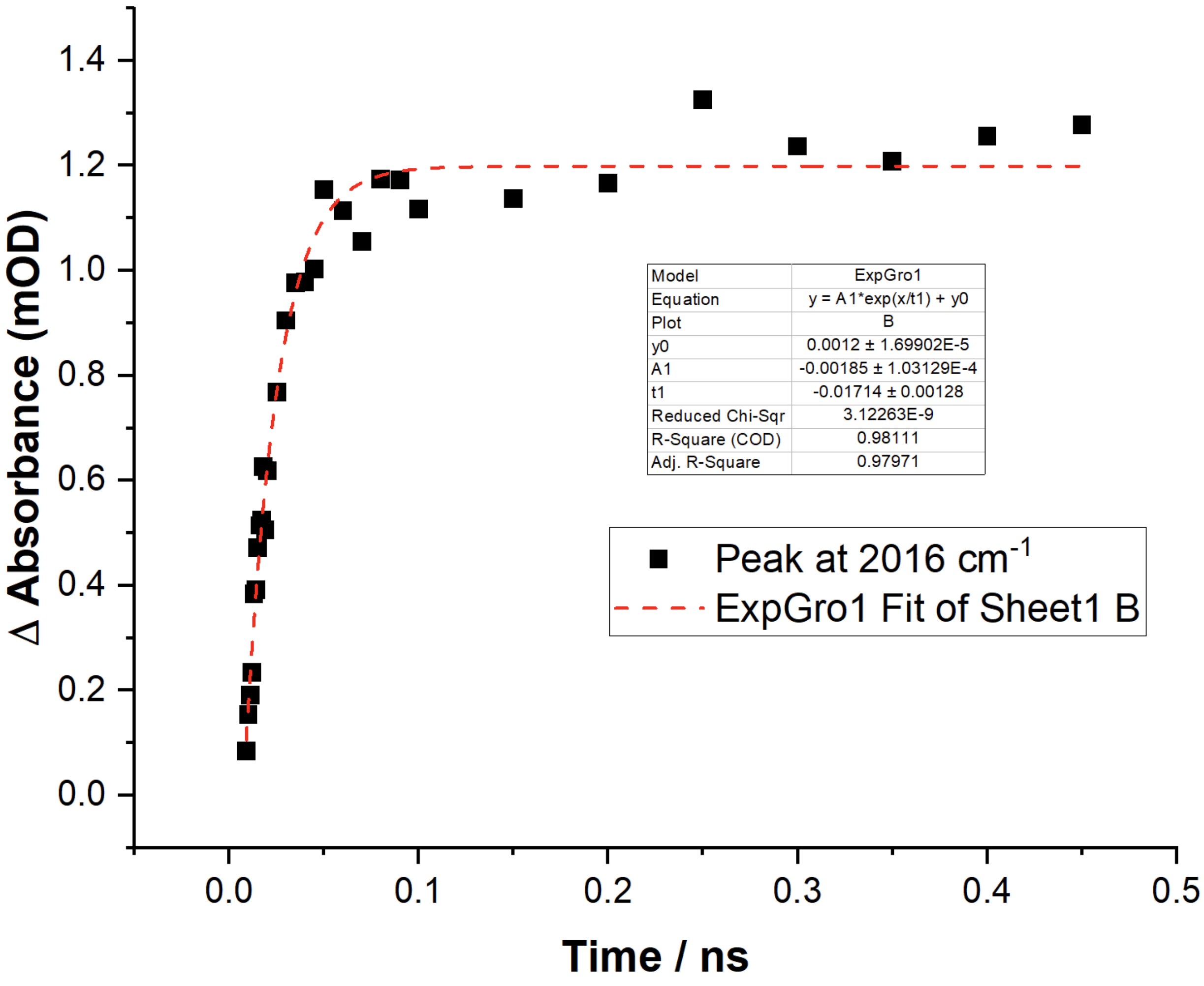

Figure S3. Kinetic plot for the reaction of **6-(7-NEt$_2$)** in phenylacetylene **2a** (1.52 mmol dm^{-3}). Black squares correspond to the growth of **6-(7-NEt$_2$)$_{arene}$** (band at 2016 cm^{-1}). The dashed line fits to an exponential growth function (**6-(7-NEt$_2$)$_{arene}$**, k_{growth} = (-5.83 ± 0.44) x 10^{10} s^{-1}). The kinetic analysis was performed using data from intensity of the peak at 2016 cm^{-1}.

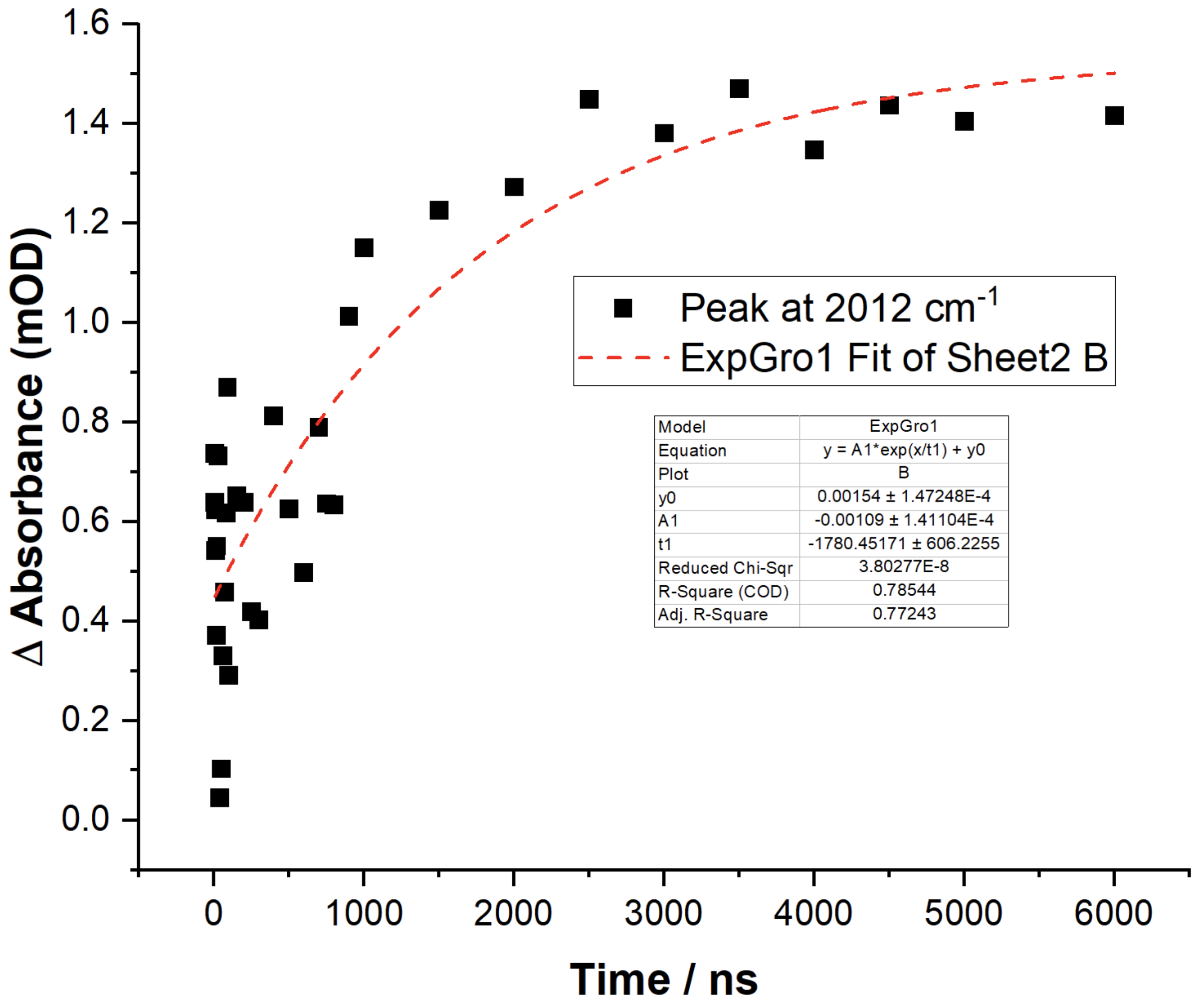

Figure S4. Kinetic plot for the reaction of **6-(7-NEt$_2$)** in phenylacetylene **2a** (1.52 mmol dm^{-3}). Black squares correspond to the growth of **6-(7-NEt$_2$)$_{-7-a}$** (band at 2012 cm^{-1}). The dashed line fits to an exponential growth function (**6-(7-NEt$_2$)$_{-7-a}$**, k_{growth} = (-5.62 ± 1.91) x 10^5 s^{-1}). The kinetic analysis was performed using data from intensity of the peak at 2012 cm^{-1}.

2.2 ReactIR (*in situ* IR) Experiments on the Second Timescale

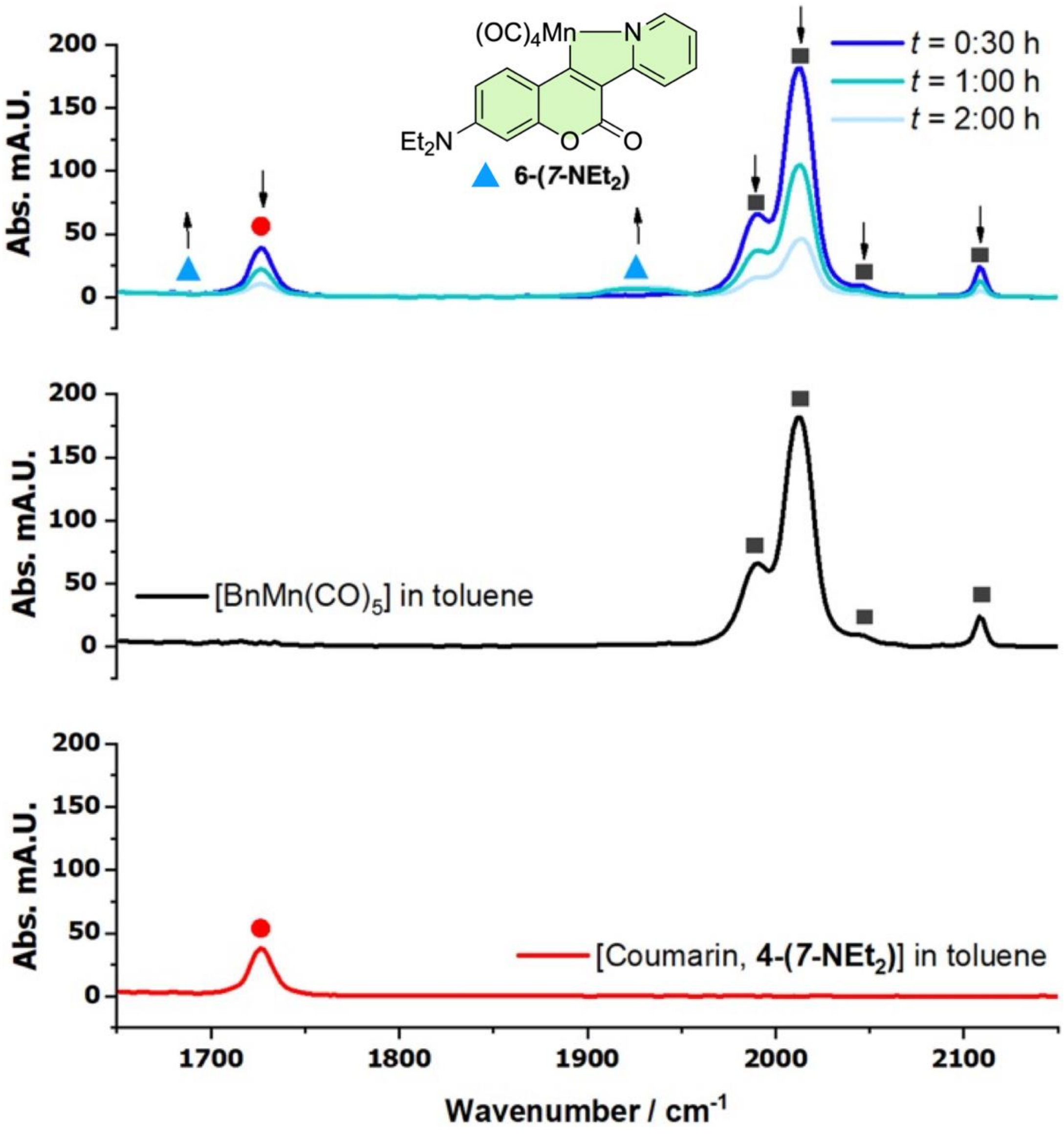

Figure S5. Top: ReactIR (*in situ* IR) data for the cyclometallation of **4-(7-NEt₂)** (red circles) with benzyl manganese pentacarbonyl (black squares), leading to formation of **6-(7-NEt₂)** (blue triangles) in dry toluene at 60 ºC. Middle: solution phase IR spectrum of benzyl manganese pentacarbonyl in dry toluene. Bottom: solution phase IR spectrum of coumarin **4-(7-NEt₂)** in dry toluene.

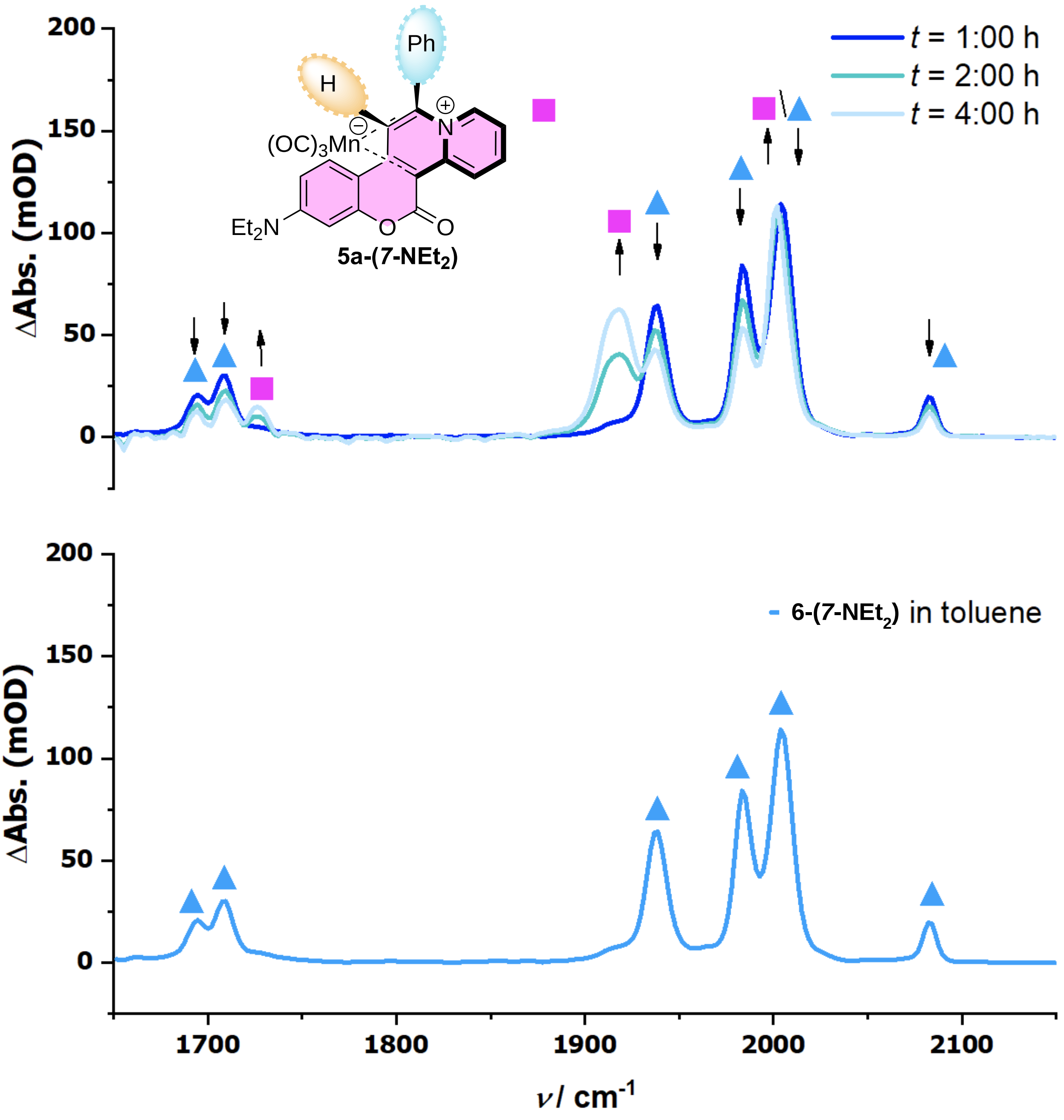

Figure S6. Top: ReactIR (*in situ* IR) data for the reductive elimination of **4-(7-NEt₂)** (blue triangles) with phenylacetylene, leading to formation of **5a-(7-NEt₂)** (magenta squares) in dry toluene at 60 ºC. Bottom: solution phase IR spectrum of coumarin **6-(7-NEt₂)** in dry toluene.

2.3 Light-Induced Reaction Time Course

Table S1: Time Vs. yield of **5a-(7-NEt$_2$)**.

Light (ON/OFF)	Reaction time / min	Yield of Product **5a-(7-NEt$_2$)** / %
(OFF)	0	0
(ON)	5	1.6
(OFF)	10	27
(ON)	15	31
(OFF)	20	47
(ON)	25	49
(OFF)	30	63
(OFF)	35	66

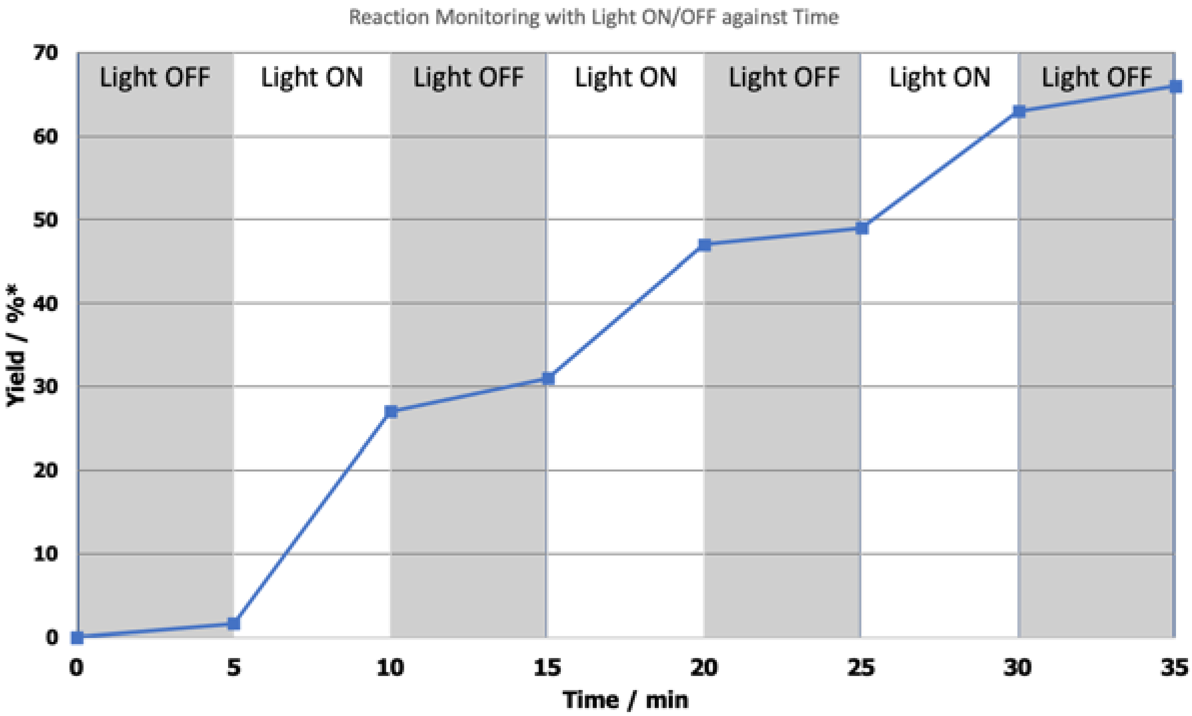

Graph S1: Time vs. yield of **5a-(7-NEt$_2$)**.

*Yield determined by ^{1}H NMR spectroscopic analysis.

3.0 Computational Calculations Using Density Functional Theory (DFT) Methods

All calculations were performed using the TURBOMOLE V6.4 package using the resolution of identity (RI) approximation.[6] Initial optimisations were performed at the (RI-)BP86/SV(P) level, followed by frequency calculations at the same level. All minima were confirmed as such by the absence of imaginary frequencies. Single-point energies were calculated on the (RI-)BP86/SV(P) optimised geometries using the hybrid PBE0 functional and the flexible def2- TZVPP basis set and corrected for dispersion effects using Grimme's D3 method with BJ dampening.[7,8] Solvation effects were modelled using COMSO[9] using the dielectronic constant of 2.38 for toluene.

Transition states were located by analysis of a trial structure with fixed atoms to find an appropriate negative vibrational frequency corresponding to the bond formation/cleavage event. The transition state was then located through an eigenvector following routine and were defined as having a single negative vibrational mode. Dynamic reaction coordinate analysis determined the nature of the states which were connected by the transition state.

Comparison of Experimental and Predicted CO bands.

$y = 0.7333x + 567.5$[5]

**Compound**	**DFT calculated CO bands** ν **/ cm**$^{-1}$	**Corrected DFT CO bands** ν **/ cm**$^{-1}$	**Experimental CO bands** ν **/ cm**$^{-1}$	**Difference in corrected CO bands to Experimental** $\Delta\nu$ **/ cm**$^{-1}$
**6-(7-NEt$_2$)**	N/A	N/A	1934, 1981, 2003, 2072	N/A
**6-(7-NEt$_2$)$_{arene}$**	1950, 1973, 2030	1885, 1917, 1994	1905, 2017	N/A,
**6-(7-NEt$_2$)$_{PhC≡CH}$**	1960, 1990, 2039	1899, 1940, 2007	1905, 1950, 2015	-6, -10, -8
**6-(7-NEt$_2$)$_{-7-a}$**	1960, 1980, 2034	1899, 1926, 2000	1907, 1922, 2011,	-8, +4, -11
**5a-(7-NEt$_2$)-iso**	1954, 1977, 2025	1891, 1922, 1988	N/A	N/A
**5a-(7-NEt$_2$)**	1965, 1968, 2026	1906, 1910, 1989	1920, 2002	N/A, -10, -13

[a] Experimental IR data recorded in solution phase of compound in phenylacetylene.

3.1 DFT xyz Coordinates and Collated Energies

6-(*7*-NEt$_2$)$_{arene}$

```
SCF Energy (au) BP86/SV(P)                        -2754.548618212
SCF Energy (au) PBE0/def2-TZVPP                   -2754.076416679
SCF Energy (au) PBE0/def2-TZVPP COSMO Toluene     -2754.0881855171
Zero Point Energy (au)                            0.4415964
Chemical potential (kJ mol-1)                     975.92
Dispersion correction (au) PBE0/def2-TZVPP        -0.09187118

xyz coordinates
60

Mn    0.1749752    2.8678909   -0.0492866
C     1.0501268    2.8809751    1.4857551
C     1.5988518    2.2391169   -0.9394425
C     0.7518630    4.5405761   -0.4732587
O     1.2016143    5.5992607   -0.6833467
O     2.5468033    1.9385480   -1.5553401
O     1.6226599    2.8804304    2.5021839
C    -2.4251327    2.6008327    1.2949543
C    -1.9871812    4.8571392    0.7768276
C    -3.2097853    5.2836887    1.3010089
C    -4.0768355    4.3121846    1.8339409
C    -3.6863911    2.9721666    1.8309185
C    -1.8749500    1.2500634    1.2701226
C    -0.5838718    1.0931967    0.7203389
C     0.0049605   -0.2212669    0.8068935
C    -0.7593237   -1.3025583    1.3492098
O    -2.0329163   -1.1161631    1.7906009
C    -2.6597529    0.1355331    1.8101121
H    -1.2774575    5.5862692    0.3568046
H    -3.4661563    6.3541684    1.2901866
H    -5.0539322    4.6005563    2.2557048
H    -4.3215793    2.1743291    2.2375424
N    -1.5967503    3.5604256    0.7571535
C    -1.0203229    3.3594409   -2.4285717
C    -0.1262860    3.5918994   -3.5070071
C     0.4437422    2.5231747   -4.1911618
C     0.1329727    1.1751648   -3.8283041
C    -0.7570629    0.9456623   -2.7614218
C    -1.3335917    2.0299429   -2.0569115
H     0.1090869    4.6254819   -3.8080516
H     1.1355348    2.7020932   -5.0290033
C     0.7073594    0.0834548   -4.5545328
H    -1.0170545   -0.0871977   -2.4842221
H    -2.1375436    1.8282745   -1.3348873
C     1.3318485   -0.5714627    0.4227112
C    -0.2676938   -2.6062328    1.4737202
O    -3.7940954    0.1506038    2.2623951
C     1.0560126   -2.9288104    1.0713344
H    -0.9453709   -3.3440861    1.9217536
H     1.9866354    0.2050766    0.0106364
C     1.8470361   -1.8545651    0.5434932
H     2.8774936   -2.0309924    0.2067743
N     1.5565398   -4.2110974    1.1847448
```

```
C     2.9507674   -4.5302652    0.8695488
C     0.7199809   -5.3258882    1.6341029
C     0.6877396   -5.5104304    3.1592011
H     1.1091522   -6.2496844    1.1516144
H    -0.3086468   -5.1923309    1.2358878
H     3.2485691   -5.3874251    1.5129901
H     3.6005880   -3.6855114    1.1827900
C     3.1945258   -4.8797709   -0.6066391
H     4.2647280   -5.1340670   -0.7740506
H     2.9321092   -4.0286523   -1.2714312
H     2.5825069   -5.7550722   -0.9175353
H     0.0514482   -6.3817029    3.4308485
H     0.2778647   -4.6110614    3.6670907
H     1.7081018   -5.6933763    3.5625981
H    -1.5772098    4.2007487   -1.9898283
C     1.1990458   -0.8440459   -5.1871874
H     1.6336500   -1.6646528   -5.7430728
```

```
$vibrational spectrum (first 50 lines)
#  mode     symmetry     wave number   IR intensity    selection rules
#                         cm**(-1)        km/mol          IR     RAMAN
     1                      0.00         0.00000         -       -
     2                      0.00         0.00000         -       -
     3                      0.00         0.00000         -       -
     4                      0.00         0.00000         -       -
     5                      0.00         0.00000         -       -
     6                      0.00         0.00000         -       -
     7         a           13.05         0.98803        YES     YES
     8         a           18.78         0.02036        YES     YES
     9         a           24.79         0.16123        YES     YES
    10         a           37.87         1.72974        YES     YES
    11         a           44.43         0.22376        YES     YES
    12         a           52.99         0.07568        YES     YES
    13         a           58.44         0.20649        YES     YES
    14         a           65.29         0.23795        YES     YES
    15         a           70.68         0.25220        YES     YES
    16         a           76.23         0.18018        YES     YES
    17         a           78.68         0.35782        YES     YES
    18         a           82.78         0.18089        YES     YES
    19         a           89.27         0.27058        YES     YES
    20         a           94.48         0.20507        YES     YES
    21         a          102.22         0.19654        YES     YES
    22         a          105.85         0.42047        YES     YES
    23         a          111.66         0.38030        YES     YES
    24         a          116.07         0.28998        YES     YES
    25         a          120.63         0.33845        YES     YES
    26         a          132.04         2.25929        YES     YES
    27         a          137.24         0.44259        YES     YES
    28         a          155.18         1.11633        YES     YES
    29         a          177.82         1.63761        YES     YES
    30         a          190.67         0.27280        YES     YES
    31         a          196.96         0.07426        YES     YES
    32         a          208.01         1.39387        YES     YES
    33         a          227.18         0.60606        YES     YES
    34         a          228.57         0.35904        YES     YES
    35         a          243.10         0.13295        YES     YES
    36         a          246.10         0.34625        YES     YES
```

37	a	265.43	0.22546	YES	YES
38	a	283.72	3.96904	YES	YES
39	a	320.43	1.11912	YES	YES
40	a	331.41	0.49089	YES	YES
41	a	353.47	0.40978	YES	YES
42	a	365.34	2.12014	YES	YES
43	a	378.25	0.05203	YES	YES
44	a	405.33	0.06015	YES	YES
45	a	406.72	3.10545	YES	YES
46	a	421.93	2.36370	YES	YES
47	a	425.66	5.33392	YES	YES
48	a	453.45	3.16349	YES	YES
49	a	457.55	0.36052	YES	YES
50	a	460.18	2.17066	YES	YES

6-(7-NEt$_2$)$_{PhC\equiv CH}$

SCF Energy (au)	BP86/SV(P)	-2754.571166854
SCF Energy (au)	PBE0/def2-TZVPP	-2754.094682905
SCF Energy (au)	PBE0/def2-TZVPP COSMO Toluene	-2754.1051695370
Zero Point Energy (au)		0.4422096
Chemical potential (kJ mol^{-1})		978.75
Dispersion correction (au)	PBE0/def2-TZVPP	-0.08654855

xyz coordinates
60

Mn	0.4412394	2.1335978	0.0766911
C	0.8558203	2.0463911	1.8267994
C	2.0667246	1.5733428	-0.4326983
C	1.0689404	3.8320471	-0.0651419
O	1.5213198	4.9065103	-0.1064979
O	3.1419873	1.3136456	-0.8121314
O	1.1289268	1.9774723	2.9559368
C	-2.4047732	1.8325727	0.8051042
C	-1.9011613	4.0911476	0.3869694
C	-3.2201825	4.4947147	0.6139863
C	-4.1674824	3.5099271	0.9455987
C	-3.7616143	2.1776492	1.0397877
C	-1.8433381	0.4891005	0.8992015
C	-0.4657178	0.3441912	0.6483279
C	0.1110083	-0.9675431	0.8171858
C	-0.7408838	-2.0639806	1.1584999
O	-2.0788608	-1.8913456	1.3335015
C	-2.7091741	-0.6442342	1.2444076
H	-1.1325196	4.8331002	0.1235097
H	-3.4872651	5.5595123	0.5311146
H	-5.2199118	3.7804363	1.1328029
H	-4.4583357	1.3689412	1.2956946
N	-1.4932610	2.8052955	0.4745941
C	-0.2212165	3.6871089	-2.9011253
C	0.9489229	4.3461493	-3.3584667
C	0.8491437	5.4860184	-4.1672519
C	-0.4119640	5.9950201	-4.5275977
C	-1.5781025	5.3524539	-4.0761273
C	-1.4892618	4.2090524	-3.2686458
H	1.9372059	3.9495031	-3.0777954
H	1.7673379	5.9844972	-4.5191921
H	-0.4850673	6.8941883	-5.1613233
H	-2.5698696	5.7439493	-4.3572557
H	-2.4008406	3.7000765	-2.9183101
C	-0.1481622	2.4867644	-2.1052212
C	-0.2752795	1.2830316	-1.7436715
H	-0.4994972	0.2409939	-1.9719939
C	1.4902811	-1.3027852	0.7008063
C	-0.2746575	-3.3696016	1.3461522
O	-3.9117611	-0.6451713	1.4523225
C	1.1053629	-3.6789423	1.2131280
H	-1.0243305	-4.1209115	1.6251048
H	2.2096909	-0.5134261	0.4534514
C	1.9790182	-2.5890036	0.8862626
H	3.0575765	-2.7543636	0.7607198
N	1.5817582	-4.9631560	1.3913502

```
C     3.0133751   -5.2706855    1.3614289
C     0.6791222   -6.0913255    1.6299533
C     0.3367621   -6.3184998    3.1105772
H     1.1654143   -7.0007162    1.2126250
H    -0.2471882   -5.9485729    1.0332170
H     3.1793638   -6.1435540    2.0307796
H     3.5784980   -4.4329264    1.8228749
C     3.5583993   -5.5813407   -0.0410902
H     4.6419315   -5.8292092    0.0090806
H     3.4325879   -4.7140994   -0.7246603
H     3.0299152   -6.4494057   -0.4933075
H    -0.3353813   -7.1979652    3.2225617
H    -0.1748257   -5.4343836    3.5481369
H     1.2542529   -6.5112540    3.7092586

$vibrational spectrum (first 50 lines)
#  mode     symmetry     wave number   IR intensity    selection rules
#                         cm**(-1)        km/mol         IR     RAMAN
     1                        0.00         0.00000        -       -
     2                        0.00         0.00000        -       -
     3                        0.00         0.00000        -       -
     4                        0.00         0.00000        -       -
     5                        0.00         0.00000        -       -
     6                        0.00         0.00000        -       -
     7         a             12.68         0.82890       YES     YES
     8         a             17.43         0.62533       YES     YES
     9         a             22.99         0.03612       YES     YES
    10         a             30.63         0.34214       YES     YES
    11         a             39.08         1.09407       YES     YES
    12         a             46.75         0.08269       YES     YES
    13         a             61.01         0.46969       YES     YES
    14         a             66.38         0.08140       YES     YES
    15         a             71.33         0.09169       YES     YES
    16         a             77.62         0.14300       YES     YES
    17         a             78.65         0.00891       YES     YES
    18         a             89.45         1.19050       YES     YES
    19         a             94.72         0.11686       YES     YES
    20         a            103.79         0.06664       YES     YES
    21         a            107.10         0.42465       YES     YES
    22         a            112.84         0.61829       YES     YES
    23         a            117.66         1.05017       YES     YES
    24         a            123.00         0.30290       YES     YES
    25         a            129.48         0.85478       YES     YES
    26         a            143.87         0.76234       YES     YES
    27         a            159.48         8.69837       YES     YES
    28         a            165.01         0.76313       YES     YES
    29         a            195.81         2.86578       YES     YES
    30         a            200.60         0.64977       YES     YES
    31         a            226.01         0.21715       YES     YES
    32         a            230.11         0.40742       YES     YES
    33         a            231.64         0.09811       YES     YES
    34         a            243.75         0.55261       YES     YES
    35         a            249.97         1.45407       YES     YES
    36         a            264.54         0.29105       YES     YES
    37         a            281.69         2.90519       YES     YES
    38         a            314.40        25.45803       YES     YES
    39         a            320.70         2.13177       YES     YES
```

40	a	333.15	1.42734	YES	YES
41	a	354.38	0.64655	YES	YES
42	a	376.99	0.05101	YES	YES
43	a	399.57	0.05741	YES	YES
44	a	406.51	2.18504	YES	YES
45	a	409.46	5.85899	YES	YES
46	a	423.12	6.98149	YES	YES
47	a	435.27	2.96004	YES	YES
48	a	453.93	20.43287	YES	YES
49	a	459.44	2.91880	YES	YES
50	a	470.96	10.14126	YES	YES

$TS_{6\text{-}(7\text{-}NEt2)3a\text{-}7\text{-}a}$

SCF Energy (au) BP86/SV(P)	-2754.565552324
SCF Energy (au) PBE0/def2-TZVPP	-2754.085929394
SCF Energy (au) PBE0/def2-TZVPP COSMO Toluene	-2754.0961532279
Zero Point Energy (au)	0.4420199
Chemical potential (kJ mol^{-1})	982.74
Dispersion correction (au) PBE0/def2-TZVPP	-0.08580724

xyz coordinates
60

Mn	0.0032097	2.3693823	0.6465974
C	-0.0512985	2.1821156	2.4468499
C	1.7839901	2.1851067	0.6404213
C	0.2964266	4.1511757	0.7386305
O	0.5124854	5.2947185	0.8186564
O	2.9535905	2.1665268	0.6483551
O	-0.0858987	2.0496957	3.6015301
C	-2.8486397	1.5310028	0.7042440
C	-2.6967281	3.8640870	0.4556843
C	-4.0852272	4.0147416	0.4257136
C	-4.8824964	2.8606417	0.5394850
C	-4.2662565	1.6162886	0.6746838
C	-2.0785605	0.3017595	0.8261488
C	-0.6773760	0.3908529	0.6518073
C	0.0911930	-0.8338243	0.7817842
C	-0.5839258	-2.0477763	1.0980642
O	-1.9332322	-2.0849965	1.2830340
C	-2.7549628	-0.9631838	1.1314490
N	-2.0855027	2.6665993	0.5969274
C	-0.2888683	3.4632975	-2.5261774
C	0.5678332	4.5929325	-2.5745281
C	0.5314108	5.4693979	-3.6676385
C	-0.3720094	5.2552536	-4.7241015
C	-1.2384489	4.1479869	-4.6819454
C	-1.1991296	3.2590716	-3.5995330
C	-0.2372376	2.4974910	-1.4508889
C	-0.2197983	1.2438954	-1.1427495
H	-2.0406497	4.7423776	0.3650879
H	-4.5236173	5.0184236	0.3160920
H	-5.9825100	2.9323327	0.5208739
H	-4.8393699	0.6858979	0.7714791
H	1.2786198	4.7708392	-1.7537163
H	1.2151031	6.3347899	-3.6921204
H	-0.4033236	5.9526612	-5.5778829
H	-1.9523340	3.9724035	-5.5040597
H	-1.8765667	2.3899787	-3.5666671
H	0.0117153	0.2693551	-1.5828995
O	-3.9497417	-1.1567551	1.2858427
C	0.0753831	-3.2748695	1.2382917
C	1.4904792	-0.9630341	0.5711716
H	2.0726932	-0.0754835	0.2932801
C	2.1657205	-2.1722430	0.6824625
H	3.2455301	-2.1799887	0.4824602
C	1.4764700	-3.3807881	1.0307162
H	-0.5479923	-4.1350616	1.5133043
N	2.1404723	-4.5871793	1.1450633

```
C     1.4240792   -5.8365361    1.4099067
C     3.5938880   -4.6878913    0.9971788
H     2.0071602   -6.6602606    0.9424485
H     0.4507435   -5.8205509    0.8735355
C     1.2122558   -6.1311504    2.9028394
H     3.9323153   -5.5455040    1.6192607
H     4.0722575   -3.7910464    1.4456757
C     4.0630272   -4.8814488   -0.4533115
H     5.1711230   -4.9723787   -0.4956006
H     3.7613919   -4.0241465   -1.0931139
H     3.6271088   -5.8042691   -0.8956513
H     2.1844394   -6.1994615    3.4390365
H     0.6793422   -7.0986474    3.0357344
H     0.6102446   -5.3346049    3.3907776

$vibrational spectrum (first 50 lines)
#  mode     symmetry     wave number   IR intensity    selection rules
#                         cm**(-1)        km/mol          IR     RAMAN
     1        a             -182.64         0.00000       YES     YES
     2                         0.00         0.00000        -       -
     3                         0.00         0.00000        -       -
     4                         0.00         0.00000        -       -
     5                         0.00         0.00000        -       -
     6                         0.00         0.00000        -       -
     7                         0.00         0.00000        -       -
     8        a               15.59         0.61808       YES     YES
     9        a               20.25         0.26882       YES     YES
    10        a               25.87         0.20714       YES     YES
    11        a               29.31         0.75843       YES     YES
    12        a               44.09         1.35004       YES     YES
    13        a               49.54         0.01096       YES     YES
    14        a               64.41         0.14502       YES     YES
    15        a               69.15         0.07554       YES     YES
    16        a               76.02         0.10448       YES     YES
    17        a               76.84         0.08049       YES     YES
    18        a               85.51         0.09170       YES     YES
    19        a               90.80         0.39316       YES     YES
    20        a               99.47         0.33017       YES     YES
    21        a              104.05         0.26546       YES     YES
    22        a              107.59         0.41519       YES     YES
    23        a              118.61         0.49190       YES     YES
    24        a              126.61         1.38941       YES     YES
    25        a              130.83         0.05366       YES     YES
    26        a              135.98         0.39048       YES     YES
    27        a              169.81         0.54815       YES     YES
    28        a              183.71         0.05557       YES     YES
    29        a              192.40         0.84982       YES     YES
    30        a              203.11         0.13360       YES     YES
    31        a              217.17         0.06029       YES     YES
    32        a              227.53         0.37517       YES     YES
    33        a              235.44         0.03774       YES     YES
    34        a              242.84         0.58964       YES     YES
    35        a              248.08         0.08867       YES     YES
    36        a              264.99         0.40387       YES     YES
    37        a              280.94         4.61650       YES     YES
    38        a              320.36         0.46089       YES     YES
    39        a              327.25         0.71093       YES     YES
```

40	a	344.65	5.54541	YES	YES
41	a	355.85	0.94377	YES	YES
42	a	381.90	1.00508	YES	YES
43	a	397.59	0.76142	YES	YES
44	a	402.40	0.03221	YES	YES
45	a	408.07	3.59121	YES	YES
46	a	425.80	3.83547	YES	YES
47	a	435.42	2.97566	YES	YES
48	a	448.91	3.22656	YES	YES
49	a	455.31	11.89874	YES	YES
50	a	465.26	5.27173	YES	YES

6-(7-NEt$_2$)$_{a\text{-}7\text{-}a}$

SCF Energy (au)	BP86/SV(P)	-2754.601788044
SCF Energy (au)	PBE0/def2-TZVPP	-2754.128987614
SCF Energy (au)	PBE0/def2-TZVPP COSMO Toluene	-2754.1411491565
Zero Point Energy (au)		0.4441057
Chemical potential (kJ mol^{-1})		987.24
Dispersion correction (au)	PBE0/def2-TZVPP	-0.08590743

xyz coordinates
60

Mn	0.0206866	2.8331651	0.5673792
C	0.3387895	3.1165803	2.3502838
C	1.6789944	2.1883555	0.2840176
C	0.4935637	4.4866479	0.1711654
O	0.7636234	5.6021872	-0.0599069
O	2.7537033	1.7834109	0.0773938
O	0.5936648	3.3130993	3.4697118
C	-2.4677418	2.0022402	1.0188467
C	-2.7712201	4.2662392	0.4814284
C	-4.1584709	4.1573661	0.6485873
C	-4.7024363	2.9190048	1.0293538
C	-3.8450785	1.8228935	1.2193970
C	-1.4267866	0.9363279	1.1845814
C	-0.8019746	0.3011154	0.0755906
C	-0.0994775	-0.9393863	0.3098035
C	-0.0491215	-1.4796172	1.6246245
O	-0.6510562	-0.8464054	2.6748871
C	-1.4087427	0.3219587	2.5250848
H	-2.2844023	5.2119710	0.1938812
H	-4.7985752	5.0387320	0.4873039
H	-5.7886718	2.8071479	1.1788311
H	-4.2289204	0.8392521	1.5278556
N	-1.9603542	3.2087230	0.6772407
C	-0.4144953	2.9374958	-2.6235671
C	0.6747861	3.7687458	-2.9882734
C	0.7029580	4.4232665	-4.2285399
C	-0.3675603	4.2860366	-5.1302911
C	-1.4638526	3.4769871	-4.7821480
C	-1.4839088	2.8059379	-3.5502190
H	1.5200996	3.8825835	-2.2917637
H	1.5688636	5.0538729	-4.4911616
H	-0.3501778	4.8129752	-6.0988562
H	-2.3137285	3.3684632	-5.4772922
H	-2.3510377	2.1813359	-3.2779974
C	-0.4532147	2.2218562	-1.3383034
C	-0.8395260	0.9110710	-1.2608663
H	-1.0400407	0.2684364	-2.1424981
O	-2.0105289	0.7221528	3.5009007
C	0.6243229	-2.6660440	1.9238967
C	0.5999292	-1.6626007	-0.6939934
C	1.3012761	-3.3958034	0.9060515
H	0.6232512	-2.9820837	2.9744713
C	1.2665484	-2.8493290	-0.4223926
N	1.9667300	-4.5712689	1.1849208
H	1.7706977	-3.3643587	-1.2509094
H	0.6195376	-1.2622467	-1.7191545

```
C     2.7587203   -5.2757092    0.1726764
C     1.9339672   -5.1773172    2.5190141
H     0.9272663   -5.0245008    2.9631656
C     3.0277431   -4.6634513    3.4675098
H     2.0368622   -6.2767555    2.3856121
H     3.5793387   -5.8050331    0.7048215
H     3.2626127   -4.5332204   -0.4819853
C     1.9517348   -6.2783366   -0.6659922
H     1.1404260   -5.7719513   -1.2322527
H     1.4825704   -7.0541198   -0.0216603
H     2.6138531   -6.7949838   -1.3956285
H     2.9348229   -3.5696996    3.6402760
H     4.0415710   -4.8569759    3.0530250
H     2.9575872   -5.1764361    4.4520623

$vibrational spectrum (first 50 lines)
#  mode     symmetry     wave number   IR intensity    selection rules
#                         cm**(-1)        km/mol         IR     RAMAN
     1                        0.00         0.00000         -       -
     2                        0.00         0.00000         -       -
     3                        0.00         0.00000         -       -
     4                        0.00         0.00000         -       -
     5                        0.00         0.00000         -       -
     6                        0.00         0.00000         -       -
     7        a              17.64         1.80197        YES     YES
     8        a              25.56         0.03410        YES     YES
     9        a              29.19         0.13671        YES     YES
    10        a              38.41         0.14186        YES     YES
    11        a              46.12         0.33483        YES     YES
    12        a              51.75         0.10895        YES     YES
    13        a              55.18         0.88063        YES     YES
    14        a              62.70         1.58963        YES     YES
    15        a              69.86         0.45211        YES     YES
    16        a              73.79         0.08973        YES     YES
    17        a              80.43         0.13184        YES     YES
    18        a              83.38         0.19564        YES     YES
    19        a              89.50         0.85527        YES     YES
    20        a              96.09         0.33941        YES     YES
    21        a             100.27         0.95179        YES     YES
    22        a             106.40         0.30567        YES     YES
    23        a             110.92         0.30545        YES     YES
    24        a             121.26         1.13117        YES     YES
    25        a             146.47         0.37897        YES     YES
    26        a             153.68         0.29499        YES     YES
    27        a             155.65         0.23604        YES     YES
    28        a             180.74         0.38661        YES     YES
    29        a             187.37         2.24222        YES     YES
    30        a             210.32         2.40040        YES     YES
    31        a             226.70         0.16071        YES     YES
    32        a             232.49         0.28476        YES     YES
    33        a             238.18         1.63080        YES     YES
    34        a             247.97         1.64340        YES     YES
    35        a             255.94         0.70737        YES     YES
    36        a             274.63         5.98705        YES     YES
    37        a             289.80         0.97923        YES     YES
    38        a             308.15         0.55235        YES     YES
    39        a             327.14         0.19053        YES     YES
```

40	a	347.81	0.55490	YES	YES
41	a	366.09	4.58773	YES	YES
42	a	406.94	1.16124	YES	YES
43	a	415.00	1.99332	YES	YES
44	a	421.15	6.18192	YES	YES
45	a	426.14	1.09691	YES	YES
46	a	434.11	2.61537	YES	YES
47	a	452.67	4.22496	YES	YES
48	a	458.83	8.13179	YES	YES
49	a	460.62	2.65651	YES	YES
50	a	462.63	3.60616	YES	YES

TS$_{5a-(7-NEt2)-iso}$

SCF Energy (au) BP86/SV(P)	-2754.581131464
SCF Energy (au) PBE0/def2-TZVPP	-2754.100927423
SCF Energy (au) PBE0/def2-TZVPP COSMO Toluene	-2754.1119623745
Zero Point Energy (au)	0.4431386
Chemical potential (kJ mol^{-1})	988.47
Dispersion correction (au) PBE0/def2-TZVPP	-0.08586884

xyz coordinates

60

C	0.2457950	1.7203897	2.5527246
C	-1.9790501	2.7302766	2.8794580
C	-1.3986033	2.4760373	0.0595078
C	-1.7706005	0.0533680	2.7961003
C	-0.6839606	0.0473714	-0.0619016
C	0.4768483	-0.8488433	-0.0259256
O	-0.8915415	-2.7015021	-0.8220349
C	-2.0413418	-1.9206685	-0.9453158
C	-1.9605403	-0.5449276	-0.4480491
C	-3.1135027	0.2705632	-0.3775146
C	-4.3685383	0.0202108	-1.0217573
H	-4.4347297	-0.8688210	-1.6621418
C	-5.4442638	0.8612800	-0.8083564
H	-6.4080905	0.6633865	-1.3048441
C	-5.2953794	1.9953650	0.0451377
H	-6.1385221	2.6699383	0.2586584
C	-4.0647141	2.2504067	0.6057087
H	-3.8844276	3.1317317	1.2374416
Mn	-1.3390960	1.4977645	1.7691993
N	-2.9722540	1.4258951	0.4138417
O	1.2961833	1.8747690	3.0447601
O	-2.3626322	3.5438363	3.6310650
C	-0.4703194	1.4677235	-0.2309909
O	-2.0931920	-0.8566915	3.4464310
C	0.3140266	-2.2057512	-0.3932460
H	0.5440954	1.7956048	-0.5188434
C	-1.5523709	3.7805736	-0.5710107
C	-1.8324723	6.2970484	-1.8364940
C	-1.9848726	6.1881510	-0.4420067
C	-1.8515231	4.9423097	0.1863718
C	-1.4061126	3.9065587	-1.9790530
C	-1.5423926	5.1530316	-2.6036682
H	-1.9397182	7.2781932	-2.3281559
H	-2.2046215	7.0841457	0.1614301
H	-1.9536682	4.8599477	1.2797082
H	-1.1852735	3.0057674	-2.5753918
H	-1.4219688	5.2361160	-3.6964233
C	1.7883276	-0.4648383	0.3444180
C	1.3673830	-3.1279113	-0.3709132
O	-3.0159589	-2.4744835	-1.4284816
C	2.6800612	-2.7329843	0.0041359
H	1.1207350	-4.1569052	-0.6611975
H	1.9795291	0.5720202	0.6624359
C	2.8586866	-1.3560682	0.3547411
H	3.8448465	-0.9748217	0.6514458

```
N     3.7320609   -3.6339764    0.0285721
C     3.5406848   -5.0434714   -0.3174983
C     5.0857543   -3.2281603    0.4068253
H     5.5981125   -4.1227116    0.8237462
H     5.0364261   -2.5006185    1.2456464
C     5.9084194   -2.6528431   -0.7568716
H     4.5115550   -5.4290952   -0.6983526
H     2.8355654   -5.1236423   -1.1727365
C     3.0603292   -5.9094903    0.8577759
H     6.9304827   -2.3751541   -0.4163867
H     5.4256378   -1.7453770   -1.1799335
H     6.0106357   -3.3961169   -1.5780599
H     3.7890990   -5.8803845    1.6980821
H     2.9434863   -6.9693561    0.5421409
H     2.0814861   -5.5541666    1.2460464

$vibrational spectrum (first 50 lines)
#  mode     symmetry     wave number   IR intensity    selection rules
#                         cm**(-1)        km/mol         IR     RAMAN
     1        a           -320.41         0.00000        YES     YES
     2                       0.00         0.00000         -       -
     3                       0.00         0.00000         -       -
     4                       0.00         0.00000         -       -
     5                       0.00         0.00000         -       -
     6                       0.00         0.00000         -       -
     7                       0.00         0.00000         -       -
     8        a             20.98         1.44809        YES     YES
     9        a             27.25         0.27617        YES     YES
    10        a             37.88         0.07377        YES     YES
    11        a             42.49         0.18217        YES     YES
    12        a             44.37         0.36800        YES     YES
    13        a             52.84         0.83028        YES     YES
    14        a             57.10         0.69846        YES     YES
    15        a             60.09         0.37033        YES     YES
    16        a             68.78         0.03600        YES     YES
    17        a             74.96         0.48644        YES     YES
    18        a             79.93         0.27071        YES     YES
    19        a             86.74         0.01327        YES     YES
    20        a             94.46         0.39025        YES     YES
    21        a            105.84         0.87441        YES     YES
    22        a            108.40         1.48823        YES     YES
    23        a            110.37         0.75363        YES     YES
    24        a            118.43         0.47191        YES     YES
    25        a            125.15         0.31132        YES     YES
    26        a            146.71         0.19266        YES     YES
    27        a            155.91         0.87699        YES     YES
    28        a            181.21         0.23293        YES     YES
    29        a            201.69         1.37090        YES     YES
    30        a            212.86         0.78731        YES     YES
    31        a            227.93         0.11153        YES     YES
    32        a            232.03         0.59617        YES     YES
    33        a            241.73         3.10180        YES     YES
    34        a            244.71         0.16324        YES     YES
    35        a            258.82         4.95946        YES     YES
    36        a            272.39         0.78905        YES     YES
    37        a            282.67         8.12434        YES     YES
    38        a            319.14         1.01633        YES     YES
```

39	a	332.11	0.10184	YES	YES
40	a	353.83	1.17287	YES	YES
41	a	380.25	8.25302	YES	YES
42	a	391.90	1.21782	YES	YES
43	a	406.97	0.12842	YES	YES
44	a	411.02	8.56761	YES	YES
45	a	417.82	2.67863	YES	YES
46	a	426.67	2.19554	YES	YES
47	a	435.45	4.27103	YES	YES
48	a	458.14	1.46274	YES	YES
49	a	462.70	5.32959	YES	YES
50	a	470.39	7.39146	YES	YES

5a-(7-NEt$_2$)-iso

SCF Energy (au) BP86/SV(P)	-2754.595926462
SCF Energy (au) PBE0/def2-TZVPP	-2754.118701734
SCF Energy (au) PBE0/def2-TZVPP COSMO Toluene	-2754.1294016319
Zero Point Energy (au)	0.444623
Chemical potential (kJ mol^{-1})	991.74
Dispersion correction (au) PBE0/def2-TZVPP	-0.08670155

xyz coordinates
60

C	1.5790571	1.2716743	2.2736030
C	0.6682531	3.5059241	2.5925929
C	0.1872399	2.8419257	-0.2281536
C	-0.9746784	1.2993162	2.8916500
C	-0.4953694	0.4766424	-0.0238986
C	-0.1639796	-0.9509068	0.0257974
O	-2.4641770	-1.5339184	-0.5621944
C	-2.8849022	-0.2150573	-0.6662246
C	-1.9001156	0.8213061	-0.3260248
C	-2.2624921	2.1623160	-0.2430045
C	-3.5516031	2.7423947	-0.4745860
H	-4.3110448	2.0775823	-0.9074725
C	-3.8295594	4.0400558	-0.1010545
H	-4.8378342	4.4543020	-0.2609102
C	-2.8093719	4.8543297	0.5014713
H	-3.0244303	5.8759489	0.8486359
C	-1.5392288	4.3683677	0.6141908
H	-0.6916568	4.9632792	0.9787282
Mn	0.1244542	2.0772926	1.6623466
N	-1.2064903	3.0507031	0.2208740
O	2.5459707	0.7347771	2.6593393
O	1.0524515	4.4127184	3.2244240
C	0.4998498	1.4598086	-0.3328663
O	-1.6855906	0.8327661	3.6866776
C	-1.1744118	-1.9029724	-0.2450947
H	1.5020370	1.1724576	-0.6858741
C	0.9414652	3.9323513	-0.9010599
C	2.4182265	5.9345203	-2.2509074
C	3.0444605	5.1179103	-1.2915571
C	2.3111447	4.1324856	-0.6148208
C	0.3214416	4.7522510	-1.8729787
C	1.0549124	5.7499904	-2.5356714
H	2.9928251	6.7171915	-2.7729725
H	4.1116469	5.2610426	-1.0547142
H	2.7925055	3.5173095	0.1631376
H	-0.7407479	4.5977773	-2.1240252
H	0.5558065	6.3818413	-3.2889389
C	1.1206001	-1.4797412	0.2973803
C	-0.9399336	-3.2824268	-0.2337989
O	-4.0428054	-0.0412940	-1.0101825
C	0.3517800	-3.8041886	0.0494342
H	-1.8028158	-3.9266305	-0.4447415
H	1.9498193	-0.7937409	0.5286076
C	1.3840608	-2.8486032	0.3100418
H	2.4104653	-3.1748316	0.5252810
N	0.5958098	-5.1683296	0.0693392

```
C    -0.4405857   -6.1380090   -0.2844834
C     1.8955179   -5.7129007    0.4610500
H     1.7157336   -6.7117369    0.9170089
H     2.3246087   -5.0904388    1.2758930
C     2.8967395   -5.8464662   -0.6975234
H     0.0691248   -7.0343200   -0.7025677
H    -1.0533909   -5.7310190   -1.1178470
C    -1.3404597   -6.5546416    0.8899499
H     3.8550287   -6.2826582   -0.3371666
H     3.1165864   -4.8586181   -1.1571156
H     2.4978567   -6.5100938   -1.4962608
H    -0.7428370   -7.0129839    1.7086262
H    -2.0944832   -7.3020885    0.5568476
H    -1.8824839   -5.6819436    1.3137643

$vibrational spectrum (first 50 lines)
#  mode     symmetry     wave number   IR intensity    selection rules
#                         cm**(-1)        km/mol         IR     RAMAN
     1                        0.00         0.00000        -       -
     2                        0.00         0.00000        -       -
     3                        0.00         0.00000        -       -
     4                        0.00         0.00000        -       -
     5                        0.00         0.00000        -       -
     6                        0.00         0.00000        -       -
     7         a             21.09         1.39508       YES     YES
     8         a             27.64         0.25787       YES     YES
     9         a             39.06         0.18606       YES     YES
    10         a             40.81         0.06146       YES     YES
    11         a             43.74         0.25672       YES     YES
    12         a             49.74         1.58315       YES     YES
    13         a             53.90         1.25713       YES     YES
    14         a             61.21         0.96785       YES     YES
    15         a             67.54         0.32312       YES     YES
    16         a             77.55         1.12232       YES     YES
    17         a             83.51         0.10937       YES     YES
    18         a             85.10         0.63983       YES     YES
    19         a             91.75         0.79416       YES     YES
    20         a            102.16         7.37346       YES     YES
    21         a            106.65         0.65189       YES     YES
    22         a            112.27         0.29107       YES     YES
    23         a            117.24         0.71930       YES     YES
    24         a            133.94         0.59894       YES     YES
    25         a            139.42         1.79057       YES     YES
    26         a            151.32         3.52962       YES     YES
    27         a            183.42         1.26573       YES     YES
    28         a            199.68         0.80527       YES     YES
    29         a            206.07         1.99329       YES     YES
    30         a            227.27         0.25747       YES     YES
    31         a            231.89         0.34197       YES     YES
    32         a            241.72         0.13422       YES     YES
    33         a            248.03         0.41884       YES     YES
    34         a            251.77         3.74591       YES     YES
    35         a            271.18        10.71220       YES     YES
    36         a            273.78         1.72413       YES     YES
    37         a            321.02         1.29219       YES     YES
    38         a            332.05         0.51452       YES     YES
    39         a            362.53         0.30148       YES     YES
```

40	a	383.46	7.45796	YES	YES
41	a	399.35	2.64510	YES	YES
42	a	402.54	0.93610	YES	YES
43	a	409.60	10.62324	YES	YES
44	a	421.80	0.06396	YES	YES
45	a	429.10	0.42529	YES	YES
46	a	432.95	5.62189	YES	YES
47	a	450.39	3.29336	YES	YES
48	a	458.21	10.33911	YES	YES
49	a	464.09	3.55311	YES	YES
50	a	467.02	8.07193	YES	YES

5a-(7-NEt$_2$)

SCF Energy (au)	BP86/SV(P)	-2754.628187626
SCF Energy (au)	PBE0/def2-TZVPP	-2754.156258615
SCF Energy (au)	PBE0/def2-TZVPP COSMO Toluene	-2754.1687773889
Zero Point Energy (au)		0.4460519
Chemical potential (kJ mol^{-1})		997.64
Dispersion correction (au)	PBE0/def2-TZVPP	-0.08660747

xyz coordinates
60

C	0.1188442	2.8462428	2.1160181
C	-2.1841995	1.7834835	2.9131968
C	-1.8064795	2.2885556	0.0109060
C	0.1620480	0.4585652	2.8328994
C	-0.5503872	0.2047798	0.0173131
C	0.5397957	-0.7428142	-0.1895602
O	-0.6056172	-2.3218668	1.2740905
C	-1.8010007	-1.6071832	1.2971817
C	-1.7974141	-0.2781461	0.6523409
C	-3.0636945	0.2188661	0.1097743
C	-4.2550723	-0.5178436	-0.0580311
H	-4.2679386	-1.5550955	0.3001382
C	-5.3874726	0.0794172	-0.6211347
H	-6.3202272	-0.4983591	-0.7226194
C	-5.3221371	1.4151512	-1.0574865
H	-6.1843161	1.9233153	-1.5143382
C	-4.1297938	2.1143700	-0.8901023
H	-4.0017920	3.1616209	-1.1956976
Mn	-0.8851428	1.4228994	1.7246563
N	-3.0499664	1.5403800	-0.2910954
O	0.7825384	3.7764274	2.3654415
O	-3.0511985	2.0414769	3.6535619
C	-0.5777390	1.5740497	-0.3370147
O	0.8472558	-0.1500139	3.5569019
C	0.4682034	-1.9884581	0.4758050
H	0.2865149	2.0838426	-0.7877221
C	-1.9025019	3.7672828	-0.2206729
C	-2.0878641	6.5671813	-0.6518390
C	-1.3960279	5.7566536	-1.5661584
C	-1.3122989	4.3690625	-1.3560853
C	-2.5934165	4.5991029	0.6929105
C	-2.6908077	5.9815420	0.4765571
H	-2.1532862	7.6558287	-0.8137716
H	-0.9190124	6.2048179	-2.4536588
H	-0.7818017	3.7411599	-2.0910406
H	-3.0448466	4.1492686	1.5920042
H	-3.2264275	6.6109041	1.2064172
C	1.6891340	-0.5151307	-0.9787332
O	-2.7485523	-2.1384311	1.8450748
C	1.4826677	-2.9462231	0.3889753
H	1.7809593	0.4309206	-1.5364311
C	2.7127600	-1.4562755	-1.0883146
H	3.5668339	-1.2207494	-1.7374472
C	2.6468073	-2.7061885	-0.3935622
H	1.3417377	-3.8664492	0.9696449
N	3.6633843	-3.6439224	-0.4836530

```
C     4.9144889   -3.3597557   -1.1856375
C     3.5543070   -4.9577825    0.1508506
H     5.7192138   -3.9422423   -0.6845024
H     5.1864447   -2.2925825   -1.0346564
C     4.8872539   -3.7015517   -2.6840607
H     2.5064408   -5.3185796    0.0655608
C     4.0164272   -4.9902658    1.6164575
H     4.1622240   -5.6699720   -0.4502986
H     5.8721944   -3.4809917   -3.1528645
H     4.1093208   -3.1146534   -3.2187354
H     4.6665246   -4.7800143   -2.8441227
H     5.0812680   -4.6813814    1.7070678
H     3.9224567   -6.0184974    2.0312096
H     3.4103832   -4.3057478    2.2480922

$vibrational spectrum (first 50 lines)
#  mode     symmetry     wave number   IR intensity    selection rules
#                         cm**(-1)        km/mol          IR     RAMAN
```

3.2 Time-Dependent Density Functional Theory (TD-DFT) Calculations

Density functional theory (DFT) methods were used to probe the electronic structure of the of the five-membered manganacycle **6-(*7*-NEt$_2$)**. The geometries were optimised initially using a B3LYP functional and def2svp basis set, applied with Gimme's empirical dispersion correction (GD3), using a CH_2Cl_2 implicit solvent model (cpcm model, using Gaussian 16 Rev. A.03 Win64). Time-dependent DFT (TD-DFT) calculations were then conducted, using a cam-b3lyp13 functional and DGTZVP basis set (50 states; first 10 states shown below).

```
Excited State   1:      Singlet-A      2.9685 eV  417.67 nm  f=0.7762  <S**2>=0.000
     118 ->119         0.69794

 This state for optimization and/or second-order correction.
 Total Energy, E(TD-HF/TD-KS) =  -2560.42273891
 Copying the excited state density for this state as the 1-particle RhoCI density.

 Excited State   2:      Singlet-A      3.3472 eV  370.42 nm  f=0.0024  <S**2>=0.000
     117 ->120         0.11833
     118 ->120         0.68946

 Excited State   3:      Singlet-A      3.5962 eV  344.76 nm  f=0.0115  <S**2>=0.000
     115 ->119         0.17874
     115 ->120         0.54506
     115 ->126        -0.11845
     115 ->127         0.14012
     115 ->128        -0.14961
     115 ->133        -0.21618

 Excited State   4:      Singlet-A      3.6978 eV  335.29 nm  f=0.1048  <S**2>=0.000
     117 ->119         0.15301
     118 ->121         0.65695

 Excited State   5:      Singlet-A      3.7284 eV  332.54 nm  f=0.0103  <S**2>=0.000
     113 ->120         0.10880
     114 ->120        -0.24082
     115 ->119         0.33008
     115 ->120        -0.12392
     117 ->119         0.18423
     117 ->120         0.33957
     118 ->121        -0.18136

 Excited State   6:      Singlet-A      3.7725 eV  328.65 nm  f=0.0242  <S**2>=0.000
     114 ->119        -0.23870
```

114 ->120 0.11057
115 ->119 -0.22256
117 ->119 0.57020

Excited State 7: Singlet-A 3.8466 eV 322.32 nm f=0.0109 <S**2>=0.000
113 ->120 -0.10934
114 ->120 0.14671
115 ->119 0.51200
115 ->120 -0.12190
116 ->119 -0.11408
116 ->120 0.10859
117 ->120 -0.30772

Excited State 8: Singlet-A 3.9521 eV 313.72 nm f=0.0033 <S**2>=0.000
113 ->120 -0.22902
114 ->120 -0.29818
114 ->130 -0.11445
117 ->120 -0.17528
118 ->122 0.43948
118 ->123 -0.22334

Excited State 9: Singlet-A 3.9553 eV 313.47 nm f=0.0058 <S**2>=0.000
113 ->120 0.22253
114 ->119 0.11853
114 ->120 0.28969
114 ->130 0.13448
117 ->120 0.17393
118 ->122 0.45097
118 ->123 -0.19715

Excited State 10: Singlet-A 4.0074 eV 309.39 nm f=0.0087 <S**2>=0.000
115 ->119 0.10195
116 ->119 0.51010
117 ->119 0.10598
118 ->122 -0.12766
118 ->123 -0.36821
118 ->124 -0.14980
118 ->126 0.15169

4.0 Crystallographic Data

Crystallographic data of 8-(diethylamino)-5-oxo-12-phenyl-5*H*-chromeno[3,4-*a*]quinolizin-13-ium manganese tricarbonyl **5a-(*7*-NEt$_2$)**

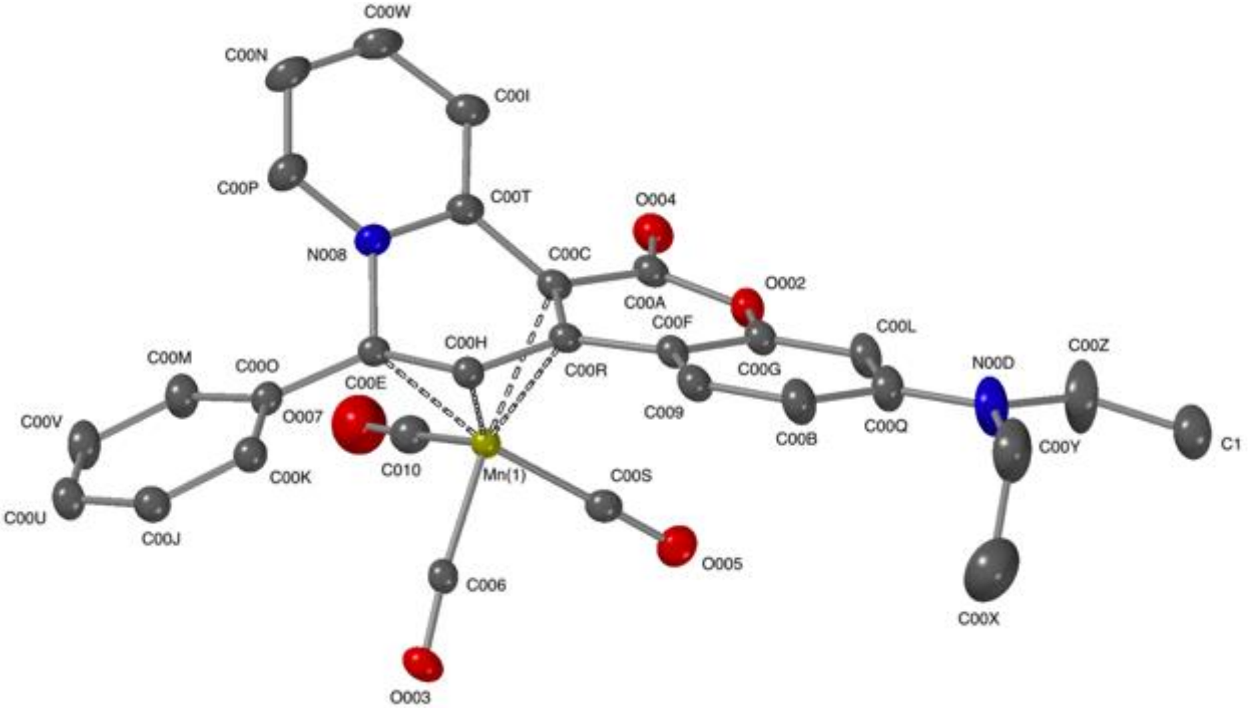

CCDC Nº	2204313
Identification code	ijsf2021
Empirical formula	$C_{29}H_{23}N_2O_5Mn$
Formula weight	534.43
Temperature/K	109.95(10)
Crystal system	monoclinic
Space group	C2/c
a/Å	15.4775(4)
b/Å	13.6687(4)
c/Å	23.6977(7)
α/°	90
β/°	105.380(3)
γ/°	90
Volume/Å^3	4833.9(2)

Z	8
ρ_{calc}g/cm^3	1.469
μ/mm^{-1}	0.591
F(000)	2208.0
Crystal size/mm^3	0.326 × 0.111 × 0.095
Radiation	Mo Kα (λ = 0.71073)
2Θ range for data collection/°	6.948 to 60.158
Index ranges	-21 ≤ h ≤ 12, -19 ≤ k ≤ 15, -22 ≤ l ≤ 33
Reflections collected	12141
Independent reflections	7079 [R_{int} = 0.0442, R_{sigma} = 0.0522]
Data/restraints/parameters	7079/1/421
Goodness-of-fit on F^2	1.075
Final R indexes [I>=2σ (I)]	R_1 = 0.0458, wR_2 = 0.1160
Final R indexes [all data]	R_1 = 0.0556, wR_2 = 0.1231
Largest diff. peak/hole / e Å^{-3}	0.53/-0.50

Crystallographic data of 8-(diethylamino)-5-oxo-11,12-diphenyl-5*H*-chromeno[3,4-*a*]quinolizin-13-ium manganese tricarbonyl **5b-(*7*-NEt$_2$)**

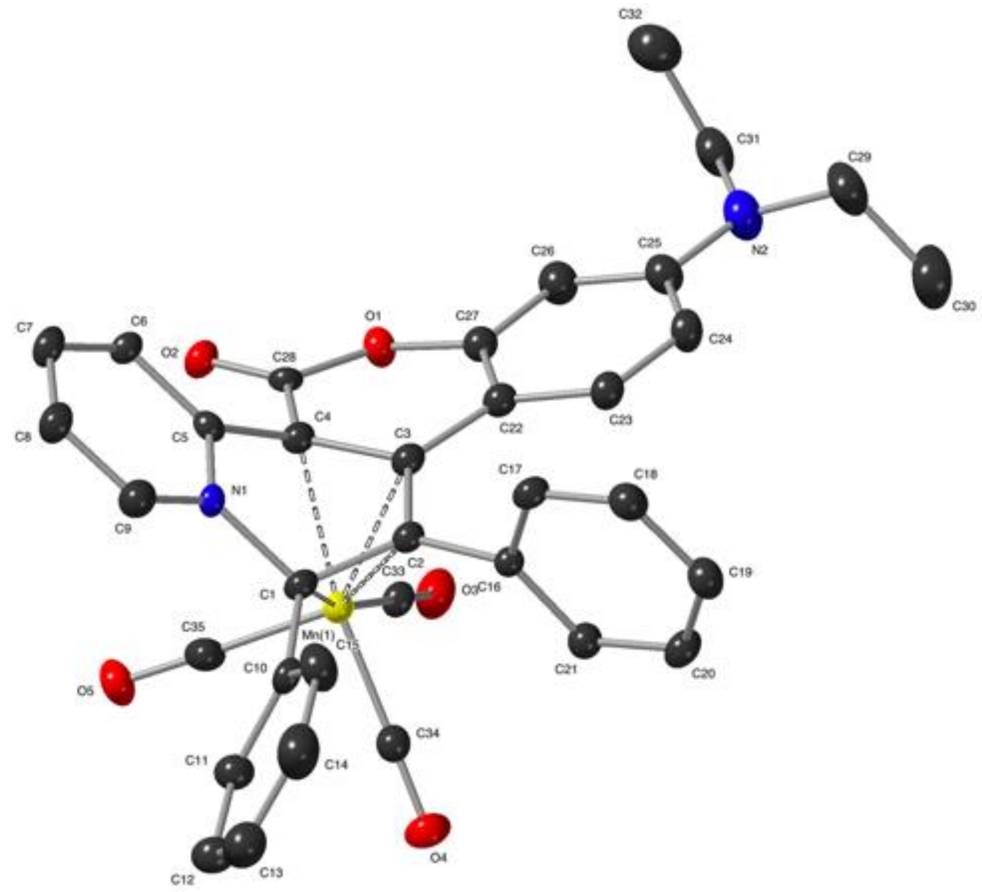

CCDC Nº	2204314
Identification code	ijsf2027
Empirical formula	$C_{35}H_{27}N_2O_5Mn$
Formula weight	610.52
Temperature/K	110.00(10)
Crystal system	triclinic
Space group	P-1
a/Å	10.3421(5)
b/Å	12.4148(6)
c/Å	23.0140(14)
α/°	80.183(5)
β/°	81.595(5)
γ/°	75.688(4)

Volume/Å^3	2804.5(3)
Z	4
ρ_{calc}g/cm^3	1.446
μ/mm^{-1}	4.226
F(000)	1264.0
Crystal size/mm^3	0.14 × 0.056 × 0.032
Radiation	Cu Kα (λ = 1.54184)
2Θ range for data collection/°	7.422 to 142.454
Index ranges	$-11 \leq h \leq 12$, $-15 \leq k \leq 13$, $-28 \leq l \leq 27$
Reflections collected	19738
Independent reflections	10634 [R_{int} = 0.0565, R_{sigma} = 0.0939]
Data/restraints/parameters	10634/0/779
Goodness-of-fit on F^2	1.007
Final R indexes [I>=2σ (I)]	R_1 = 0.0506, wR_2 = 0.0980
Final R indexes [all data]	R_1 = 0.0834, wR_2 = 0.1135
Largest diff. peak/hole / e Å^{-3}	0.60/-0.59

Crystallographic data of 8-(diethylamino)-11-methyl-5-oxo-12-phenyl-5*H*-chromeno[3,4-*a*]quinolizin-13-ium manganese tricarbonyl **5c-(*7*-NEt$_2$)**

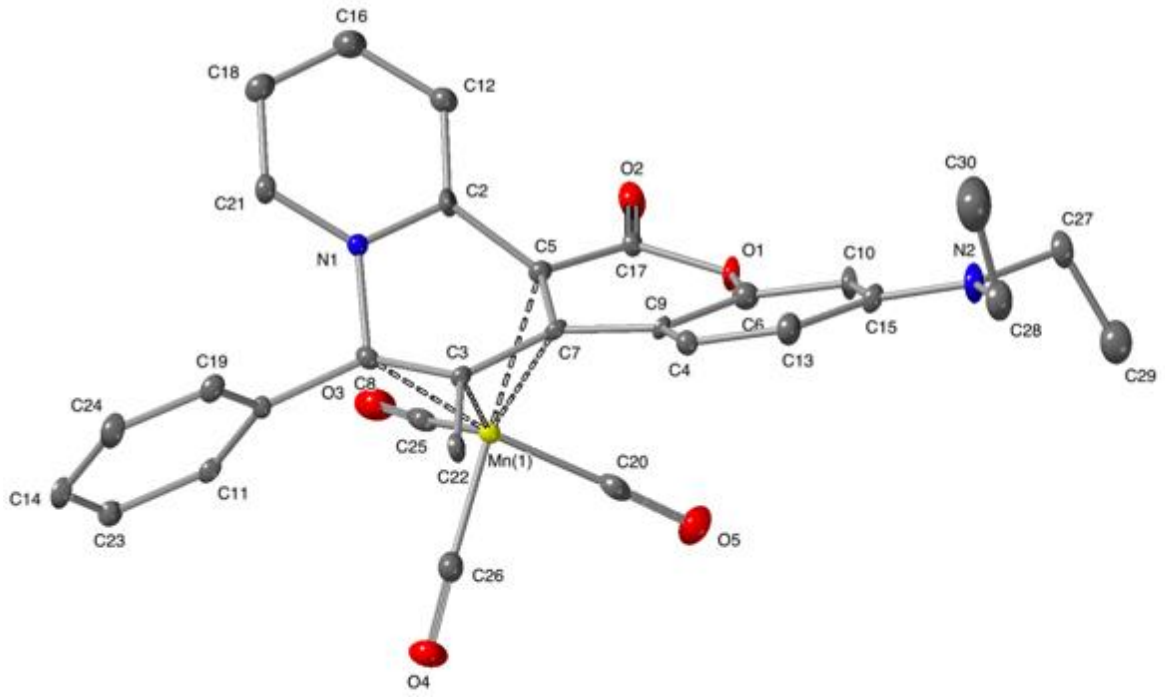

CCDC N°	2204316
Identification code	ijsf21015
Empirical formula	$C_{30.5}H_{26}ClMnN_2O_5$
Formula weight	590.92
Temperature/K	110.00(10)
Crystal system	triclinic
Space group	P-1
a/Å	7.9939(5)
b/Å	13.0868(5)
c/Å	13.4173(7)
α/°	86.730(4)
β/°	75.987(5)
γ/°	88.259(4)
Volume/Å^3	1359.44(12)
Z	2

ρ_{calc}g/cm^3	1.444
μ/mm^{-1}	5.218
F(000)	610.0
Crystal size/mm^3	0.229 × 0.1 × 0.058
Radiation	Cu Kα (λ = 1.54184)
2Θ range for data collection/°	9.348 to 134.158
Index ranges	-9 ≤ h ≤ 9, -11 ≤ k ≤ 15, -12 ≤ l ≤ 16
Reflections collected	8962
Independent reflections	4847 [R_{int} = 0.0243, R_{sigma} = 0.0366]
Data/restraints/parameters	4847/0/479
Goodness-of-fit on F^2	1.034
Final R indexes [I>=2σ (I)]	R_1 = 0.0304, wR_2 = 0.0733
Final R indexes [all data]	R_1 = 0.0344, wR_2 = 0.0758
Largest diff. peak/hole / e Å^{-3}	0.27/-0.30

Crystallographic data of 8-(diethylamino)-5-oxo-12-(cyclopropyl)-5*H*-chromeno[3,4-*a*]quinolizin-13-ium manganese tricarbonyl **5d-(*7*-NEt$_2$)**

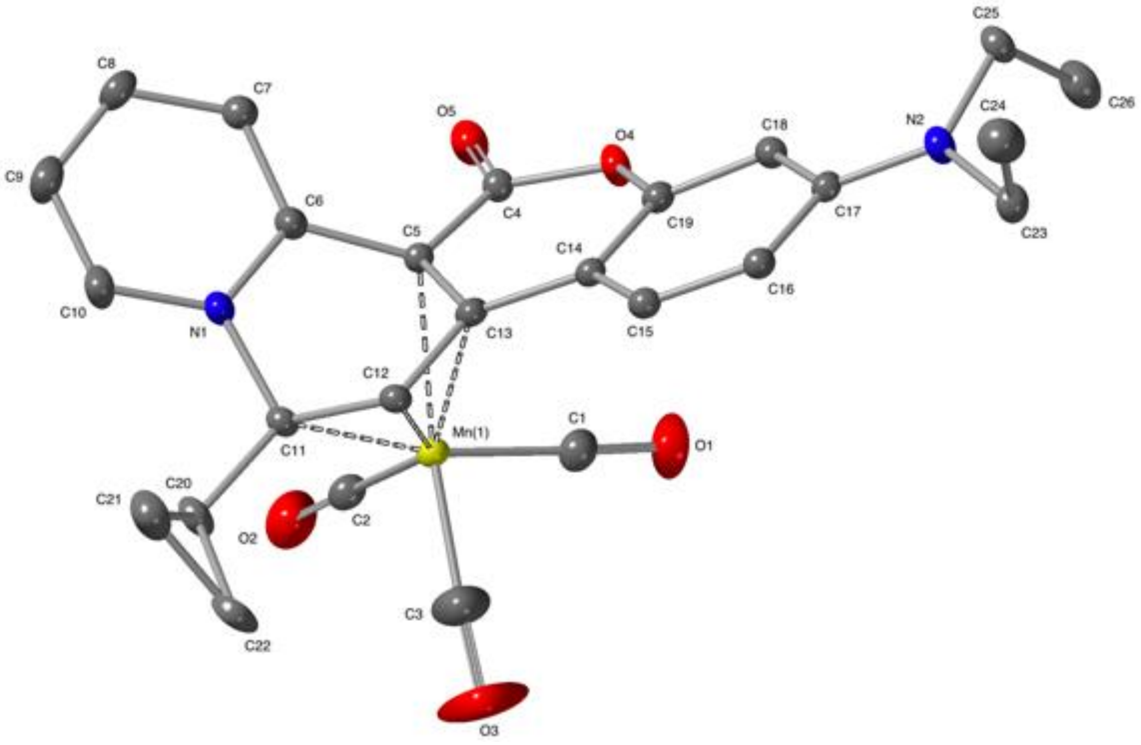

CCDC Nº	2204317
Identification code	ijsf21016
Empirical formula	$C_{26}H_{23}N_2O_5Mn$
Formula weight	498.40
Temperature/K	110.00(10)
Crystal system	orthorhombic
Space group	$P2_12_12_1$
a/Å	9.21335(12)
b/Å	14.6667(3)
c/Å	16.8114(3)
α/°	90
β/°	90
γ/°	90
Volume/Å^3	2271.72(6)
Z	4

ρ_{calc}g/cm^3	1.457
μ/mm^{-1}	5.074
F(000)	1032.0
Crystal size/mm^3	0.209 × 0.144 × 0.036
Radiation	Cu Kα (λ = 1.54184)
2Θ range for data collection/°	8 to 134.13
Index ranges	-8 ≤ h ≤ 11, -15 ≤ k ≤ 17, -20 ≤ l ≤ 20
Reflections collected	16733
Independent reflections	4054 [R_{int} = 0.0288, R_{sigma} = 0.0275]
Data/restraints/parameters	4054/0/398
Goodness-of-fit on F^2	1.051
Final R indexes [I>=2σ (I)]	R_1 = 0.0245, wR_2 = 0.0575
Final R indexes [all data]	R_1 = 0.0271, wR_2 = 0.0589
Largest diff. peak/hole / e Å^{-3}	0.19/-0.28
Flack parameter	-0.013(2)

Crystallographic data of 8-(diethylamino)-11-5-oxo-12-(*p*-tolyl)-5*H*-chromeno[3,4-*a*]quinolizin-13-ium manganese tricarbonyl **5h-(*7*-NEt$_2$)**

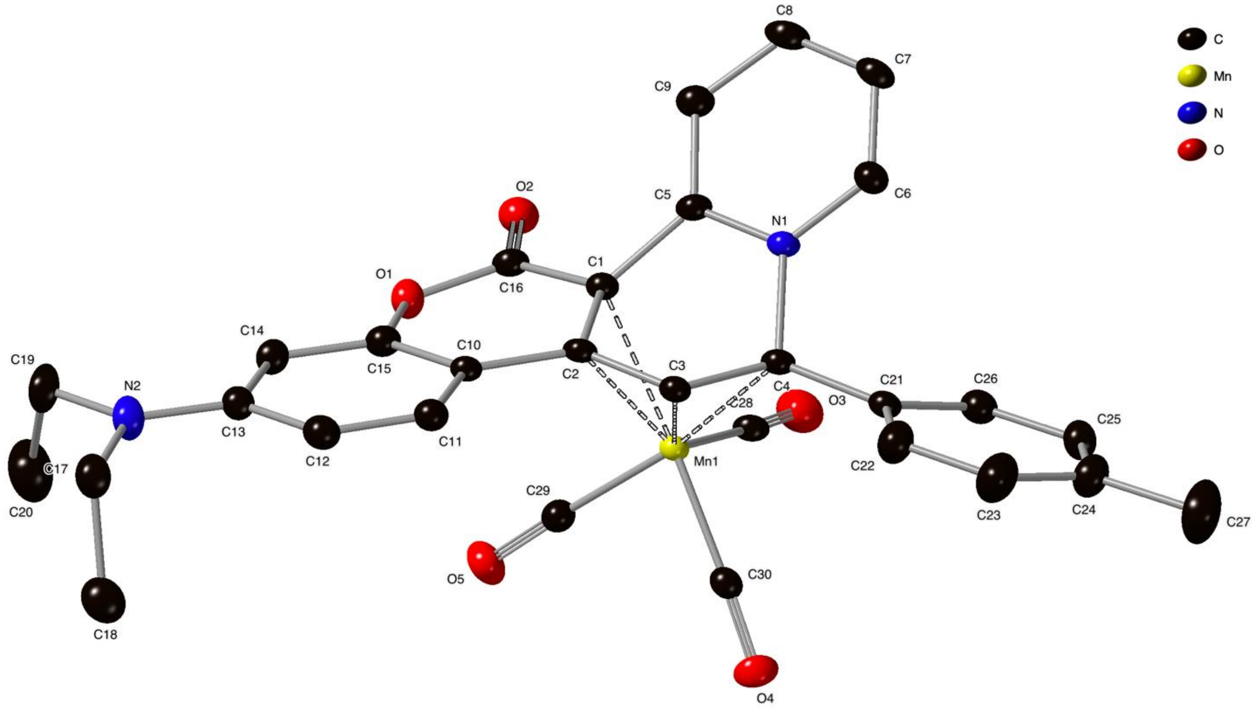

CCDC No.	2204324
Identification code	ijsf21057
Empirical formula	C30H25MnN2O5
Formula weight	548.46
Temperature/K	110.00(10)
Crystal system	monoclinic
Space group	P21/c
a/Å	12.4117(2)
b/Å	13.9516(2)
c/Å	15.6498(3)
α/°	90
β/°	108.779(2)
γ/°	90
Volume/Å3	2565.71(8)
Z	4
ρcalcg/cm3	1.420
μ/mm 1	4.549

F(000) 1136.0

Crystal size/mm3 ? × ? × ?

Radiation Cu Kα (λ = 1.54184)

2Θ range for data collection/° 7.524 to 142.228

Index ranges -14 ≤ h ≤ 15, -10 ≤ k ≤ 16, -18 ≤ l ≤ 19

Reflections collected 9525

Independent reflections 4838 [Rint = 0.0238, Rsigma = 0.0398]

Data/restraints/parameters 4838/0/346

Goodness-of-fit on F2 1.059

Final R indexes [I>=2σ (I)] R1 = 0.0371, wR2 = 0.0858

Final R indexes [all data] R1 = 0.0470, wR2 = 0.0900

Largest diff. peak/hole / e Å-3 0.33/-0.32

Data collected and refined by Theo F. N. Tanner

Crystallographic data of 3-(pyridin-2-yl)coumarin)-tetracarbonyl manganese **6-(H)**

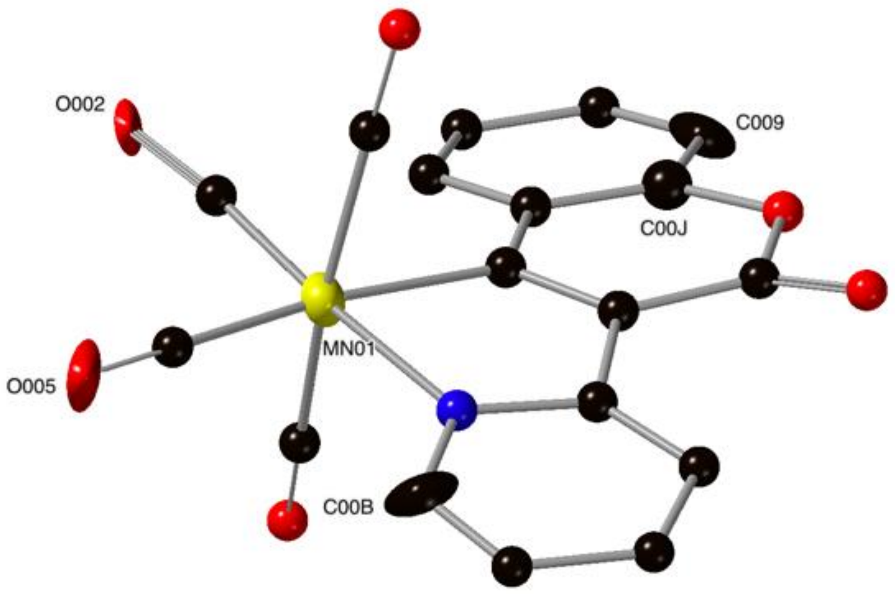

CCDC Nº	2204319
Identification code	ijsf21018
Empirical formula	$C_{18}H_8MnNO_6$
Formula weight	389.19
Temperature/K	109.95(10)
Crystal system	monoclinic
Space group	$P2_1/n$
a/Å	9.0420(3)
b/Å	18.3906(6)
c/Å	9.4848(3)
α/°	90
β/°	102.973(3)
γ/°	90
Volume/Å^3	1536.95(9)
Z	4
ρ_{calc}g/cm^3	1.682
μ/mm^{-1}	7.345
F(000)	784.0

Crystal size/mm^3	0.077 × 0.046 × 0.03
Radiation	Cu Kα (λ = 1.54184)
2Θ range for data collection/°	9.618 to 134.156
Index ranges	$-10 \leq h \leq 6$, $-21 \leq k \leq 21$, $-11 \leq l \leq 11$
Reflections collected	5487
Independent reflections	2735 [R_{int} = 0.0500, R_{sigma} = 0.0819]
Data/restraints/parameters	2735/0/235
Goodness-of-fit on F^2	1.009
Final R indexes [I>=2σ (I)]	R_1 = 0.0555, wR_2 = 0.1379
Final R indexes [all data]	R_1 = 0.0863, wR_2 = 0.1618
Largest diff. peak/hole / e $Å^{-3}$	1.69/-0.70

Refinement Special Details

Noisy data due to small size. Evidence of twinning but no twin could be determined neither non-merohedral or pseudomerohedral)

Crystallographic data of 12-phenyl-5*H*-chromeno[3,4-*a*]quinolizin-13-ium manganese tricarbonyl **5a-(H)**

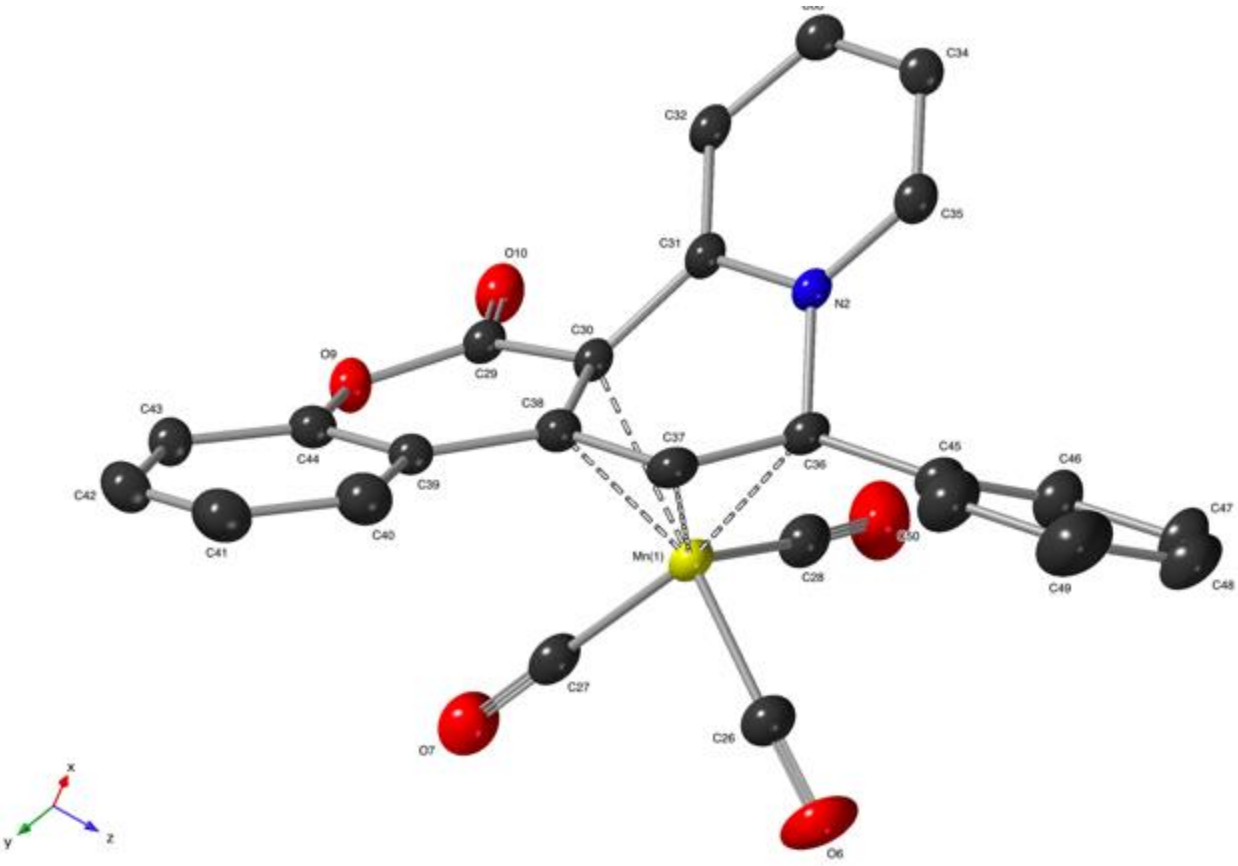

CCDC Nº	2204318
Identification code	ijsf21017
Empirical formula	$C_{26.62}H_{18.25}MnNO_{5.42}$
Formula weight	493.82
Temperature/K	109.95(10)
Crystal system	triclinic
Space group	P-1
a/Å	7.6171(2)
b/Å	17.5328(5)
c/Å	18.4382(4)
α/°	110.042(2)
β/°	94.325(2)

γ/°	100.504(2)
Volume/Å^3	2249.03(11)
Z	4
ρ_{calc}g/cm^3	1.458
μ/mm^{-1}	5.127
F(000)	1013.0
Crystal size/mm^3	0.313 × 0.049 × 0.036
Radiation	Cu Kα (λ = 1.54184)
2Θ range for data collection/°	8.812 to 134.156
Index ranges	$-9 \le h \le 8$, $-19 \le k \le 20$, $-22 \le l \le 19$
Reflections collected	15311
Independent reflections	8009 [R_{int} = 0.0275, R_{sigma} = 0.0390]
Data/restraints/parameters	8009/62/752
Goodness-of-fit on F^2	1.037
Final R indexes [I>=2σ (I)]	R_1 = 0.0403, wR_2 = 0.1032
Final R indexes [all data]	R_1 = 0.0481, wR_2 = 0.1093
Largest diff. peak/hole / e Å^{-3}	0.90/-0.45

Refinement Special Details

The crystal contained a solvent void partially occupied by a disordered diethyl ether. The ether was modelled in two positions with refined occupancies of 0.517(8) and 0.333(7). For this disordered ether, bond lengths and distances were restrained as follows:

C-C 1.51 angstroms

C-O 1.43 angstroms

C(C)O 2.14 angstroms

C(O)C 2.39 angstroms

The ADP of the atoms in the ethers were restrained to be approximately isotropic and the ADP of equivalent pairs of atoms were constrained to be equal (C51a & C51B, C52a & C52b etc.)

Crystallographic data of 7-(methoxymethoxy)-3-(pyridin-2-yl)-*2H*-chromen-2-one-tetracarbonyl manganese **6-(*7*-OMOM)**

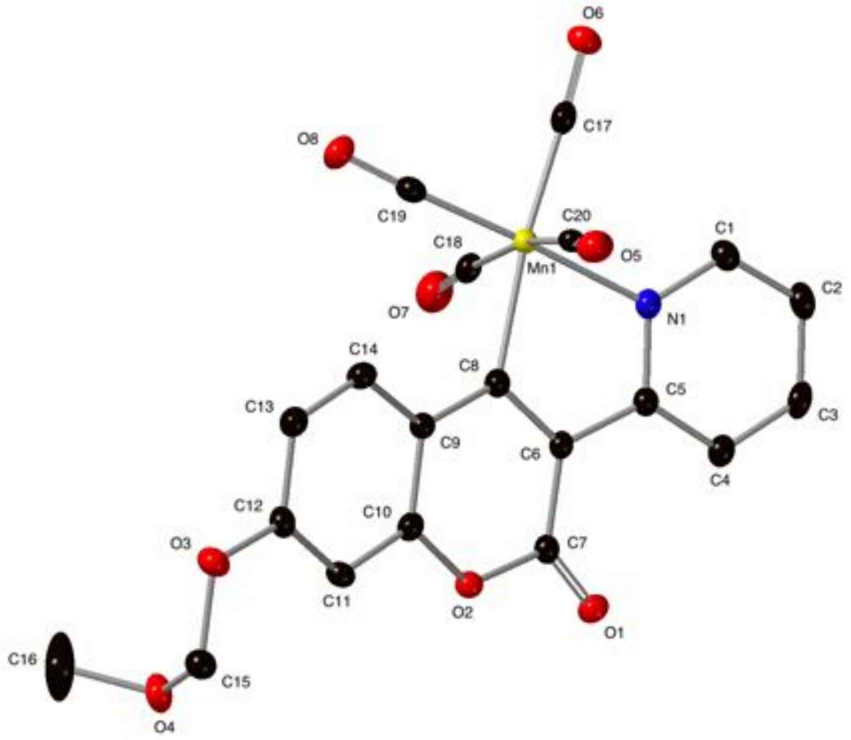

CCDC N°	2204320
Identification code	ijsf21032
Empirical formula	$C_{20}H_{12}MnNO_8$
Formula weight	449.25
Temperature/K	110.00(10)
Crystal system	monoclinic
Space group	$P2_1/n$
a/Å	8.5008(3)
b/Å	18.0287(8)
c/Å	12.1293(6)
α/°	90
β/°	101.633(4)
γ/°	90
Volume/Å^3	1820.73(14)

Z	4
ρ_{calc}g/cm^3	1.639
μ/mm^{-1}	6.373
F(000)	912.0
Crystal size/mm^3	0.143 × 0.04 × 0.03
Radiation	Cu Kα (λ = 1.54184)
2Θ range for data collection/°	8.914 to 142.09
Index ranges	-10 ≤ h ≤ 7, -21 ≤ k ≤ 21, -14 ≤ l ≤ 14
Reflections collected	6953
Independent reflections	3457 [R_{int} = 0.0263, R_{sigma} = 0.0397]
Data/restraints/parameters	3457/0/272
Goodness-of-fit on F^2	1.036
Final R indexes [I>=2σ (I)]	R_1 = 0.0324, wR_2 = 0.0771
Final R indexes [all data]	R_1 = 0.0403, wR_2 = 0.0826
Largest diff. peak/hole / e Å^{-3}	0.35/-0.36

Data collected and refined by Theo F. N. Tanner

Crystallographic data of 8-methyl-5-oxo-12-(2-pyridyl)-5*H*-chromeno[3,4-*a*]quinolizin-13-ium manganese tricarbonyl **5a-(*7*-Me)**

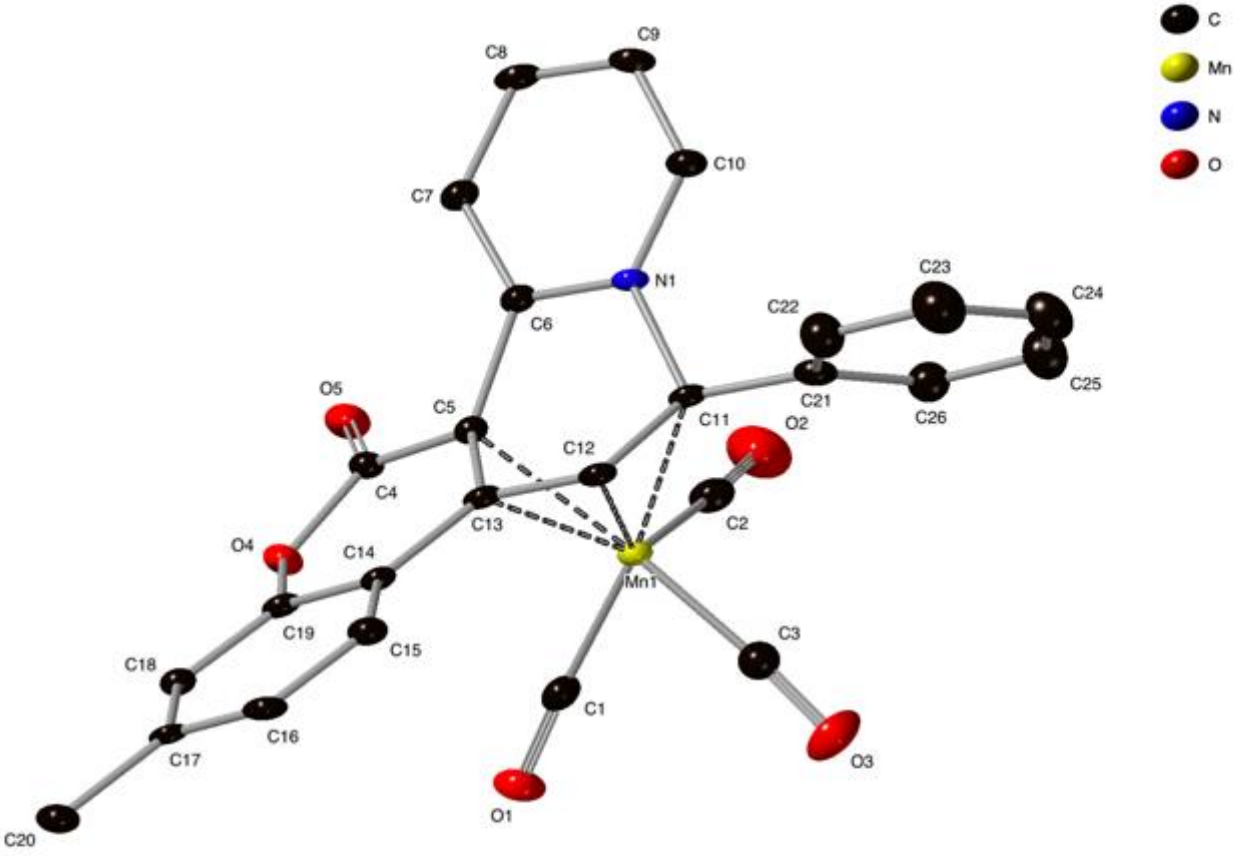

CCDC Nº	2204321
Identification code	ijsf21036
Empirical formula	$C_{26}H_{16}NO_5Mn$
Formula weight	477.34
Temperature/K	110.00(10)
Crystal system	triclinic
Space group	P-1
a/Å	7.3980(3)
b/Å	9.3678(4)
c/Å	16.7224(15)
α/°	82.119(6)

β/°	86.250(5)
γ/°	67.672(4)
Volume/Å^3	1061.78(12)
Z	2
ρ_{calc}g/cm^3	1.493
μ/mm^{-1}	5.395
F(000)	488.0
Crystal size/mm^3	0.134 × 0.064 × 0.018
Radiation	Cu Kα (λ = 1.54184)
2Θ range for data collection/°	10.284 to 134.146
Index ranges	-7 ≤ h ≤ 8, -11 ≤ k ≤ 10, -19 ≤ l ≤ 19
Reflections collected	6253
Independent reflections	3762 [R_{int} = 0.0291, R_{sigma} = 0.0458]
Data/restraints/parameters	3762/0/299
Goodness-of-fit on F^2	1.064
Final R indexes [I>=2σ (I)]	R_1 = 0.0535, wR_2 = 0.1373
Final R indexes [all data]	R_1 = 0.0597, wR_2 = 0.1420
Largest diff. peak/hole / e Å^{-3}	1.63/-0.44

Data collected, solved and refined by Adrian C Whitwood

Refinement Special Details

The relatively large residual density peaks are evidence of twinning, but a suitable way of modelling this twinning could not be found.

Crystallographic data of 8-(nitro)-5-oxo-12-(2-pyridyl)-5*H*-chromeno[3,4-*a*]quinolizin-13-ium manganese tricarbonyl **5a-(*7*-NO$_2$)**

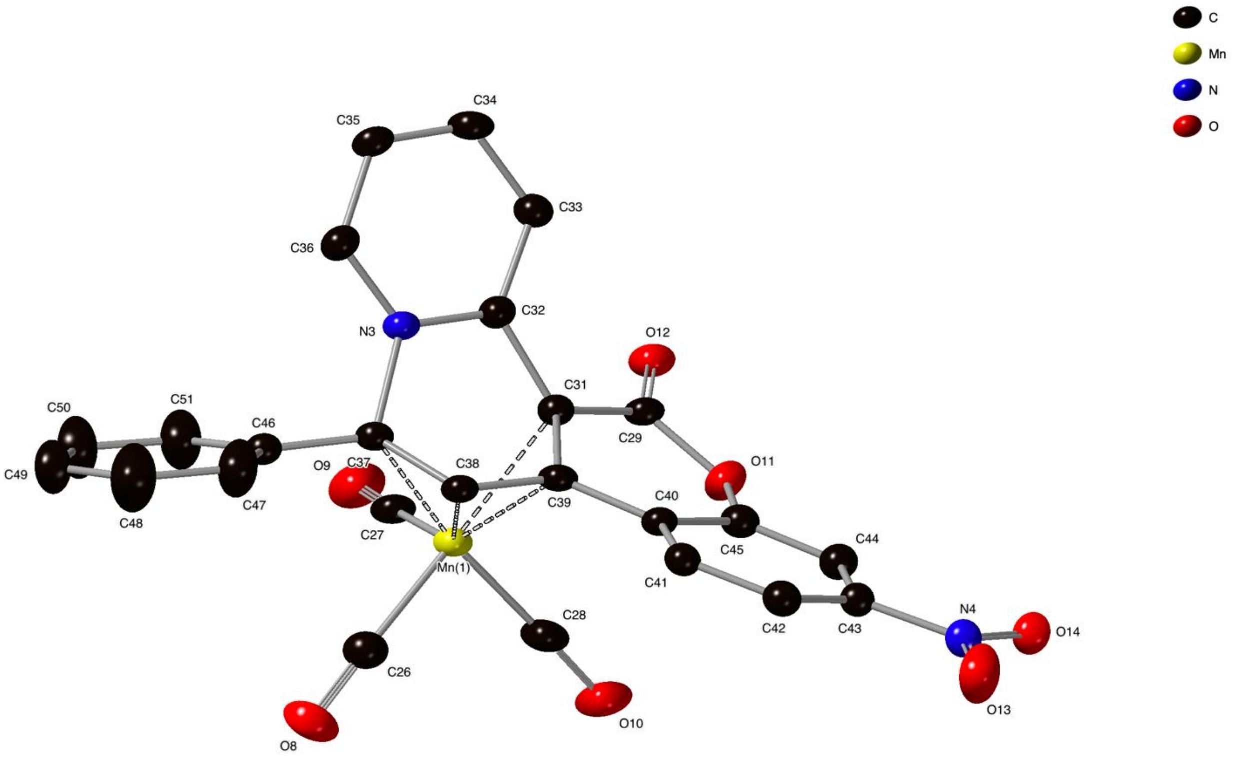

CCDC N^o	2204322
Identification code	ijsf21039
Empirical formula	$C_{25}H_{13}MnN_2O_7$
Formula weight	508.31
Temperature/K	109.95(10)
Crystal system	orthorhombic
Space group	Pbca
a/Å	13.91355(18)
b/Å	14.19775(18)
c/Å	49.2454(6)
α/°	90
β/°	90
γ/°	90

Volume/Å^3	9728.0(2)
Z	16
ρ_{calc}g/cm^3	1.388
μ/mm^{-1}	4.824
F(000)	4128.0
Crystal size/mm^3	0.163 × 0.118 × 0.03
Radiation	Cu Kα (λ = 1.54184)
2Θ range for data collection/°	7.18 to 134.154
Index ranges	$-16 \le h \le 12$, $-16 \le k \le 15$, $-54 \le l \le 58$
Reflections collected	35183
Independent reflections	8681 [R_{int} = 0.0376, R_{sigma} = 0.0325]
Data/restraints/parameters	8681/0/632
Goodness-of-fit on F^2	1.070
Final R indexes [I>=2σ (I)]	R_1 = 0.0425, wR_2 = 0.0954
Final R indexes [all data]	R_1 = 0.0487, wR_2 = 0.0981
Largest diff. peak/hole / e Å^{-3}	0.30/-0.34

Data collected, solved and refined by Adrian C Whitwood

Refinement Special Details
The crystal contained a solvent channel for which a suitable discrete model could not be determined. Therefore, the solvent was modelled using a mask. This predicted a void containing 54 electrons per asymmetric unit which is equivalent to one hexane.

Crystallographic data of 8-(diethylammonio)-5-oxo-12-phenyl-5*H*-chromeno[3,4-*a*]quinolizin-13-ium tetrachloromanganate **8a**

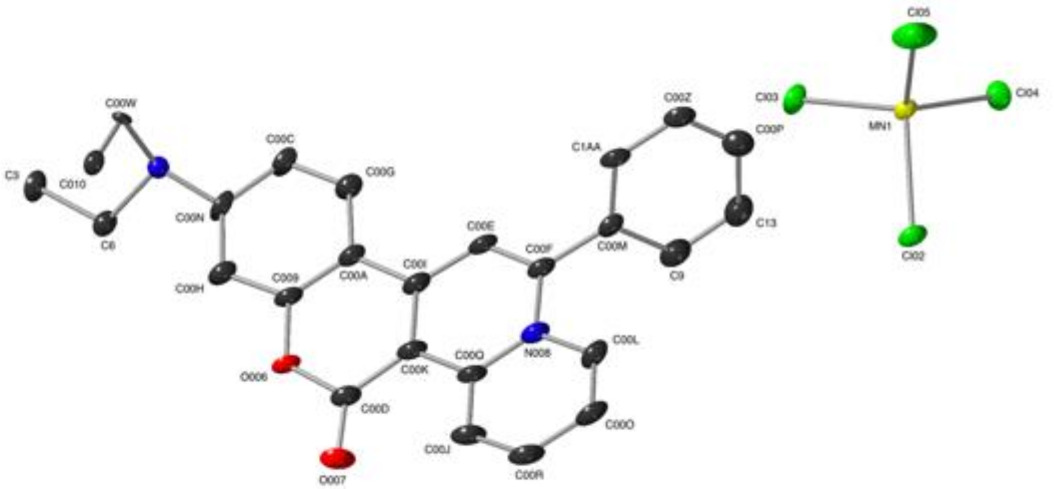

CCDC Nº	2204315
Identification code	ijsf21003
Empirical formula	$C_{28}H_{27}Cl_4MnN_3O_2$
Formula weight	634.26
Temperature/K	110.00(14)
Crystal system	orthorhombic
Space group	$Pna2_1$
a/Å	18.17238(17)
b/Å	15.66339(15)
c/Å	10.28439(9)
α/°	90
β/°	90
γ/°	90
Volume/Å^3	2927.36(5)
Z	4
ρ_{calc}g/cm^3	1.439
μ/mm^{-1}	7.272
F(000)	1300.0
Crystal size/mm^3	0.19 × 0.11 × 0.08
Radiation	Cu Kα (λ = 1.54184)

2Θ range for data collection/°	7.452 to 134.128
Index ranges	$-20 \leq h \leq 21$, $-18 \leq k \leq 18$, $-12 \leq l \leq 10$
Reflections collected	14158
Independent reflections	4363 [R_{int} = 0.0263, R_{sigma} = 0.0268]
Data/restraints/parameters	4363/2/443
Goodness-of-fit on F^2	1.063
Final R indexes [I>=2σ (I)]	R_1 = 0.0246, wR_2 = 0.0576
Final R indexes [all data]	R_1 = 0.0264, wR_2 = 0.0586
Largest diff. peak/hole / e Å^{-3}	0.29/-0.26
Flack parameter	-0.007(3)

Refinement Special Details

The crystal exhibited disorder with the diethylamino and phenyl groups and the acetonitrile modelled in two positions. Refinement of the occupancies of each component lead to similar closely similar ratios so a single variable was used so the ratio of the two components was 0.528:0.472(6). For the diethylamino group, the N-C(Ar) bond lengths were restrained to be equal and the ADP of N2 & N2a constrained to be equal.

Crystallographic data of 5-oxo-12-phenyl-5*H*-chromeno[3,4-*a*]quinolizin-13-ium tetrafluoroborate **10a**

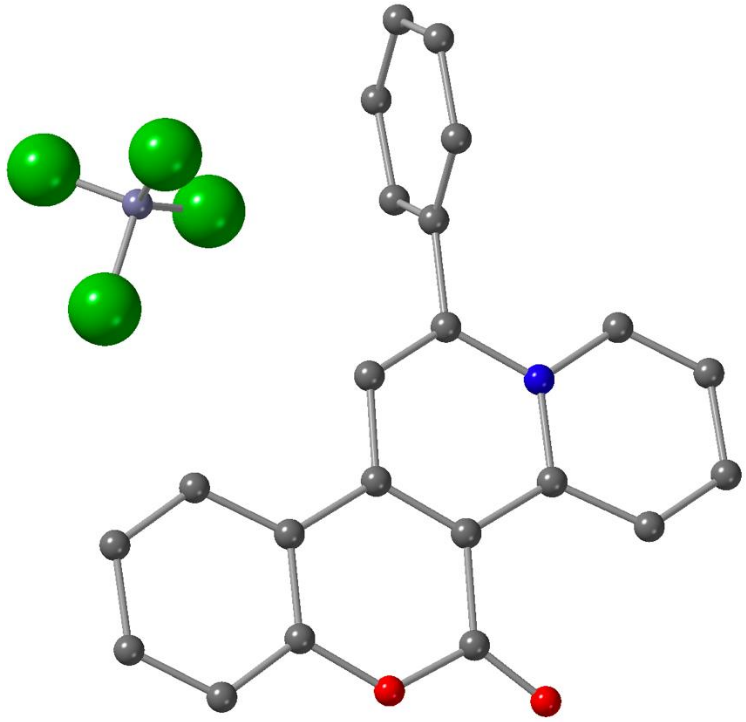

CCDC Nº	2204323
Identification code	ijsf21040
Empirical formula	$C_{22}H_{14}BF_4NO_2$
Formula weight	411.15
Temperature/K	109.95(10)
Crystal system	monoclinic
Space group	$P2_1/n$
a/Å	9.0971(2)
b/Å	9.9864(3)
c/Å	19.3198(5)
α/°	90
β/°	90.573(2)
γ/°	90
Volume/Å^3	1755.06(8)

Z	4
ρ_{calc}g/cm^3	1.556
μ/mm^{-1}	1.094
F(000)	840.0
Crystal size/mm^3	0.25 × 0.07 × 0.02
Radiation	Cu Kα (λ = 1.54184)
2Θ range for data collection/°	9.156 to 134.11
Index ranges	-10 ≤ h ≤ 8, -11 ≤ k ≤ 11, -14 ≤ l ≤ 23
Reflections collected	6211
Independent reflections	3114 [R_{int} = 0.0200, R_{sigma} = 0.0268]
Data/restraints/parameters	3114/6/346
Goodness-of-fit on F^2	1.052
Final R indexes [I>=2σ (I)]	R_1 = 0.0368, wR_2 = 0.0938
Final R indexes [all data]	R_1 = 0.0481, wR_2 = 0.1023
Largest diff. peak/hole / e Å^{-3}	0.18/-0.21

Data collected, solved and refined by Adrian C Whitwood

Refinement Special Details

The structure was disordered at two sites.

The phenyl ring was disordered by rotation about the C(ipso)-C(para) axis and modelled in two positions with refined occupancies of 0.556:0.444(6). The ADP of pairs of disordered atoms were constrained to be equal (C18A & C18B, C19A & C19B, C21A &C21B, C22A & C22B).

The tetrafluoroborate anions was also disordered by rotation about the F1-F2 axis and modelled in two positions in a refined ratio of 0.556:0.444(11). The ADP of B1A & B1B were constrained to be equal. The disordered B-F bonds were restrained to have equal length.

Crystallographic data of benzyl rhenium(I) pentacarbonyl

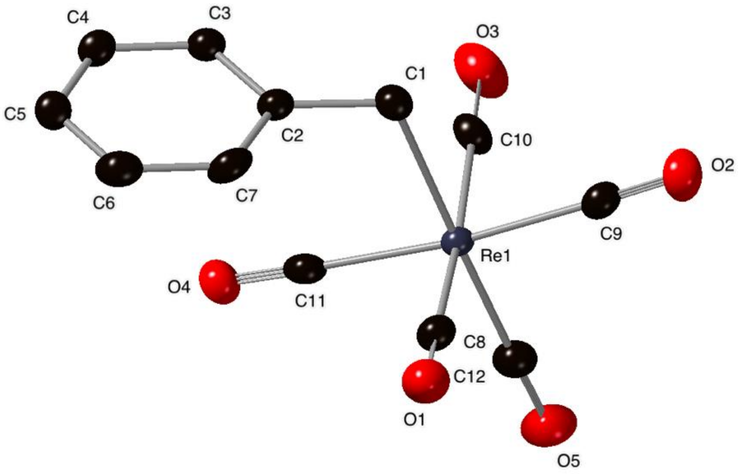

CCDC N°	2204325
Identification code	ijsf21061
Empirical formula	$C_{12}H_7O_5Re$
Formula weight	417.38
Temperature/K	110.00(10)
Crystal system	monoclinic
Space group	$P2_1/n$
a/Å	7.00910(9)
b/Å	6.63255(8)
c/Å	27.6538(4)
α/°	90
β/°	96.9500(12)
γ/°	90
Volume/Å^3	1276.13(3)
Z	4
ρ_{calc}g/cm^3	2.172
μ/mm^{-1}	9.530
F(000)	776.0
Crystal size/mm^3	0.22 × 0.179 × 0.053

Radiation	Mo Kα (λ = 0.71073)
2Θ range for data collection/°	6.824 to 52.742
Index ranges	$-8 \leq h \leq 8$, $-8 \leq k \leq 8$, $-34 \leq l \leq 34$
Reflections collected	19536
Independent reflections	2606 [R_{int} = 0.0252, R_{sigma} = 0.0133]
Data/restraints/parameters	2606/0/164
Goodness-of-fit on F^2	1.240
Final R indexes [I>=2σ (I)]	R_1 = 0.0138, wR_2 = 0.0312
Final R indexes [all data]	R_1 = 0.0147, wR_2 = 0.0315
Largest diff. peak/hole / e $Å^{-3}$	0.75/-0.41

Data collected, solved and refined by Adrian C Whitwood

Crystallographic data of 3-(pyridin-2-yl)coumarin)-tetracarbonyl rhenium **6'(H)**

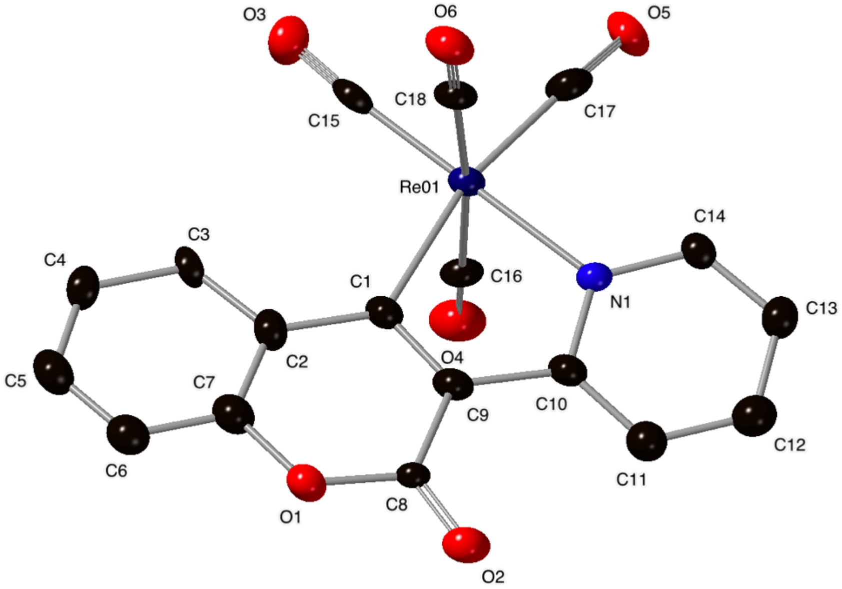

CCDC N°	2204326
Identification code	ijsf21064a
Empirical formula	$C_{18}H_8NO_6Re$
Formula weight	520.45
Temperature/K	110.00(10)
Crystal system	monoclinic
Space group	$P2_1/c$
a/Å	14.7322(5)
b/Å	12.1326(4)
c/Å	9.0965(3)
α/°	90
β/°	107.640(4)
γ/°	90
Volume/Å^3	1549.46(10)
Z	4
ρ_{calc}g/cm^3	2.231
μ/mm^{-1}	15.701
F(000)	984.0

Crystal size/mm^3	0.23 × 0.14 × 0.11
Radiation	Cu Kα (λ = 1.54184)
2Θ range for data collection/°	9.634 to 141.908
Index ranges	$-17 \leq h \leq 16$, $-14 \leq k \leq 14$, $-10 \leq l \leq 10$
Reflections collected	5754
Independent reflections	2923 [R_{int} = 0.0276, R_{sigma} = 0.0350]
Data/restraints/parameters	2923/0/235
Goodness-of-fit on F^2	1.051
Final R indexes [I>=2σ (I)]	R_1 = 0.0605, wR_2 = 0.1401
Final R indexes [all data]	R_1 = 0.0710, wR_2 = 0.1494
Largest diff. peak/hole / e Å^{-3}	10.93/-2.23

Data collected and refined by Theo F. N. Tanner

5.0 Representative ^{1}H and ^{13}C NMR Spectra

Figure S7 ^{1}H NMR spectrum of 4-(*7*-NEt$_2$)

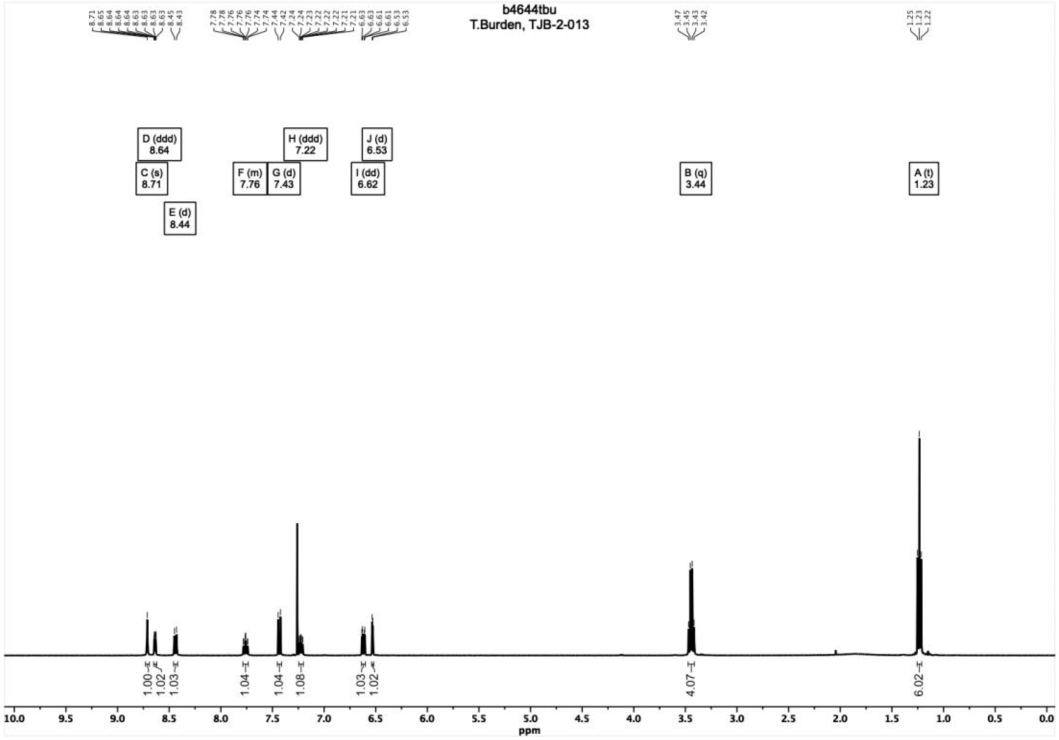

Figure S8 ^{13}C NMR spectrum of 4-(*7*-NEt$_2$)

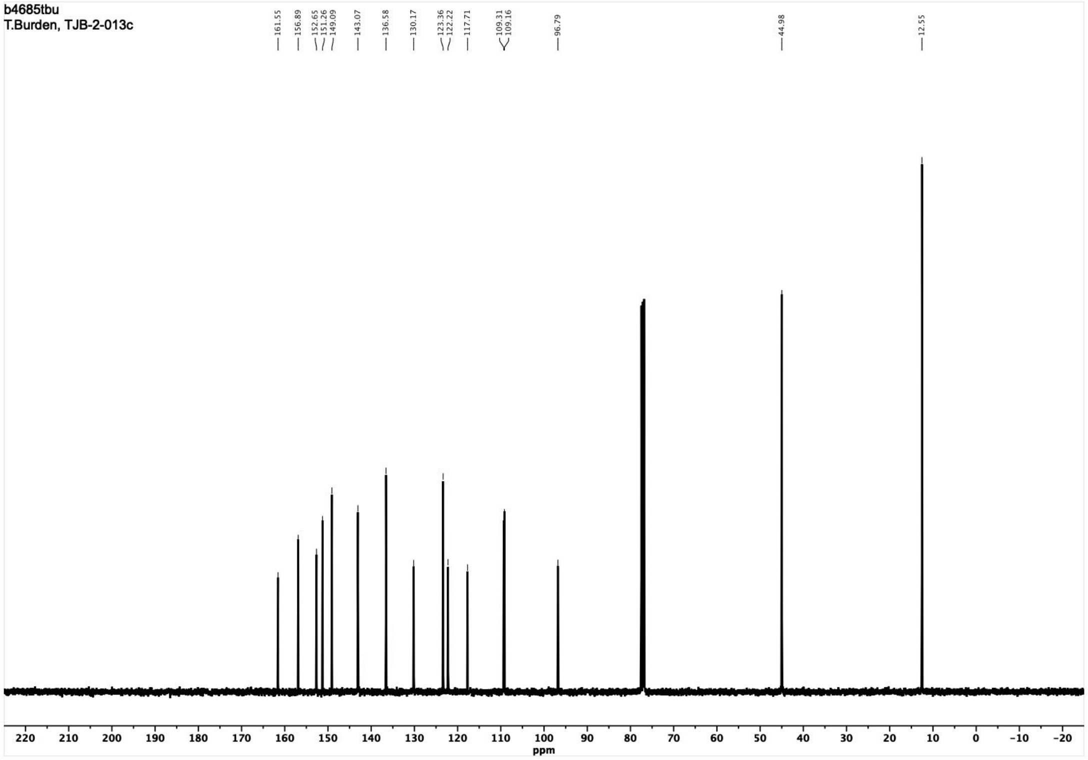

Figure S9 ^{1}H NMR spectrum of 6-(*7*-NEt2)

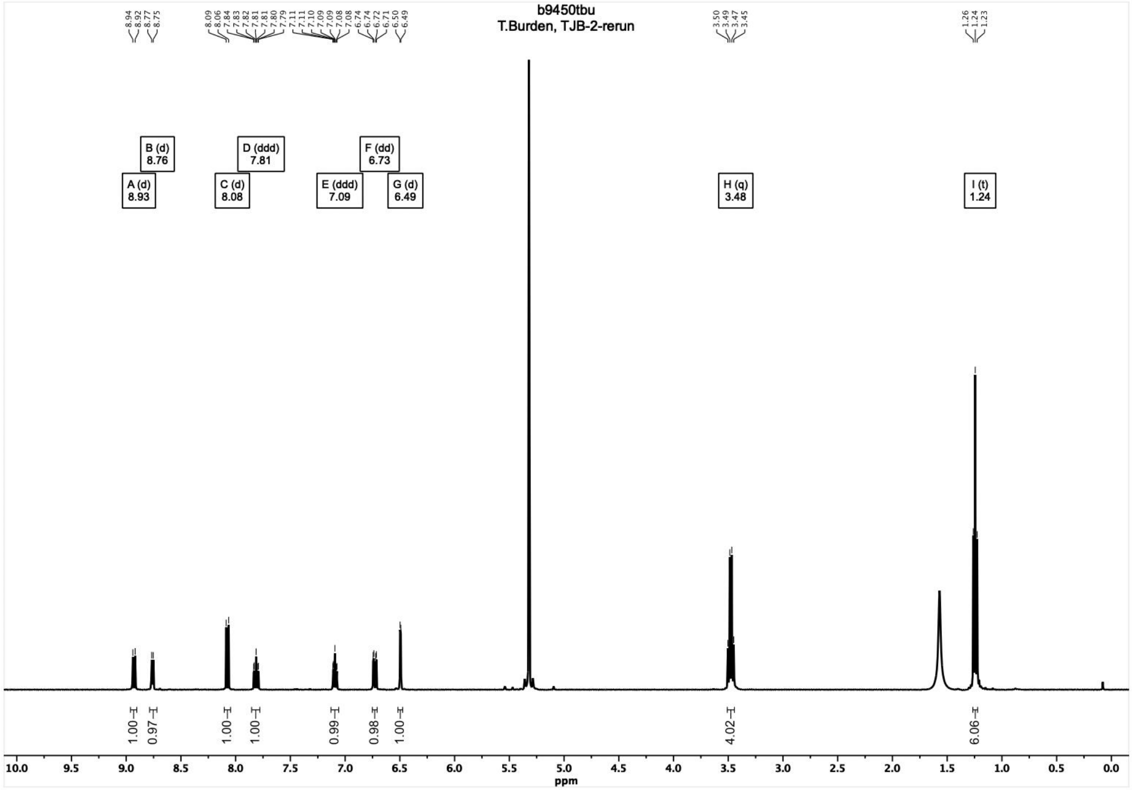

Figure S10 ^{13}C NMR spectrum of 6-(*7*-NEt2)

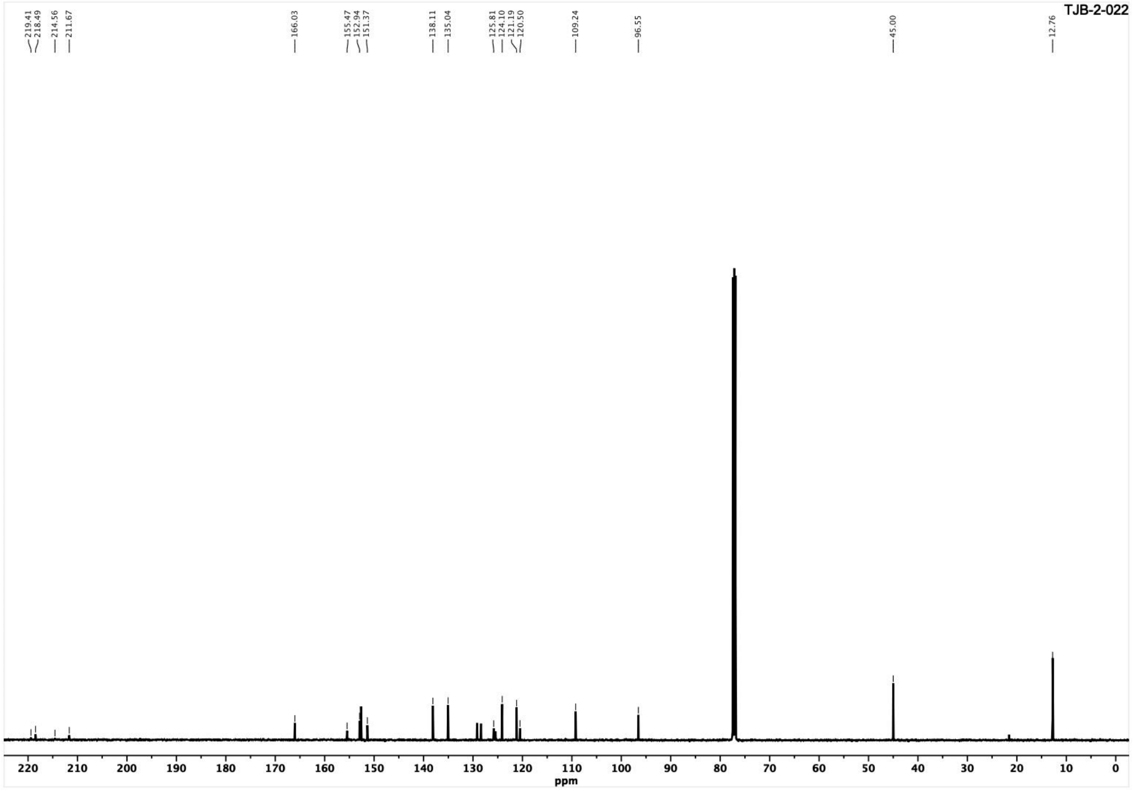

Figure S11 ^{1}H NMR spectrum of 5a-(*7*-NEt2)

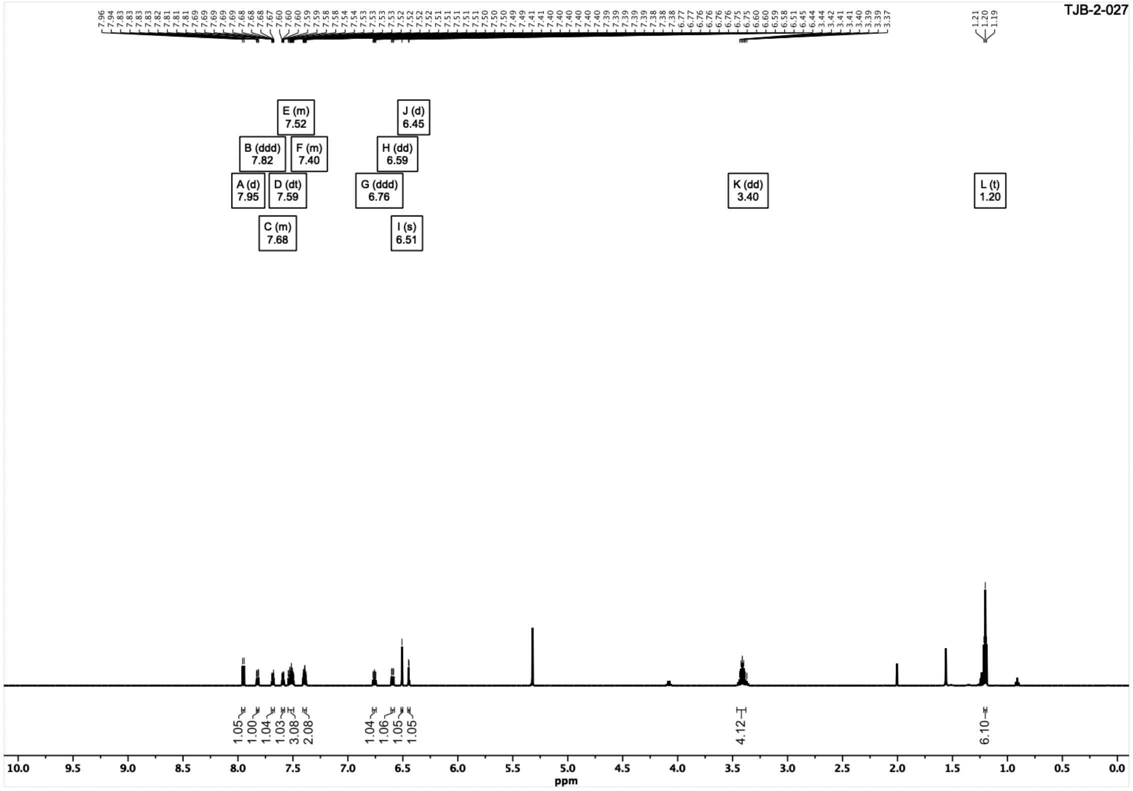

Figure S12 ^{13}C NMR spectrum of 5a-(*7*-NEt2)

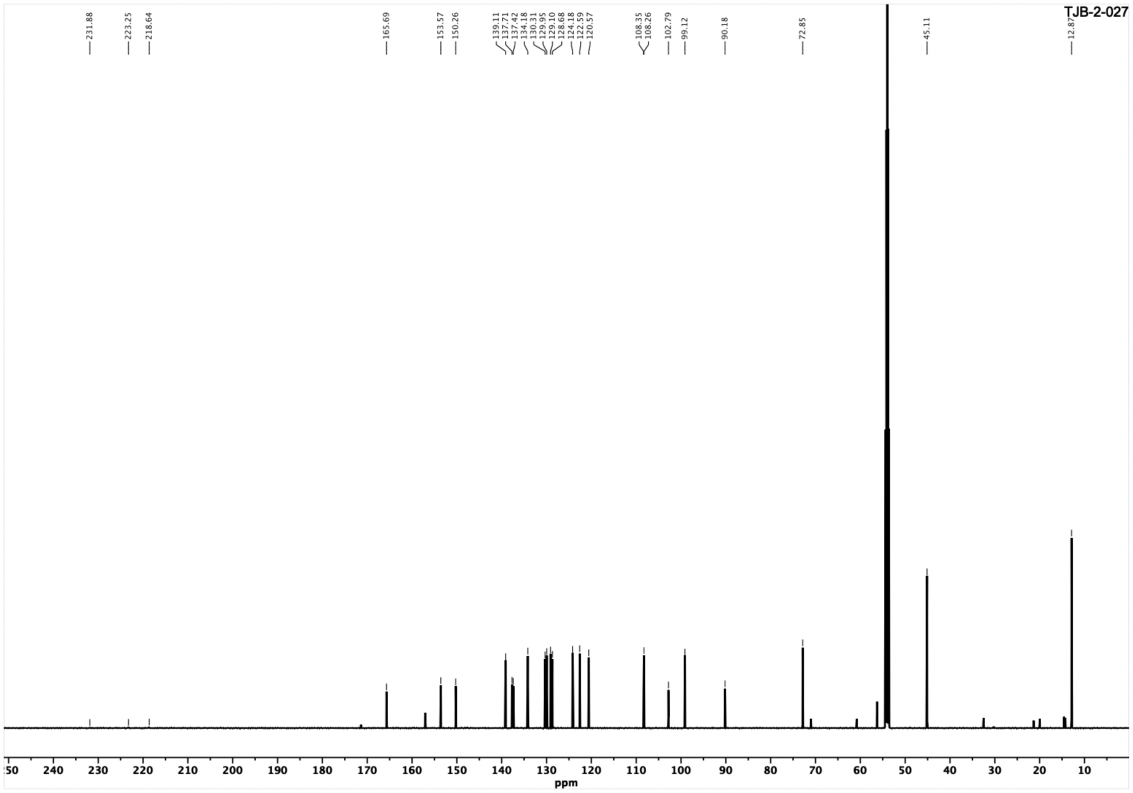

Figure S13 ^{1}H NMR spectrum of [D_1]-5a-(*7*-NEt_2)

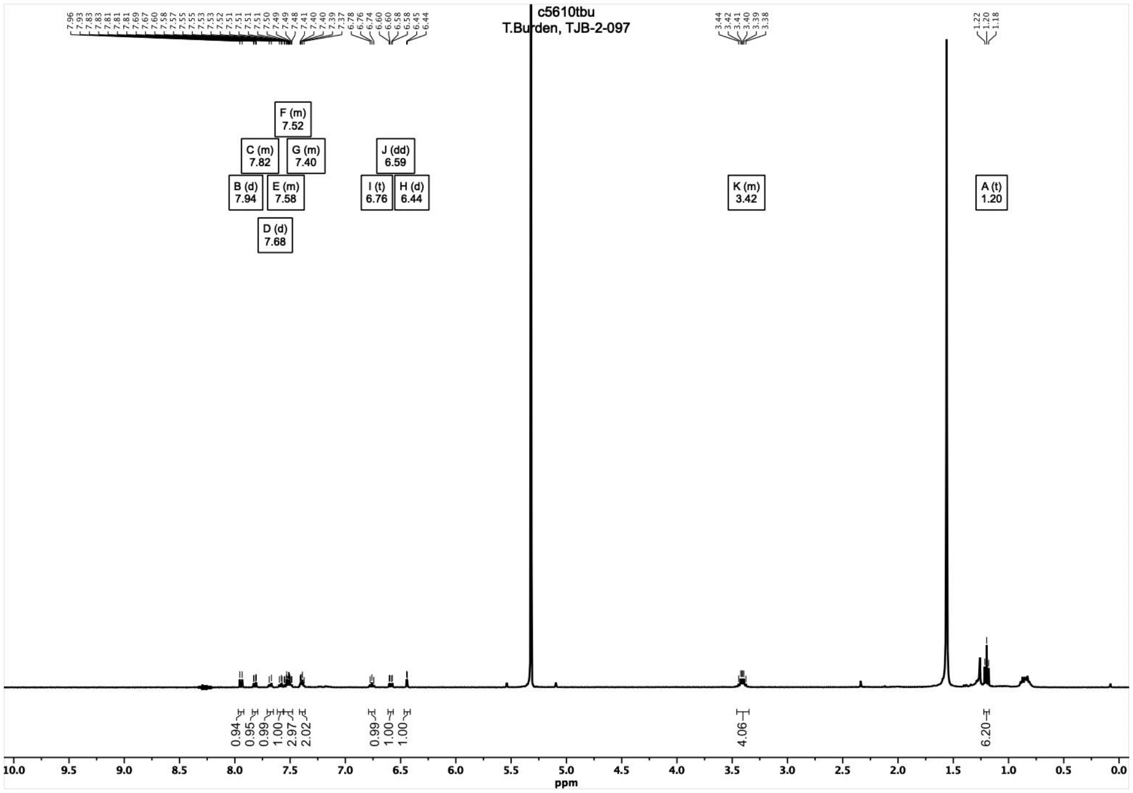

Figure S14 ^{1}H NMR spectrum of 5b-(7-NEt$_2$)

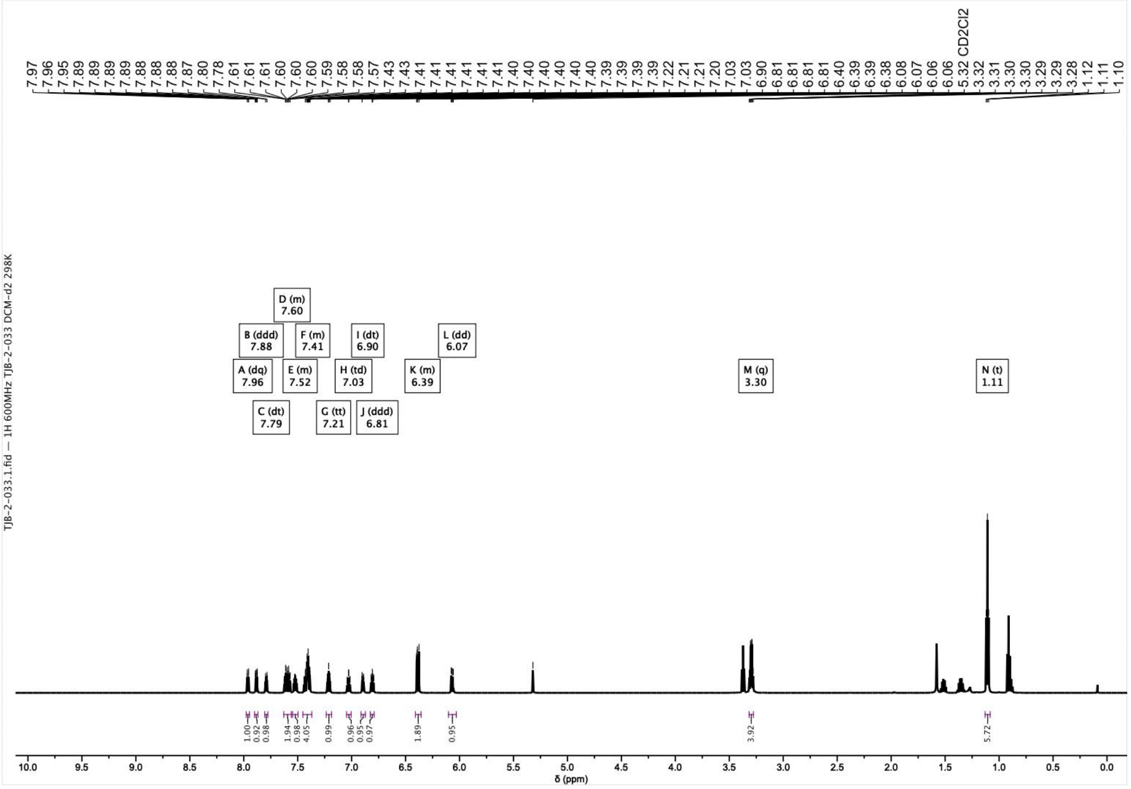

Figure S15 ^{13}C NMR spectrum of 5b-(7-NEt$_2$)

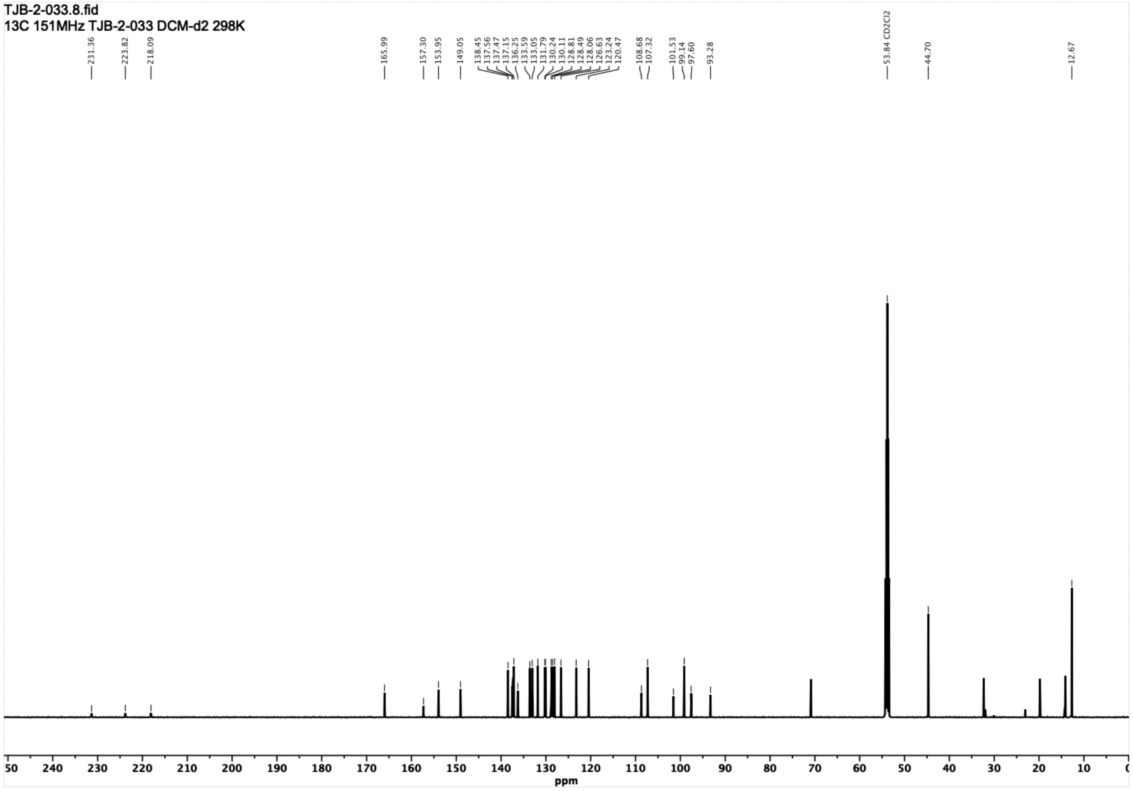

Figure S16 ^{1}H NMR spectrum of 5c-(*7*-NEt$_2$)

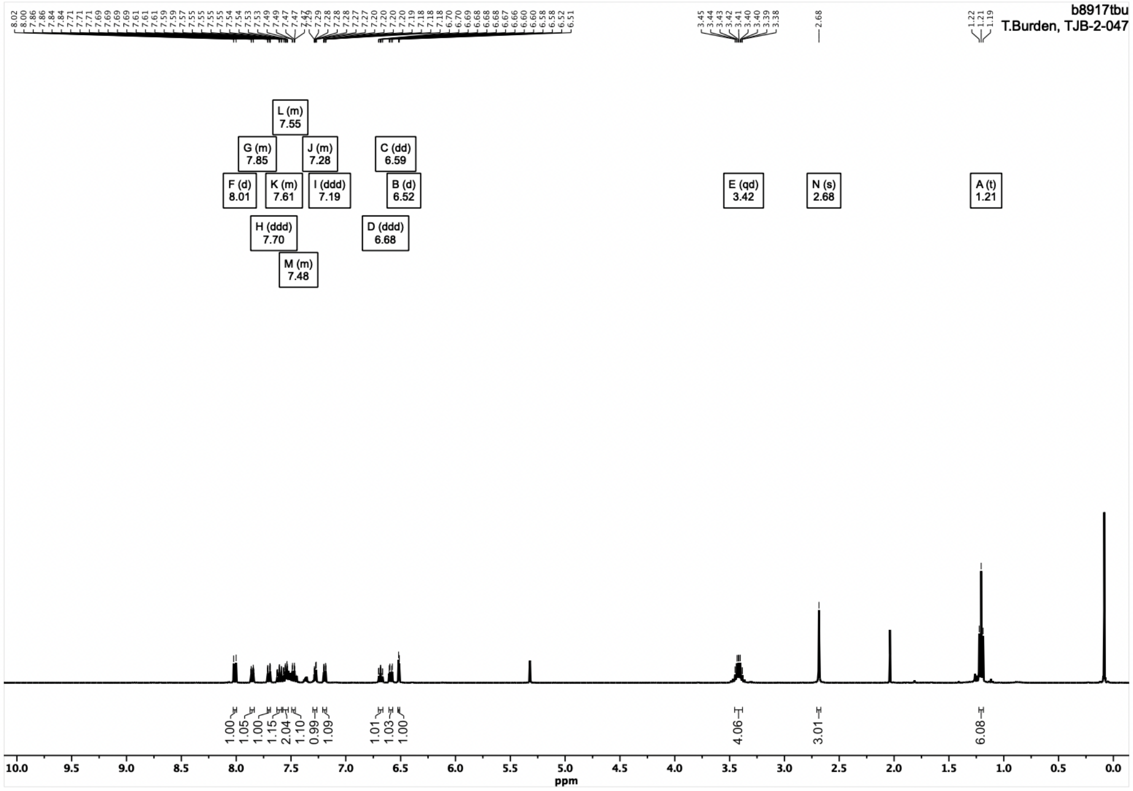

Figure S17 ^{1}H NMR spectrum of 5c-(*7*-NEt$_2$)

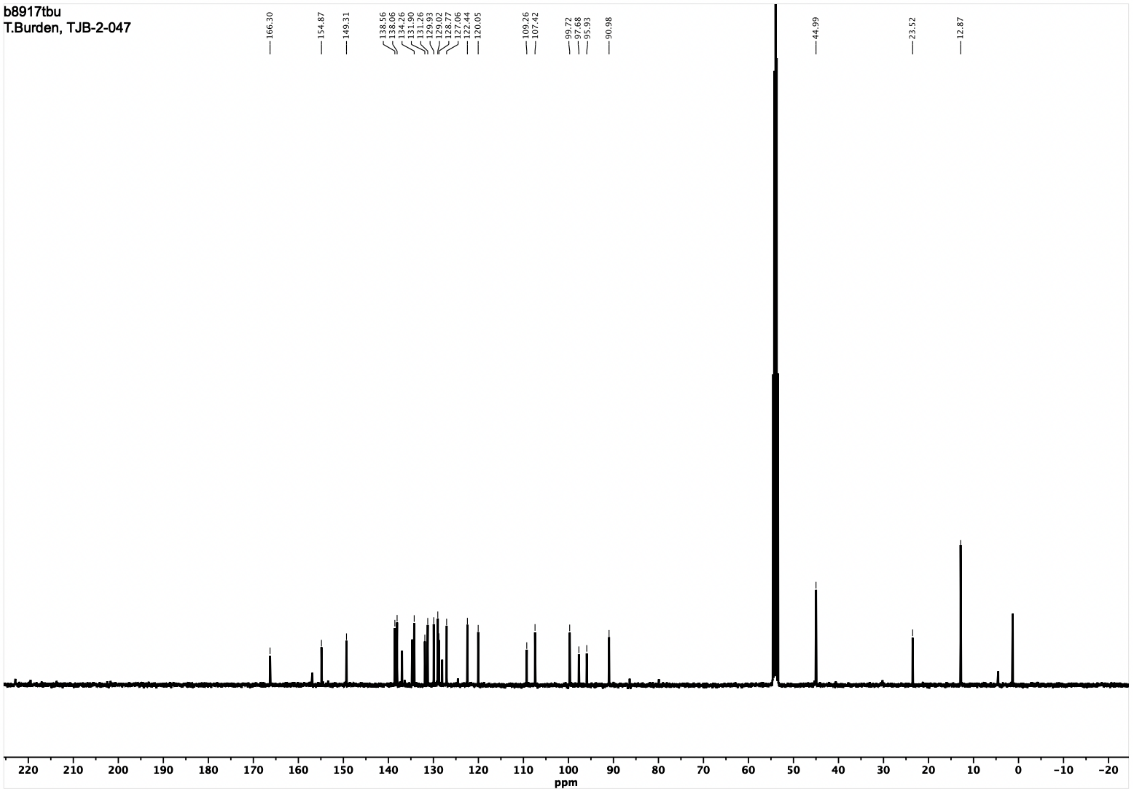

Figure S18 ^{1}H NMR spectrum of 5d-(*7*-NEt$_2$)

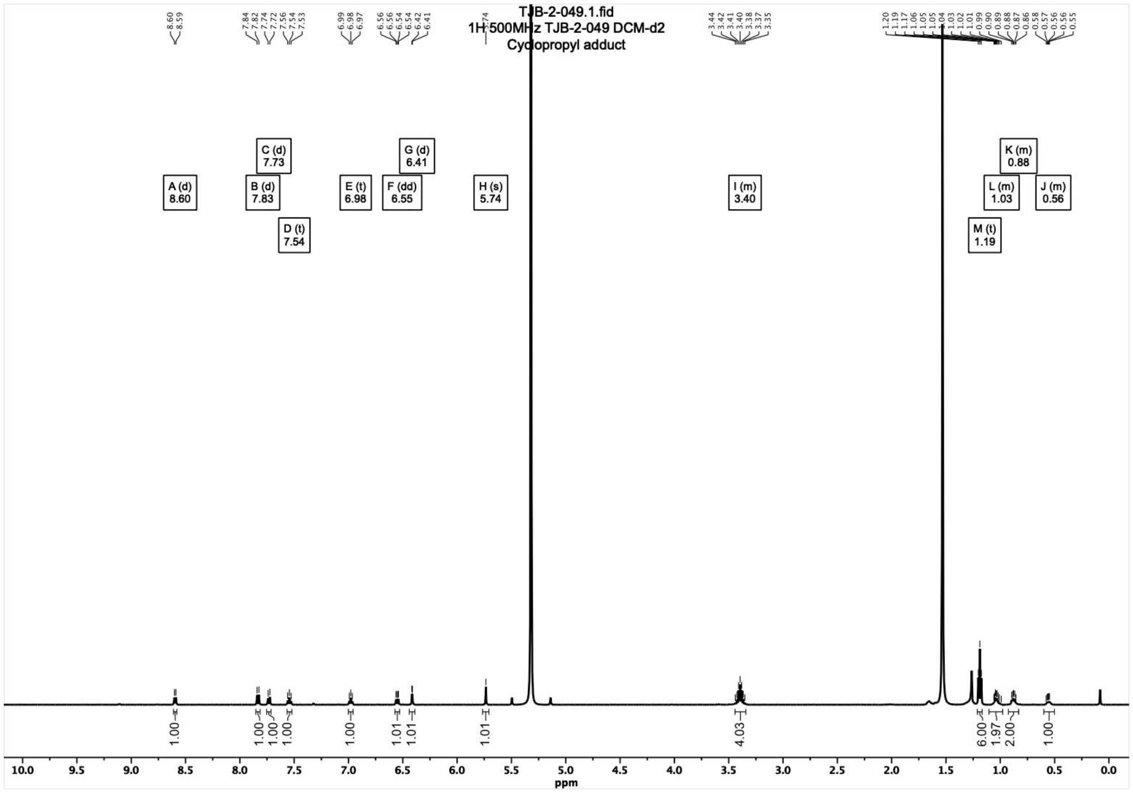

Figure S19 ^{13}C NMR spectrum of 5d-(*7*-NEt$_2$)

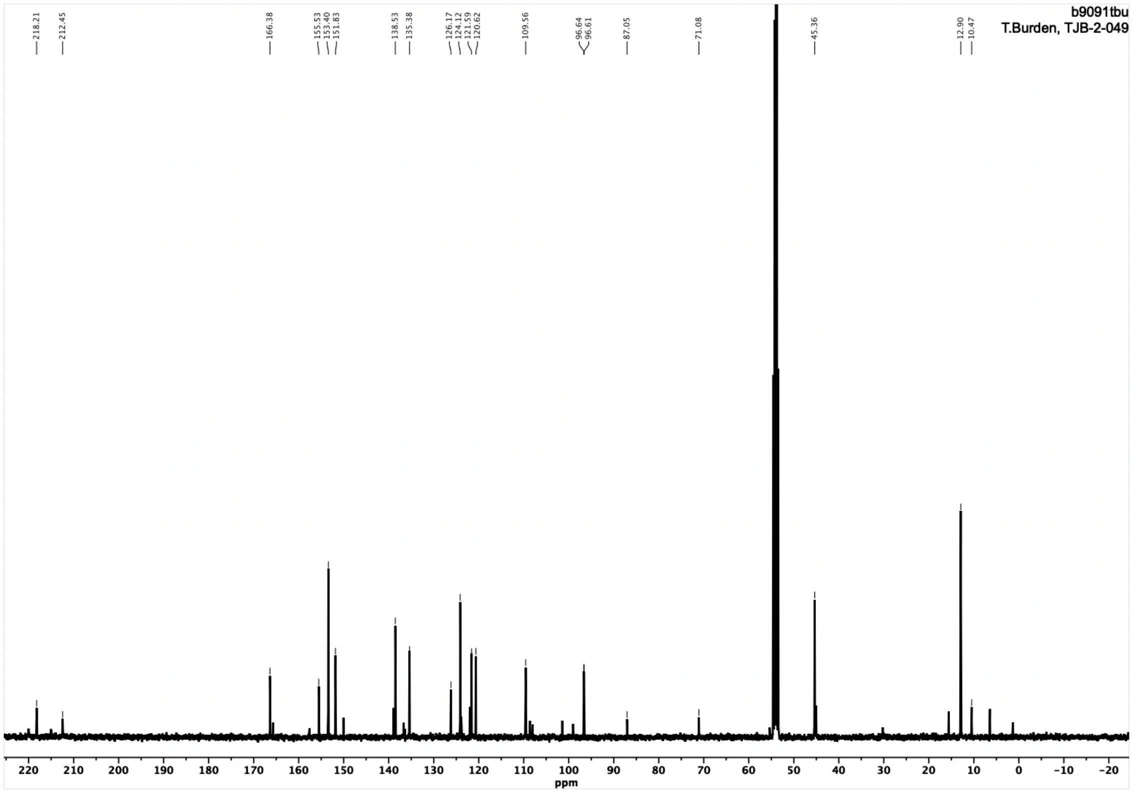

Figure S20 ^{1}H NMR spectrum of 5e-(*7*-NEt_2)

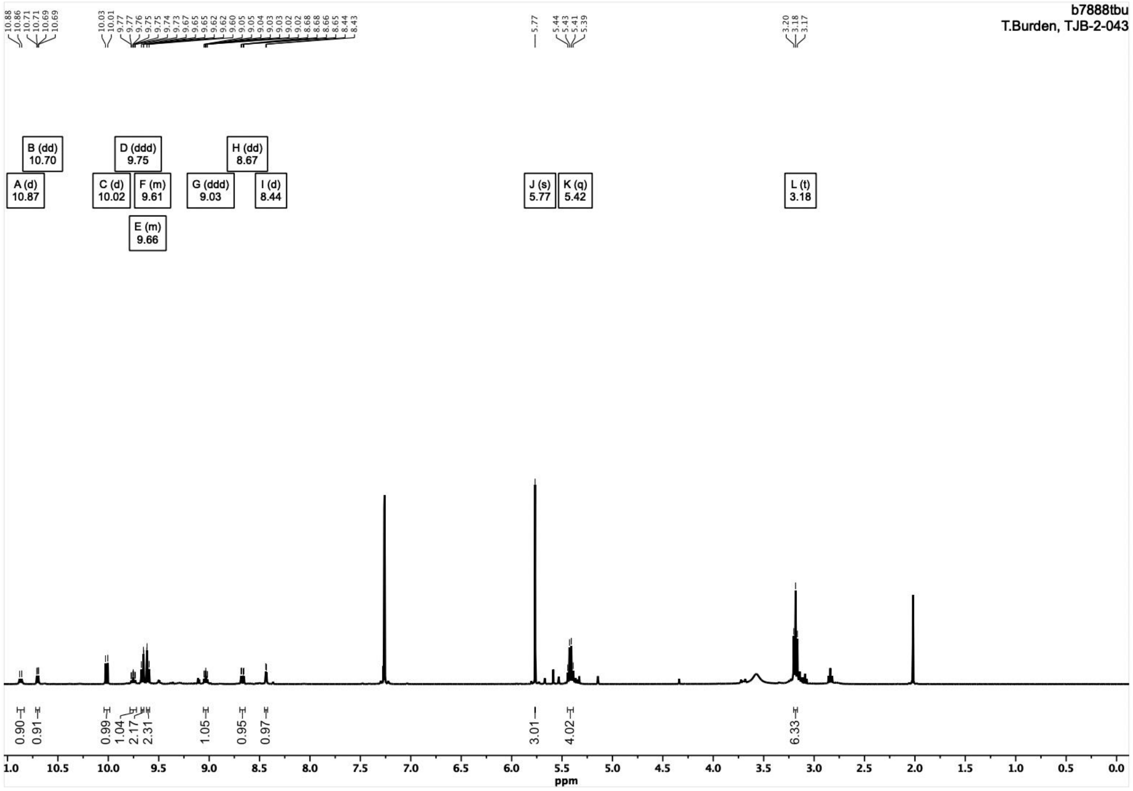

Figure S21 ^{19}F NMR spectrum of 5e-(*7*-NEt_2)

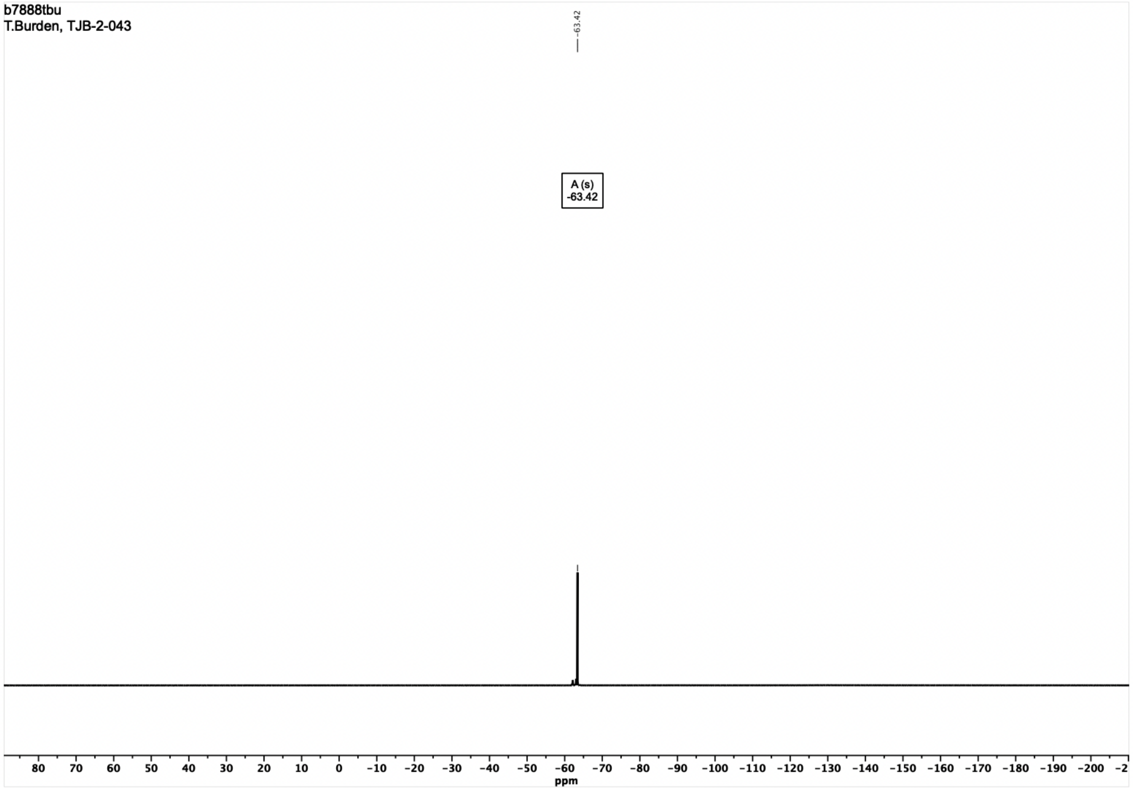

Figure S22 ^{13}C NMR spectrum of 5e-(*7*-NEt$_2$)

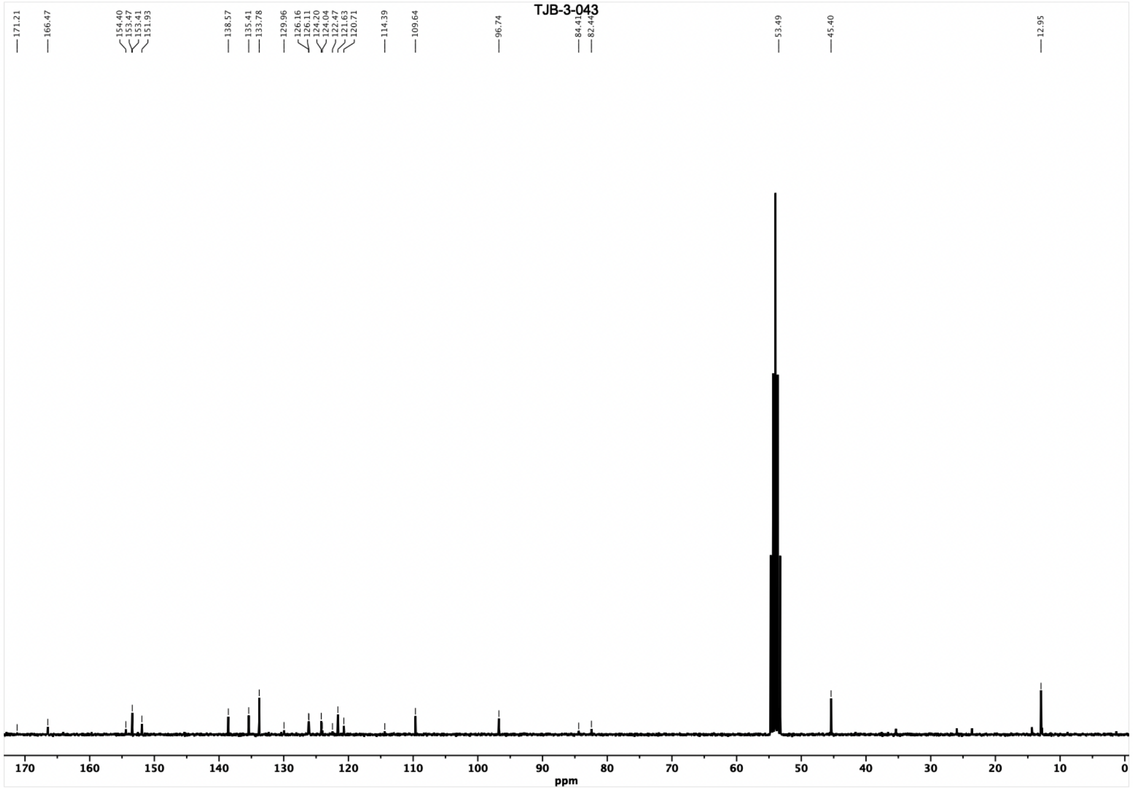

Figure S23 ^{1}H NMR spectrum of 5f-(*7*-NEt$_2$)

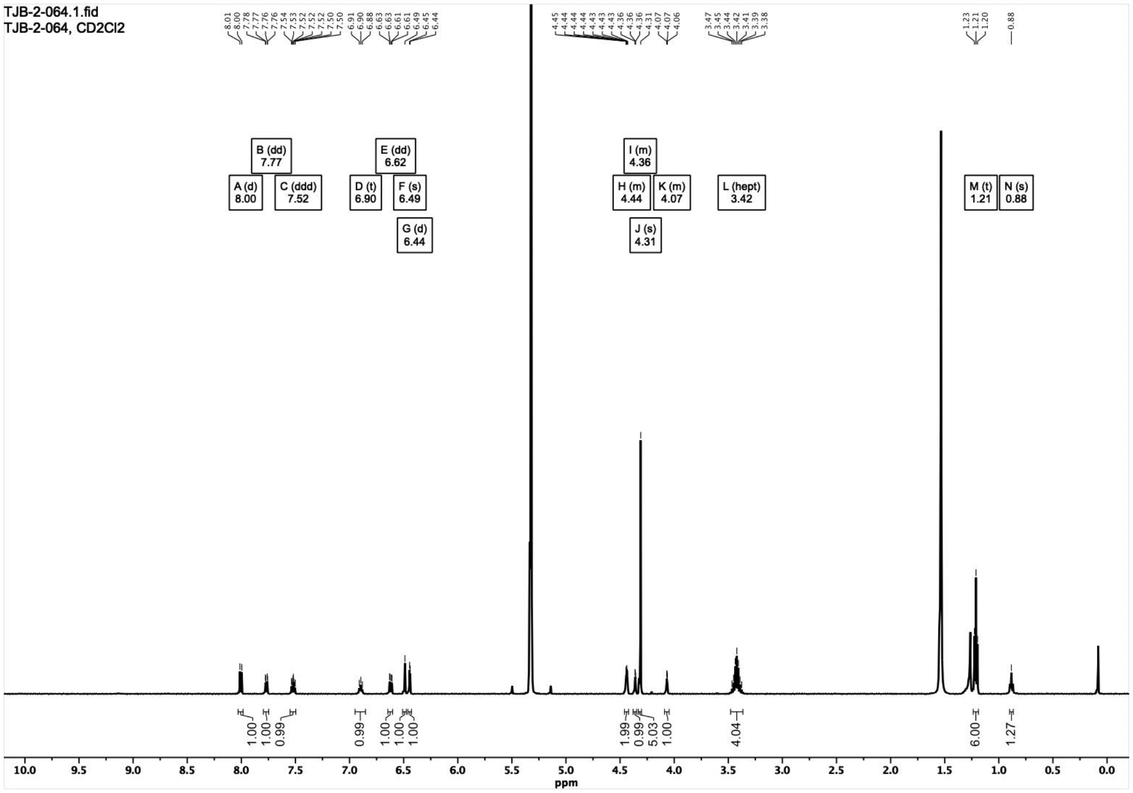

Figure S24 ^{13}C NMR spectrum of 5f-(*7*-NEt$_2$)

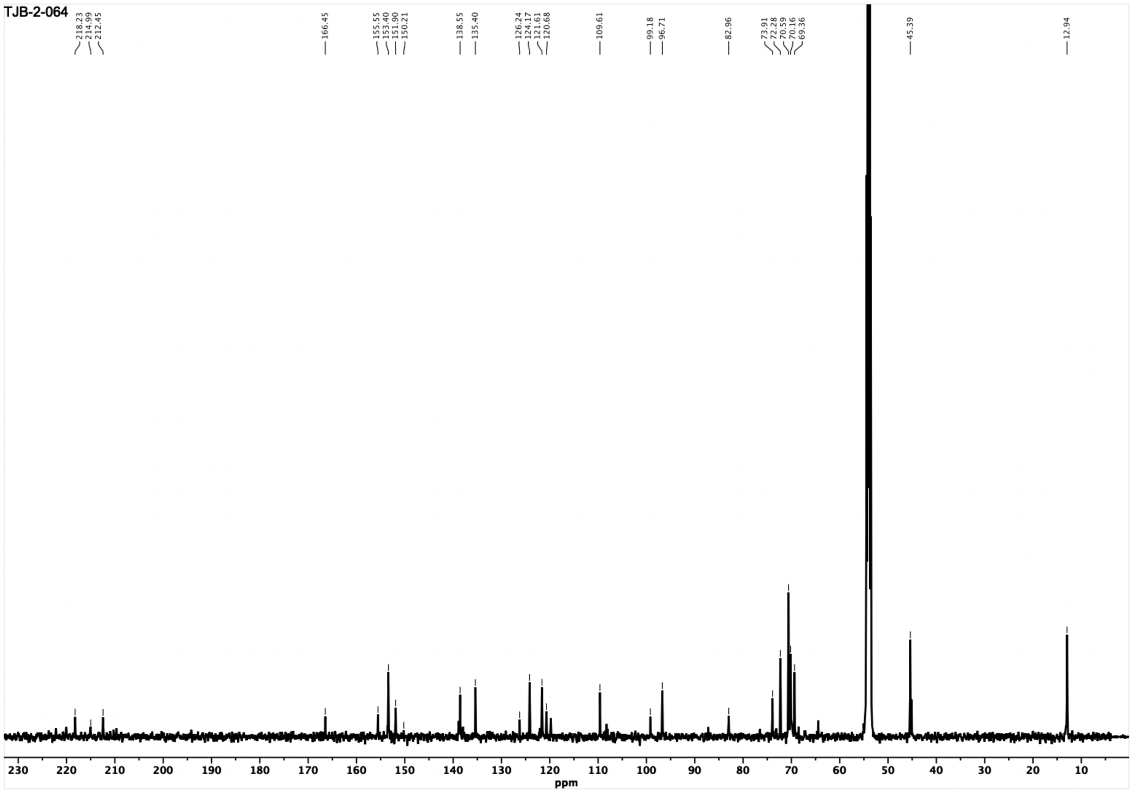

Figure S25 ^{1}H NMR spectrum of 5g-(*7*-NEt2)

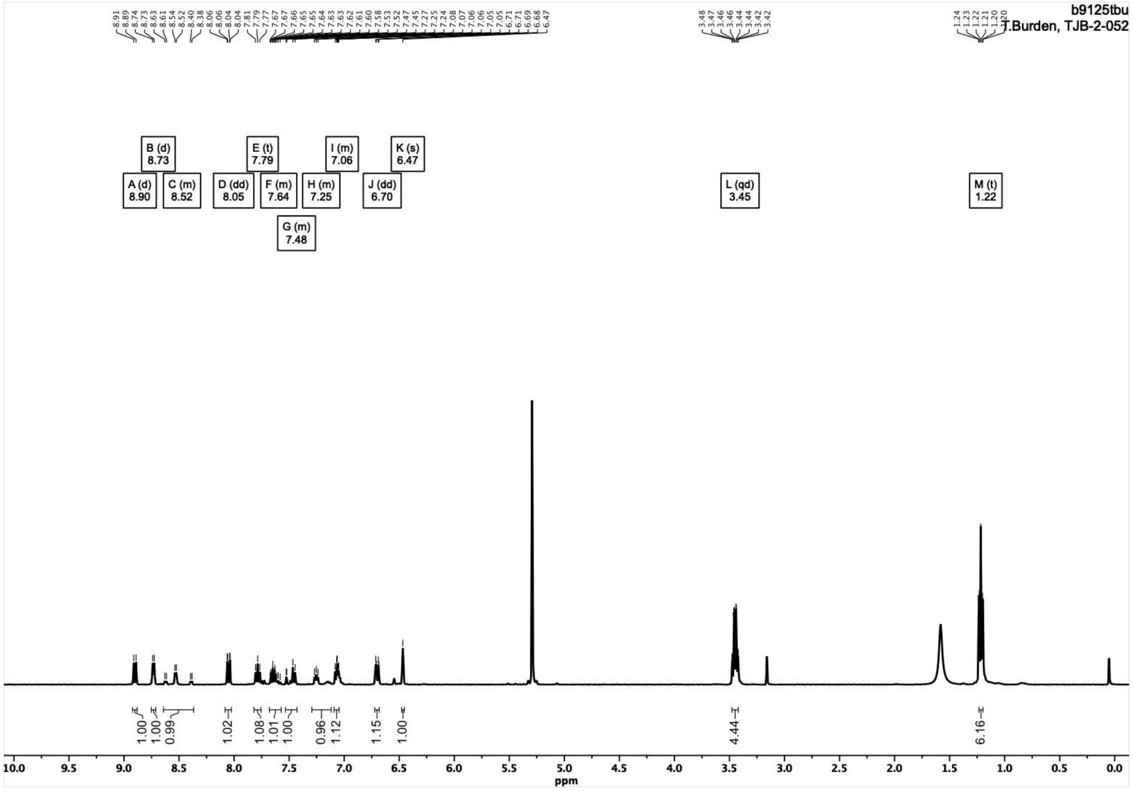

Figure S26 ^{13}C NMR spectrum of 5g-(*7*--NEt2)

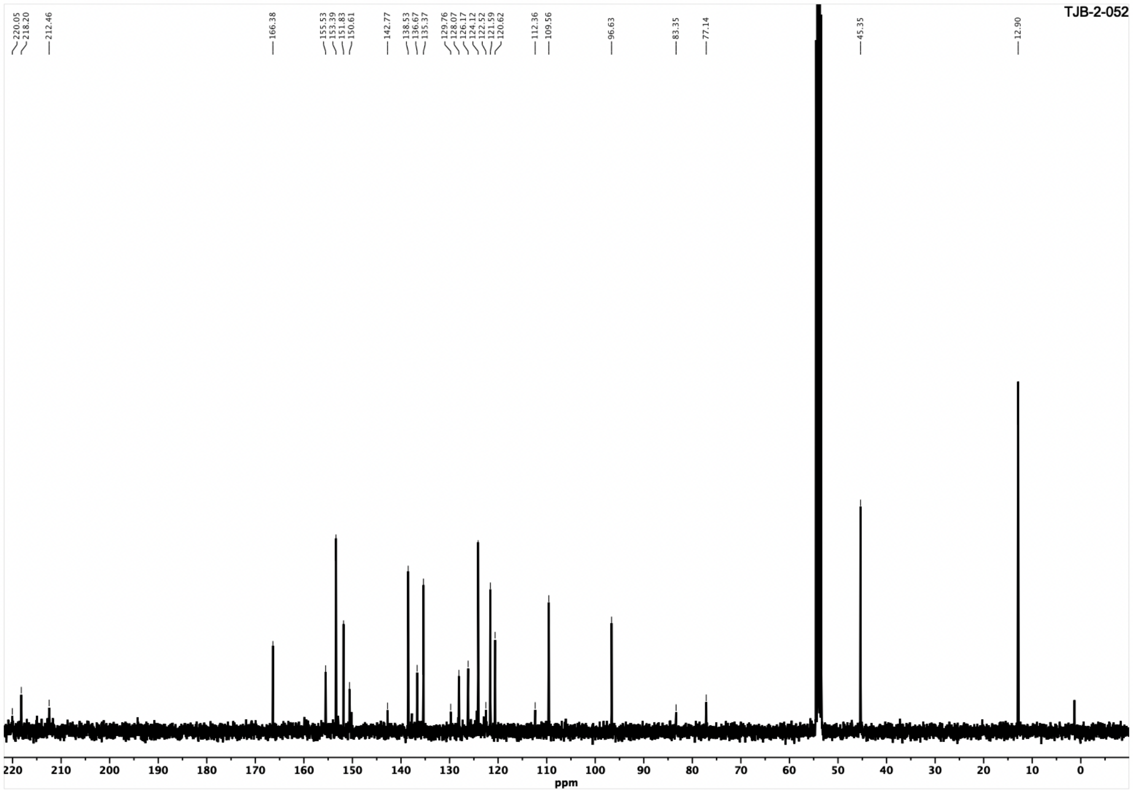

Figure S27 ^{1}H NMR spectrum of 5h-(*7*-NEt$_2$)

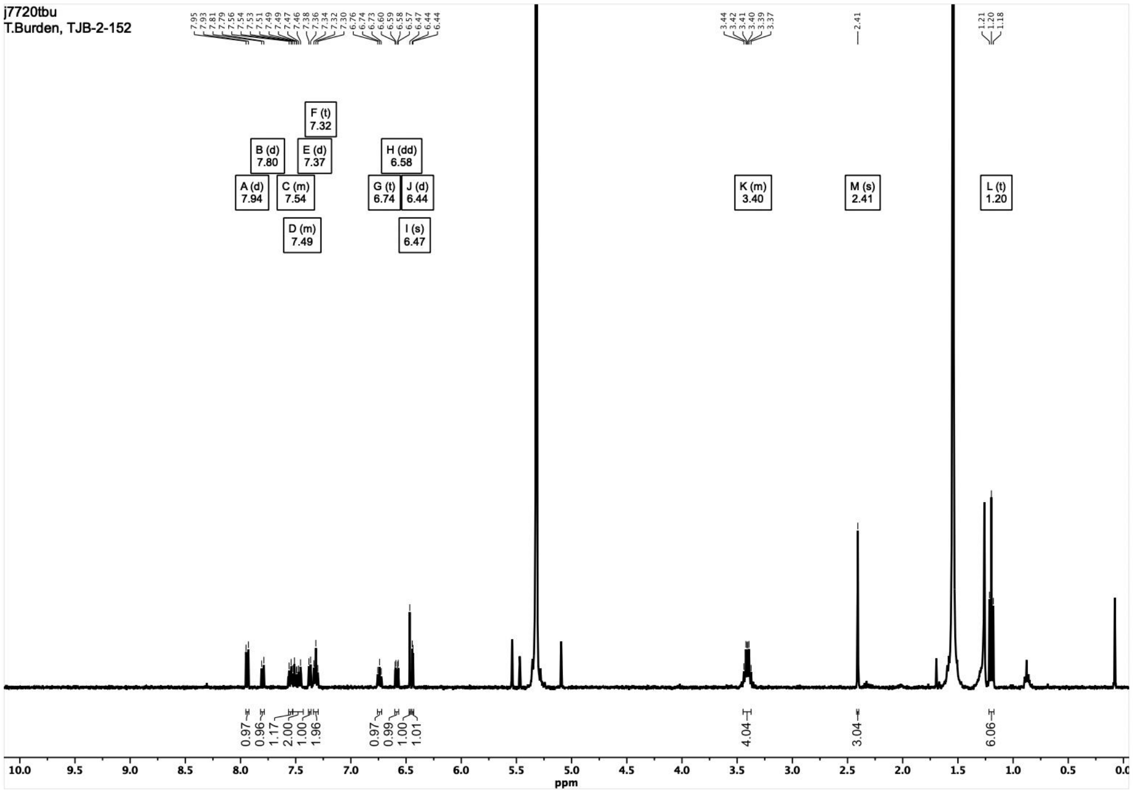

Figure S28 ^{13}C NMR spectrum of 5g-(*7*-NEt$_2$)

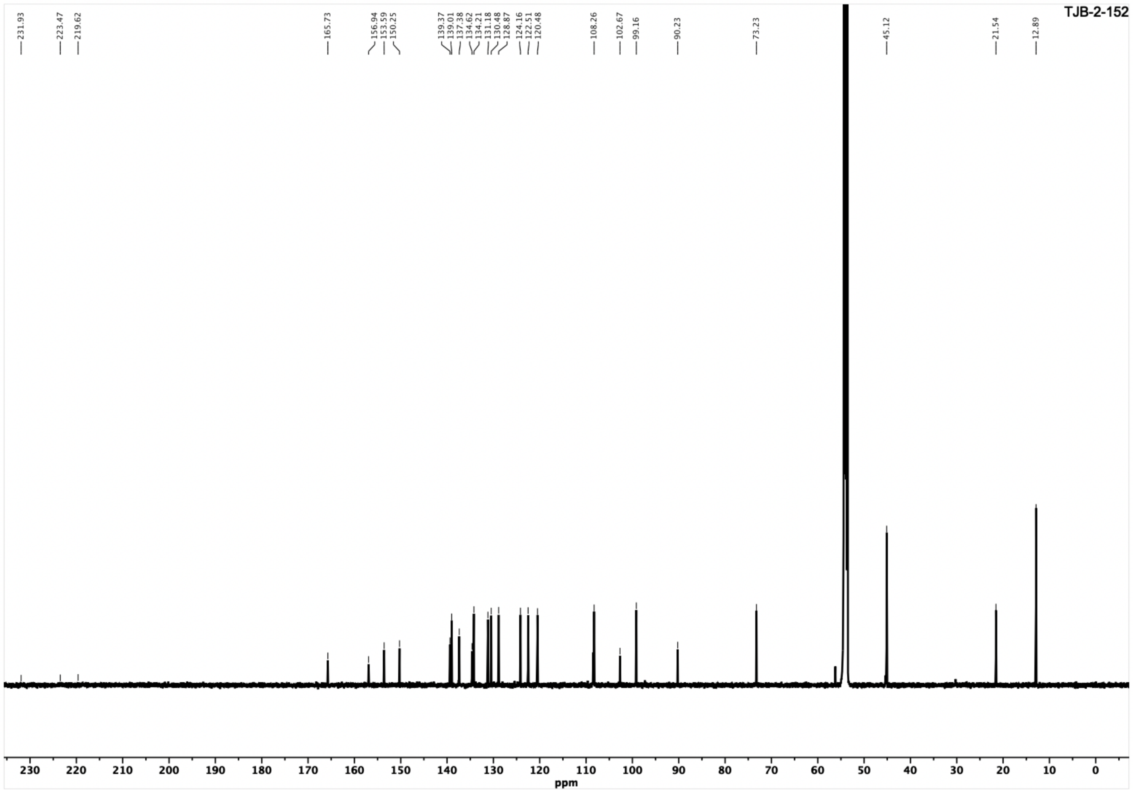

Figure S29 ^{13}C NMR spectrum of 4-(*7*-OH)

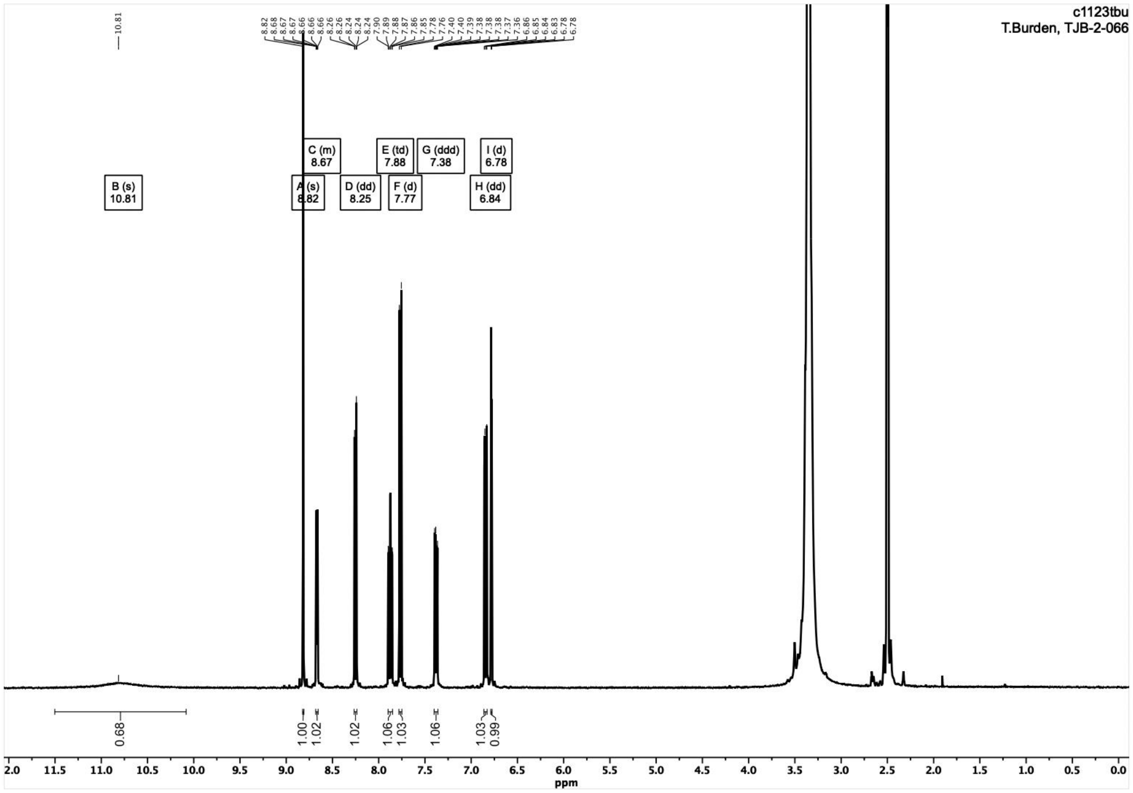

Figure S30 ^{13}C NMR spectrum of 4-(*7*-OH)

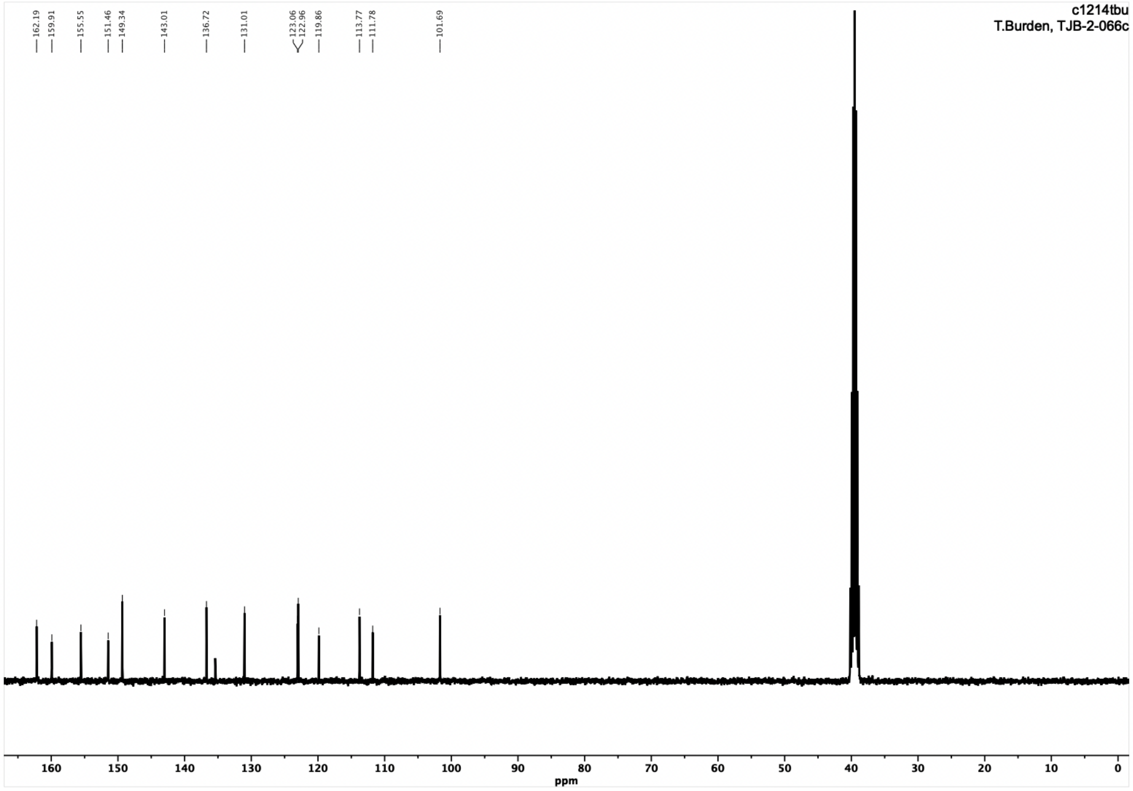

Figure S31 ^{1}H NMR spectrum of 6-(*7*-OH)

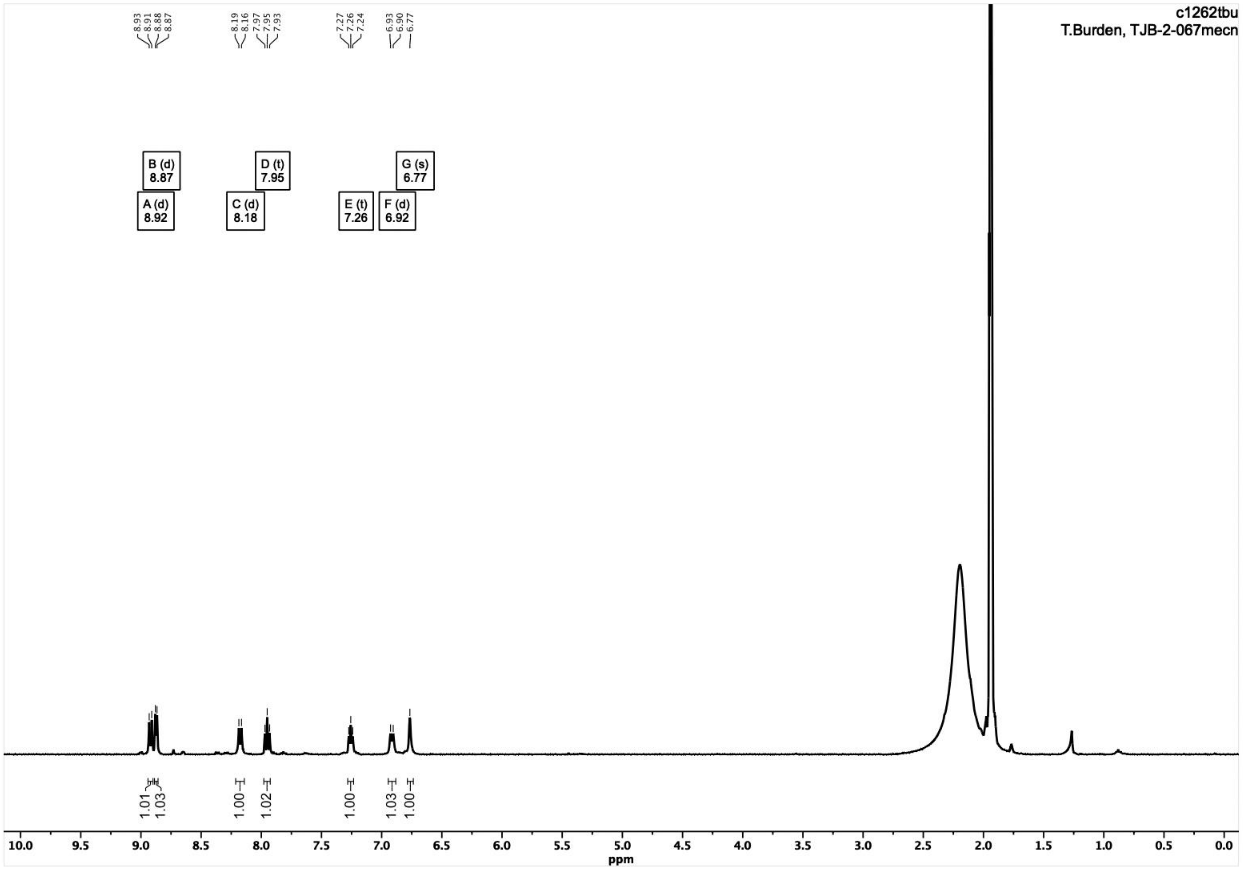

Figure S32 ^{1}H NMR spectrum of 5a-(*7*-OH)

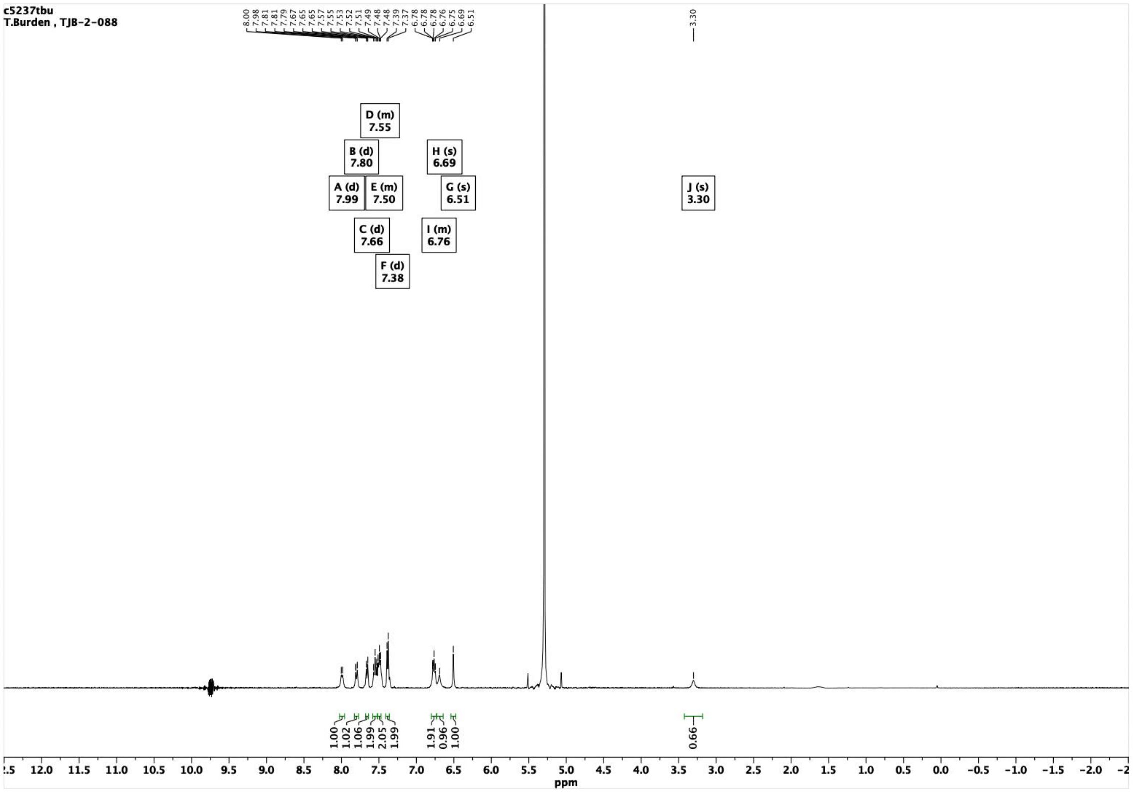

Figure S33 ^{13}C NMR spectrum of 5a-(*7*-OH)

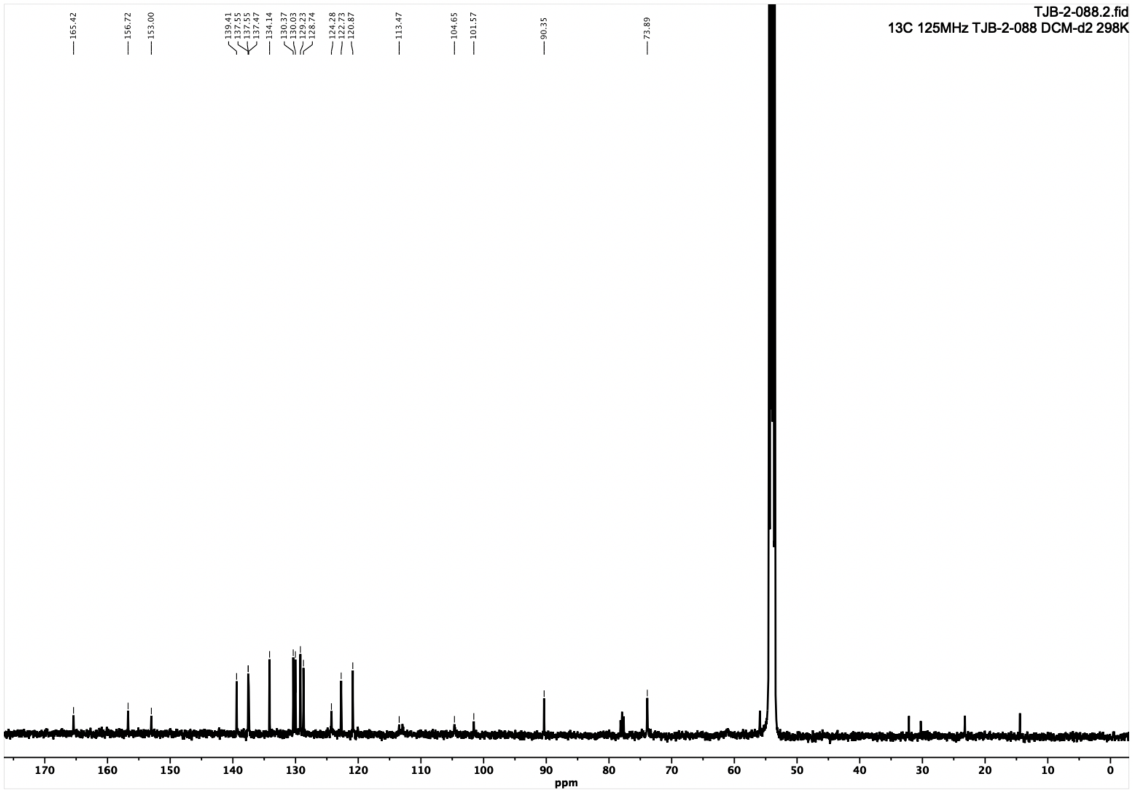

Figure S34 ^{1}H NMR spectrum of 4-(*7*-OMe)

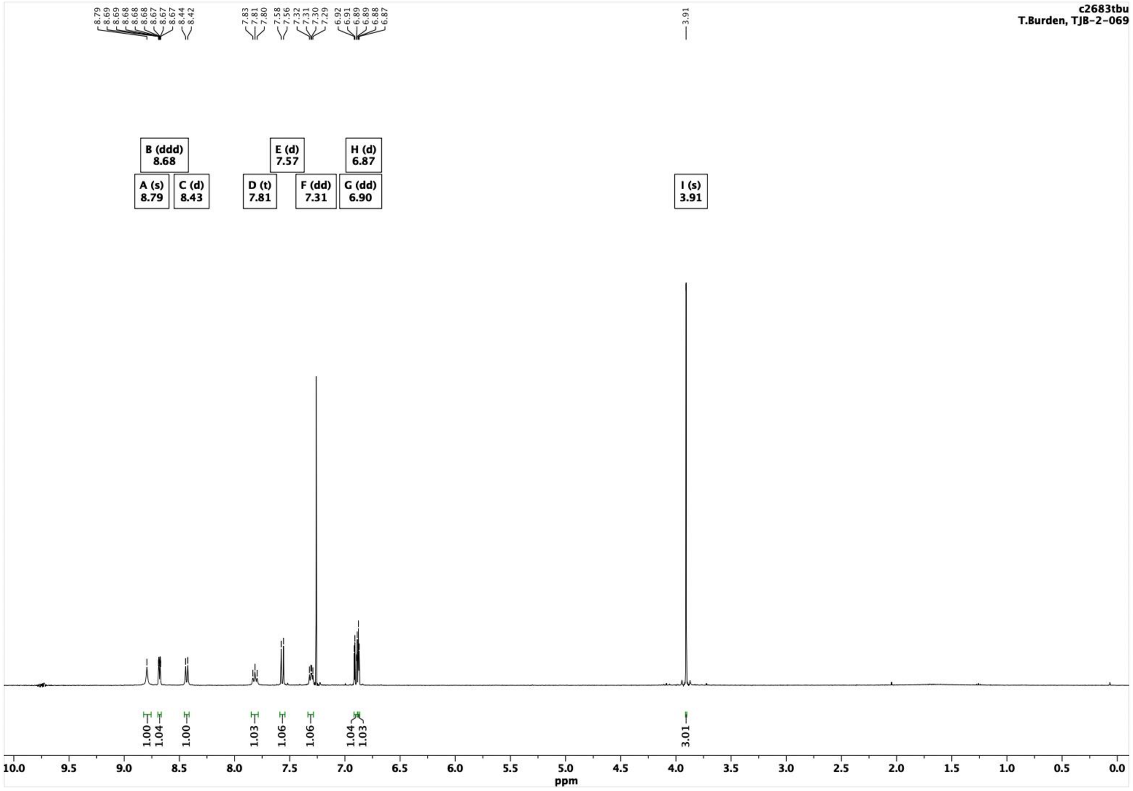

Figure S35 ^{13}C NMR spectrum of 4-(*7*-OMe)

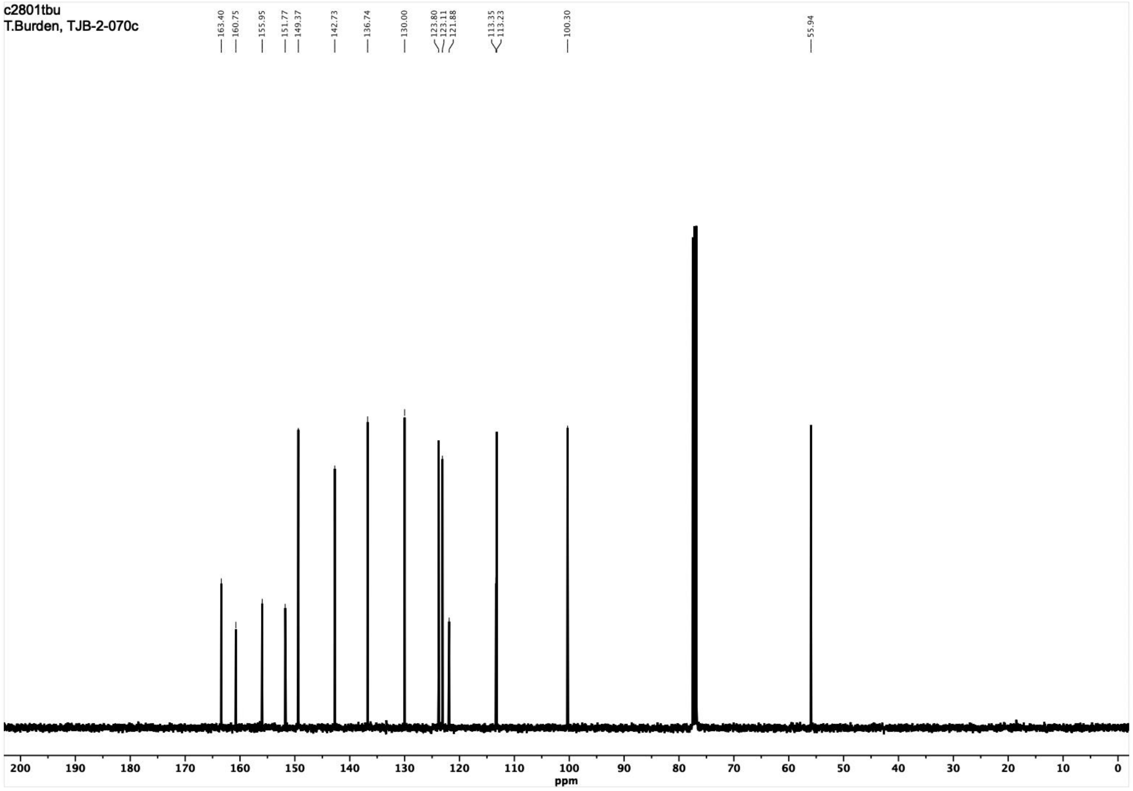

Figure S36 ^{1}H NMR spectrum of 6-(*7*-OMe)

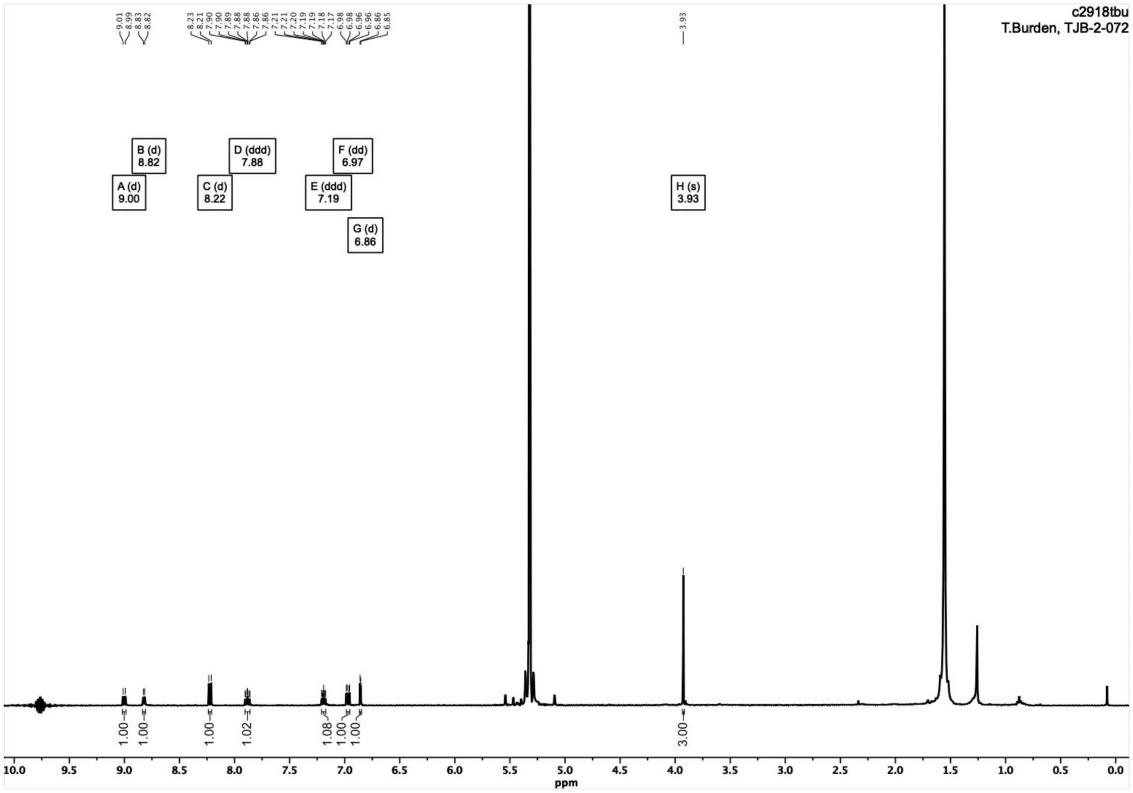

Figure S37 ^{13}C NMR spectrum of 6-(*7*-OMe)

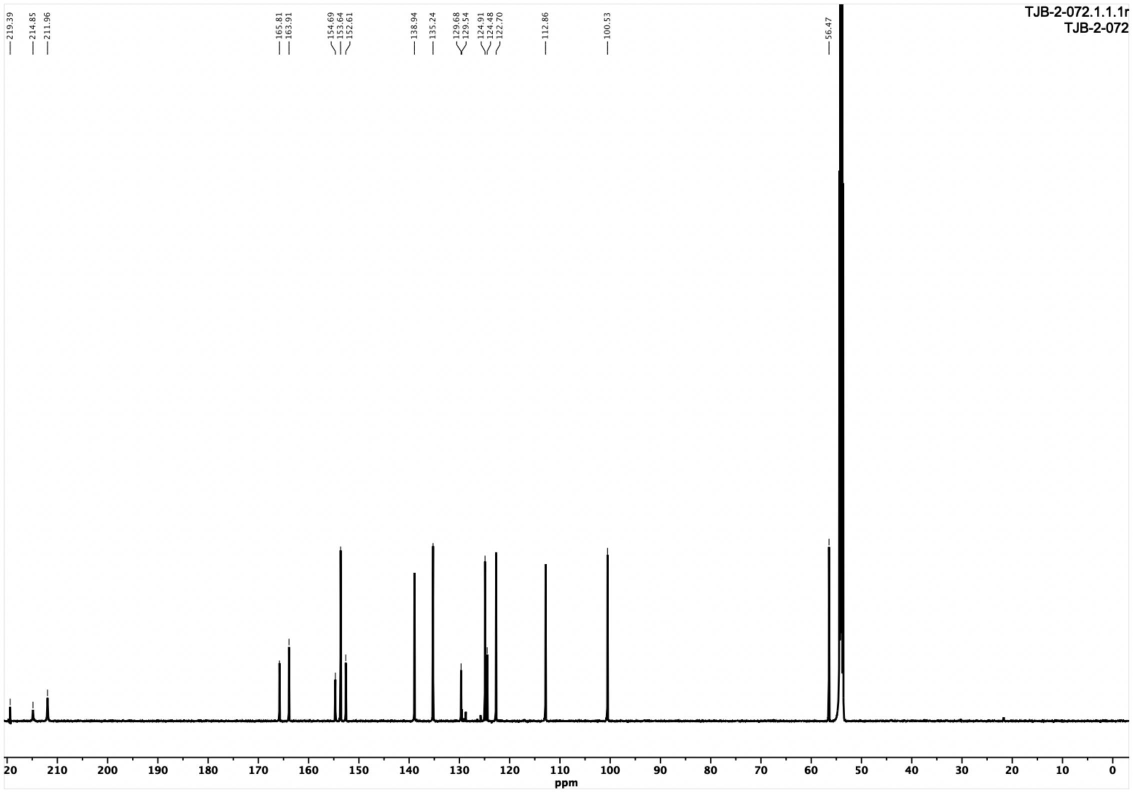

Figure S38 ^{1}H NMR spectrum of 5a-(*7*-OMe)

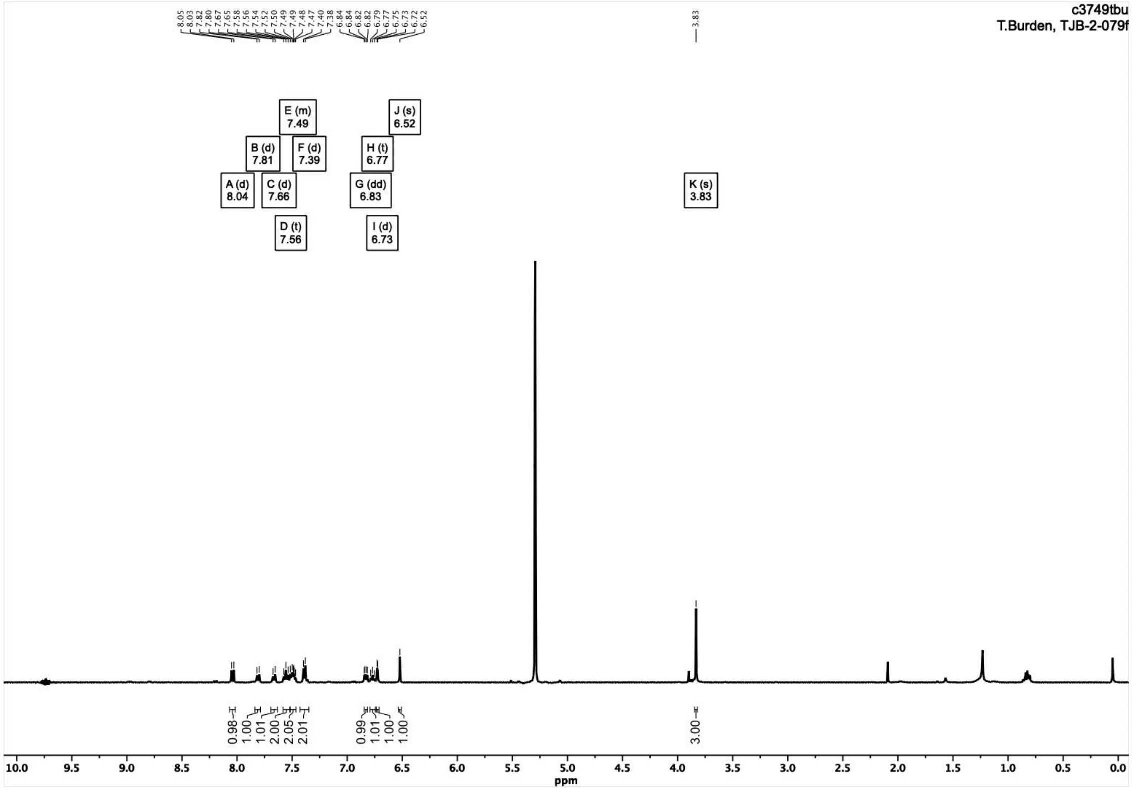

Figure S39 ^{13}C NMR spectrum of 5a-(*7*-OMe)

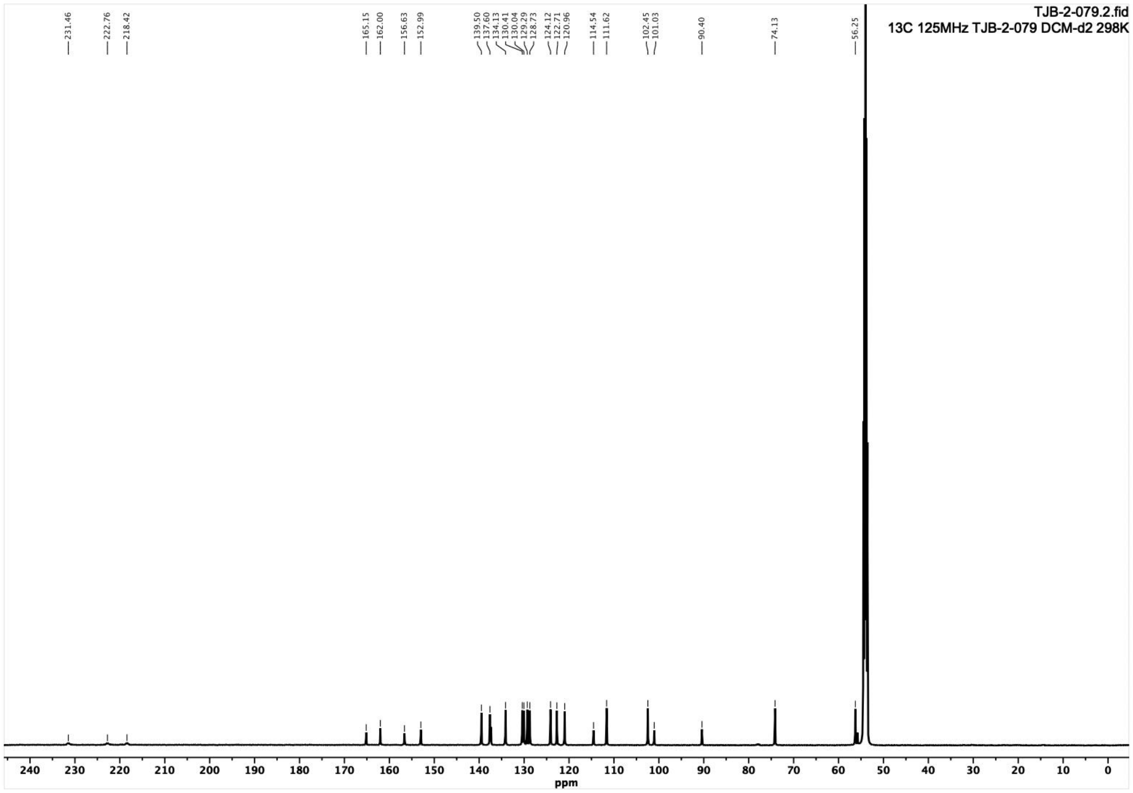

Figure S40 ^{1}H NMR spectrum of 4-(H)

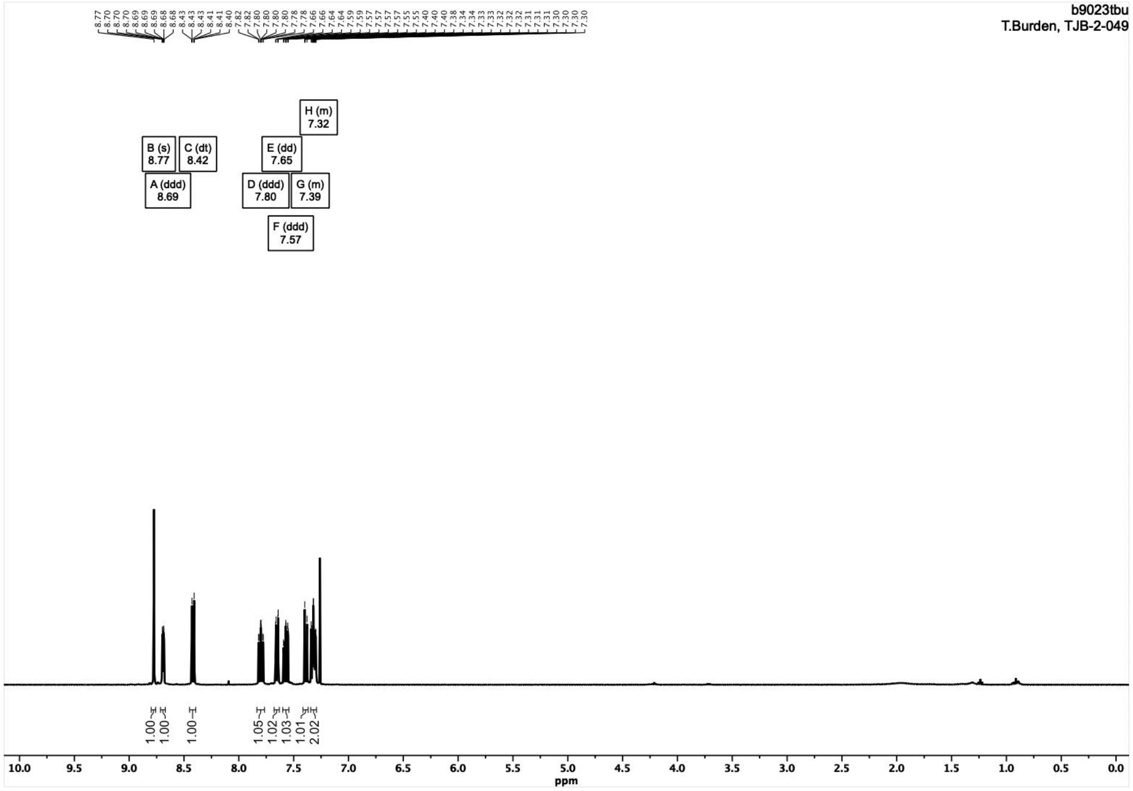

Figure S41 ^{13}C NMR spectrum of 4-(H)

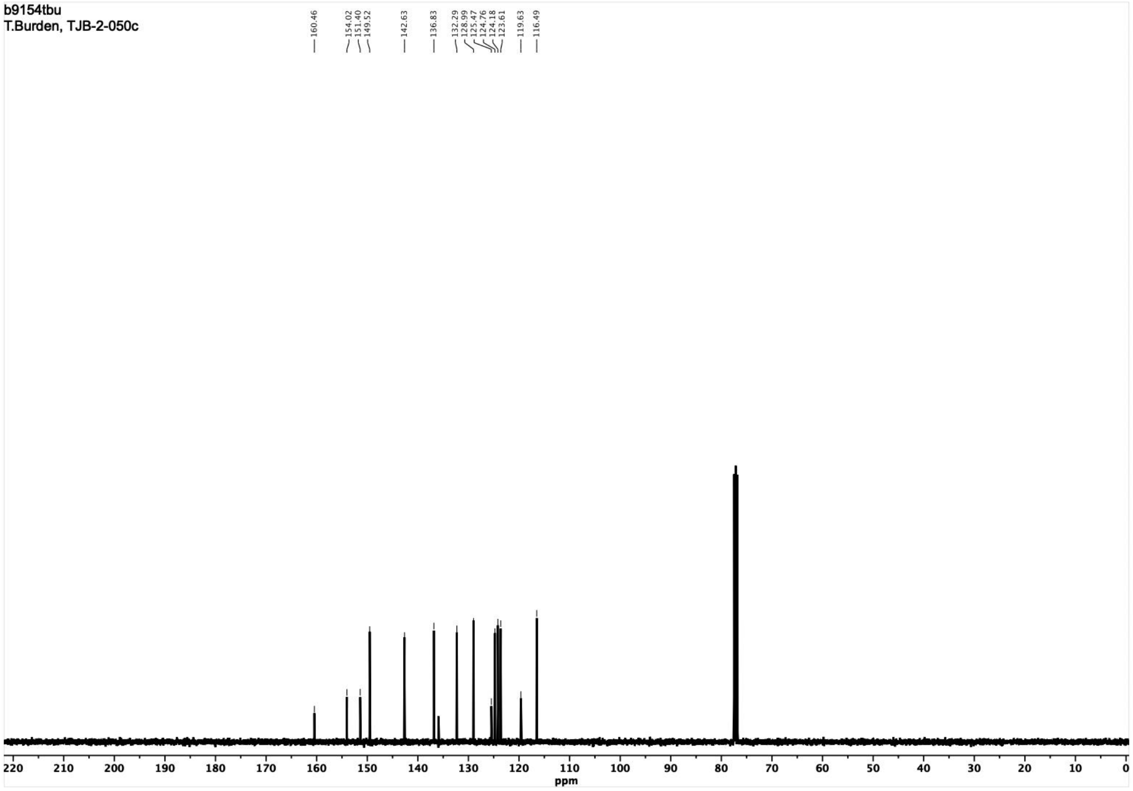

Figure S42 ^{1}H NMR spectrum of 6-(H)

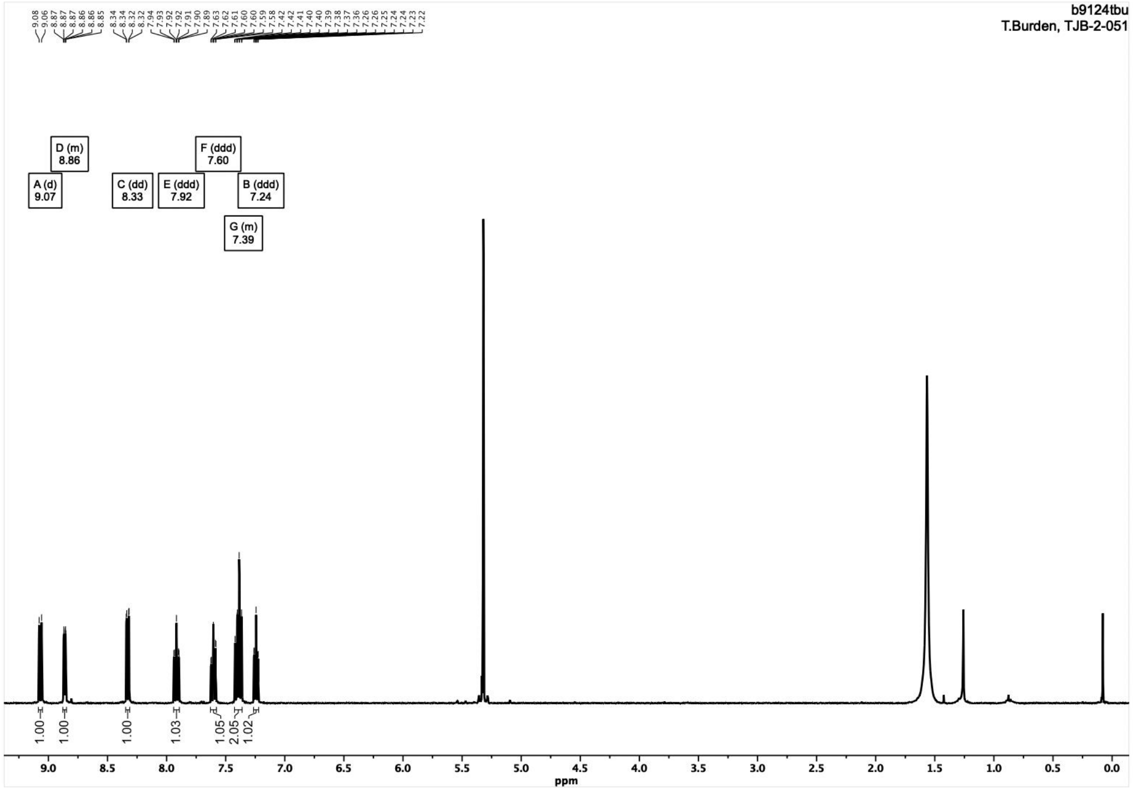

Figure S43 ^{13}C NMR spectrum of 6-(H)

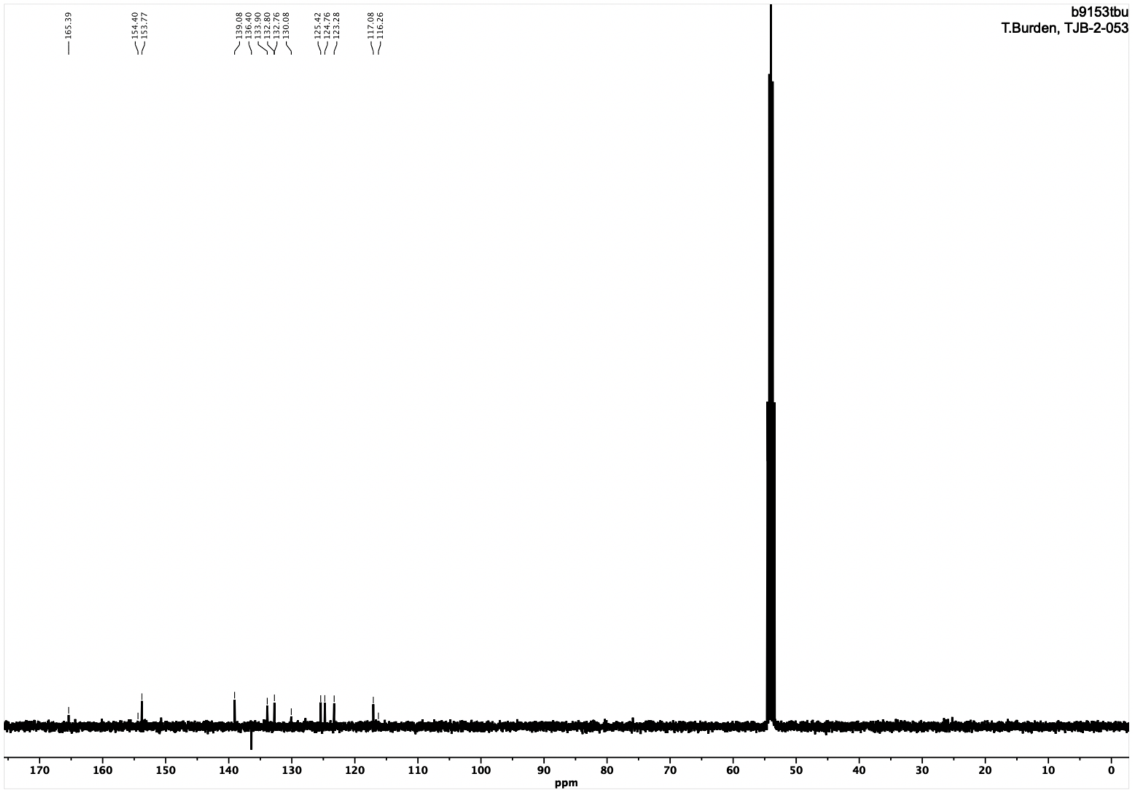

Figure S44 ^{1}H NMR spectrum of 5a-(H)

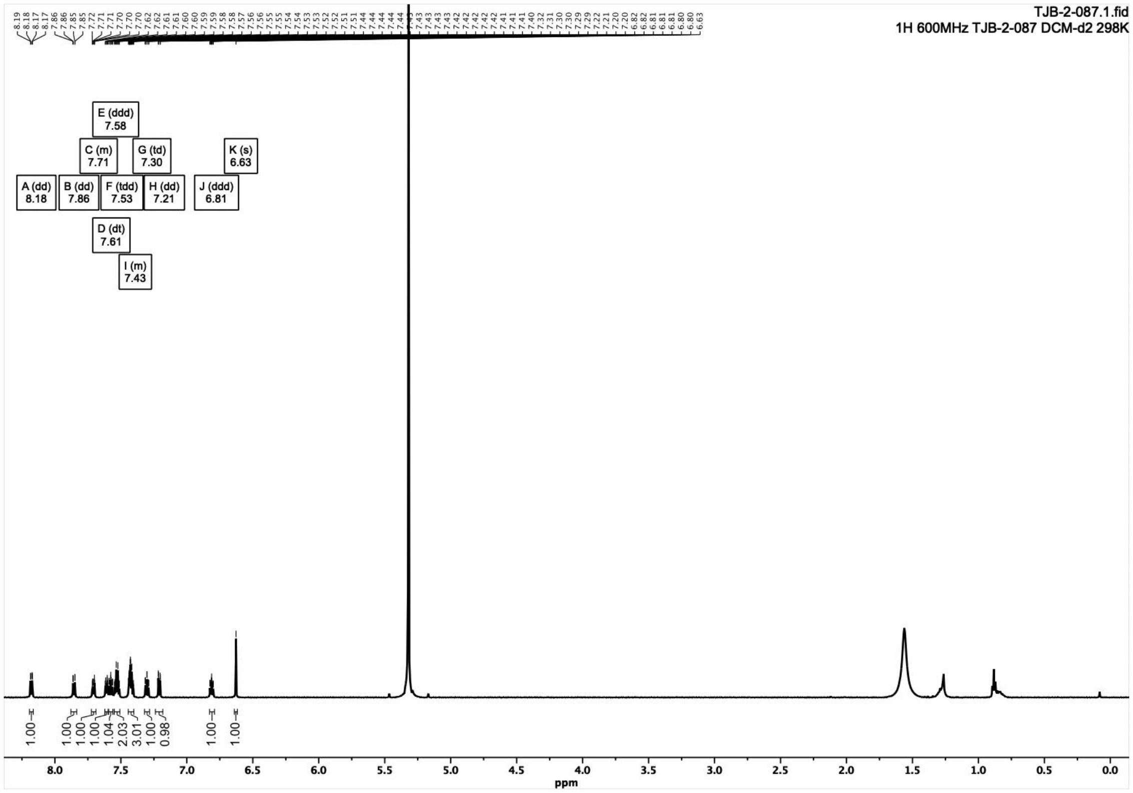

Figure S45 ^{13}C NMR spectrum of 5a-(H)

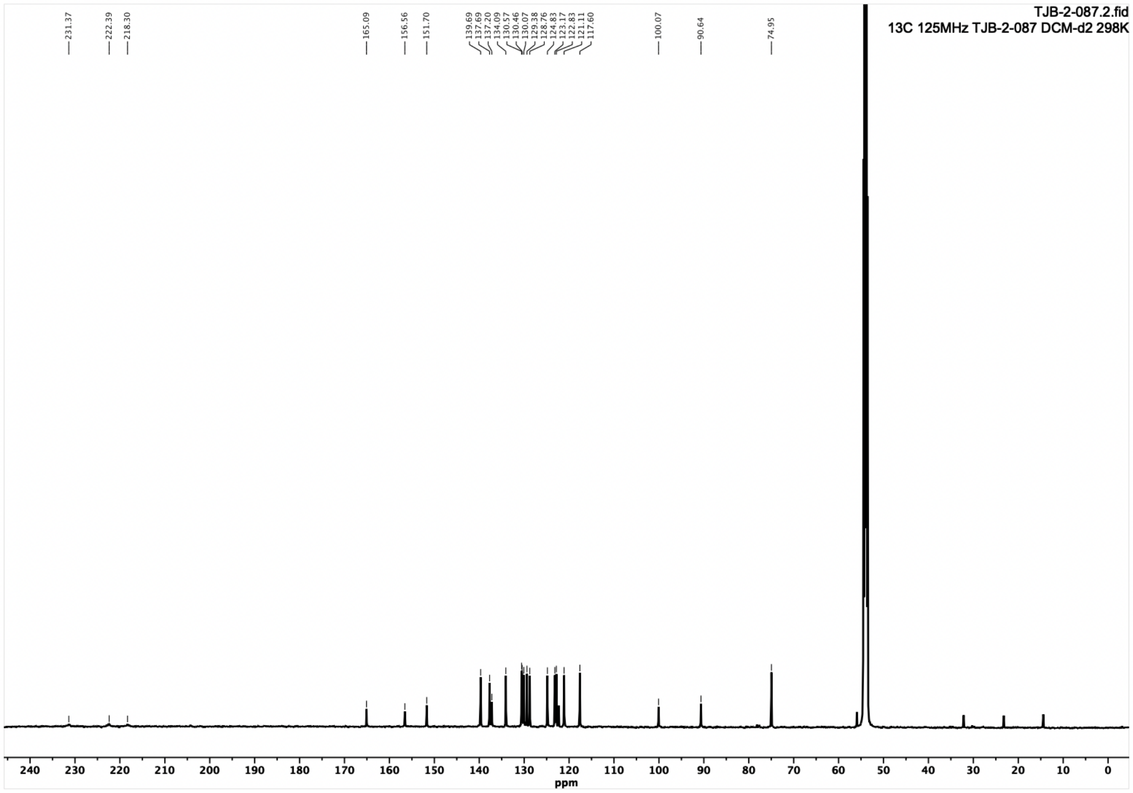

Figure S46 ^{1}H NMR spectrum of 4-(*7*-Me)

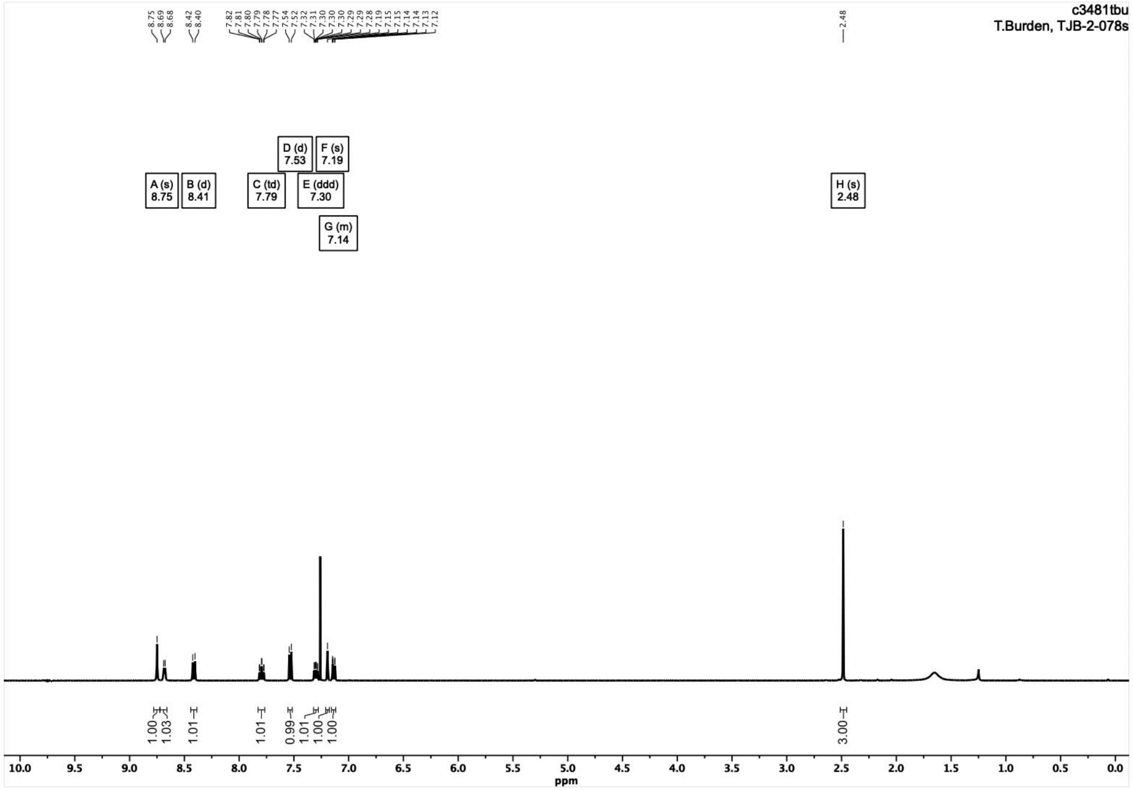

Figure S47 ^{13}C NMR spectrum of 4-(*7*-Me)

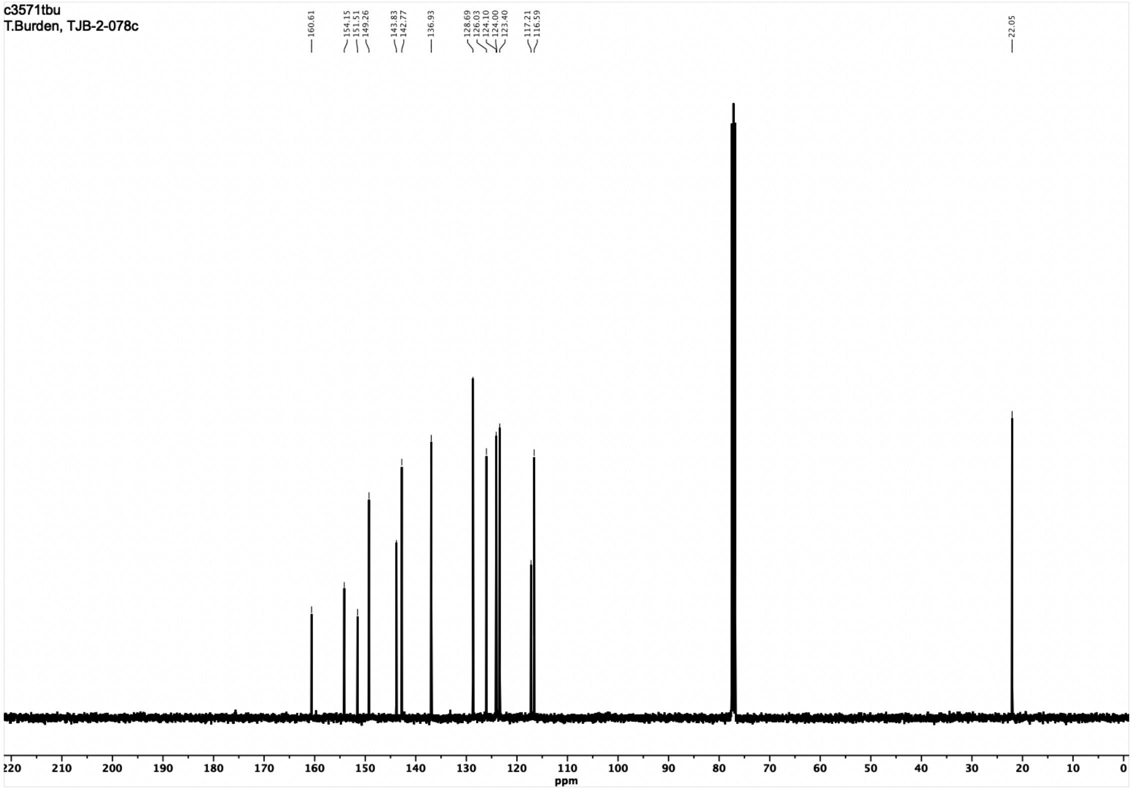

Figure S48 ^{1}H NMR spectrum of 6-(*7*-Me)

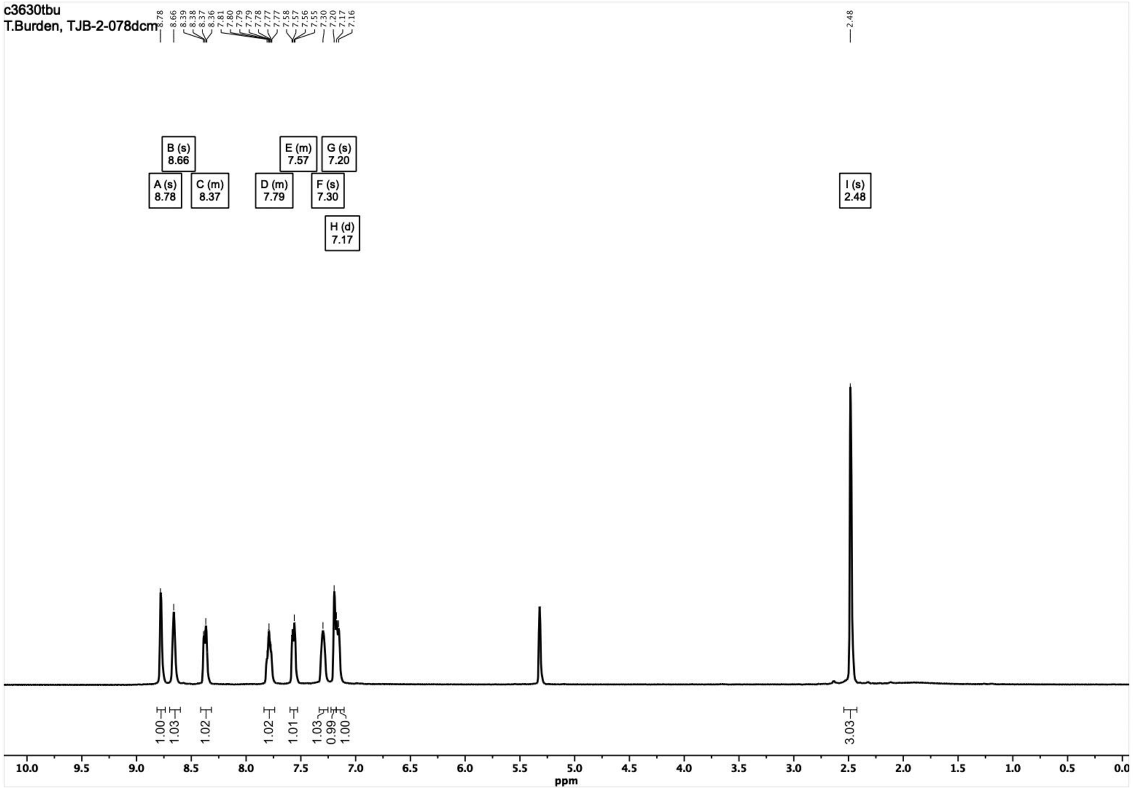

Figure S49 ^{13}C NMR spectrum of 6-(*7*-Me)

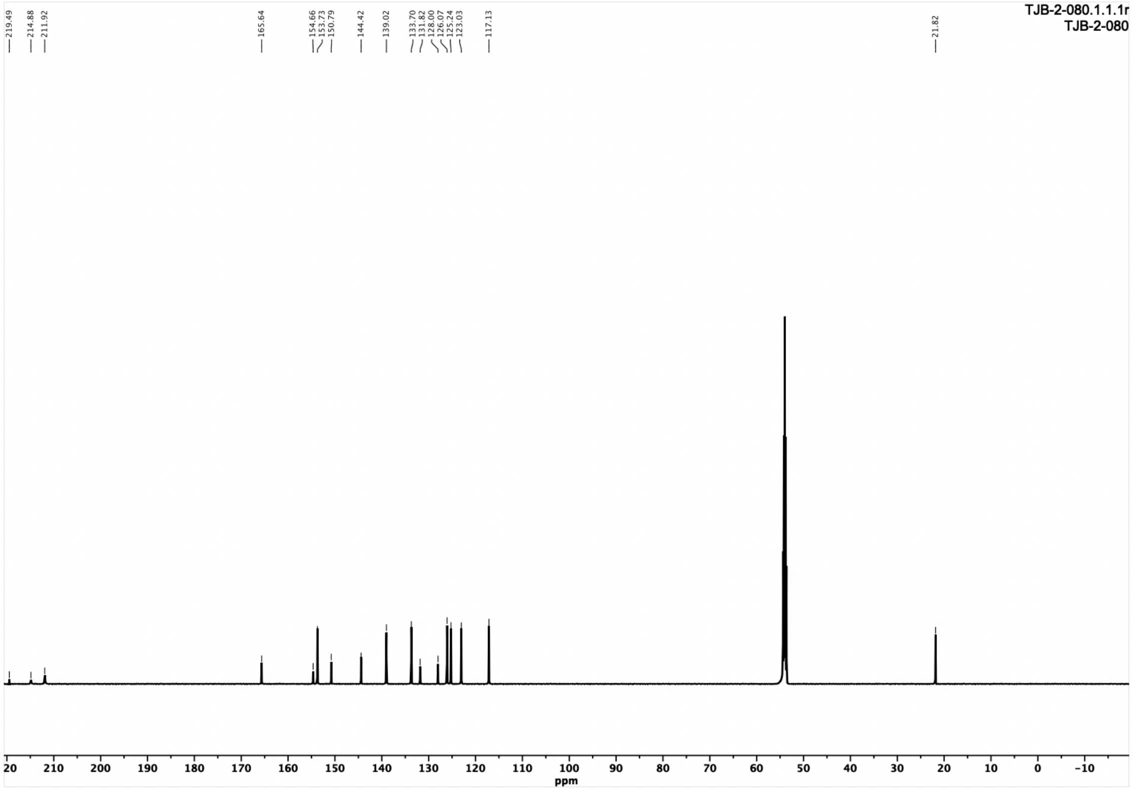

Figure S50 ^1H NMR spectrum of 5a-(*7*-Me)

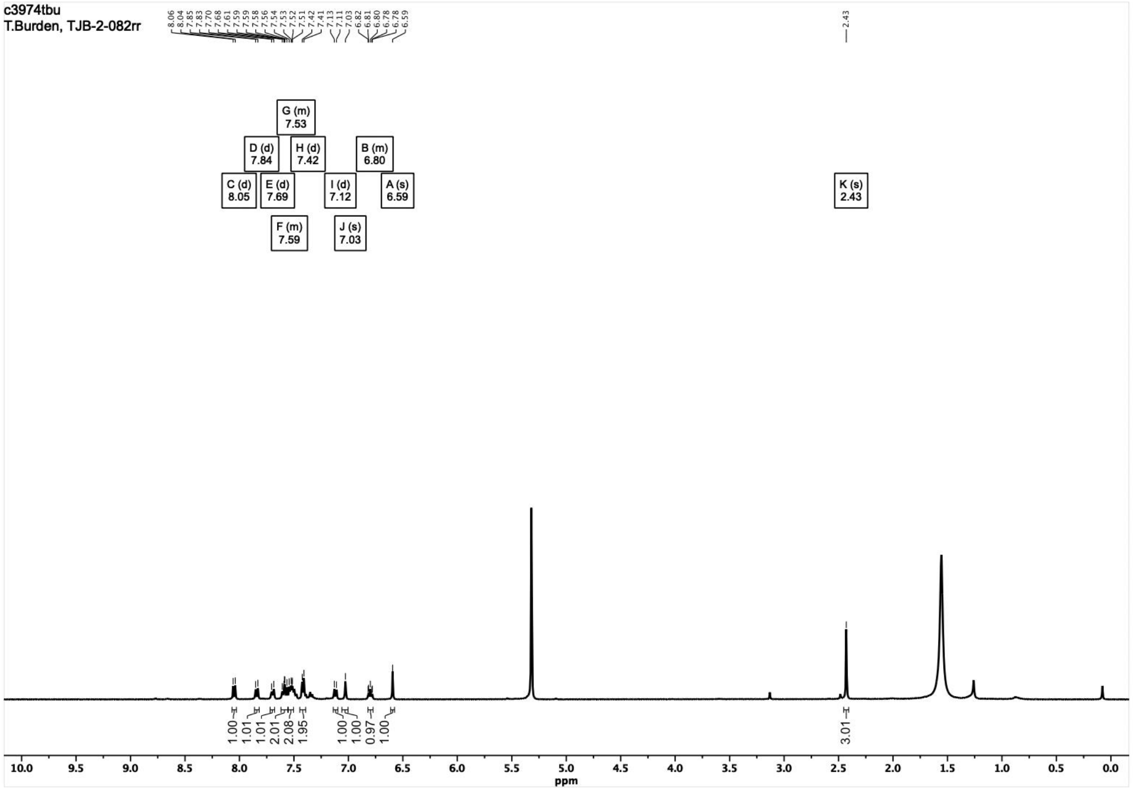

Figure S51 ^{13}C NMR spectrum of 5a-(*7*-Me)

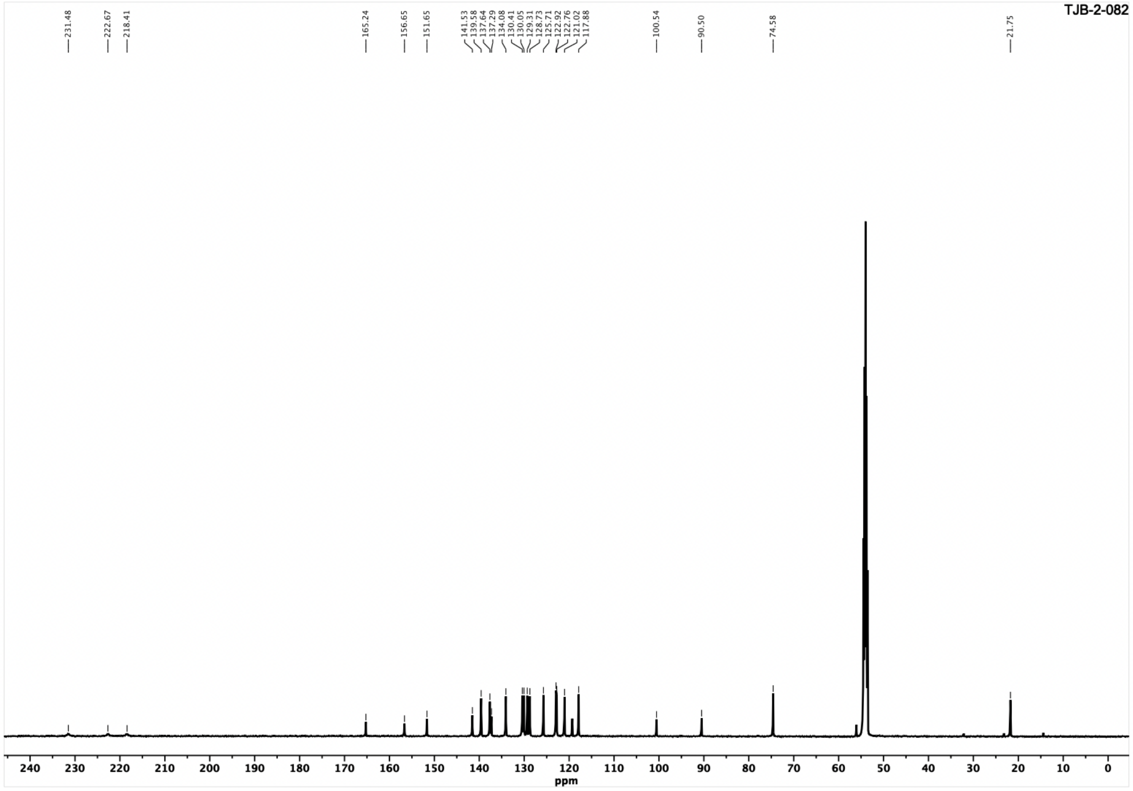

Figure S52 ^{1}H NMR spectrum of 4-(*7*-OMOM)

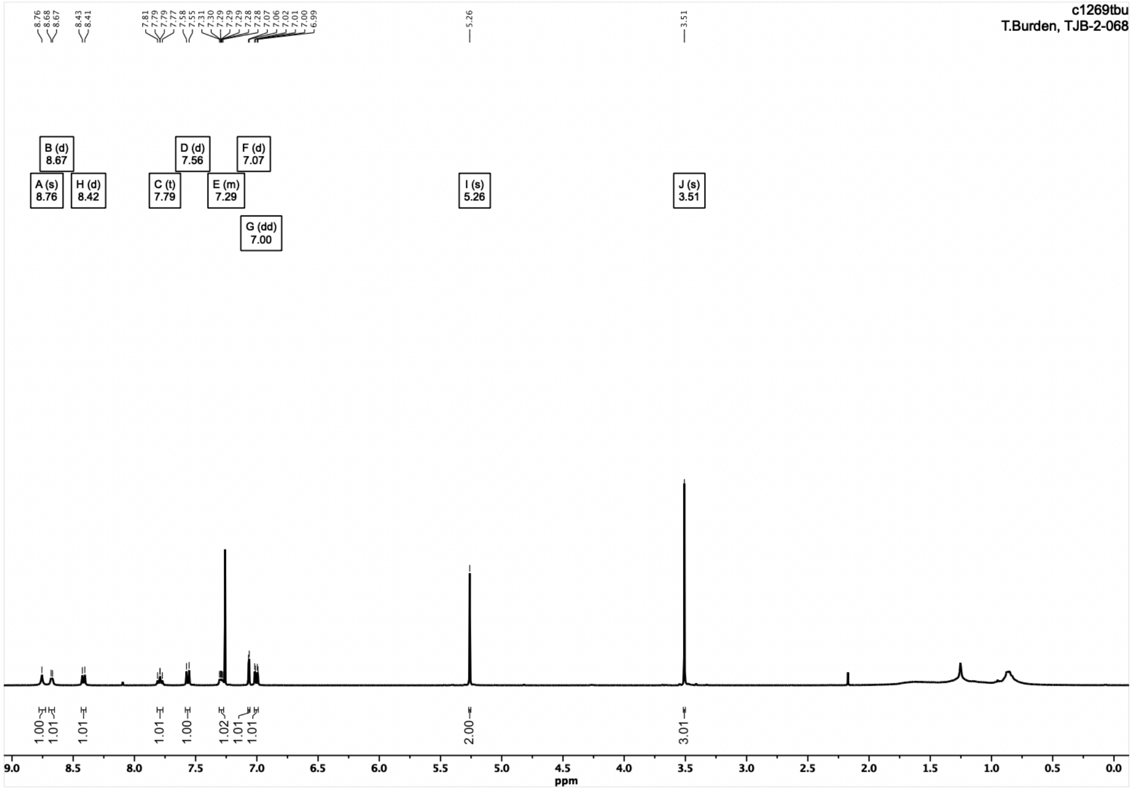

Figure S53 ^{13}C NMR spectrum of 4-(*7*-OMOM)

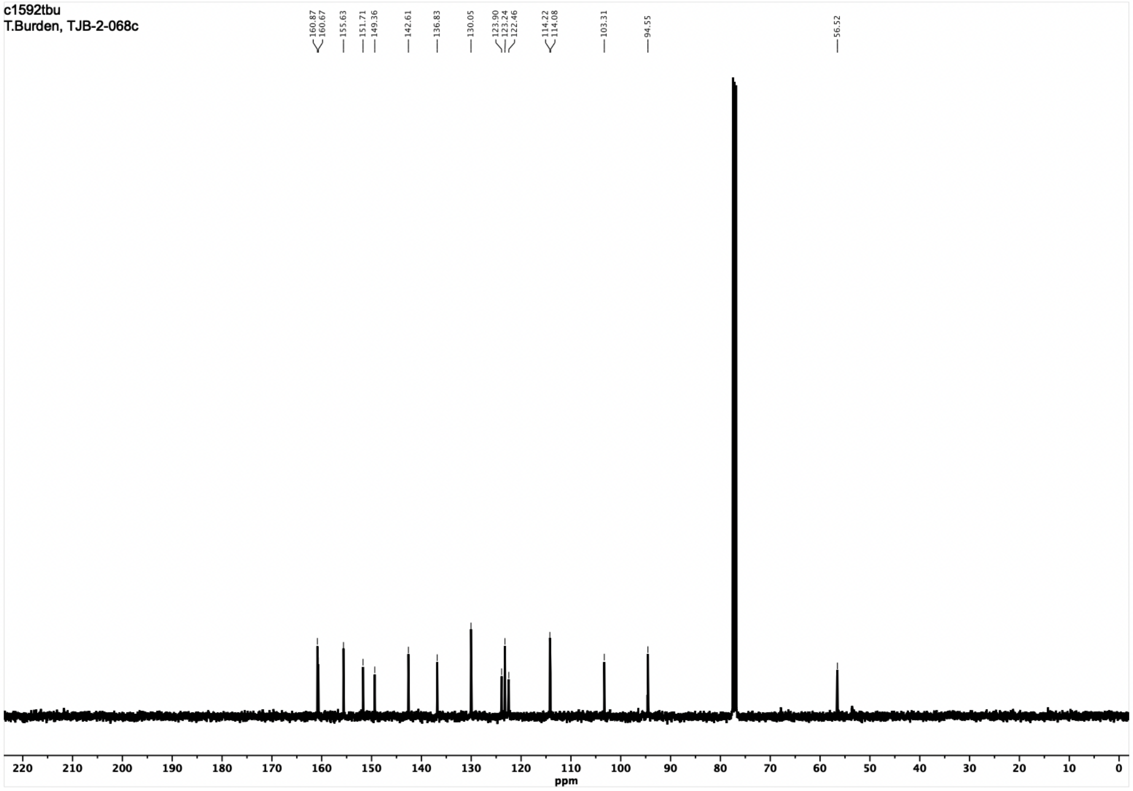

Figure S54 ^{1}H NMR spectrum of 6-(*7*-OMOM)

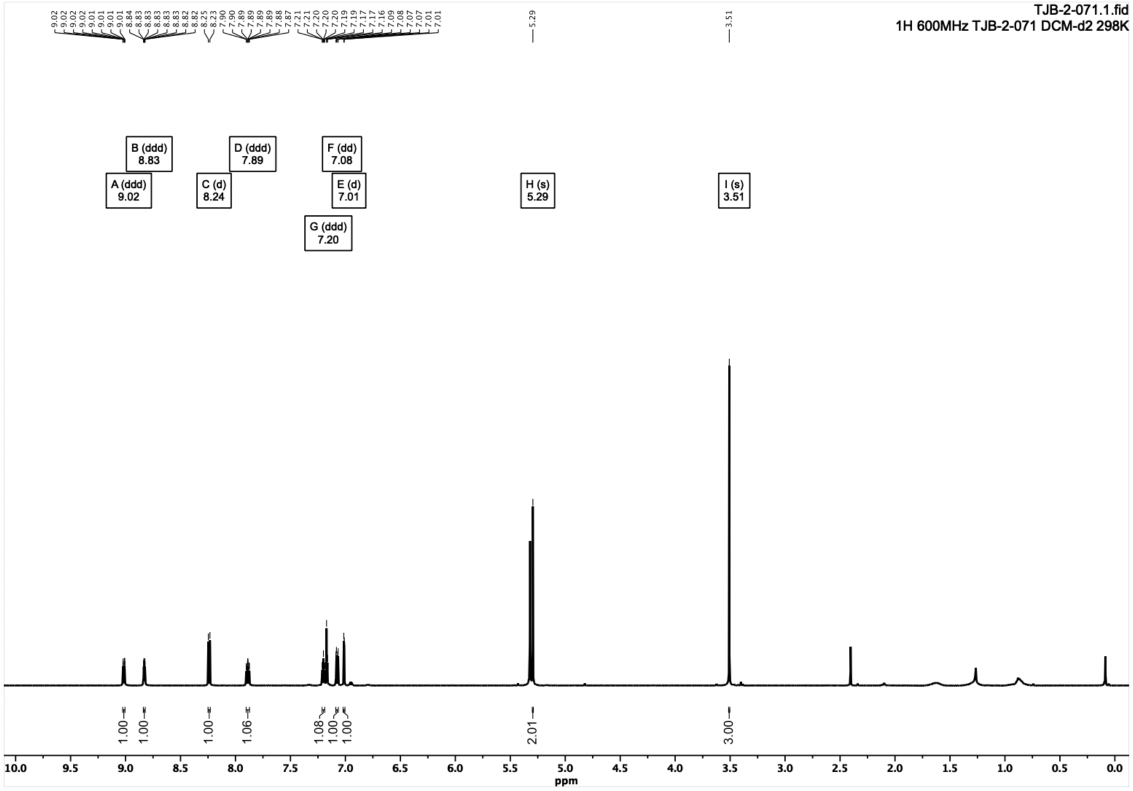

Figure S55 ^{13}C NMR spectrum of 6-(*7*-OMOM)

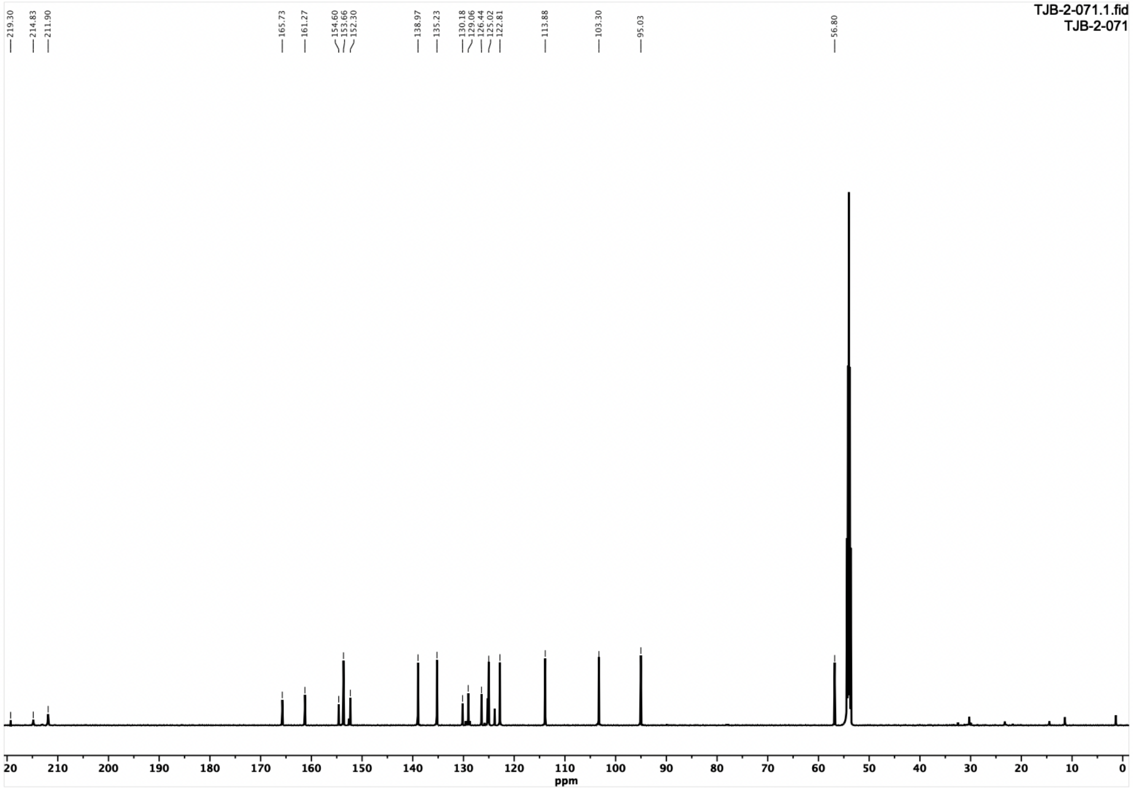

Figure S56 ^{1}H NMR spectrum of 5a-(*7*-OMOM)

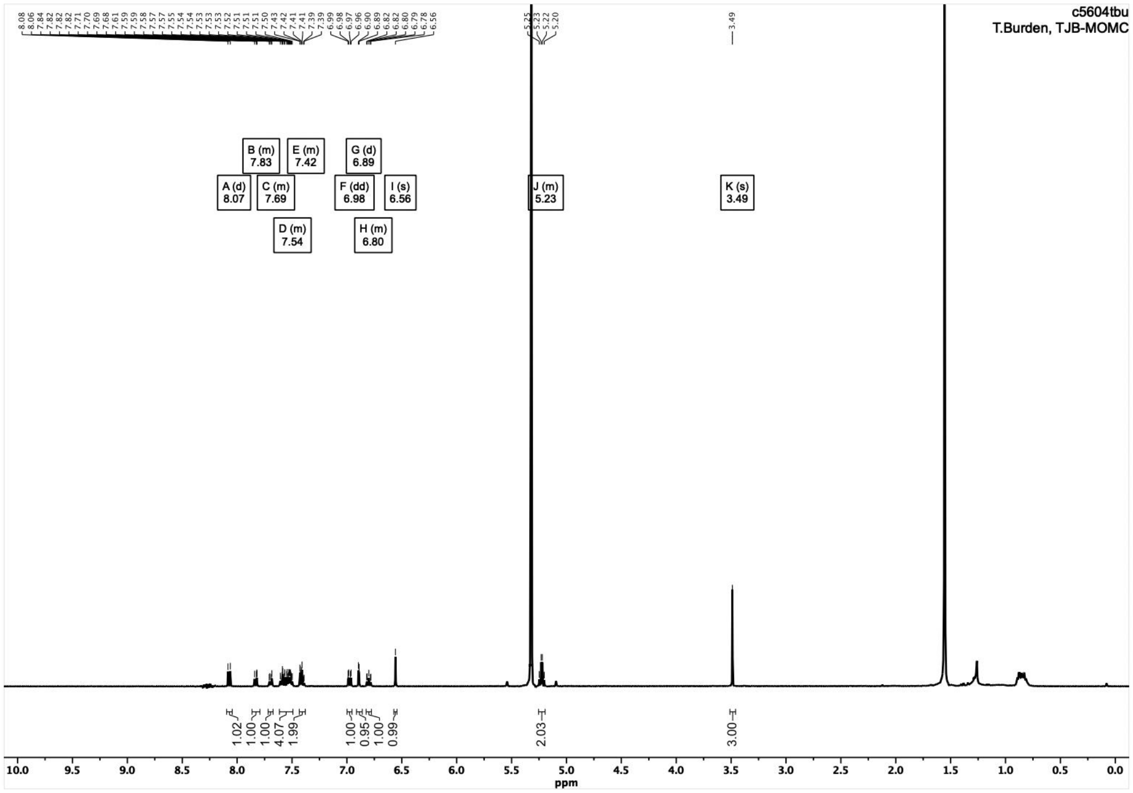

Figure S57 ^{13}C NMR spectrum of 5a-(*7*-OMOM)

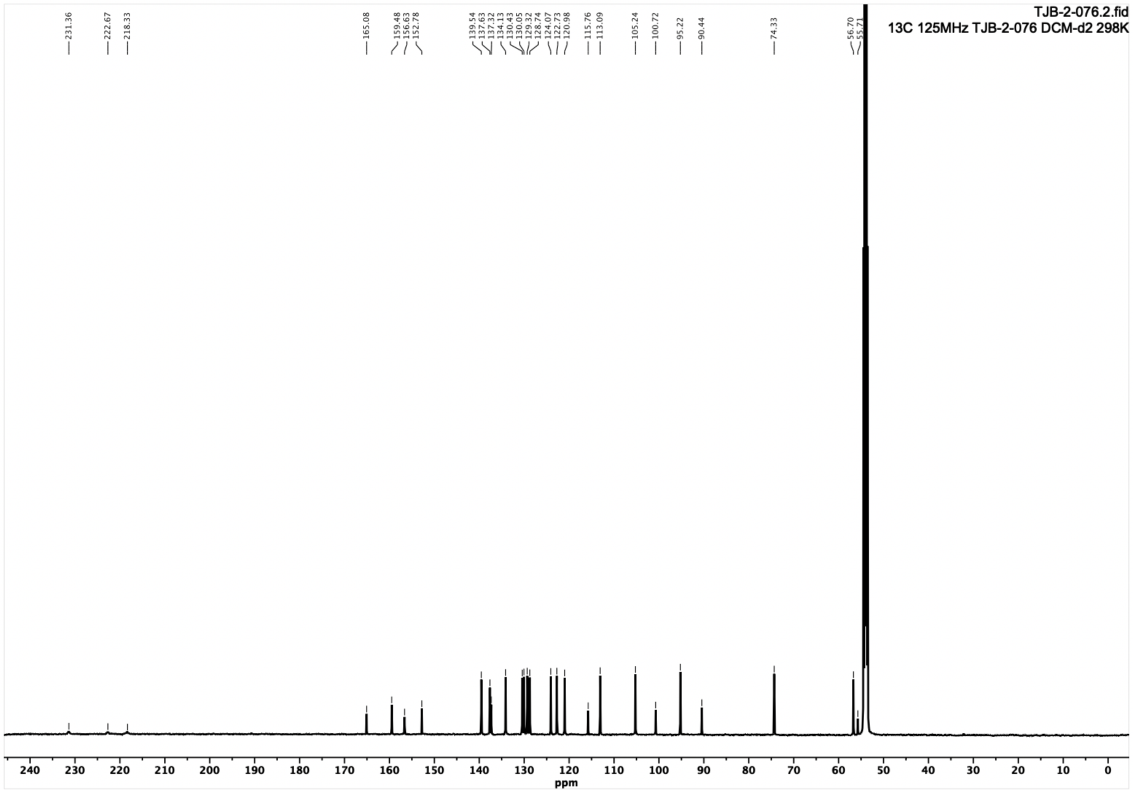

Figure S58 ^{1}H NMR spectrum of 4-(*7*-Br)

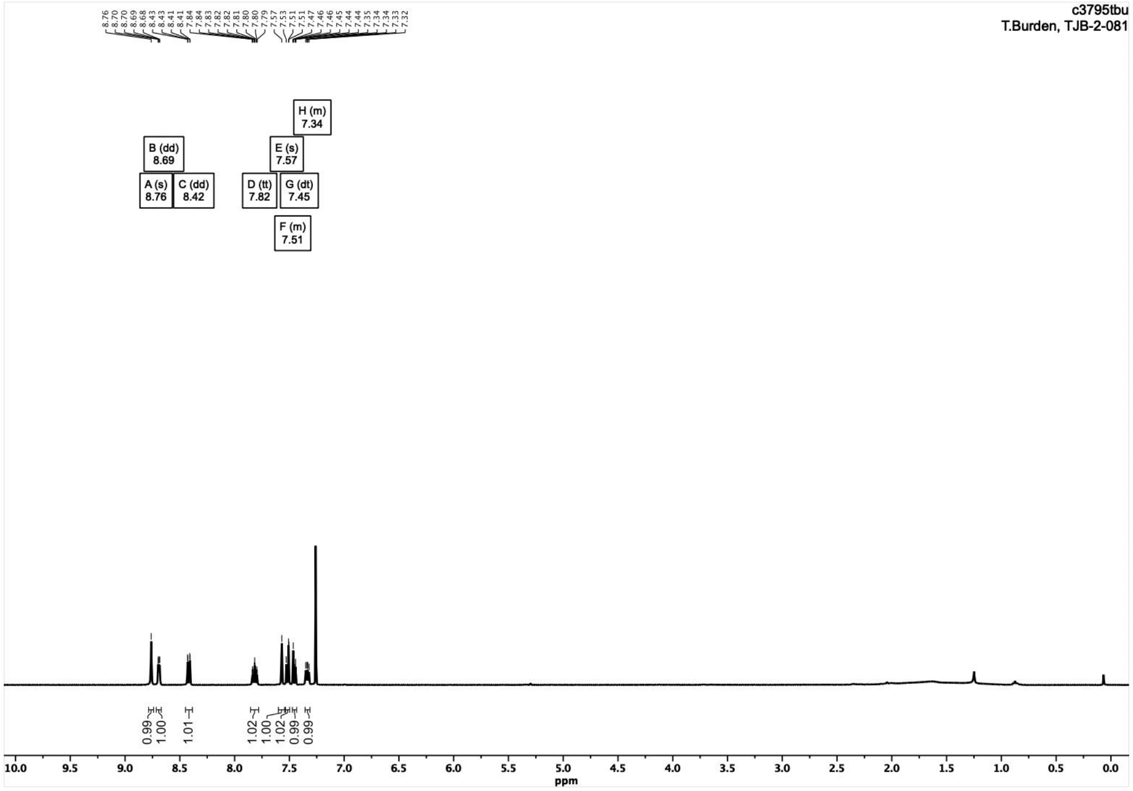

Figure S59 ^{13}C NMR spectrum of 4-(*7*-Br)

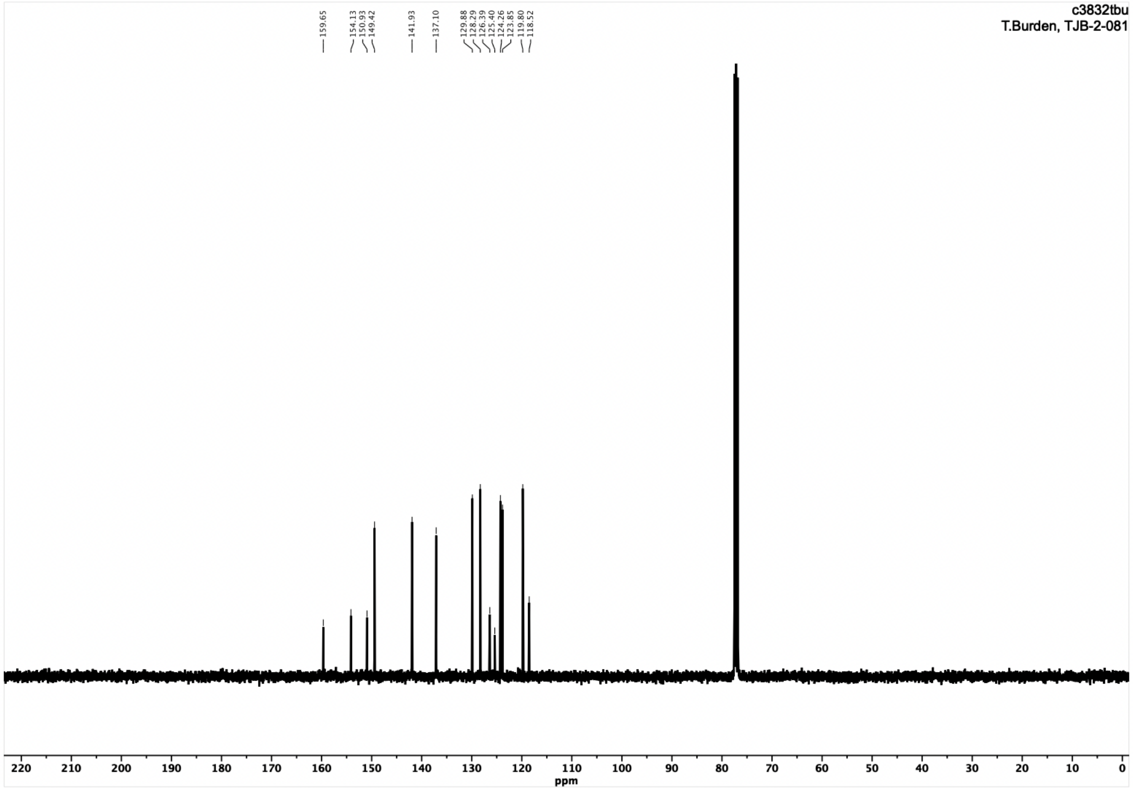

Figure S60 ^{1}H NMR spectrum of 6-(*7*-Br)

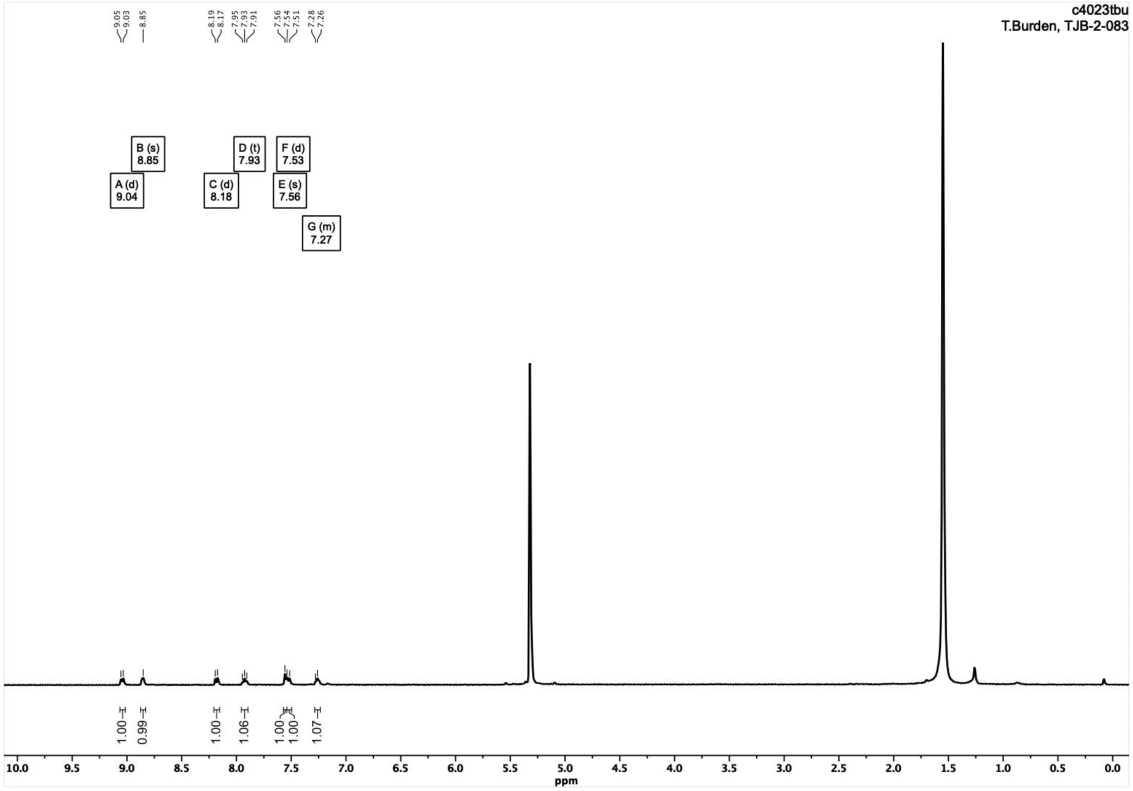

Figure S61 ^{13}C NMR spectrum of 6-(*7*-Br)

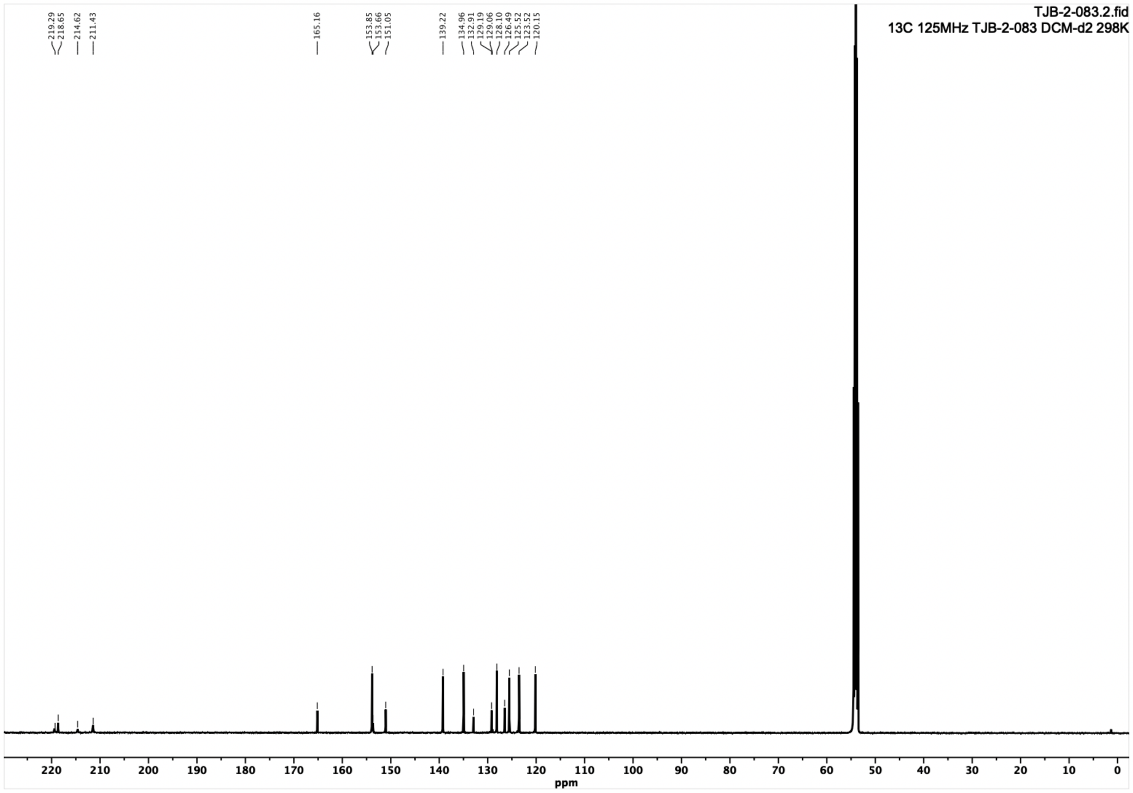

Figure S62 ^{1}H NMR spectrum of 5a-(*7*-Br)

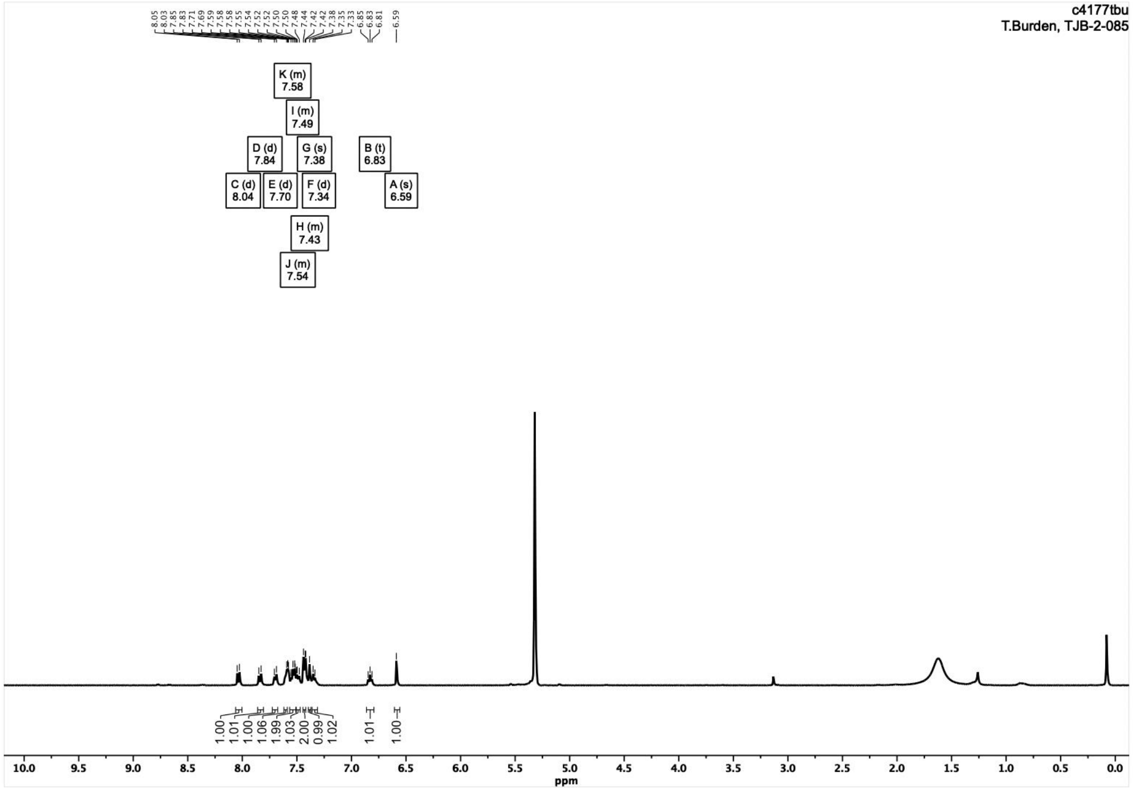

Figure S63 ^{1}H NMR spectrum of 5a-(*7*-Br)

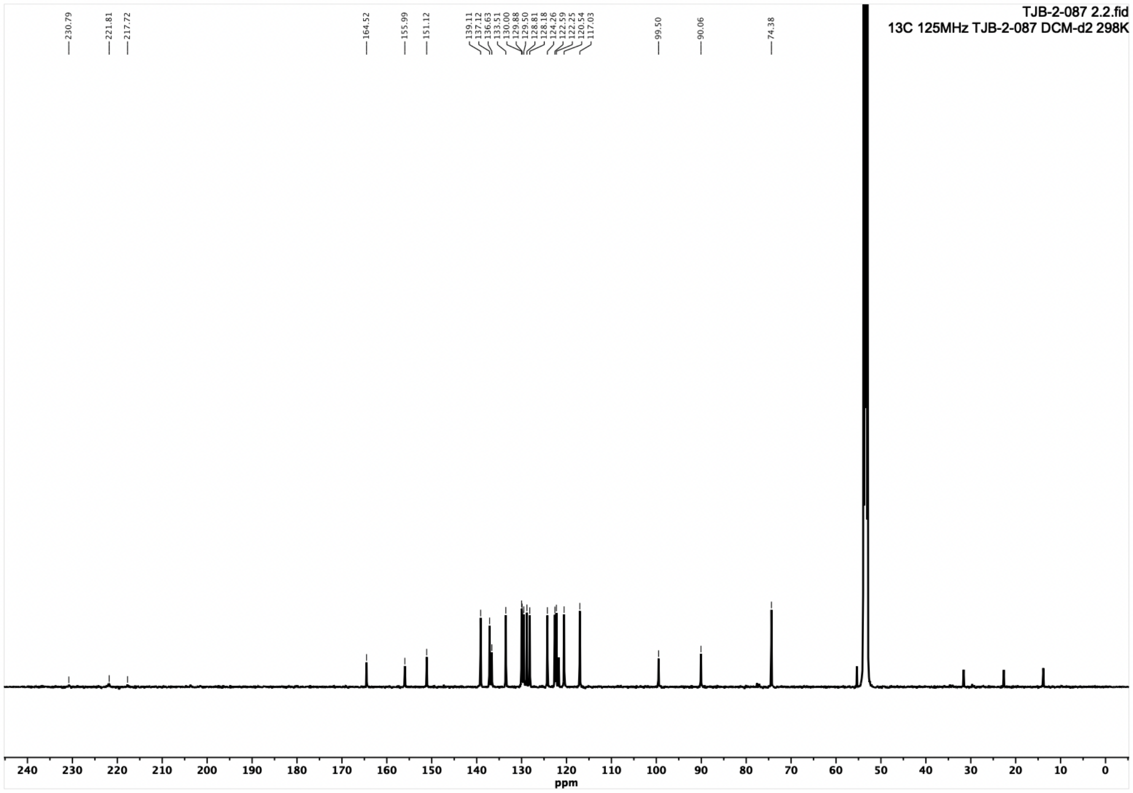

Figure S64 ^{1}H NMR spectrum of 4-(*7*-NO_2)

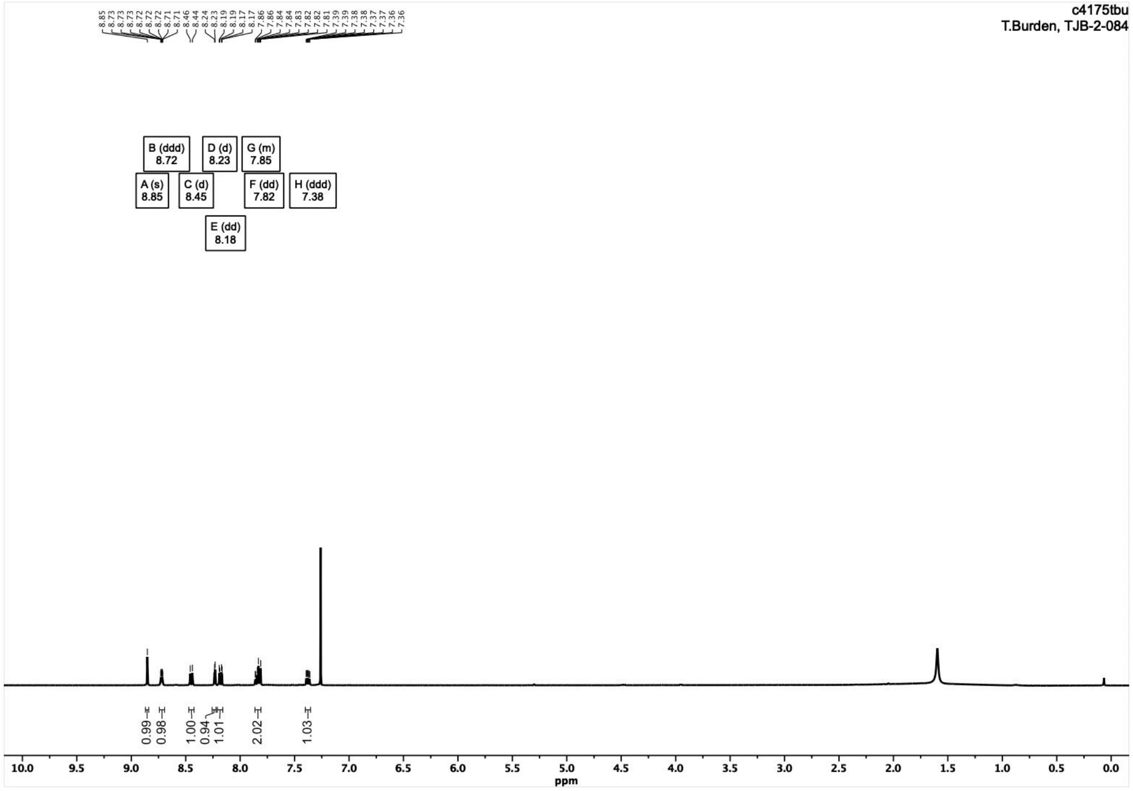

Figure S65 ^{13}C NMR spectrum of 4-(*7*-NO_2)

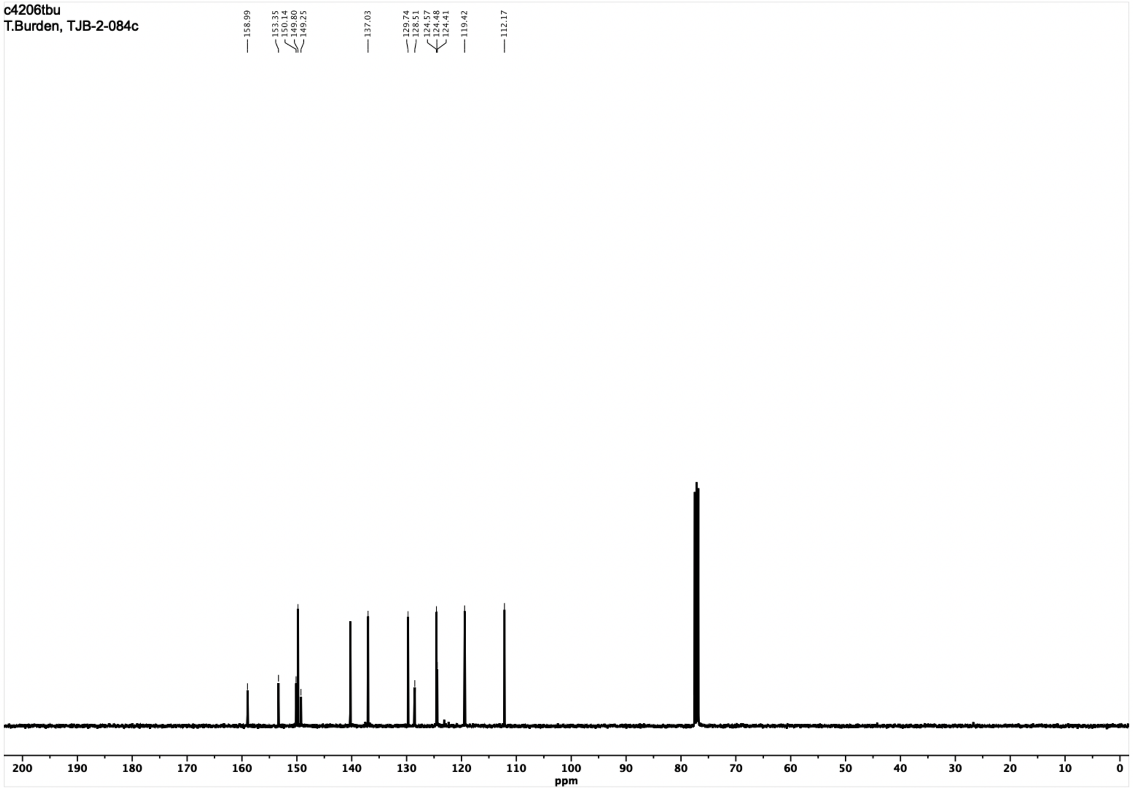

Figure S66 ^{1}H NMR spectrum of 6-(*7*-NO$_2$)

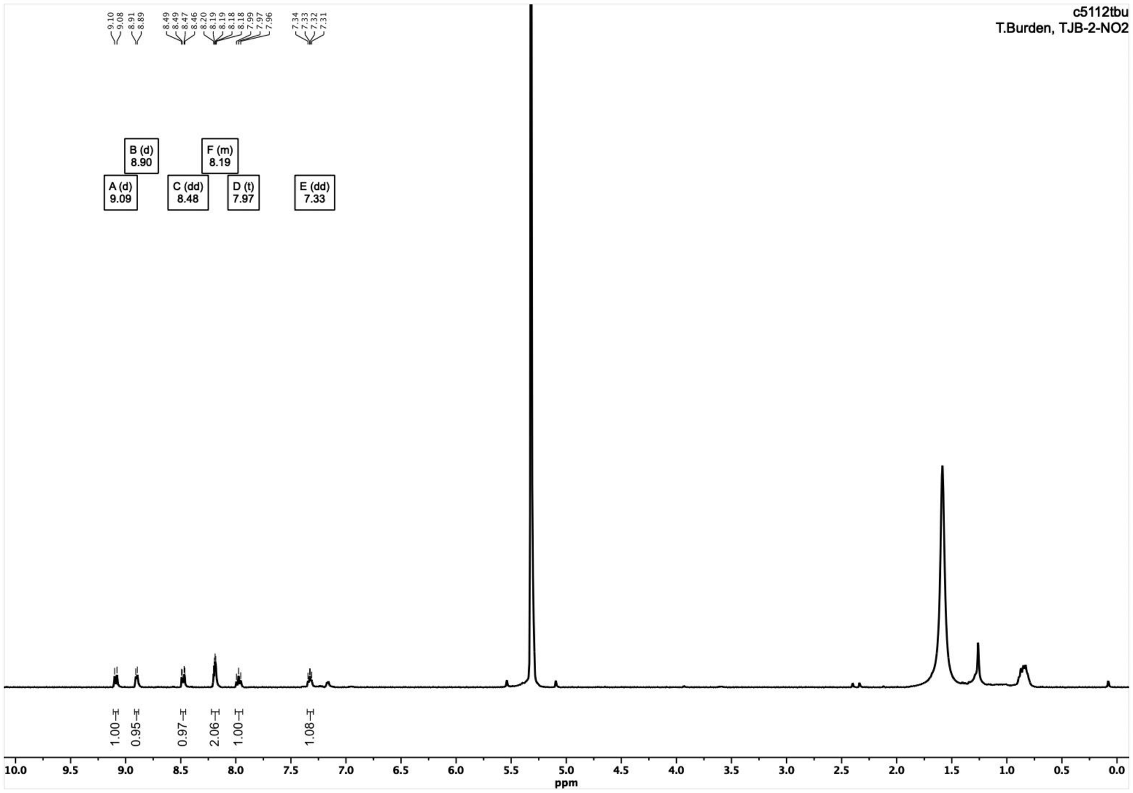

Figure S67 ^{13}C NMR spectrum of 6-(*7*-NO$_2$)

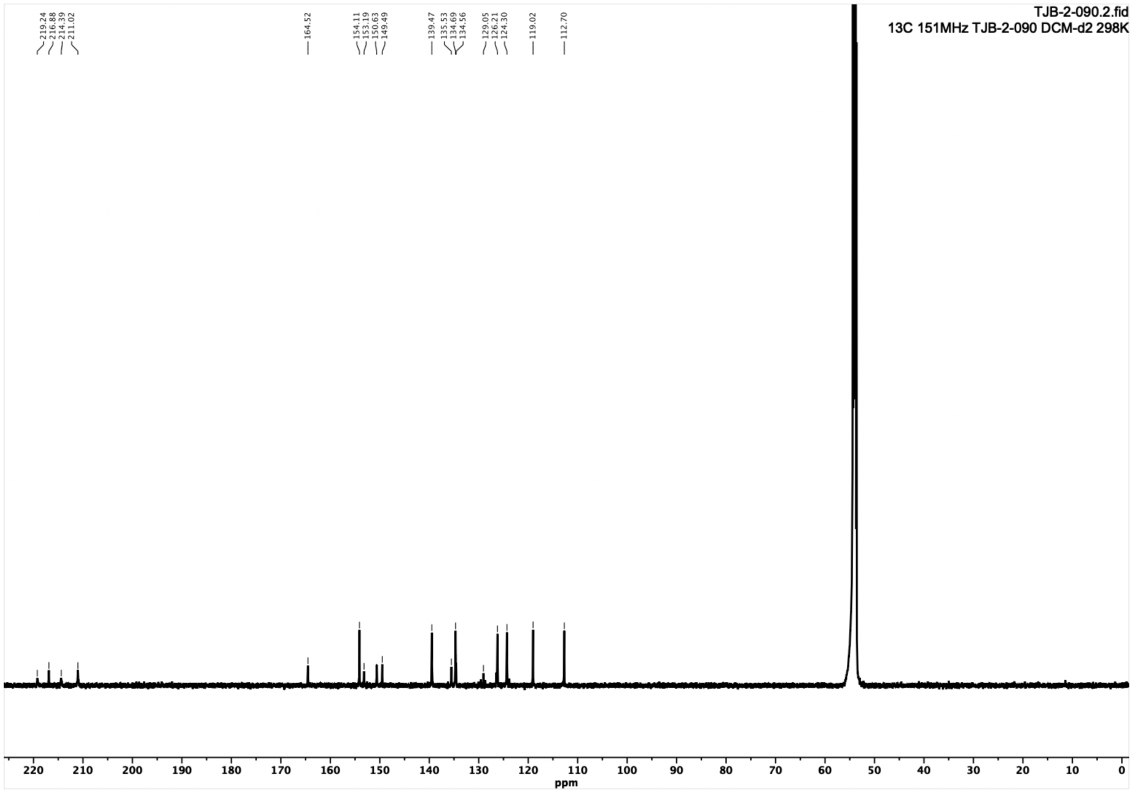

Figure S68 ^{1}H NMR spectrum of 5a-(*7*-NO_2)

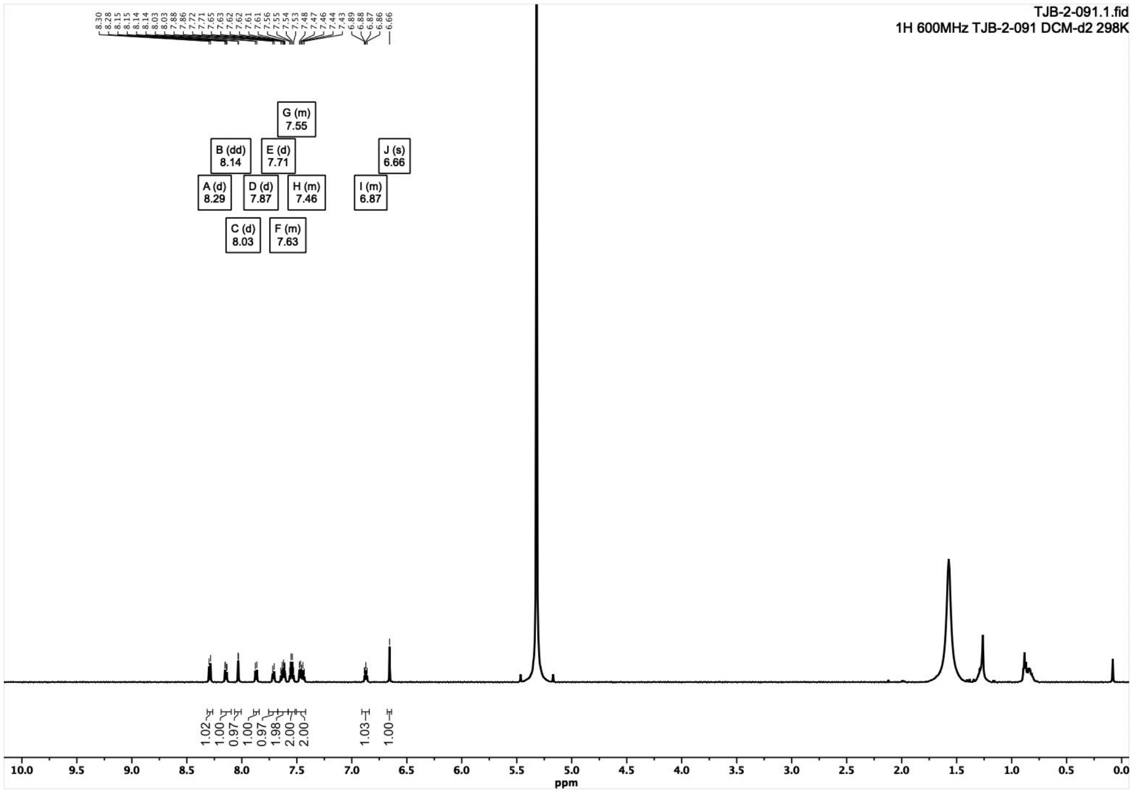

Figure S69 ^{13}C NMR spectrum of 5a-(*7*-NO_2)

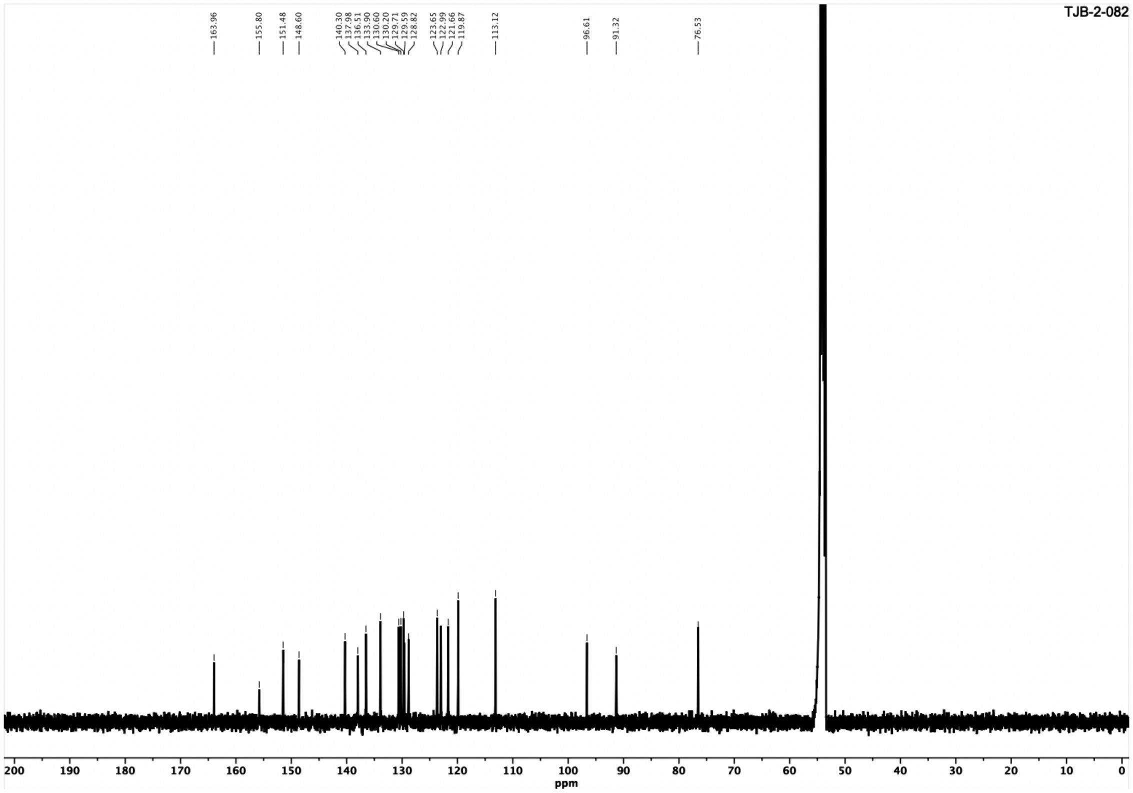

Figure S70 ^{1}H NMR spectrum of 8a

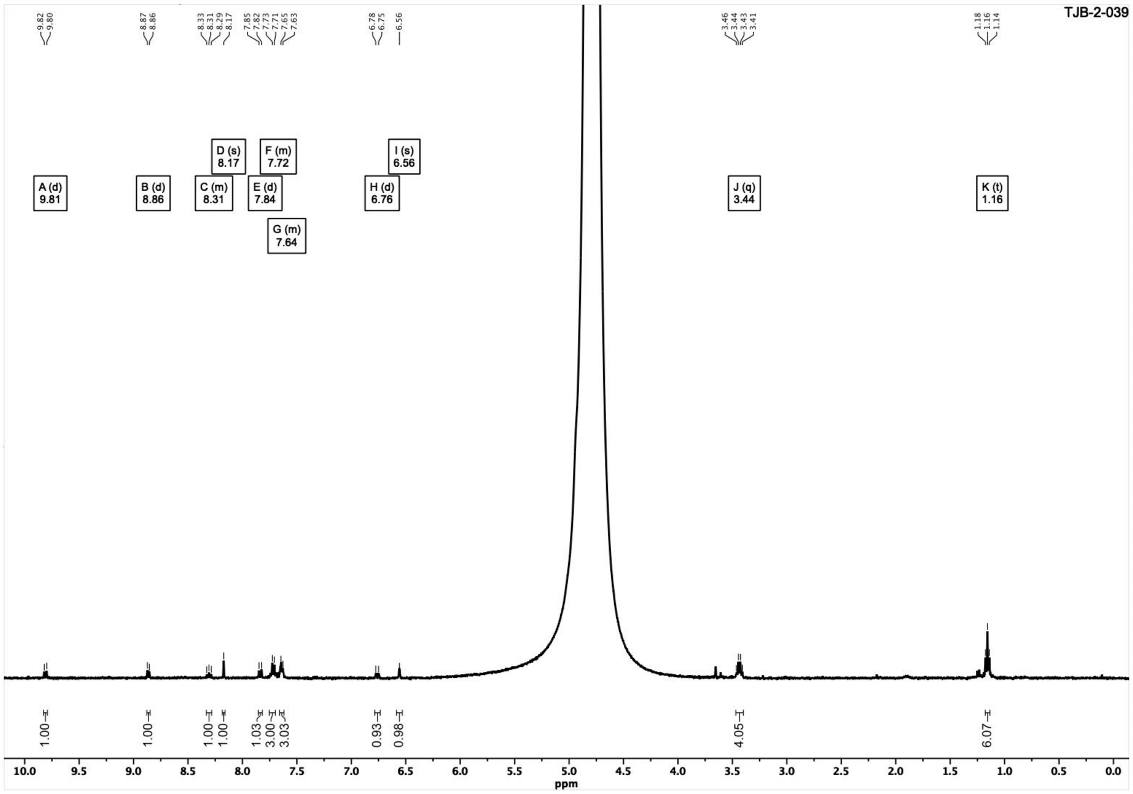

Figure S71 ^{13}C NMR spectrum of 8a

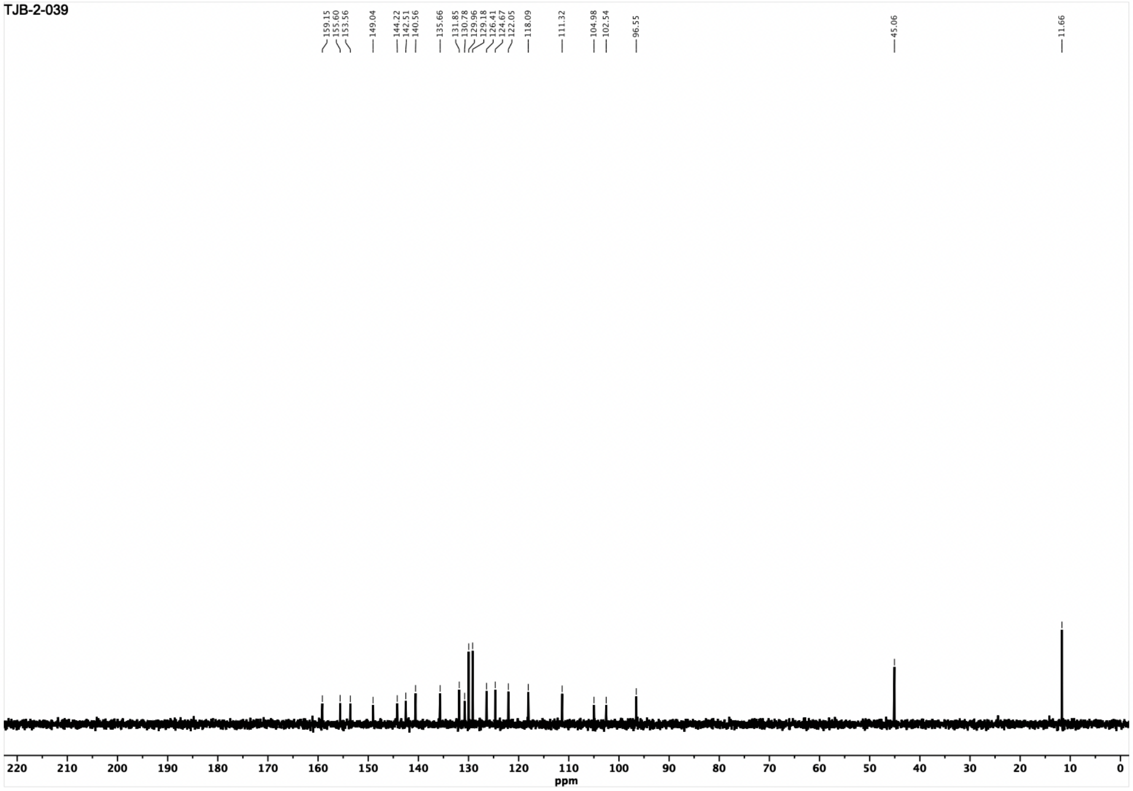

Figure S72 ^{1}H NMR spectrum of 9a

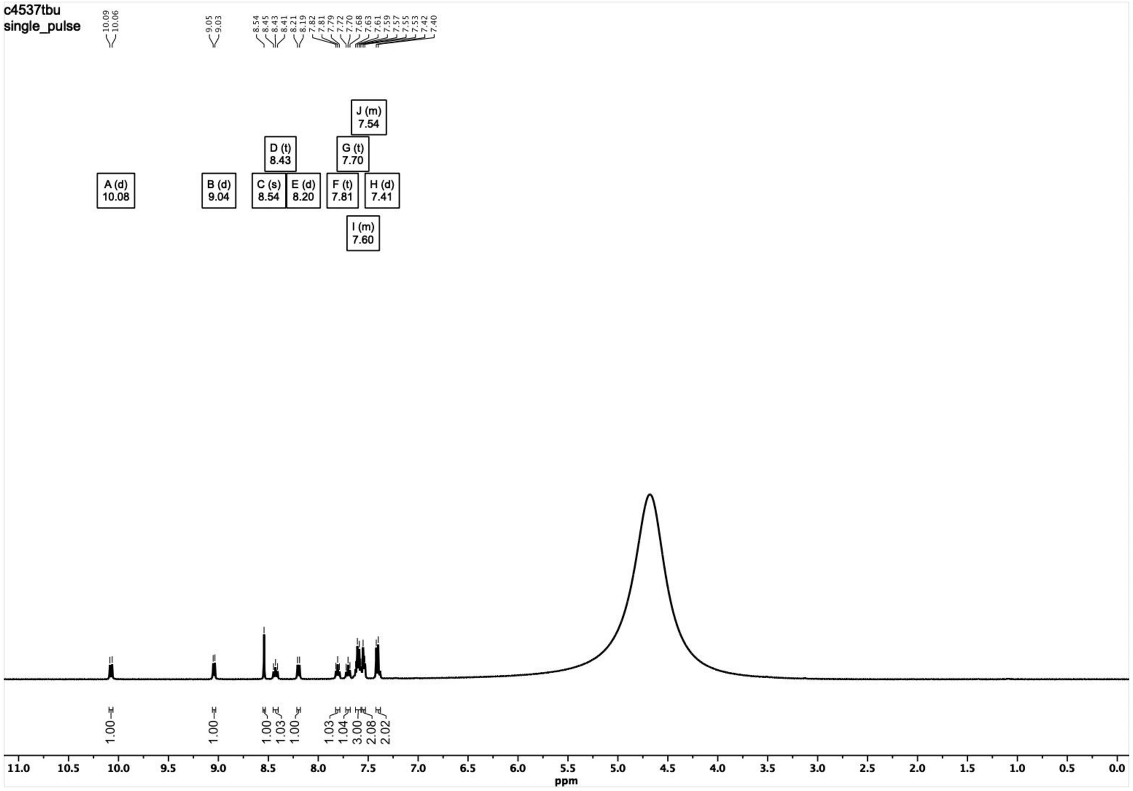

Figure S73 ^{13}C NMR spectrum of 9a

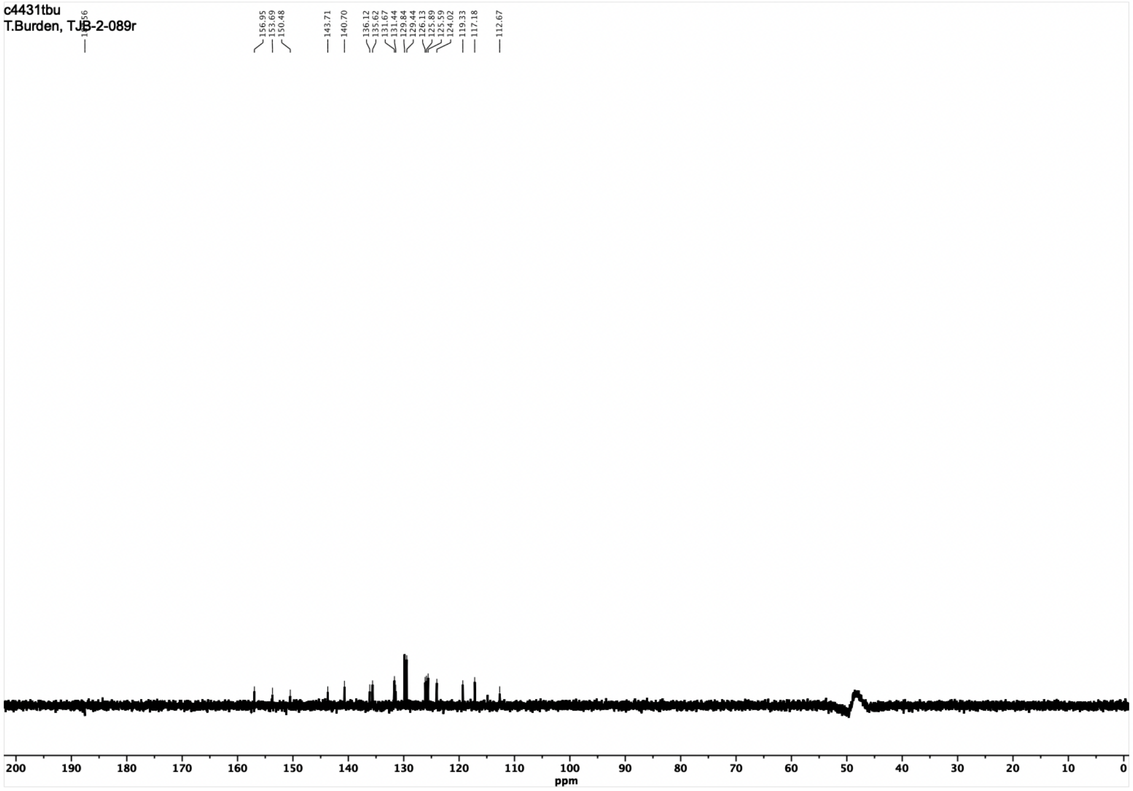

Figure S74 ^{1}H NMR spectrum of 10a

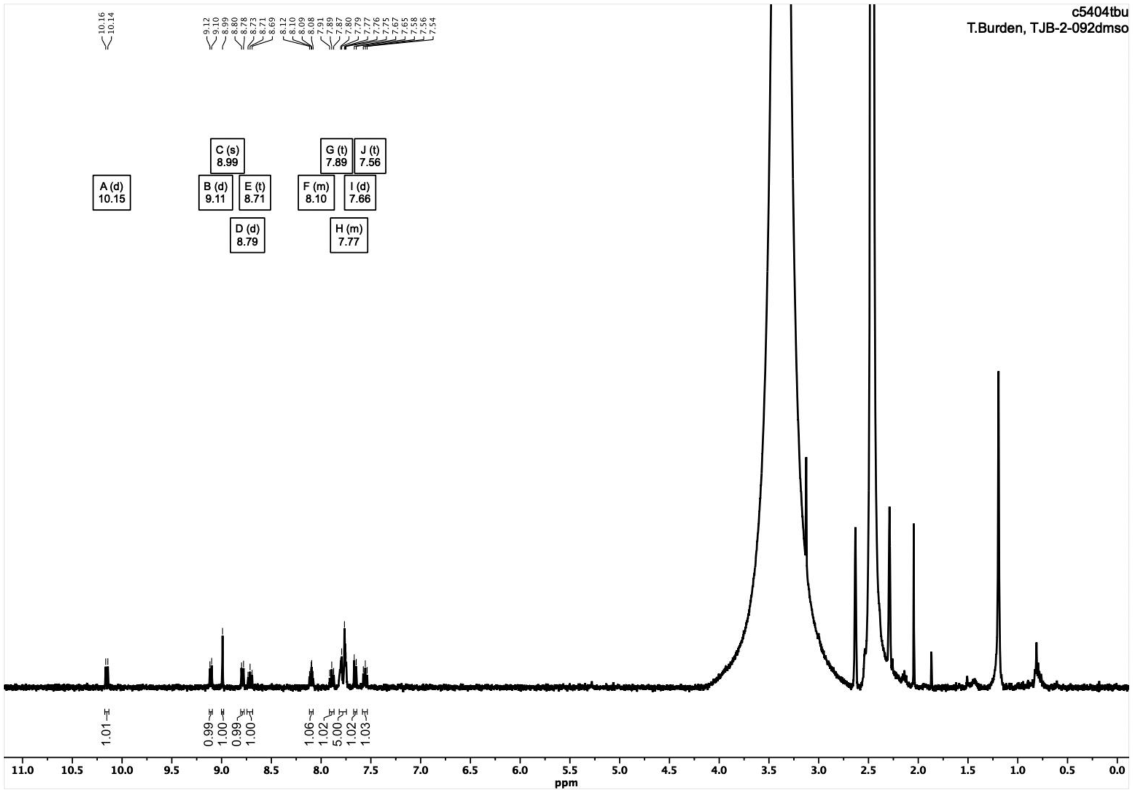

Figure S75 ^{19}F NMR spectrum of 10a

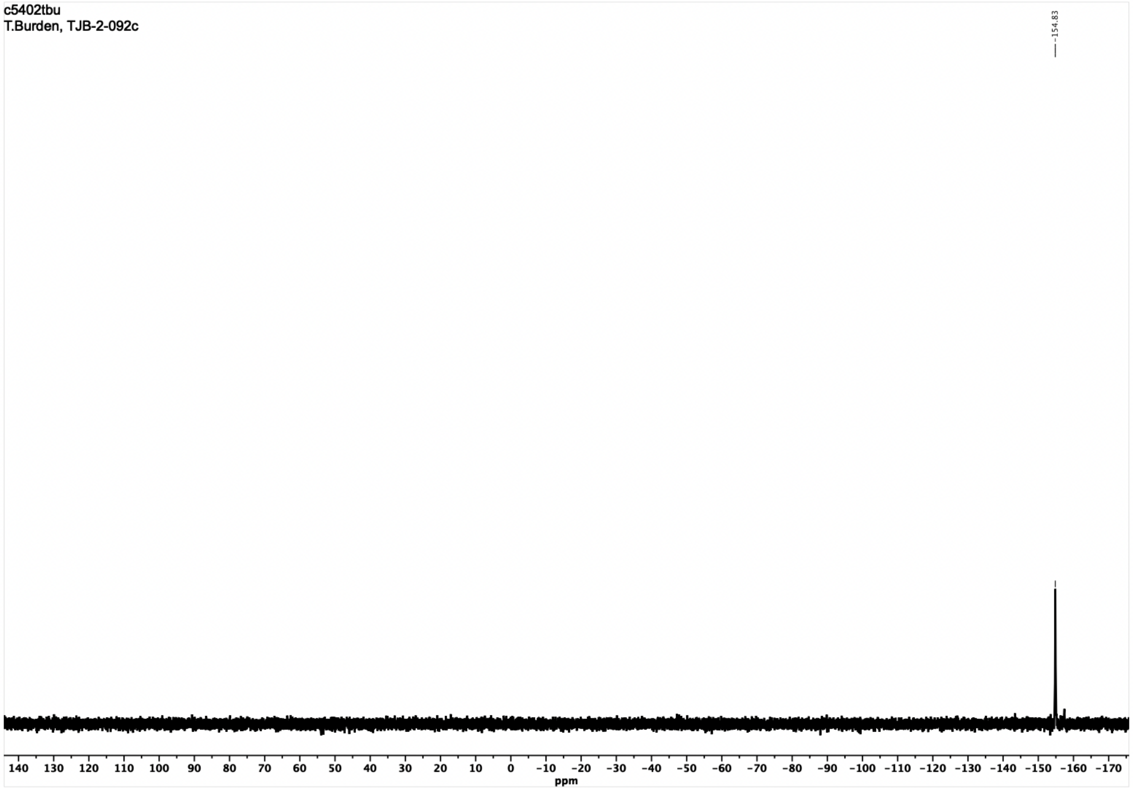

Figure S76 ^{13}C NMR spectrum of 10a

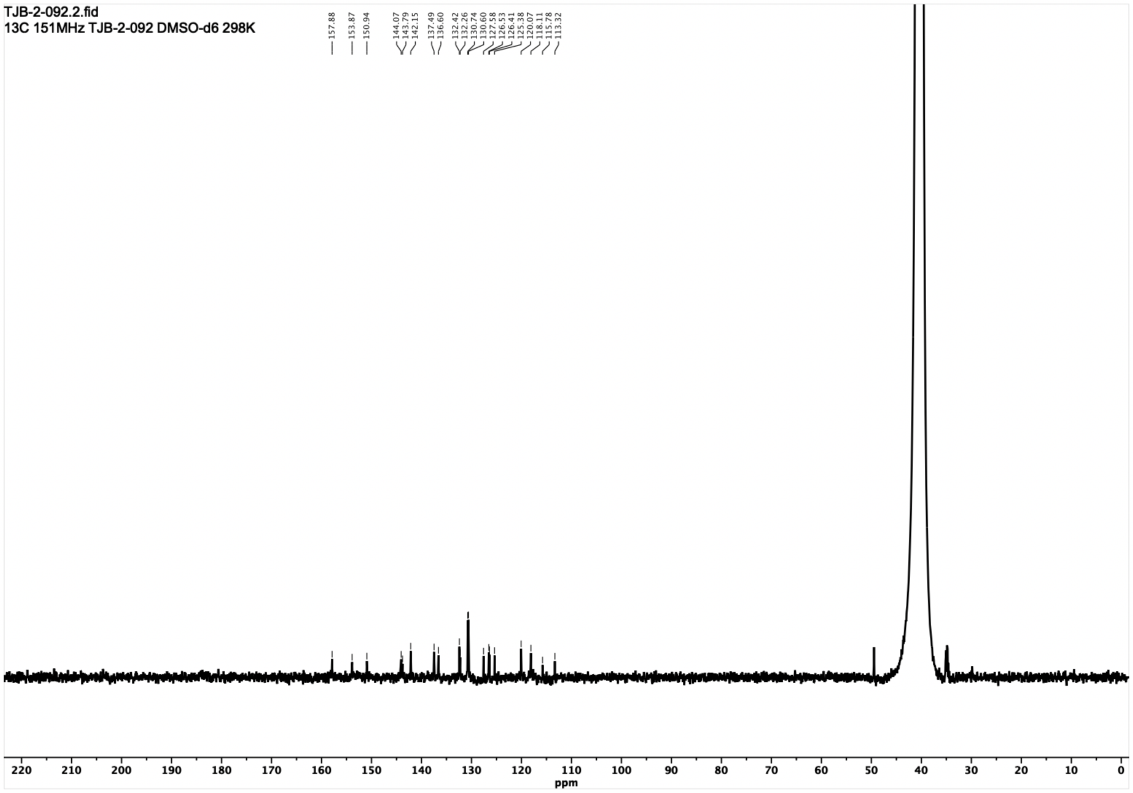

Figure S77 ^{1}H NMR spectrum of 4-(*6*-NO$_2$,*8*-OMe)

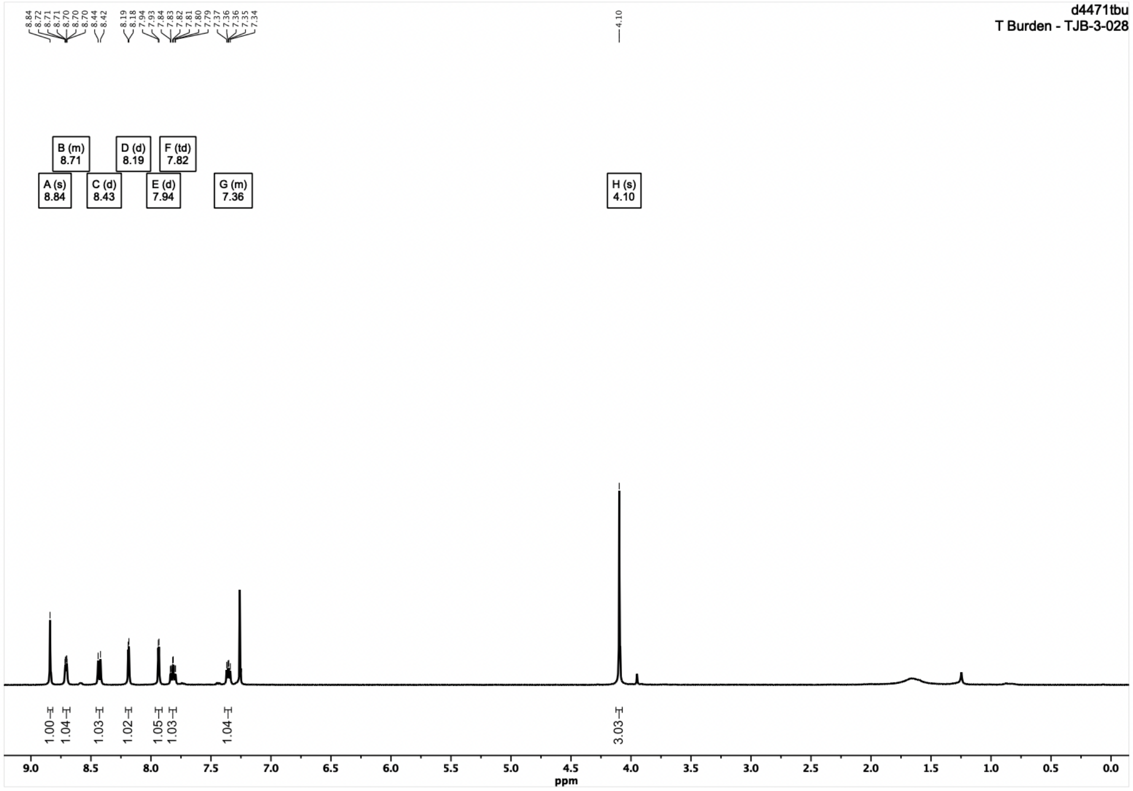

Figure S78 ^{13}C NMR spectrum of 4-(*6*-NO$_2$,*8*-OMe)

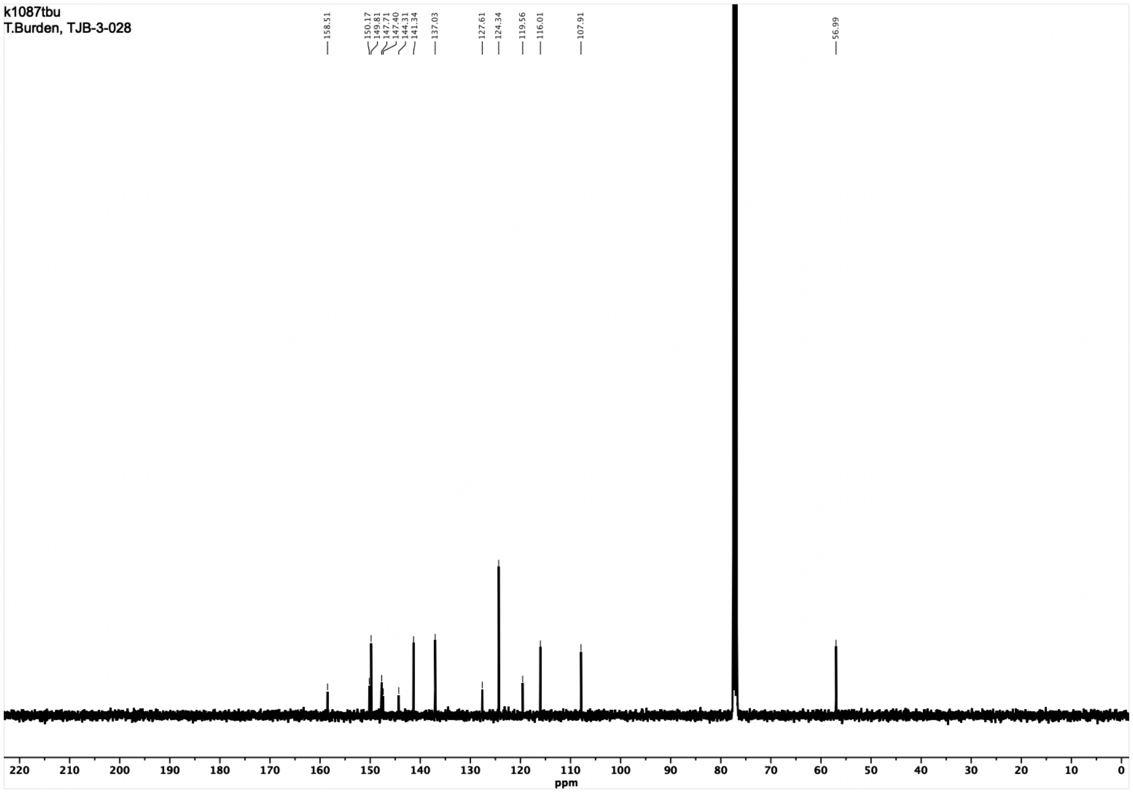

Figure S79 ^{1}H NMR spectrum of 6-(*6*-NO_2,*8*-OMe)

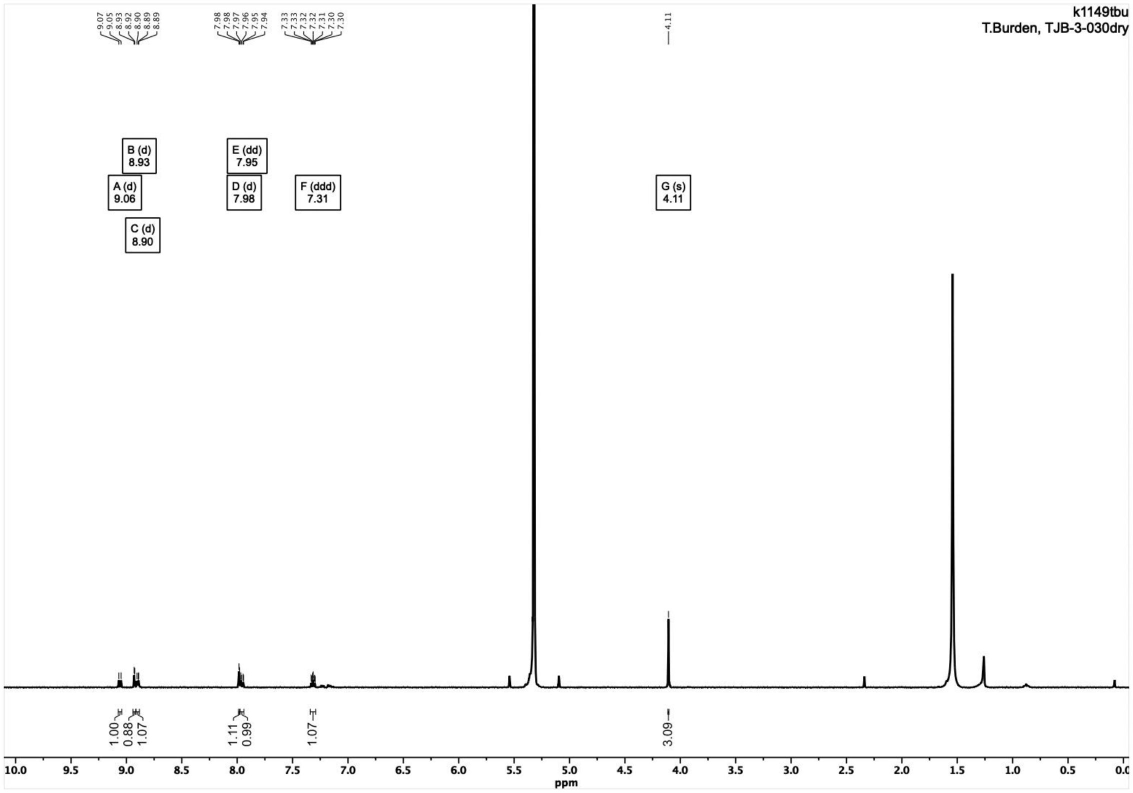

Figure S80 ^{13}C NMR spectrum of 6-(*6*-NO_2,*8*-OMe)

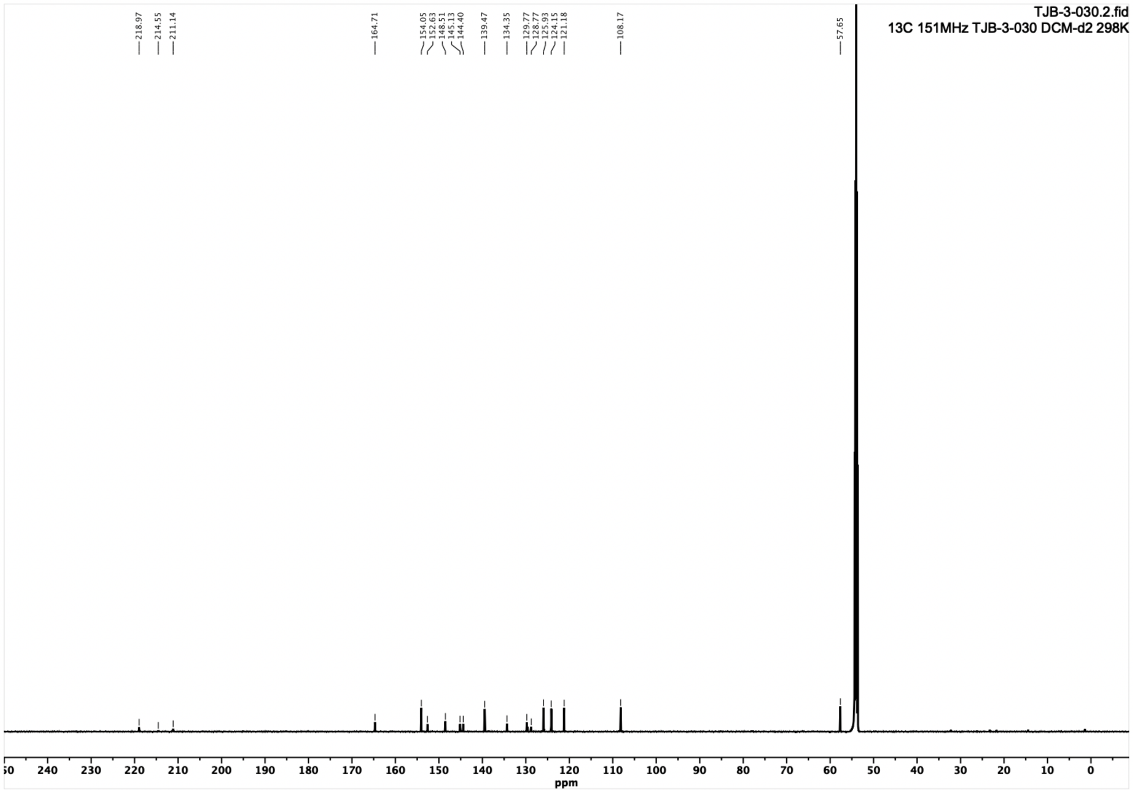

Figure S81 ^{1}H NMR spectrum of 5a-(*6*-NO_2,*8*-OMe)

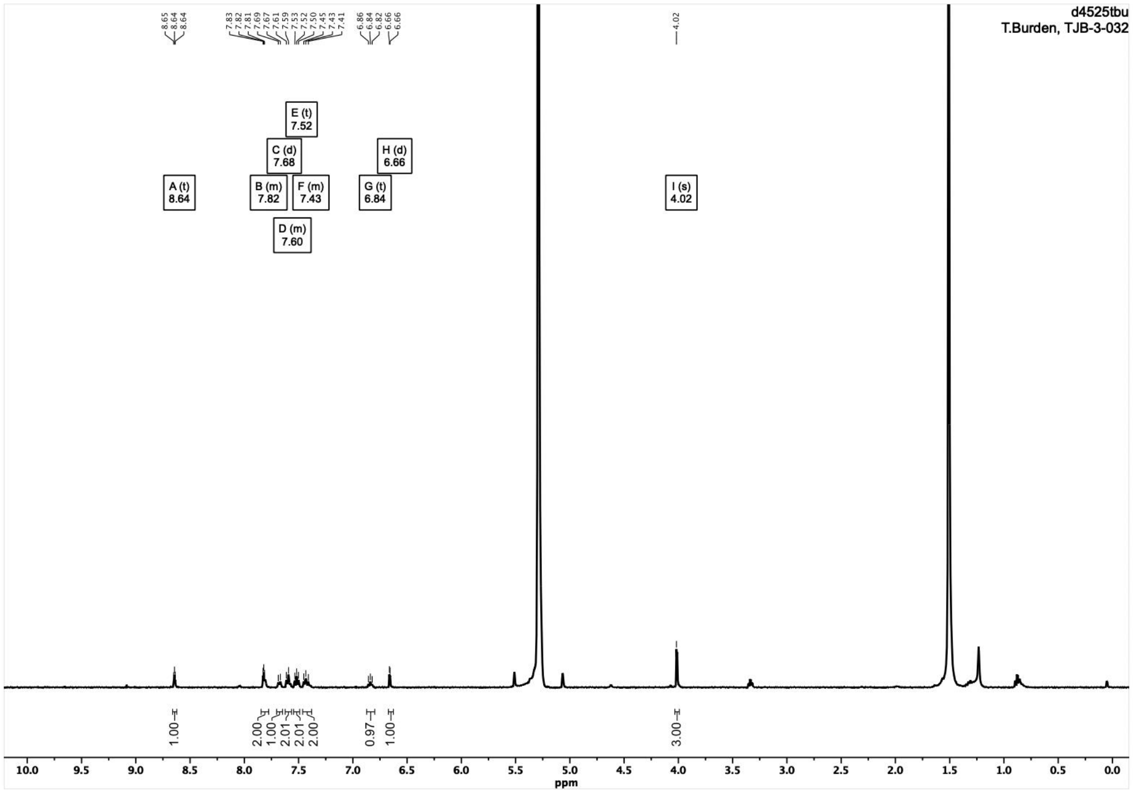

Figure S82 ^{13}C NMR spectrum of 5a-(*6*-NO_2,*8*-OMe)

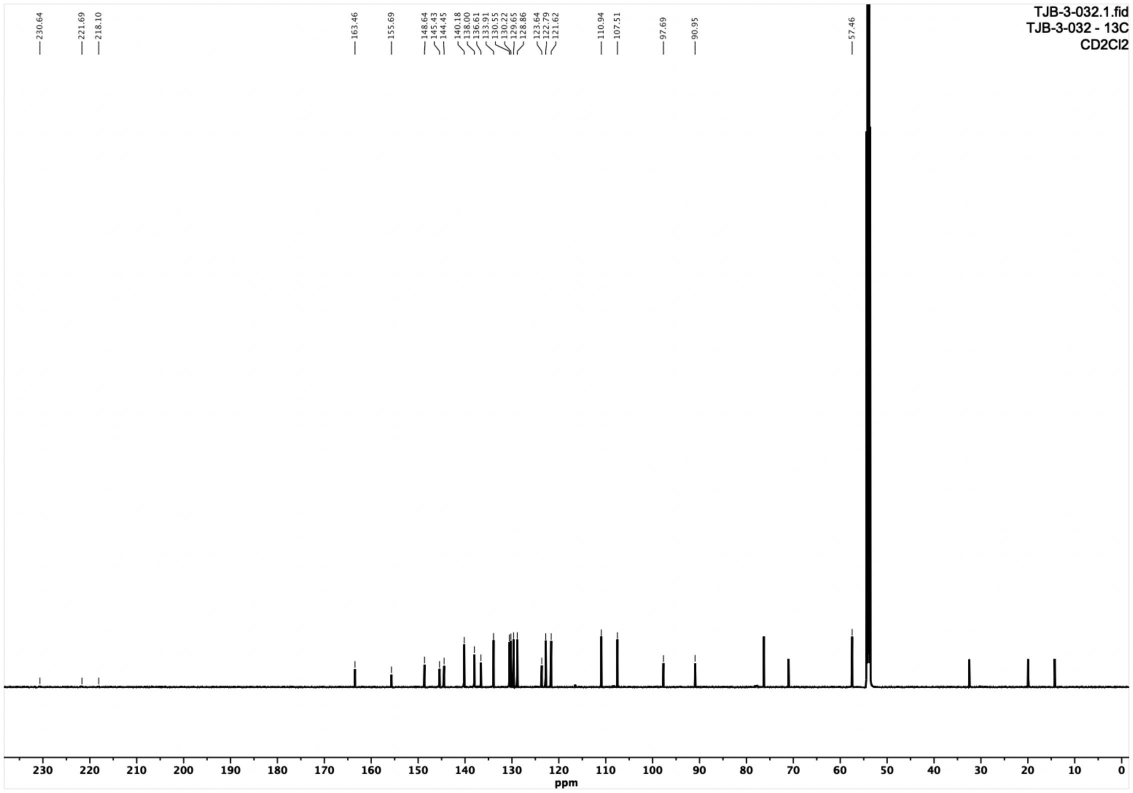

Figure S83 ^{1}H NMR spectrum of 4-(6,7-methylenedioxy)

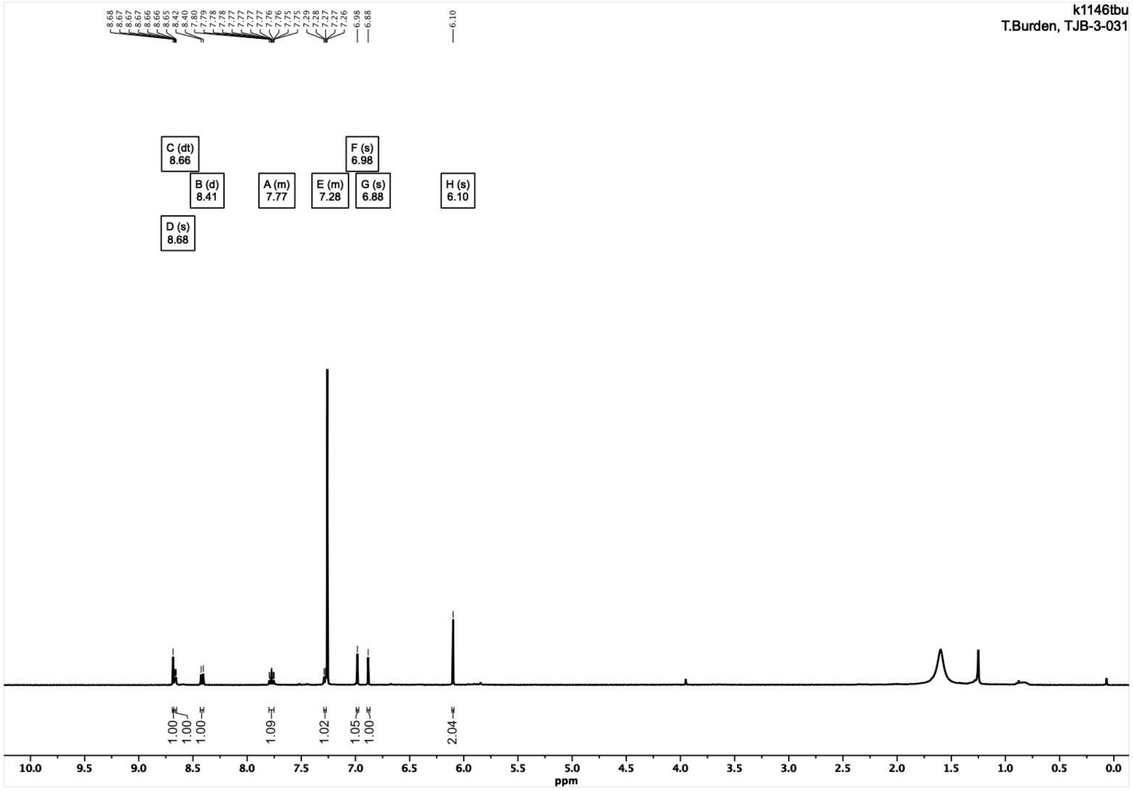

Figure S84 ^{13}C NMR spectrum of 4-(6,7-methylenedioxy)

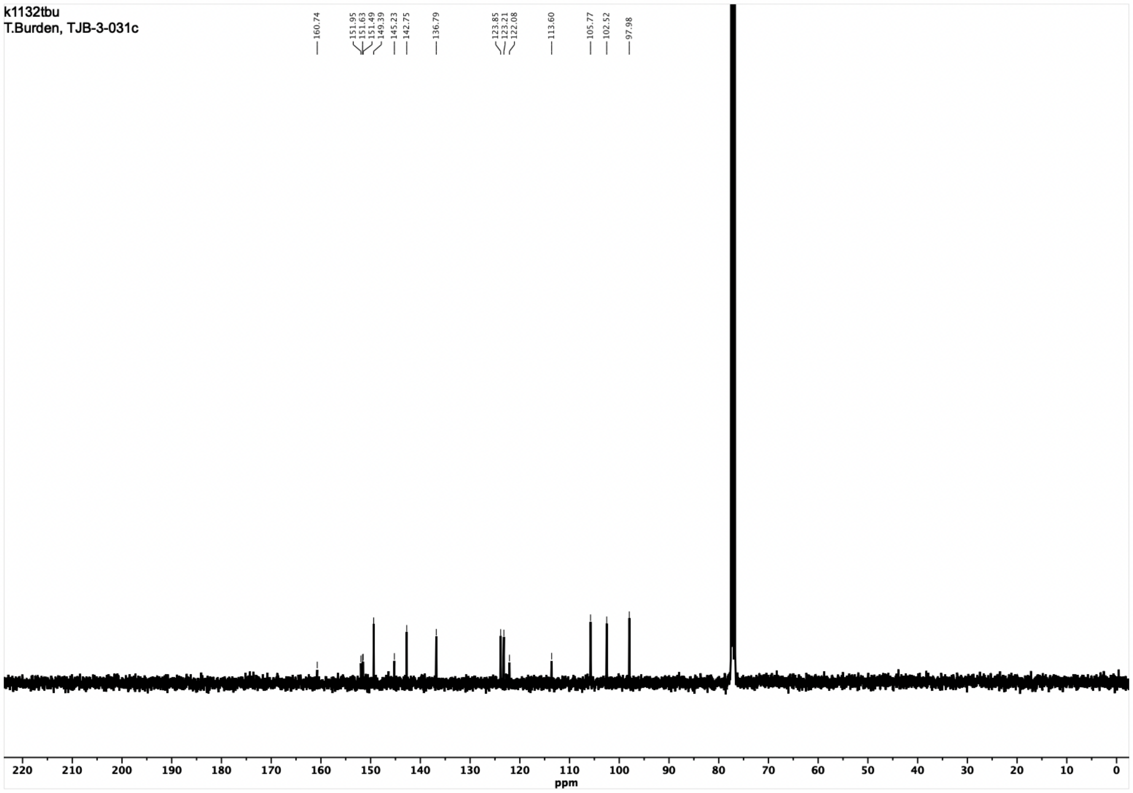

Figure S85 ^{1}H NMR spectrum of 6-(6,7-methylenedioxy)

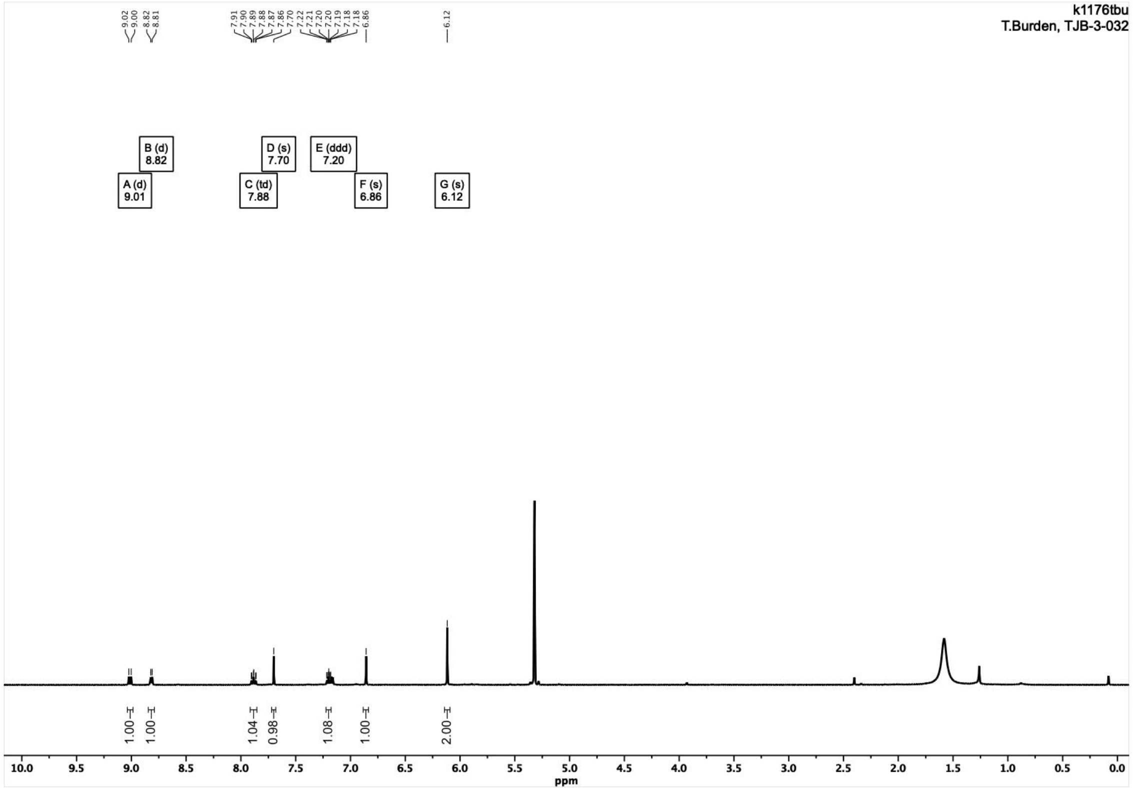

Figure S86 ^{13}C NMR spectrum of 6-(6,7-methylenedioxy)

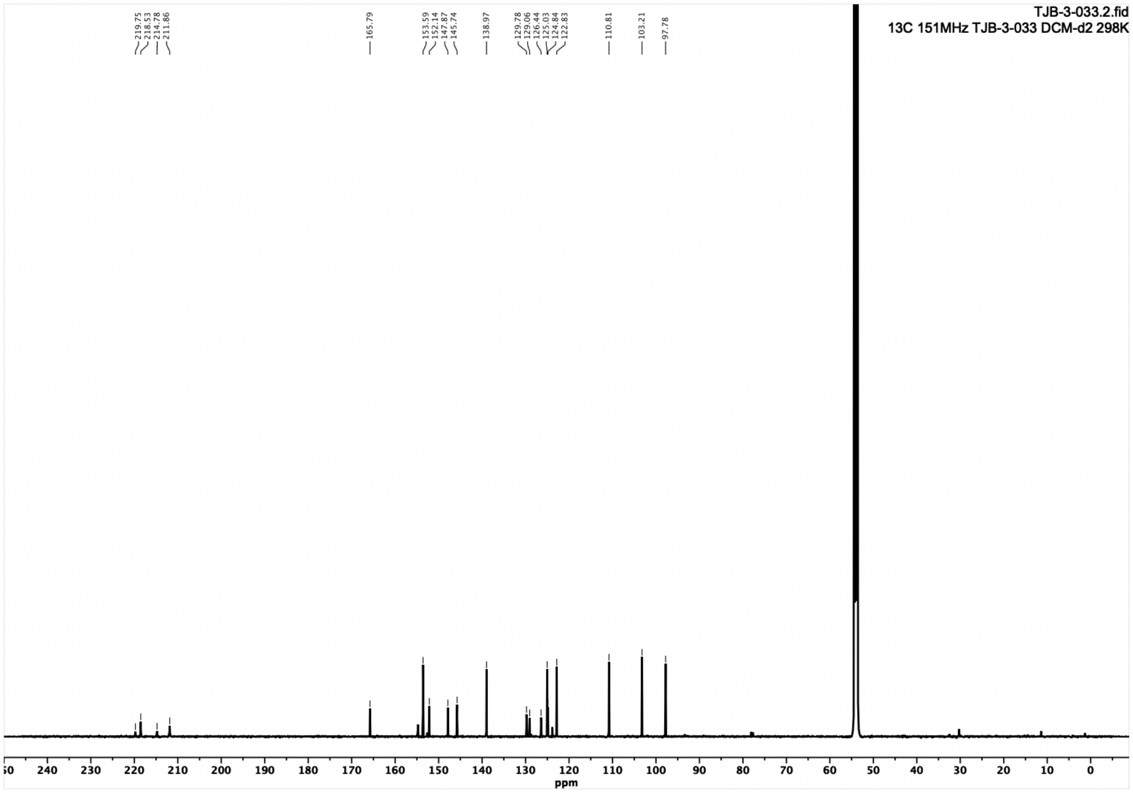

Figure S87 ^{1}H NMR spectrum of 5a-(6,7-methylenedioxy)

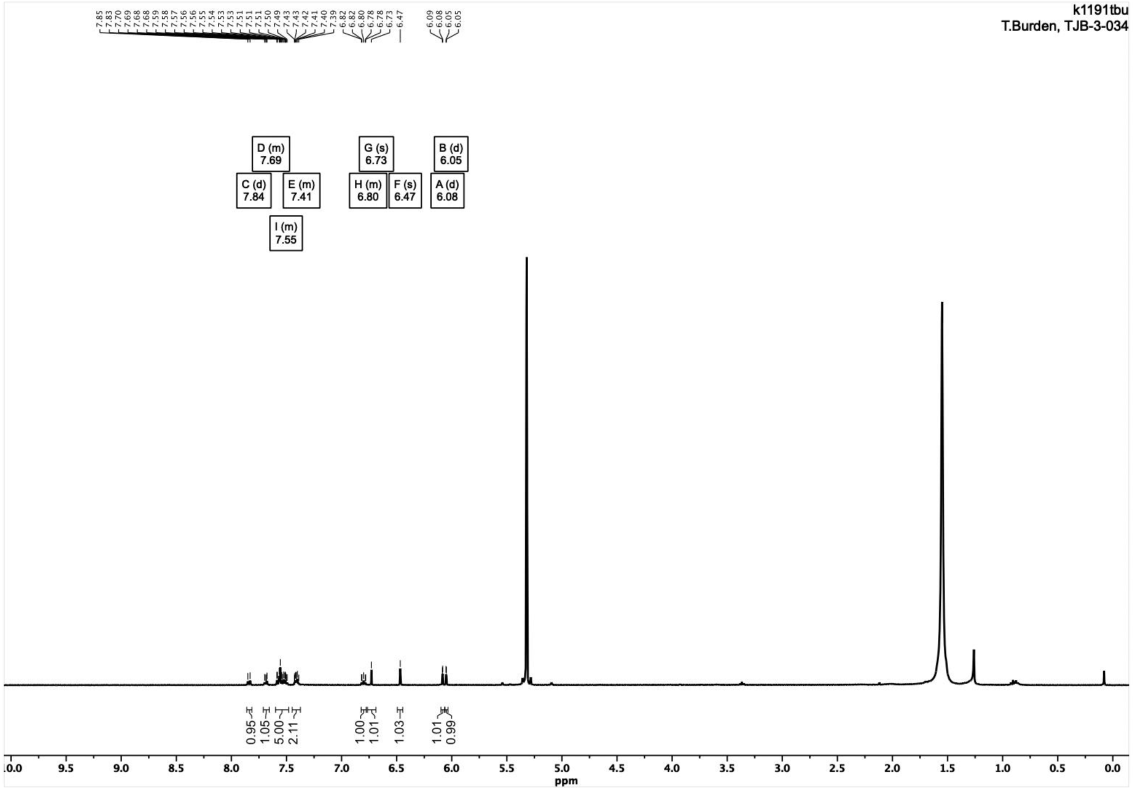

Figure S88 ^{13}C NMR spectrum of 5a-(6,7-methylenedioxy)

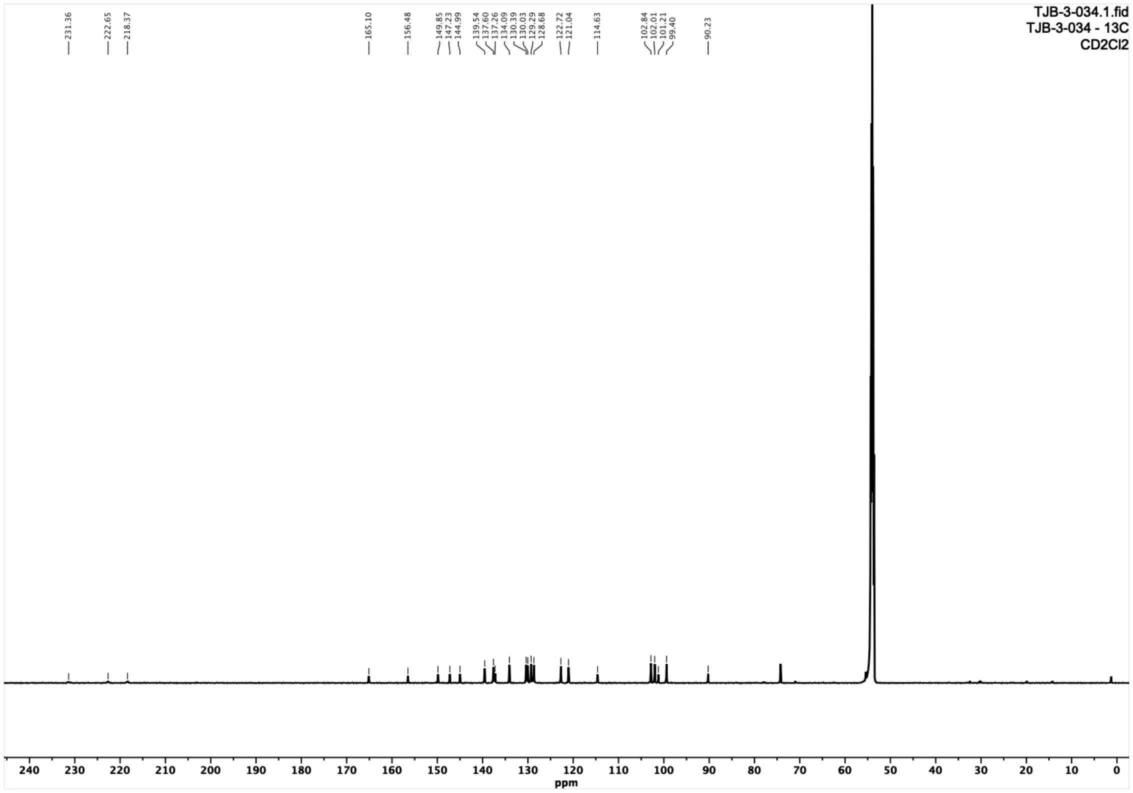

Figure S89 ^{1}H NMR spectrum of 11a

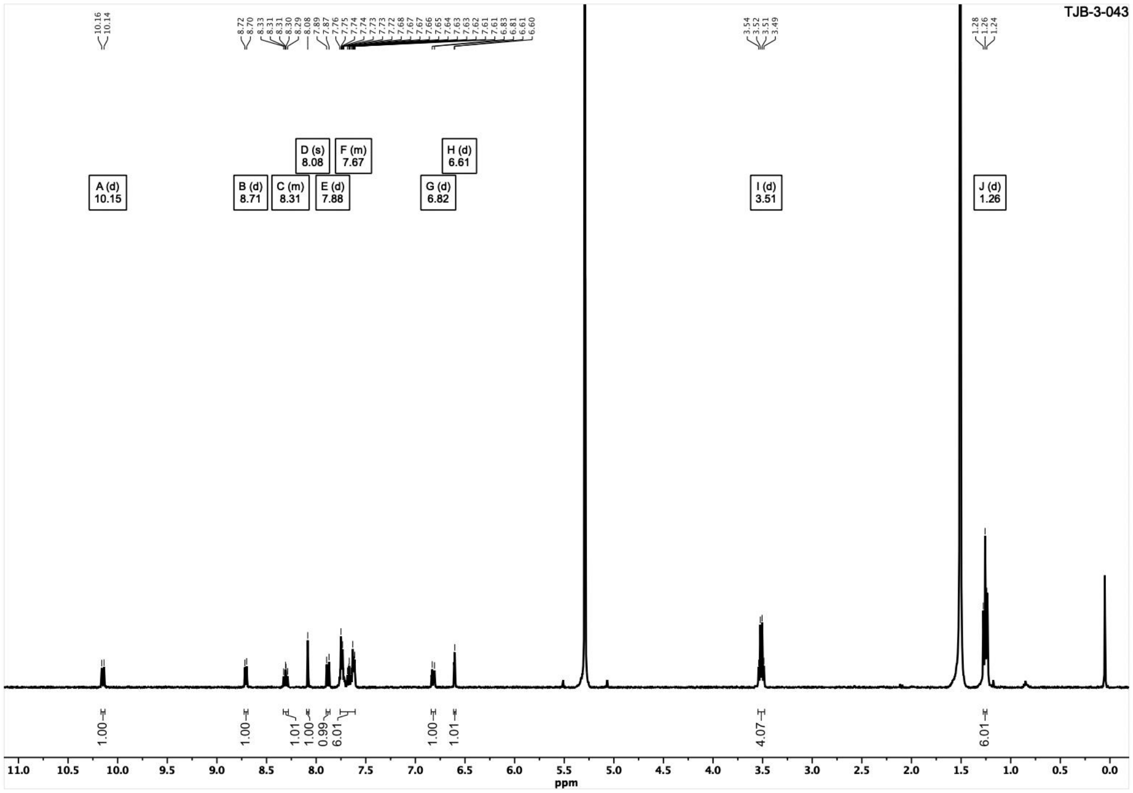

Figure S90 ^{19}F NMR spectrum of 11a

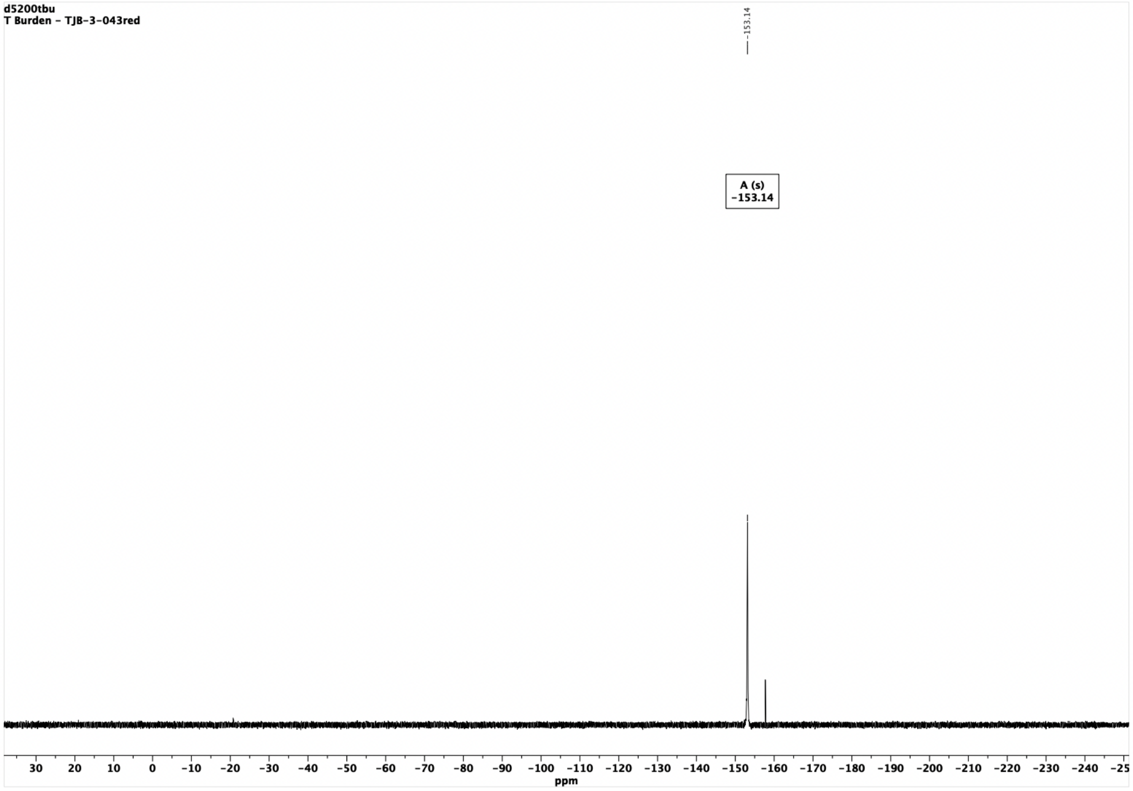

Figure S91 ^{13}C NMR spectrum of 11a

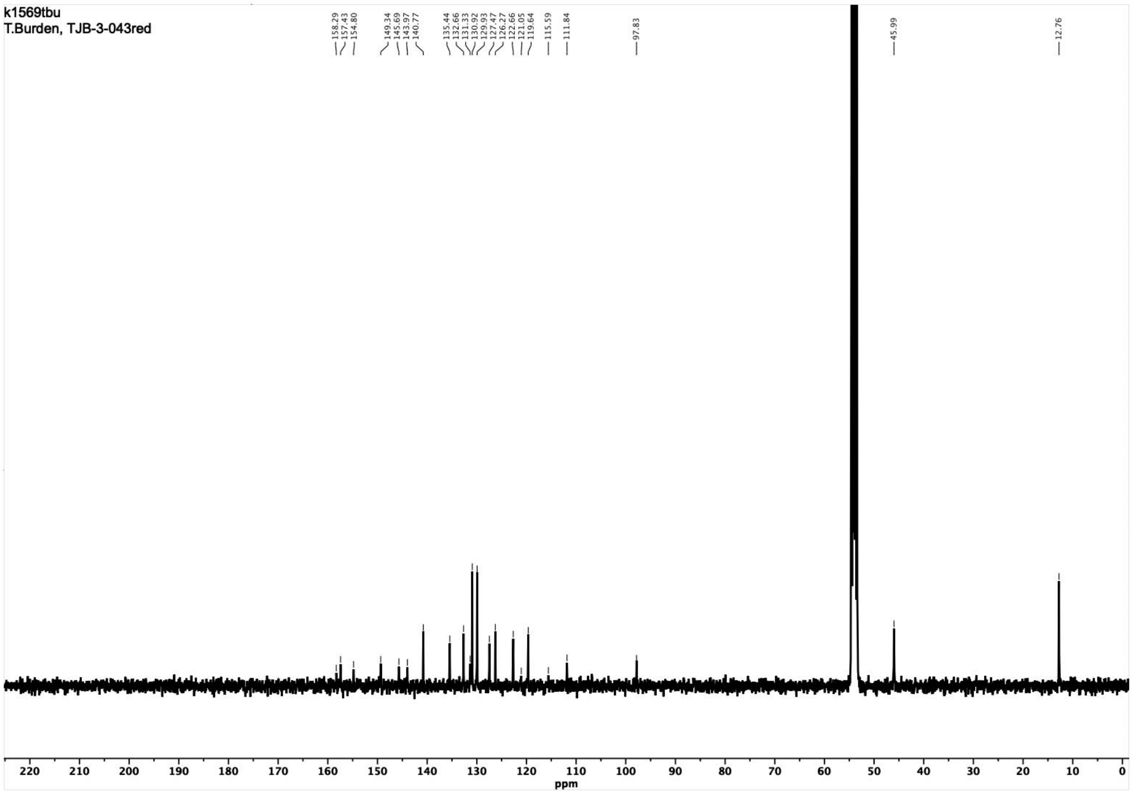

Figure S92 ^{1}H NMR spectrum of 12a

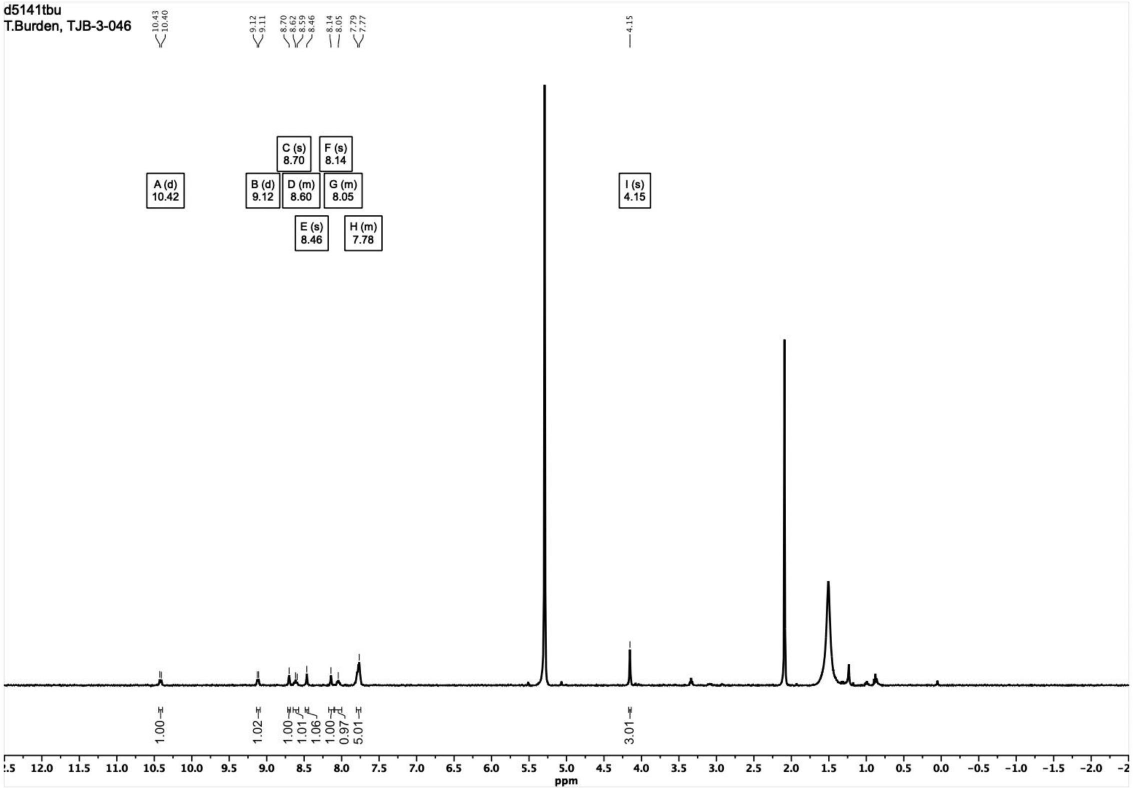

Figure S93 ^{19}F NMR spectrum of 12a

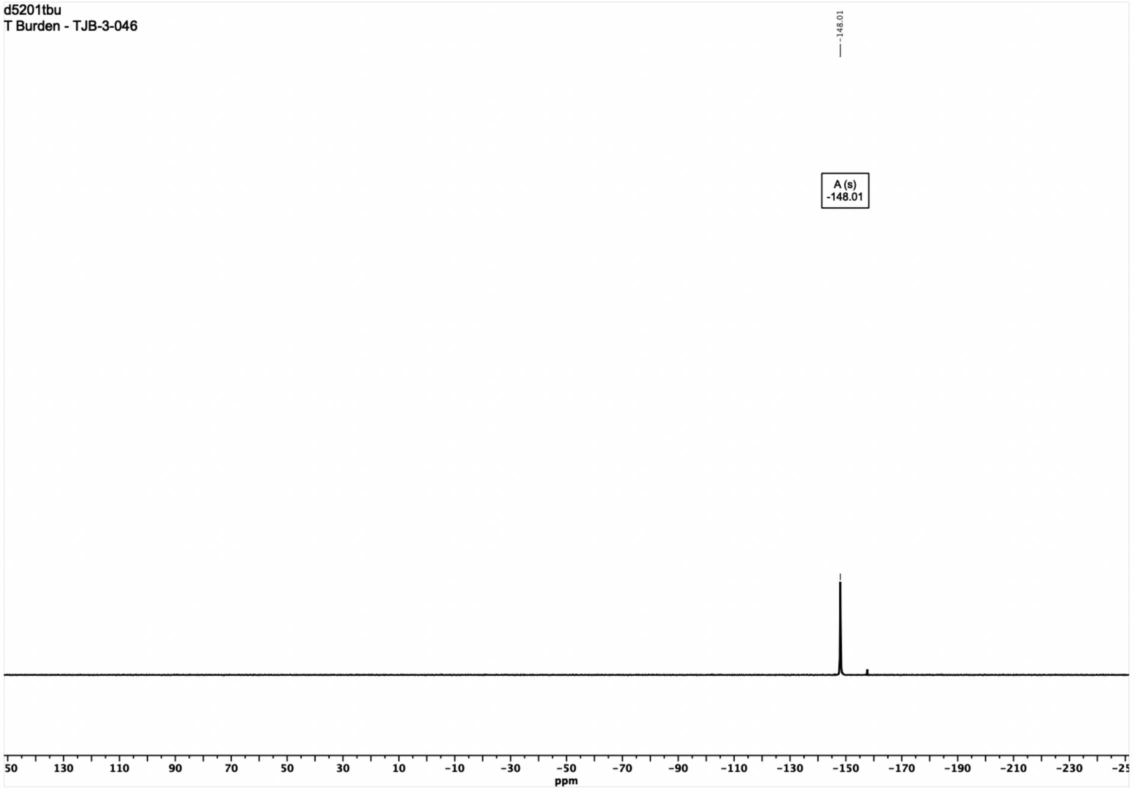

Figure S94 ^{13}C NMR spectrum of 12a

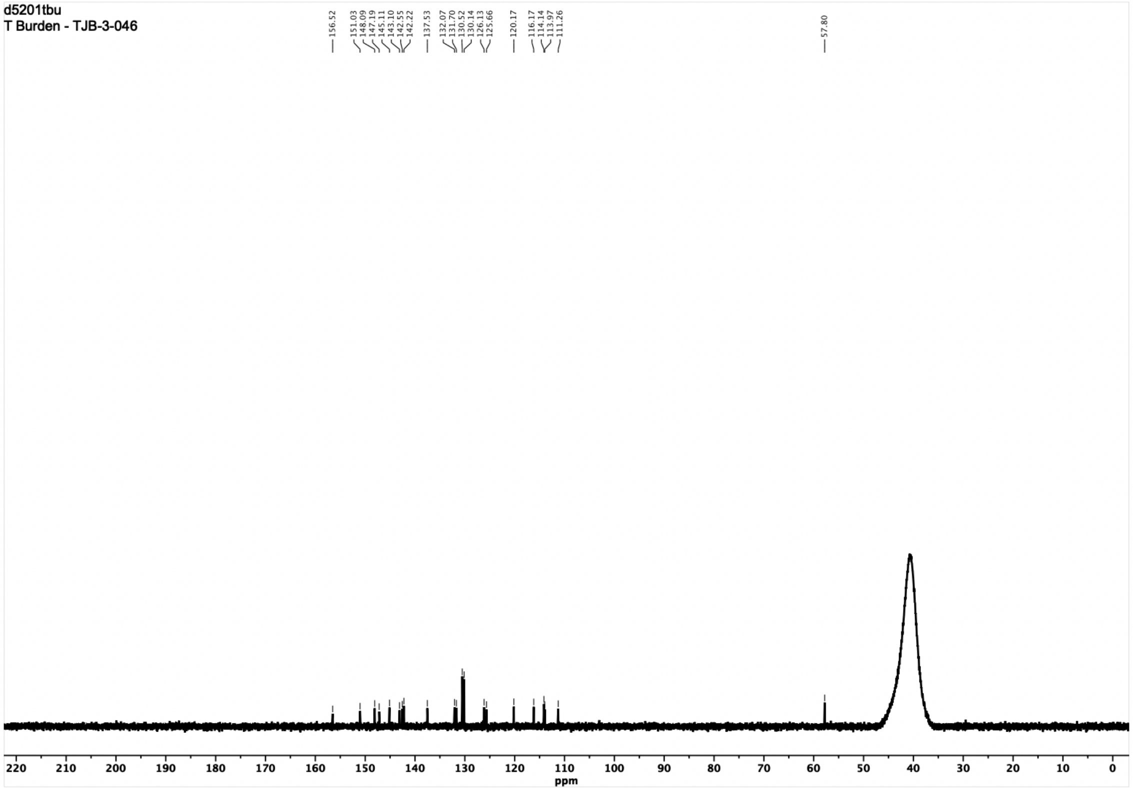

Figure S95 ^{1}H NMR spectrum of 13a

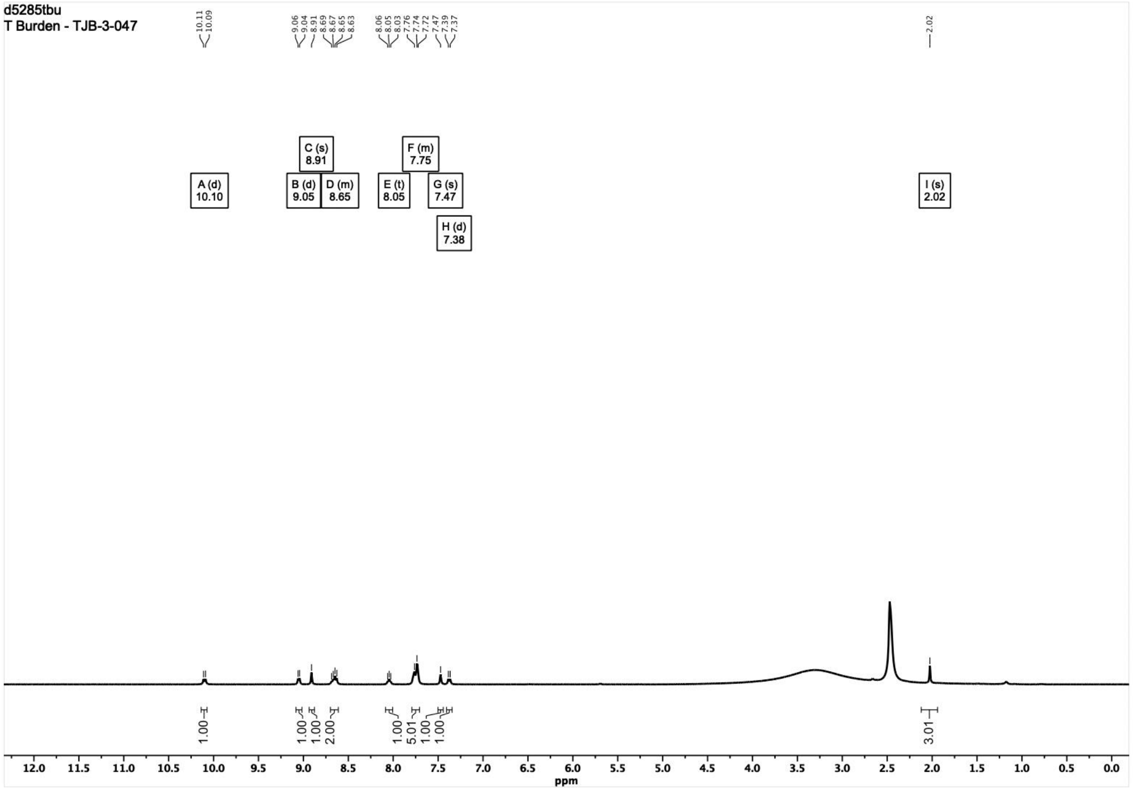

Figure S96 ^{19}F NMR spectrum of 13a

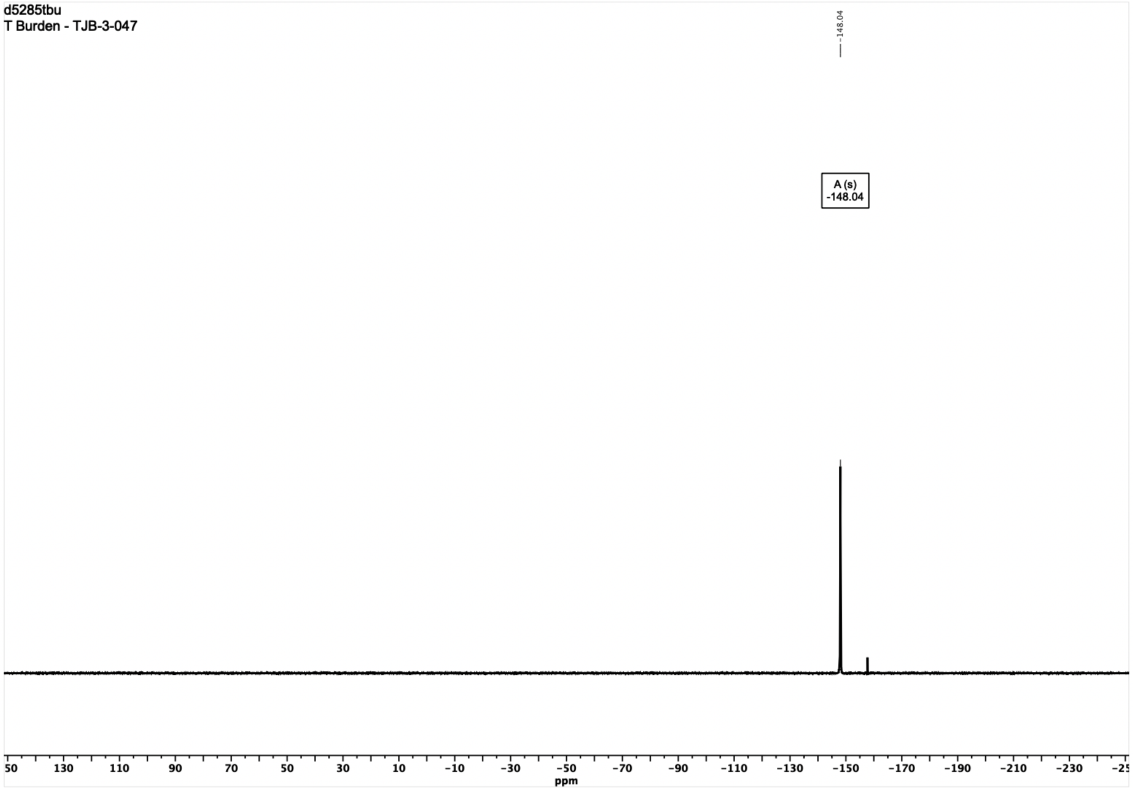

Figure S97 ^{13}C NMR spectrum of 13a

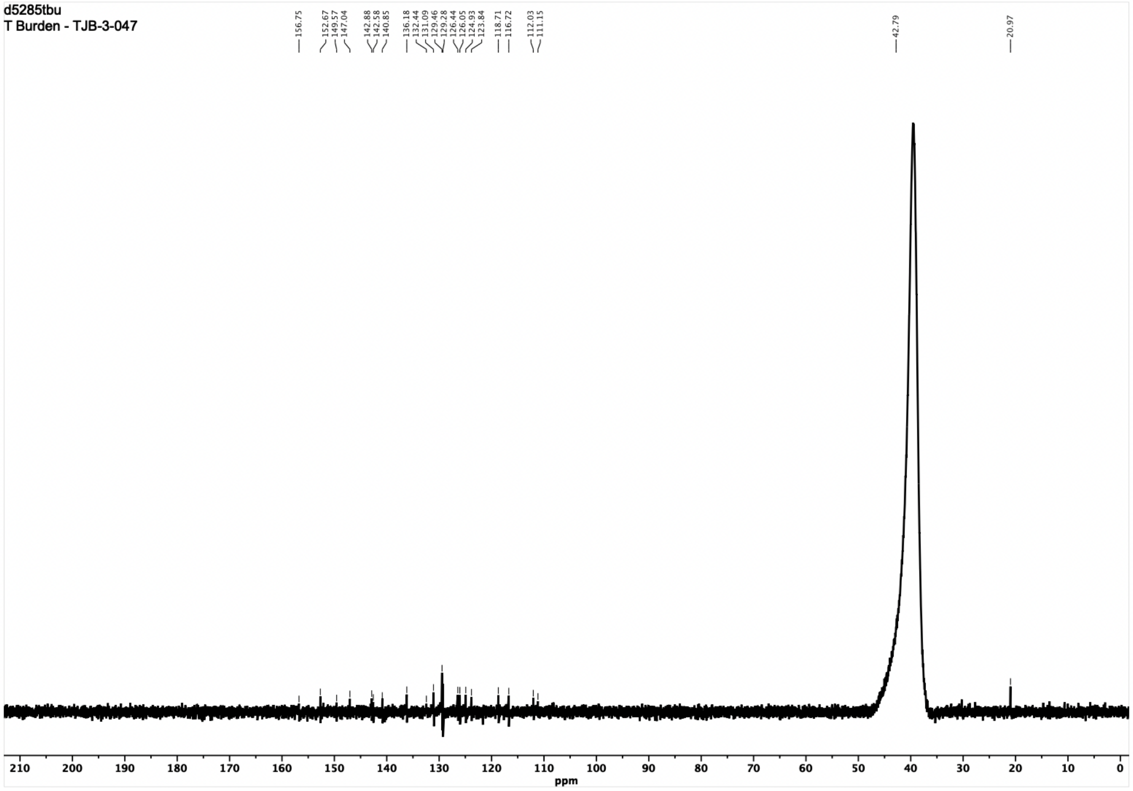

Figure S98 ^{1}H NMR spectrum of Benzyl rhenium(I) pentacarbonyl

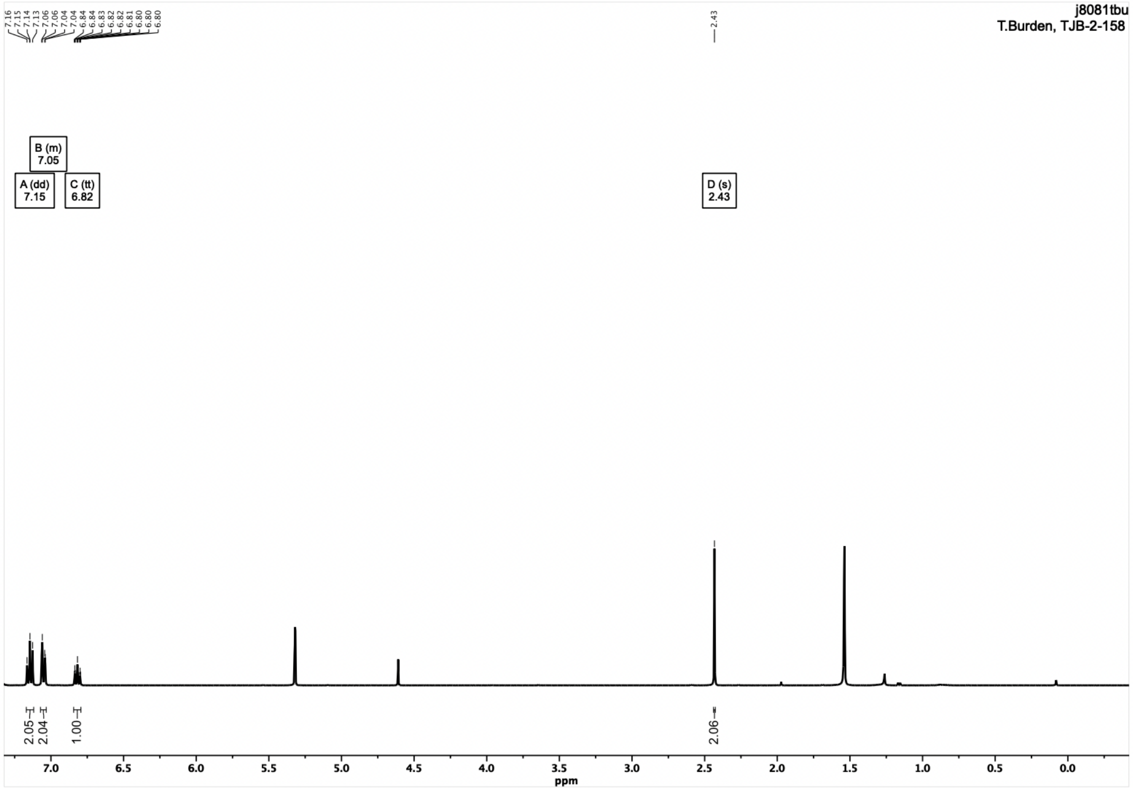

Figure S99 ^{13}C NMR spectrum of Benzyl rhenium(I) pentacarbonyl

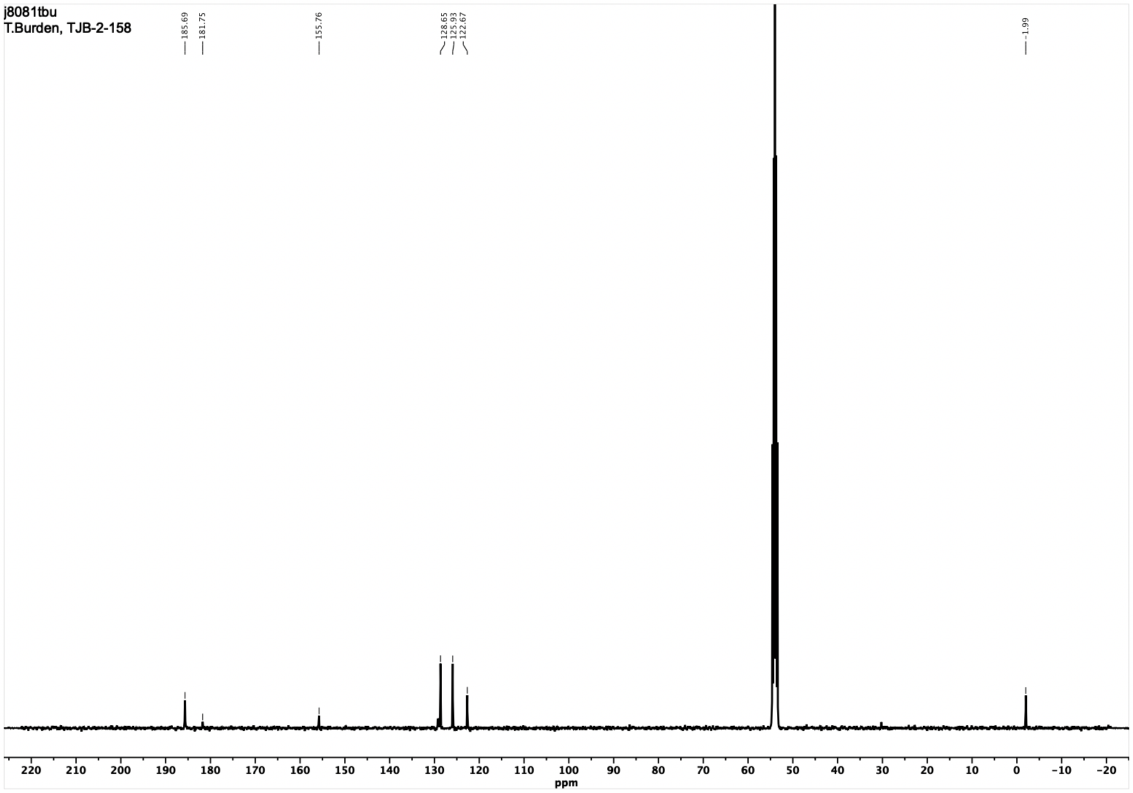

Figure S100 ^{1}H NMR spectrum of 6’-(H)

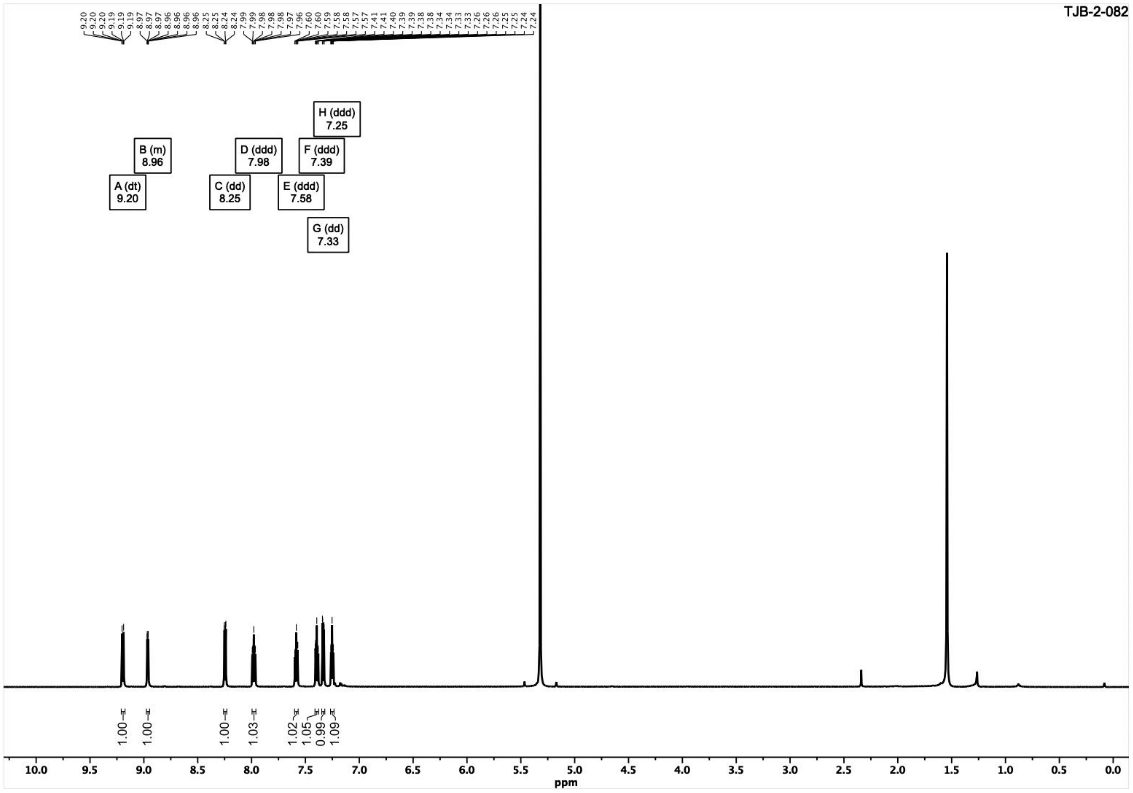

Figure S101 ^{13}C NMR spectrum of 6’-(H)

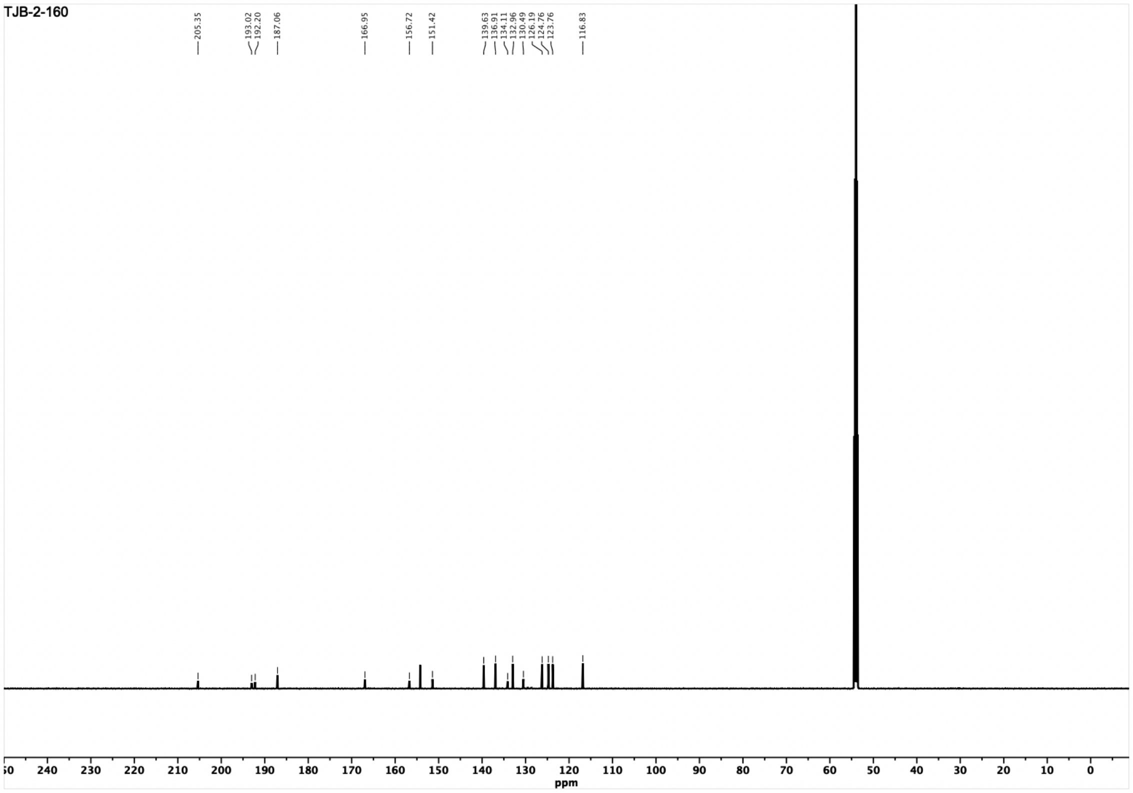

5.0 References

[1] G. M. Greetham, P. M. Donaldson, C. Nation, I. V. Sazanovich, I. P. Clark, D. J. Shaw, A. W. Parker, M. Towrie, *Appl. Spectrosc.* **2016**, *70*, 645–653.
[2] M. R. Pollard, G. M. Greetham, ULTRA View Data Analysis, 2; STFC: 2012.
[3] OriginPro; OriginLab Corporation: Northampton, MA, 2019.
[4] L. A. Hammarback, A. Robinson, J. M. Lynam, I. J. S. Fairlamb, *J. Am. Chem. Soc.* **2019**, *141*, 2316–2328.
[5] L. A. Hammarback, I. P. Clark, I. V. Sazanovich, M. Towrie, A. Robinson, F. Clarke, S. Meyer, I. J. S. Fairlamb, J. M. Lynam, *Nat. Catal.* **2018**, *1*, 830–840.
[6] (a) P. Császár, P. Pulay, *J. Mol. Struct.* **1984**, *114*, 31-34. (b) R Ahlrichs, M. Bär, M. Häser, H. Horn, C. Kölmel, *Chem. Phys. Lett.* **1989**, *162*, 165-169 (c) P. Deglmann, F. Furche, R. Ahlrichs, *Chem. Phys. Lett*. **2002**, *362*, 511-518. (d) P. Deglmann, K. May, F. Furche, R. Ahlrichs, *Chem. Phys. Lett*. **2004**, *384*, 103-107. (e) K. Eichkorn, O. Treutler, H. Öhm, M. Häser, R. Ahlrichs, *Chem. Phys. Lett*. **1995**, *242*, 652-660. (f) K. Eichkorn, F. Weigend, O. Treutler, R Ahlrichs, *Theor. Chem. Acc.* **1997**, *97*, 119-124. (g) O. Treutler, R, Ahlrichs, *J. Chem. Phys.* **1995**, *102*, 346-354. (h) M. von Arnim, R. Ahlrichs, *J. Chem. Phys.* **1999**, *111*, 9183-9190.
[7] S. Grimme, S. Ehrlich, L. Goerigk, *J. Comput. Chem.* **2011**, *32*, 1456-1465.
[8] S. Grimme, J. Antony, S. Ehrlich, H. Krieg, *J. Chem. Phys.* **2010**, *132*, 154104-154118.
[9] A. Klamt, G. Schuurmann, *J. Chem. Soc., Perkin Trans. 2* **1993**, 799-805.
